# Taxonomy and molecular evaluation of the Halosphaeriaceae (Microascales, Hypocreomycetidae)

**DOI:** 10.3897/mycokeys.135.187540

**Published:** 2026-06-25

**Authors:** Monika C. Dayarathne, E. B. Gareth Jones, Ali H. Bahkali, Bandarupalli Devadatha, Kevin D. Hyde, Mohamed A. Abdel-Wahab, Mark S. Calabon, Pedro Correia, Faten A. Abdel-Aziz, Mahmoud S. Bakhit, Jariya Sakayaroj, Chasika Prematunga, Maria F. Caeiro, Egídia Azevedo, Raheleh Asghari, Carlo Chris S. Apurillo, Amuhenage Tharindu Bhagya, Herbert Dustin Aumentado, Samhita Mukhopadhyay, Chayanard Phukhamsakda, Yanpeng Chen, Sajeewa S. Maharachchikumbura, Sheng-Yu Guo, Ka-Lai Pang

**Affiliations:** 1 Department of Plant Science, Agriculture Building, University of Manitoba, Winnipeg, Manitoba R3T 2N2, Canada College of Arts and Sciences, University of the Philippines Visayas Miagao Philippines; 2 Center of Excellence in Fungal Research, Mae Fah Luang University, Chiang Rai 57100, Thailand Center of Excellence in Fungal Research, Mae Fah Luang University Chiang Rai Thailand; 3 Department of Botany and Microbiology, King Saudi University, Riyadh, Saudi Arabia Department of Biotechnology, School of Life Sciences, Pondicherry University Pondicherry India; 4 Microbial Type Culture Collection and Gene Bank (MTCC), CSIR-Institute of Microbial Technology, Chandigarh, 160036, India CE3C – Centre for Ecology, Evolution and Environmental Changes, Departamento de Biologia (DBio), Faculdade de Ciências da Universidade de Lisboa (FCUL) Lisboa Portugal; 5 Fungal Biotechnology Lab, Department of Biotechnology, School of Life Sciences, Pondicherry University, Kalapet, Pondicherry-605014, India CHANGE – Global Change and Sustainability Institute, Departamento de Biologia (DBio), Faculdade de Ciências da Universidade de Lisboa (FCUL) Lisboa Portugal; 6 Department of Botany and Microbiology, Faculty of Science, Sohag University, Sohag 82524, Egypt Institute for Advanced Study, Shenzhen University Shenzhen China; 7 Division of Biological Sciences, College of Arts and Sciences, University of the Philippines Visayas, Miagao, Iloilo, 5023 Philippines College of Life Sciences and Oceanography, Shenzhen University Shenzhen China; 8 CE3C – Centre for Ecology, Evolution and Environmental Changes, Departamento de Biologia (DBio), Faculdade de Ciências da Universidade de Lisboa (FCUL), Campo Grande 1749-016, Lisbon, Portugal Department of Botany and Microbiology, King Saudi University Riyadh Saudi Arabia; 9 CHANGE – Global Change and Sustainability Institute, Departamento de Biologia (DBio), Faculdade de Ciências da Universidade de Lisboa (FCUL), Campo Grande 1749-016, Lisbon, Portugal Agriculture Building, University of Manitoba Manitoba Canada; 10 Department of Biology School of Science, Walailak University, 222 Thaiburi, Thasala district, Nakhon Si Thammarat 80161, Thailand Faculty of Science, Sohag University Sohag Egypt; 11 Archaeal Biology Center, Institute for Advanced Study, Shenzhen University, Shenzhen, China Institute of Marine Biology and Center of Excellence for the Oceans, National Taiwan Ocean University Keelung Taiwan; 12 College of Life Sciences and Oceanography, Shenzhen University, Shenzhen 518060, China Department of Biology School of Science, Walailak University Nakhon Si Thammarat Thailand; 13 School of Life Science and Technology, University of Electronic Science and Technology of China, Chengdu, China School of Life Science and Technology, University of Electronic Science and Technology of China Chengdu China; 14 Institute of Marine Biology and Center of Excellence for the Oceans, National Taiwan Ocean University, 2 Pei-Ning Road, Keelung 202301, Taiwan Microbial Type Culture Collection and Gene Bank (MTCC), CSIR-Institute of Microbial Technology Chandigarh India

**Keywords:** Ascomycota, ecology, life below water, new taxa, marine, Sordariomycetes, taxonomy

## Abstract

This study re-evaluates the classification of the Halosphaeriaceae (Microascales, Hypocreomycetidae) based on phylogenetic analyses of 18S and 28S regions of rDNA and protein coding genes (rDNA, RPB1, RPB2, TEF1-ɑ, MCM7) along with comprehensive morphological comparisons. Halosphaeriaceae are worldwide in distribution and commonly found in marine habitats. A natural classification was not possible when the family was introduced, since it predated molecular phylogenetic studies; therefore, morphology-based descriptions only were provided. This study recognises 77 genera with 202 species which includes seven new genera (*Neoaniptodera*, *Neogesasha*, *Neohalosarpheia*, *Pangia*, *Remisporiopsis*, *Sheareromyces*, *Shiiraspora*), and one new species *Ocostaspora japonica*. The new taxonomic combinations include *Halosarpheia australiensis*, *Halosphaeriopsis alopallonella*, *Neoaniptodera juncicola*, *Neogesasha mangrovei*, *Neohalosarpheia marina*, *Pangia limnetica*, *Remisporiopsis macrocephala*, *R. quadri-remis*, *R. spitsbergenensis*, *R. stellatus*, *R. submersa*, *Sheareromyces aquibella*, *Shiiraspora salsuginosa* and *Thalassogena unicellularis*. Divergent time estimation of Halosphaeriaceae, based on combined 18S, 28S rDNA, RPB1, RPB2, TEF1-ɑ, MCM7 sequences, indicates that the family originated in the Paleozoic at ~545 MYA. Ecologically, Halosphaeriaceae species occur widely on different substrates and play an important role in the recycling of complex organic material. They are often early colonizers of wood in the marine environment and are the source of a wide range of enzymes and antimicrobials.

## Introduction

The family Halosphaeriaceae was introduced by Muller et von Arx ex Kohlmeyer (1972) with 13 genera in the order Sphaeriales, and characterized by perithecial ascomata, necks with periphyses, and catenophyses that easily deliquesce, early deliquescing unitunicate asci lacking an apical pore and hyaline, one septate ascospores, often with elaborate appendages (Jones et al. 1983a). They are mainly marine, with a few species found in freshwater and brackish water habitats. Kohlmeyer and Kohlmeyer (1979) accepted 15 genera including *Lindra* and *Lulworthia*, all based on morphological criteria. Subsequently, the genera *Lindra* and *Lulworthia* were referred to the order Lulworthiales by Kohlmeyer et al. (2000) based on a phylogenetic and morphological study. The current study includes 77 genera and 202 species, supported by detailed descriptions of the genera with data on their phylogeny, ecology, distribution and evolution. Resolution of taxa in the Halosphaeriaceae is frequently problematic; as historical herbarium material has been totally exhausted or is not accessible, lacking cultures and sequence data. Halosphaeriaceae was validly published by Kohlmeyer (1972) and assigned as a single family in the new order Halosphaeriales by Hawksworth and Eriksson (1986). Subsequently, the family was placed in the Microascales, an order in the subclass Hypocreomycetidae (class Sordariomycetes), by Benny and Kimbrough (1980). Currently, the order comprises nine families, viz., Ceratocystidaceae, Chadefaudiellaceae, Cornuvesicaceae, Gondwanamycetaceae, Graphiaceae, Halosphaeriaceae, Microascaceae, Synnematotriadelphiaceae and Triadelphiaceae (Maharachchikumbura et al. 2016; Réblová et al. 2016; Hyde et al. 2020b, 2024; Thiyagaraja et al. 2025). Microascales has a stem age of 166 MYA and is well supported in both phylogenetic and MTC trees (Hongsanan et al. 2017). Later on, based on scanning and transmission electron microscopic studies of ascospore appendage morphology and ontogeny, several genera were proposed, such as *Bovicornua*, *Kohlmeyeriella*, *Ondiniella* and *Marinospora*, while others were re-instated, e.g. *Antennospora*, *Arenariomyces* and *Halosphaeriopsis* (Jones et al. 1983a, 1984; Jones 1995). Many new genera were introduced as the result of phylogenetic studies: *Gesasha*, *Kitesporella*, *Praelongicaulis*, *Toriella* and *Tubakiella* (Abdel-Wahab and Nagahama 2011; Sakayaroj et al. 2011; Jones et al. 2015). Recent introductions include: *Safagamyces* (Bakhit and Abdel-Wahab 2022), *Qarounispora* (Nourel-Din et al. 2022) and *Jinshana* (Manawasinghe et al. 2024). Correia et al. (2023) introduced the genera *Ajigaurospora*, *Corollosporella*, *Corollosporopsis*, *Garethelia*, *Honshuriella*, *Keraliethelia*, *Nakagariella*, *Paracorollospora*, *Shirahamella* and *Tokurathelia* but these are rejected in this monograph.

### Molecular phylogenetic studies

Extending from the single gene analysis to multigene phylogeny of the Halosphaeriaceae and Lulworthiaceae, several attempts were reported in resolving the phylogenetic relationships of these families by examining selected genera and related taxa (Spatafora et al. 1998; Abdel-Wahab et al. 2001; Anderson et al. 2001; Pang et al. 2003a, 2003b; Sakayaroj et al. 2005, 2011). Among them, Spatafora et al. (1998) proposed a terrestrial origin for the Halosphaeriaceae supported by 18S and 28S rDNA analysis. Subsequently, many other studies have been carried out emphasizing taxonomic revision of specific genera such as *Halosarpheia* and *Corollospora* rather than higher taxa (Kong et al. 2000; Abdel-Wahab et al. 2001; Anderson et al. 2001; Campbell et al. 2002; Pang et al. 2003a, 2003b; Correia et al. 2023), *Halosphaeria* (Pang et al. 2004a), *Lulworthia* and *Lindra* (Campbell et al. 2003; Koch et al. 2007), and *Antennospora* (Pang et al. 2008a). Jones et al. (2009) revised the family and Sakayaroj et al. (2011) provided a detailed account of its taxonomic status, the relationships between genera and morphological character evolution along with a multi-gene phylogeny. The higher-level classification of the halosphaeriaceous fungi remained contentious with Kohlmeyer (1972) assigning this family to the new monotypic order Halosphaeriales. Spatafora et al. (1998) demonstrated that the order Halosphaeriales was polyphyletic and comprised two distinct lineages and this was supported by Chen et al. (1999). The first clade of Halosphaeriales included eleven genera, and placed in the order Microascales, whereas the second clade, with *Lulworthia* and *Lindra* species, was assigned to Lulworthiaceae in Lulworthiales (Kohlmeyer et al. 2000), and upgraded to subclass Lulworthiomycetidae by Maharachchikumbura et al. (2015). Hibbett et al. (2007) and Schoch et al. (2007) also proposed assignment of the Halosphaeriaceae in the Microascales while others continued to retain the ordinal name Halosphaeriales (Zhang et al. 2006; Tang et al. 2007; Jones et al. 2009). However, Maharachchikumbura et al. (2015) and Jones et al. (2015) accepted the placement of the Halosphaeriaceae as one of the families in Microascales.

Circa 59% of halosphaeriaceous species are supported with molecular data (Jones et al. 2017). Moreover, classification of taxa from some genera such as *Bathyascus*, halosarpheia-like species, *Lignincola*, *Luttrellia*, *Magnisphaera* and *Nais* remains unresolved due to lack of fresh collections available for molecular study. Published new genera include *Alisea*, *Ascoglobospora*, *Jinshana*, *Safagamyces*, *Qarounispora*, while other species have been reassigned to new genera (*Aniptosporopsis*, *Paraaniptodera*, *Praelongicaulis*, *Shiiraspora*, *Toriella*, *Tubakiella*).

*Corollospora* was the most speciose and diverse genus in the family, but Correia et al. (2023) undertook a multiple sequence analysis of the ITS, 28S, and 18S rDNA with pairwise distance assessments, Bayesian, and maximum likelihood phylogenetic analyses of the genus. This resulted in the introduction of 10 new genera: *Ajigaurospora*, *Corollosporella*, *Corollosporopsis*, *Garethelia*, *Honshuriella*, *Keraliethelia*, *Nakagariella*, *Paracorollospora*, *Shirahamella* and *Tokurathelia*. These genera have been rejected in this monograph as *Corollospora* is an iconic genus with well-established morphological features with primary spines to the ascospores as well as polar and equatorial episporial fragments, while most are arenicolous (Jones et al. 1983a). Further new genera have been added as the result of studies undertaken in this manuscript: *Neoaniptodera*, *Neogesasha*, *Neohalosarpheia*, *Pangia*, *Remisporiopsis* and *Shiiraspora*.

### Ecology and mode of life

Halosphaeriaceous fungi are found predominantly in marine environments, with few transitional species in brackish water and freshwater habitats, e.g. *Aniptodera*, *Ascosacculus*. They occur as saprobes on algae, immersed or submersed on phanerogams, wood (many on mangrove wood), bark, leaves, and other cellulosic plant remains, grains of sand, or in calcareous shell fragments while they are rarely parasitic or symbiotic and are worldwide in their distribution (Jones 2011; Jones and Pang 2012b; Jones et al. 2013). Halosphaeriaceous fungi play a significant role in the decomposition of complex organic matter in the marine ecosystem by secreting wood-modifying enzymes (cellulases, laccases and ligninases) that degrade lignocelluloses (Jones and Hyde 1988). Hence, they contribute to the generation of particulate and dissolved organic matter where available in the food web of the oceanic environment (Mouzouras et al. 1988; Bucher et al. 2004; Jones and Pang 2010; Jones et al. 2019). Several studies showed that mangrove associated halosphaeriaceous fungi were capable of producing hydrolytic and oxidative enzymes thus indicating their probable role in the recycling of lignocellulose in mangroves (Bucher et al. 2004).

### General morphology of Halosphaeriaceae

Halosphaeriaceae retains its uniqueness by having perithecial ascomata, short or long necks (often with periphyses), catenophyses or early deliquescing paraphyses, unitunicate, thin-walled, early deliquescing, clavate to fusiform asci which vary or lack an apical apparatus and hyaline, unicellular to several septate ascospores with remarkable appendages and/or sheaths (Abdel-Wahab et al. 2001; Pang 2001; Sakayaroj et al. 2005, 2011; Bakhit and Abdel-Wahab 2022; Nourel-Din et al. 2022; Liu et al. 2024).

### Ascomata

Ascomata of Halosphaeriaceae are superficial or immersed to erumpent, often globose to subglobose (Jones et al. 2017), cylindrical or pyriform (Sakayaroj et al. 2011) in shape. They are hyaline or highly pigmented (Kohlmeyer 1971; Pang 2001), sometimes subiculate, scarcely stromatic. Ascomatal necks are relatively long but shorter necks are also found. Ostioles are papillate to long cylindrical, ostiolar canal usually filled with periphyses or pseudoparenchyma and ostioles are absent in very few taxa (Sakayaroj et al. 2011; Jones et al. 2017). Possession of periphyses and catenophyses provides the family with a morphological diversity and periphyses are found in a wide range of genera, such as *Halosphaeria* and *Remispora*. However, *Arenariomyces*, *Corollospora* and *Nereiospora* lack periphyses or which are deliquescent when mature (Jones et al. 2009). The genera *Aniptodera*, *Haligena*, *Halosarpheia*, *Lignincola*, *Marinospora*, *Morakotiella*, *Nais*, *Naufragella*, *Remispora* and *Tirispora* possess catenophyses which have been used to differentiate certain genera (Pang 2001; Sakayaroj et al. 2011; Jones et al. 2017).

### Asci

Halosphaeriaceae members have evanescent asci with or without an apical structure (e.g. *Ceriosporopsis halima*, *Sablicola chinensis*), persistent asci with retraction of the plasmalemma around the apical pore (e.g. *Aniptodera chesapeakensis*, *Saagaromyces abonnis*) (Baker et al. 2001; Pang et al. 2003a), or persistent asci that lack an apical pore (e.g. *Lignincola laevis*). Occurrences of various apical structures: apical pore, apical plate, apical thickening and retraction of the plasmalemma increase the inter- and intra-generic variations within this family (Sakayaroj et al. 2011). However, evanescent asci in the early deliquescing centrum and the passive ascospore release mechanism are the characteristic ascal features of this family (Spatafora et al. 1998). Length of ascus stalk is also an interesting feature in species delineation of Halosphaeriaceae; long stalks are present in *Praelongicaulis kandeliae*, *Saagaromyces abonnis*, and *S. ratnagiriensis*, while short stalk is a feature in *Halosarpheiasensu stricto* (Pang et al. 2003a).

### Ascospores

**Figure 1.**
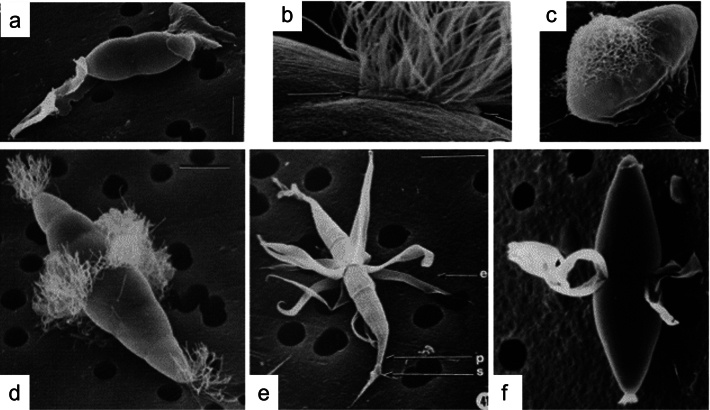
Scanning electron micrographs of different types of ascospore appendages in the Halosphaeriaceae. **a**. *Remispora maritima* with polar appendages; **b**. *Nereiospora cristata* hair-like equatorial appendages arising from a pad (arrowed); **c**. *Nimbospora effusa* with sticky appendage; **d**. *Nereiospora comata* with hair-like polar and equatorial appendages; **e**. *Corollospora intermedia* with polar spine peritrichous appendages apically and at the central septum (e=equatorial appendages, p=polar spine appendage, s=polar secondary appendages); **f**. *Halosphaeriopsis mediosetigera* with polar caps and lunate equatorial appendages. Scale bars: 10 mm (**a, c–f**); 1 mm (**b**).

**Figure 2.**
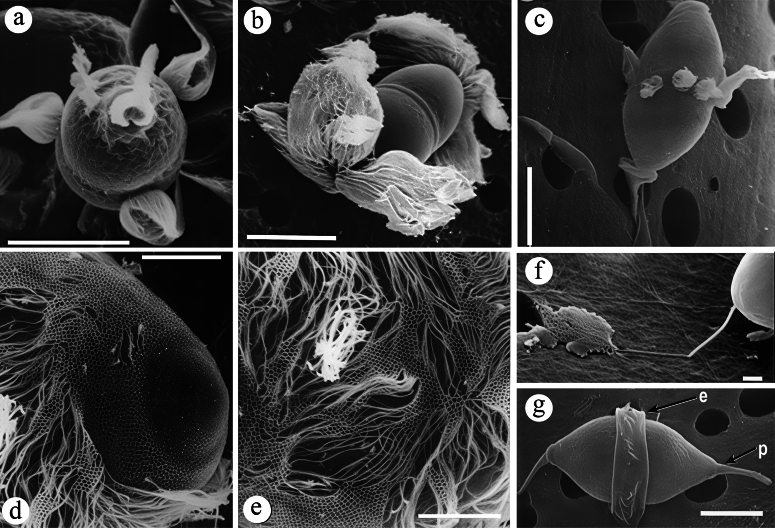
Scanning electron micrographs of different types of ascospore appendages in the Halosphaeriaceae. **a**. *Halosphaeria appendiculata* with cup-like appendages apically and at central septum; **b**. *Remisporiopsis quadri-remis* with stellate crown of appendages; **c**. *Ocostaspora apilongissima* with polar appendages and equatorial appendages; **d, e**. *Carbosphaerella leptosphaerioides* with net-like appendage sheath; **f**. *Halosarpheia* sp. with polar unfurling appendages; **g**. *Ondiniella torquata* with spine-like polar appendages and a gelatinous band around the central septum (g=equatorial band-like appendage, p=polar appendage). Scale bars: 10 mm (**a–c, g**); 1 mm (**d, e**).

**Figure 3.**
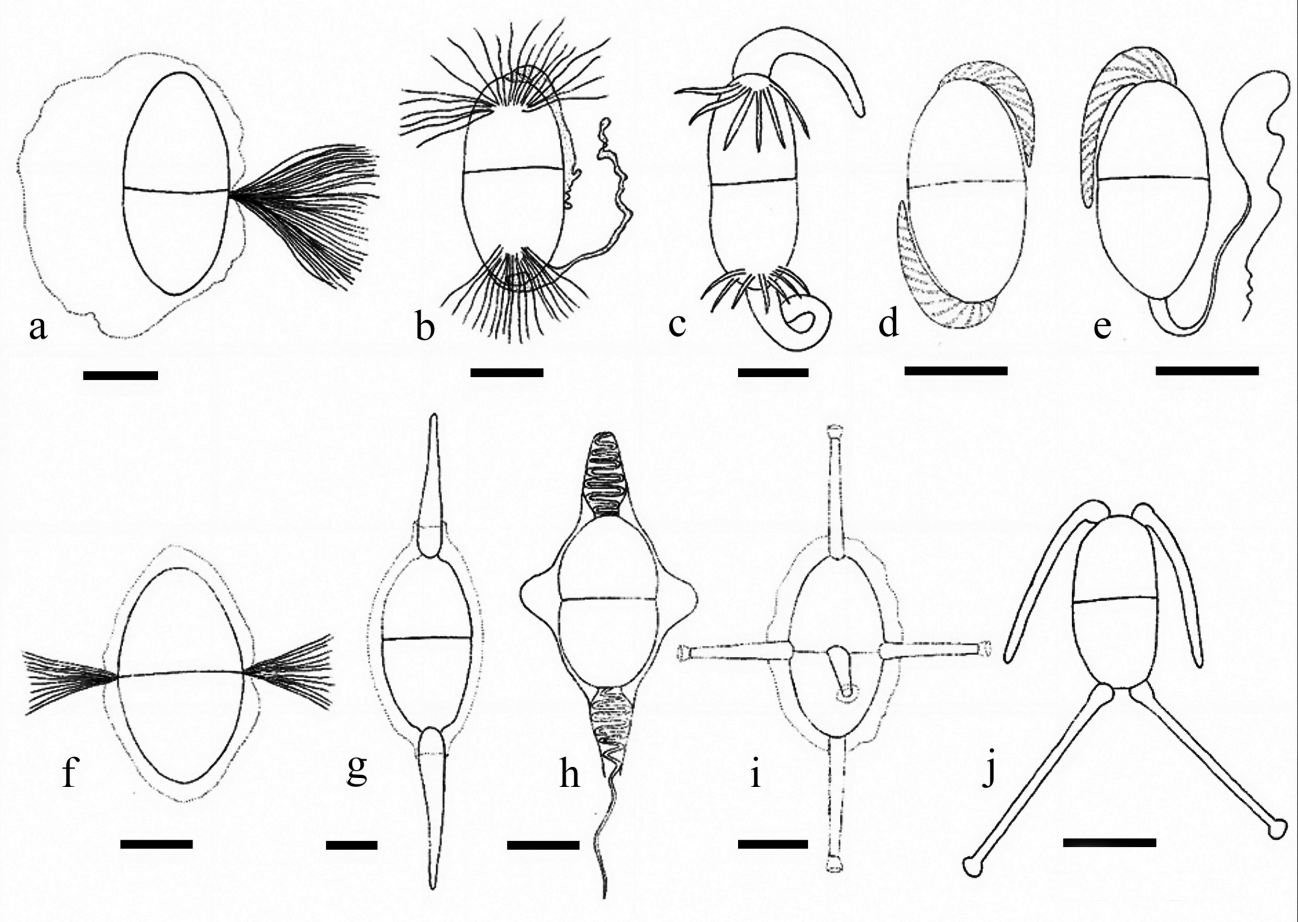
Line drawings of ascospores and their appendages in the *Halosphaeriaceae*. **a**. *Nimbospora effusa* with a gelatinous sheath and hair-like equatorial appendages (Redrawn from Koch 1982); **b**. *Nohea umiumi* (Kohlmeyer and Volkmann-Kohlmeyer 1991b); **c**. *Naufragella spinibarbata* both with subterminal hair-like appendages and long polar appendages (Koch 1989), **d, e**. *Saagaromyces abonnis* with hamate appendages that unfurl to form long thread-like polar appendages (Kohlmeyer 1984); **f**. *Nimbospora bipolaris* with a sheath and two equatorial appendages that are hair-like (Hyde and Jones 1985); **g**. *Cucullosporella mangrovei* with an apical collar from which an appendage is released that forms long thread-like strands (Hyde and Jones 1990); **h**. *Toriella tubulifera* with polar and equatorial appendages (Kohlmeyer 1960); **i**. *Marinospora calyptrata* with polar and equatorial appendages terminating in cup-like fragments of the exosporium (Cavaliere 1966a); **j**. *Arenariomyces trifurcatus* with sub-apical appendages (Jones et al. 1983a). Scale bars: 10 mm (**a–j**).

While ellipsoidal, hyaline ascospores is the prominent type among halosphaeriaceous fungi, several other ascospore phenotypes such as, clavate to cylindrical (e.g. *Haligena*), clavate-broadly fusoid (e.g. *Morakotiella*, *Neptunella*, *Remispora*), elongate, fusiform (e.g. *Magnisphaera*, *Oceanitis*) and saccate ascospores types are also present (Spatafora et al. 1998; Sakayaroj et al. 2011). Ascospore shape and ascospore appendage ontogeny play an important role in the generic delineation in the Halosphaeriaceae (Jones et al. 1983a, 1983b, 1983c, 1986, 1995; Johnson et al. 1984, 1987; Pang 2001). Regarding the ascospore coloration, halosphaeriaceous ascospores are primarily hyaline except in *Carbosphaerella*, *Nereiospora*, *Phaeonectriella*, *Corollospora cinnamomea*, *C. fusca*, and *C. novofusca*. Different ascospore septation patterns viz. aseptate. (e.g. *Nautosphaeria*), 1-septate (e.g. *Morakotiella*, *Remispora*, *Neptunella*), or multi-septate (e.g. *Haligena*, *Magnisphaera*, *Oceanitis*), further increase the morphological diversity of the family. Halosphaeriaceae members basically comprise ascospore appendages or sheaths while species like *Paraaniptodera longispora*, *Okeanomyces cucullatus*, *Pseudolignincola siamensis* and *Saagaromyces glitra* lack ascospore appendages or sheaths (Sakayaroj et al. 2011). Ultrastructural studies of Halosphaeriaceae ascospores show that they basically comprise three wall layers; epi-(outermost), meso- (central) and endo- (inner) sporium. Ascospore appendages are formed by different mechanisms, such as, fragmentation of one or more layers (e.g. *Corollospora* spp., *Halosphaeriopsis mediosetigera*, *Remispora* spp.), outgrowths of one or more wall layers and emergence of the exosporium (e.g. *Ceriosporopsis*, *Halosphaeria*) and wall outgrowths (e.g. *Ondiniella torquata*). In some species, e.g. *Halosarpheia aquatica* and *Thalespora appendiculata*, appendages are only formed after release from the ascus and when mounted in seawater, but never in freshwater (Hyde 1992c; Jones and Hyde 1992; Jones et al. 2006). However, unfurling polar appendages have evolved many times within the family and are believed to be a special adaptation for successful dispersal, attachment and survival in aquatic habitats (Jones 1994). Figs 1, 2, 3 illustrate the great diversity in ascospore appendage morphology within the Halosphaeriaceae, which play a key role in identification of genera and species.

### Asexual morphs

Halosphaeriaceae contains many asexual morphs with several linked to their sexual morphs by culturing and phylogeny (Shearer 1986; Abdel-Wahab and Bahkali 2012). Many asexual morphs are more common than their sexual states: *Periconia pelagica* (=*Okeanomyces cucullatus*), *Monodictys pelagica* (=*Nereiospora cristata*). Others are only known as asexual morphs: *Cirrenalia basiminuta*, *Trichocladium melhae*. Morphological types include: 1. sigmoid conidia: *Halosigmoidea luteola* (=*Corollospora luteola*), *Halosigmoidea parvula* (=*Corollospora parvula*), 2. branched conidia: *Varicosporina ramulosa* (=*Corollospora ramulosa*), 3. conidia with bulbous basal cell with radiating arms: *Clavatospora bulbosa* (*Corollospora pulchella*), 4. conidia dark brown to fuscous, basal cell hyaline, muriform, pyriform, oblong ovoid, spherical, slightly constricted at the septa: *Monodictys pelagica* (=*Nereiospora cristata*), *Cirrenalia basiminuta*, 5. chlamydospores: *Corollospora mediterranea*.

### Aim of the paper

Morphological characteristics still play a crucial role in identifying and determining the relationships among fungal species even though using molecular data is the current trend in fungal classification. Furthermore, the nomenclatural type specimens constituted an integral part of fungal classification and nomenclature (Dayarathne et al. 2016). Some halosphaeriaceous genera/species lack molecular data and their assignment to the family require further study, such as *Aniptodera* spp. In this modern era, it is possible to prepare advanced morphological accounts for earlier observed poorly illustrated materials using greatly improved microscopes, which allow for more reliable phenotypic characterizations. Therefore, this monograph provides a complete morpho-molecular account for Halosphaeriaceae, with the introduction of several novel taxa and resolution of the taxonomic assignment of selected taxa.

Many genera in the Halosphaeriaceae introduced before sequence data became available were poorly delineated, e.g. *Aniptodera*. This has resulted in significant nomenclature changes and identification of cryptic genera: *Aniptodera*, *Corollospora*, and *Halosarpheia* leading to the introduction of many new genera. Other potential cryptic genera needing revision, with molecular data, are *Lignincola*, *Morakotiella*, *Nais*, *Natantispora*, *Saagaromyces*, *Aniptodera* and *Remispora*. Another cryptic genus may be *Bathyascus*, with all five species lacking molecular data. *Corollospora maritima*, a common and worldwide marine fungus, has been demonstrated to be a cryptic species (Velez et al. 2016; Correia et al. 2023) with circa 177 isolates sequenced. Of these, 103 gene sequences characterize *C. maritima*, the remaining representing putative new species (Correia et al. 2023). This highlights the necessity to identify gene sequences that represent the required species and thus avoid confusion in the construction of phylogenetic trees. Disputable identity may be due to the existence of a cryptic species, but these sequences may also be of poor quality, too short, or solely resulted from incorrect identification and/or registration. Furthermore, it is worth noting the need for more comprehensive molecular data, given that most analyses were only based on a single locus, most often the 28S rDNA.

Many species were described as asexual morphs, later matched to their sexual morphs by culture studies (*Trichocladium achrasporum*/*Halosphaeriopsis mediosetigera*, *Sigmoidea luteola*/*Corollospora luteola*) (Shearer 1986) and more recently by molecular data Humicola (Trichocladium) alopallonella/*Halosphaeriopsis alopallonella*, *Periconia prolifica*/*Morakotiella salina* (Sakayaroj et al. 2011; Calabon et al. 2023). Genera, such as *Chadefaudia*, *Fluviatispora*, *Luttrellia*, *Trichomaris*, are known only from their initial collection and lack cultures and molecular data, likewise for the species *Amphitrite annulata*, *Appendichordella amicta* and *Corallicola nana*. All require recollecting, isolation and sequencing.

## Materials and methods

### Specimens, morphological observation and redrawing

Nomenclatural type specimens or appropriate authentic specimens were loaned from accessible herbaria: BIOTEC Bangkok Herbarium (**BBH**), New York State Museum (**NY**), Mae Fah Luang University Herbarium (**MFLU**), Royal Botanic Gardens, Kew (**K**), Queensland Department of Agriculture and Fisheries (**BRIP**), Plant Protection Research Institute (**PREM**), National Museum in Prague (**PR**), Swedish Museum of Natural History (**S**), U.S. National Fungus Collections (**BPI**). A small portion of the herbarium material was detached and rehydrated with water or 5% KOH. Micro-morphological characters were examined from rehydrated ascomata with a Motic SMZ 168 stereomicroscope. Morphologies of ascomata, asci, ascospores and other special tissues (when necessary) were photographed with a Canon 550D digital camera ﬁtted to a Nikon ECLIPSE 80i compound microscope. Illustrations were assembled with Adobe Photoshop v. CS6 extended version and measurements were taken using Tarosoft v. 0.9.0.7. Many of the type specimens were in poor condition or have been lost or cannot be located, hence, few morphological characters were observed. Therefore, morphological information for these taxa were obtained depending on the protologue and hand drawings made for those taxa. Taxonomic descriptions were registered in Facesoffungi and Index Fungorum numbers (Jayasiri et al. 2015; Index Fungorum 2025).

### Phylogenetic analysis

New sequences were generated from pure cultures and reference sequences were retrieved from the GenBank (Suppl. material 1). Sequences from two isolates of each species were used to promote better defined clades, if available.

For the generation of new sequences, genomic DNA was used, followed by PCR amplification and sequencing methodology, as described by Pang et al. (2023b). The primers used for the amplification and sequencing of the genes were: 18S rDNA–NS1/NS4, (White et al. 1990), 28S rDNA–LROR/LR6 (Vilgalys and Hester 1990; Bunyard et al. 1994), TEF1–EF1-983F/EF1-2218R (Rehner and Buckley 2005), RPB2–fRPB2-5F/fRPB2-7cR (Liu et al. 1999), RPB1–RPB1-Af/RPB1-Cr (Stiller and Hall 1997), MCM7– Mcm7-709for/Mcm7-1348rev (Schmitt et al. 2009). All newly generated sequences of the Halosphaeriaceae and those in the GenBank were used to maximize the phylogenetic signals, although no protein gene sequences were available for comparison.

All genes were aligned separately in the program MUSCLE (Edgar 2004) in MEGA11 (Tamura et al. 2021) with *Microascus trigonosporus* and *Petriella setifera* as the outgroup taxa. The final dataset contained 184 taxa and a total of 8945 nucleotide positions. The genes were analyzed simultaneously. A maximum likelihood analysis including bootstrapping was performed in MEGA11 (Tamura et al. 2021) with the following settings: 1000 bootstrap, General Time Reversible model (GTR), gamma distributed (G), number of discrete gamma categories set at 5, heuristic search with Nearest-Neighbor-Interchange, initial tree from NJ/BioNJ method, branch swapping strong. A maximum parsimony (MP) analysis including bootstrapping was also run in MEGA11 with the following settings: 1000 bootstrap, Tree-Bisection-Reconnection (TBR), number of initial trees (random addition) = 5, MP search level = 1, maximum number of trees to retain = 100.

For Bayesian analysis, BEAUti v1.10.4 was used for prior settings and the dataset was analyzed in BEAST v1.10.4 (Suchard et al. 2018). The following analytical settings were used for the analysis: GTR, estimated base frequency, gamma distribution, number of gamma categories set at 5, a strict clock, Coalescent: Constant Size as the speciation model, running 10 million generations with parameters and trees sampled every 1000 generations. The first 10% of the trees were discarded as the burn-in based on the effective sample size (ESS) of the parameter statistics in Tracer v1.7.2 (Rambaut et al. 2018). A summary tree was generated in TreeAnnotator v1.10.4 (Suchard et al. 2018), viewed and edited in FigTree v1.4.4 (available at https://github.com/rambaut/figtree/releases).

### Phylogenetic analysis and time dating

For the phylogenetic analysis, barcode sequences (18S, 28S rDNA, TEF1, RPB1, RPB2 and MCM7) of representative taxa (Suppl. material 1) from Halosphaeriaceae, and the outgroup taxa *Microascus trigonosporus* AFTOL-ID 914 and *Petriella setifera* AFTOL-ID 956 were downloaded from the NCBI nucleotide database using the R package Analysis of Phylogenetics and Evolution (APE) (Paradis et al. 2004). Multiple sequence alignments were performed using MAFFT version 7.310 with options “--adjustdirection --auto” (Katoh et al. 2002) and the aligned datasets were further trimmed using trimAl version 1.4 (Capella-Gutiérrez et al. 2009) with the option “-gapthreshold 0.5”. The best-fit nucleotide substitution models were determined using ModelFinder version 2.1.1 under the Corrected Akaike Information Criterion (Kalyaanamoorthy et al. 2017). Aligned datasets were concatenated using an in-house Python script. Maximum-likelihood tree was reconstructed with IQ-TREE version 2.0.3 (Minh et al. 2020) based on the concatenated dataset.

For the time dating, we used RelTime implemented in MEGA 12 (Kumar et al. 2024) and the ML tree as input to inference the TimeTree. Since no fossil is available for divergence time estimation of Halosphaeriaceae, we initially selected two secondary calibrations from TimeTree (https://timetree.org/home), the divergence time of *Nohea* and *Nimbospora* was set to 50.6~170 MYA, and *Lignincola* and *Halosphaeria* set to 120.5~170 MYA. But these two calibrations conflicted. Then, we referred to the recent study by (Chen et al. 2023), used one calibration to fix the divergence time of *Nohea* and *Nimbospora* at 50.6 MYA. The GTR model was used to estimate divergence time.

## Results

### Phylogenetic analysis

**Figure 4.**
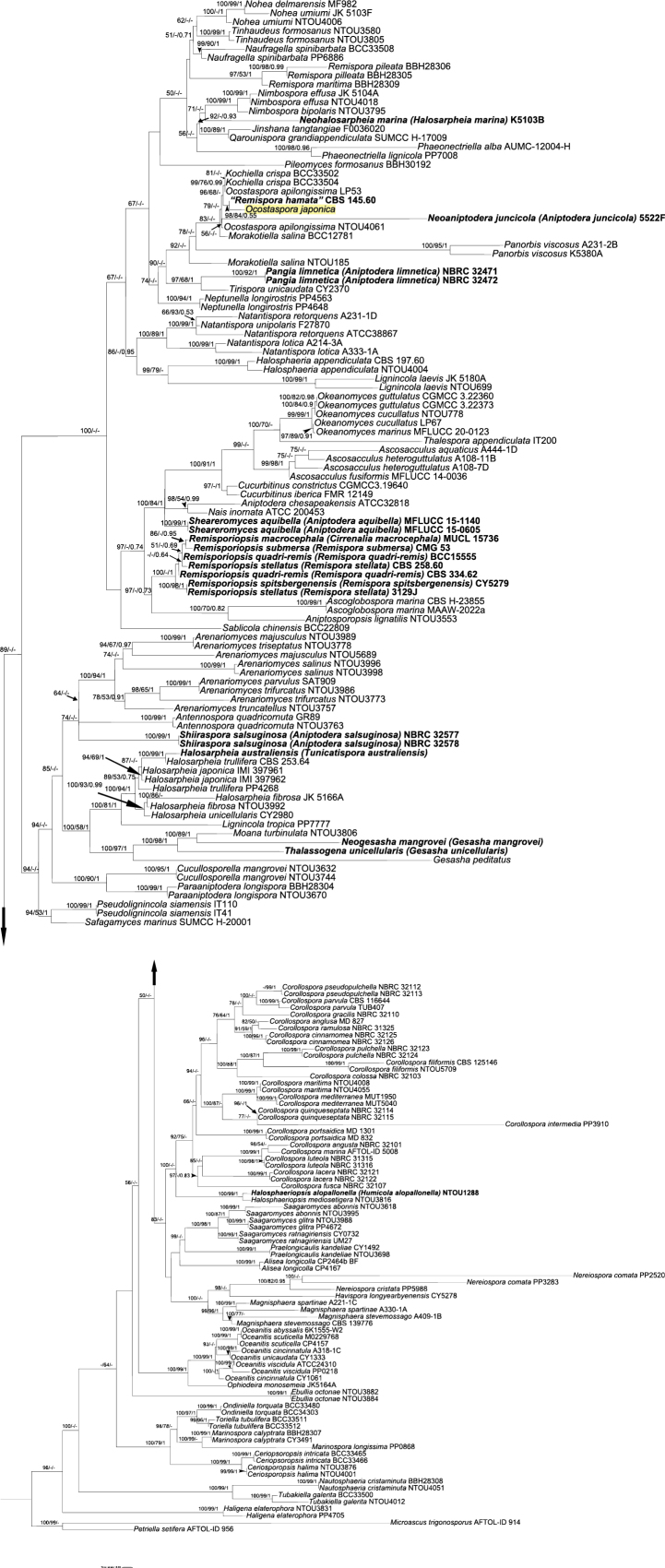
Maximum likelihood phylogenetic tree of 184 taxa of Halosphaeriaceae based on 6 ribosomal rRNA and protein coding genes, including 18S, 28S rDNA, RPB1, RPB2, TEF1-ɑ, MCM7. The numbers at the nodes represent maximum likelihood bootstrap value (>50), maximum parsimony bootstrap value (>50) and posterior probability (>0.5), respectively. The scale bar shows the mean expected rates of substitution per site. The new taxon is highlighted in yellow, and new combinations are indicated in bold.

The maximum likelihood tree shown in Fig. 4 was constructed based on six genes of the Halosphaeriaceae. The following groupings were observed: (1). *Nohea* was composed of *N. umiumi* NTOU4006 and *N. delmarensis* MF982 and with two *Tinhaudeus formosanus* strains (NTOU3580, NTOU3805), while two strains of *Naufragella spinibarbata* (BCC33508, PP6886) are placed in an adjacent clade. (2). *Remispora* was composed of *R. maritima* (type species) and *R. pilleata* with high support. (3). *Nimbospora* included *N. effusa* NTOU4018 and *N. bipolaris* NTOU3795 with *Neohalosarpheia marina* comb. nov. in a sister clade. (4). *Jinshana tangtangiae* (F0036020) formed a sister clade to *Qarounispora grandiappendiculata* (SUMCCH-17009) in a well-supported clade and adjacent to the *Nimbospora*/*Neohalosarpheia* clade. (5). *Phaeonectriella* comprised *Ph. lignicola* (PP7008) and *Ph. alba* (AUMC-12004-H) in a well-supported clade. (6). *Pileomyces formosanus* formed a basal unsupported branch to the previous five groups. (7). *Kochiella crispa*, grouped with *Ocostaspora apilongissima* LP53 and *O. japonica* sp. nov. in a moderatly supported clade. (8). *Neoaniptodera juncicola* comb. nov. grouped with another isolate of *Ocostaspora apilongissima* (NTOU4061). (9). *Panorbis viscosus*, two strains (A231-2B, K5380A), formed a group (long branch) with *Morakotiella salina*. (10). Two strains of *Pangia* (gen. nov.) *limnetica* comb. nov., (NBRC32471, NBRC32472) grouped with *Tirispora unicaudata* (CY2370) with high statistical support. (11). Two isolates of *Neptunella longirostris* (PP4563, PP4648) formed a well-supported branch basal to 7 to 10 groups. (12). *Natantispora retorquens* (A23-1D, ATCC39967) and *N. unipolaris* (F27870) formed a sister clade to *N. lotica* and the latter needs to be referred to a new genus when fresh material is available. (13). For *Halosphaeria appendiculata*, the two isolates formed a well-supported clade with *Lignincola laevis* as a sister group. (14). Three *Okeanomyces* species (*O. cucullatus*, *O. marinus* and *O. guttulatus*) formed a well-supported clade with a close relationship with *Thalespora appendiculata*. (15). *Ascosacculus* species (*As. aquaticus*, *As. fusiformis*, *As. heteroguttulatus*) grouped in highly supported clade. (16). *Remisporiopsis* is a new genus to accommodate five *Remispora* species that did not group with type species of *Remispora* (*R. maritima*) and *Cirrenalia macrocephala* also referred to this genus as *Re. macrocephalus*. (17). *Ascoglobospora marina* formed a distinct sister clade with *Aniptosporopsis lignatilis*. (18). Two isolates of *Antennospora quadricornuta* formed a well-supported clade. (19). The new genus *Shiiraspora* gen. nov. accommodates two isolates of *Aniptodera salsuginosa*, which were not monophyletic with *Aniptodera chesapeakensis*. (20). *Halosarpheia* clade comprised three species (*H. fibrosa*, *H. japonica* sp. nov., *H. unicellularis*) and two species synonymized in the genus (*Remispora trullifera* and *Tunicatispora australiensis* to *Halosarpheia*). (21) *Gesasha peditatus* formed sister clades with *Moana turbinulata* (NTOU3806), *Neogesasha* (new genus) *mangrovei* comb. nov. and *Thalassogena unicellularis* comb. nov. with high statistical support. (22). *Cucullosporella mangrovei* and *Paraaniptodera longispora* formed distinct well-supported clades. (23). Two strains of *Pseudolignincola siamensis* formed a sister clade to the asexual morph genus *Safagamyces marinus*. (24). *Corollospora* species formed a highly supported clade with *Halosphaeriopsis* (*H. mediosetigera* and *H. alopallonella*). (25). *Saagaromyces* clade comprised the species *S. ratnagiriensis*, *S. abonnis*, *S. glitra* and *S. mangrovei*, however, *S. abonnis* is a species complex and requires further study. (26–27). *Praelongicaulis* and *Alisea* constituted two well-supported clades. (28). *Nereiospora* contained two species, *N. cristata* and *N. comata*. (29). *Magnisphaera* (*M. spartinae* and *M. stevemossago*) formed a clade adjacent to the *Nereiospora* clade. (30). *Oceanitis* is a specious genus with *O. scuticella*, *O. abyssalis*, *O. cincinnatula*, *O. unicaudata* and *O. viscidula*. (31–33). *Ondiniella*, *Toriella* and *Marinospora* formed well-supported clades with the genus *Ceriosporopsis*. (34) in an adjacent clade. (35–37). *Nautosphaeria*, *Tubakiella* and *Haligena* were three well-supported basal clades.

Jones (2011) opined that many cosmopolitan marine fungi, *Ceriosporopsis halima*, *Corollospora maritima* and *Haiyanga salina*, were species complexes. Pang (2012) in an early study of the family undertook p-distance estimations highlighting that *Remispora* was divided into two clades: *Remisporasensu stricto* with *R. maritima* as the type, while the second group comprised *R. stellata*, *R. spitsbergenensis* and *R. quadri-remis*. This has also been reported by Sakayaroj et al. (2011) and Gonçalves et al. (2021).

### Time line analysis

**Figure 5.**
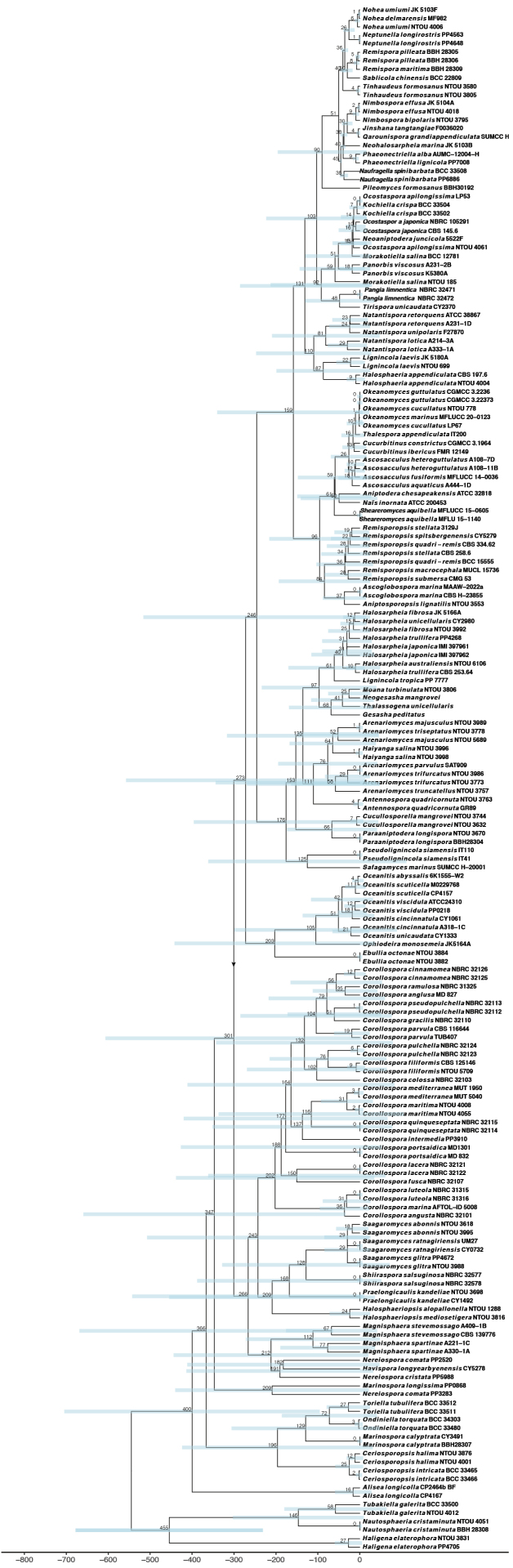
Divergent time estimation of Halosphaeriaceae based on combined 18S, 28S rDNA, RPB1, RPB2, TEF1-ɑ, MCM7 sequences. Numbers at the nodes represent estimated divergence times in million years ago (MYA), with 95% confidence intervals shown as bars. Outgroup taxa were removed from TimeTree.

The evolutionary tree (Fig. 5) indicates that Halosphaeriaceae originated in the Paleozoic at ~545.1 MYA (95% CI: 351.1–846.3 MYA). Several lineages have much younger crowns: *Haligena elaterophora* (~27.3 MYA; 95% CI: 9.9–75.2 MYA) and *Nautosphaeria cristaminuta* (0–9.4 MYA) show shallow in-genus divergences, whereas *Tubakiella galerita* has a deeper crown (~58.0 MYA; 95% CI: 13.1–257.0 MYA). Within the core clade that includes *Halosphaeria* and *Lignincola*, the split between these genera dates to ~86.8 MYA (95% CI: 29.7–253.5 MYA), but the crown of *Halosphaeria* itself is much younger (~9.3 MYA; 95% CI: 1.9–45.6 MYA). Divergence times well support several generic boundaries. *Remisporiopsis* has a crown of ~35.8 Ma (95% CI: 9.2–144.3 MYA) and is separated from *Remispora* (crown ~7.8 MYA; 95% CI: 3.5–17.1 MYA) by a much older inter-generic split (~158.7 MYA; 95% CI: 59.6–422.6 MYA), justifying their recognition as distinct genera. *Shiiraspora* shows a very recent crown (0–12.2 MYA), yet its split from *Aniptodera* is ancient (~300.8 MYA; 95% CI: 123.1–734.8 MYA), supporting its segregation. *Neogesasha* and *Gesasha* are separated by ~68.4 MYA (95% CI: 21.1–221.8 MYA); *Neoaniptodera* and *Ocostaspora* diverged ~15.0 MYA (95% CI: 3.5–65.1 MYA); and *Neohalosarpheia marina* is well separated from *Nimbospora* (~37.8 MYA; 95% CI: 29.9–46.4 MYA).

## Taxonomy

### Halosphaeriaceae

Taxon classificationFungiMicroascalesHalosphaeriaceae

E. Müll. & Arx ex Kohlm., Canad. J. Bot. 50 (9): 1951 (1972)

0370DBD2-15F4-549E-BB00-BD875D7DECFC

80832

#### Description.

Saprobic on algae, immersed or submersed on phanerogams, wood, bark, leaves, and other cellulosic plant remains, grains of sand, or in calcareous shell fragments, rarely parasitic or symbiotic, on polyurethane panels, found in marine (oceans and estuaries) and freshwater habitats. **Sexual morph. *Ascomata*** subglobose, ellipsoidal or cylindrical or pyriform, hyaline or dark; sometimes subiculate, rarely stromatic; superficial or immersed. ***Ostioles*** papillate to long cylindrical; ostiolar canal with periphyses or pseudoparenchyma; rarely without ostioles. ***Peridium*** soft or subcarbonaceous, composed of flattened, thick or thin-walled cells. Centre of immature ascomata consisting of polygonal, thin-walled, pseudoparenchymatic cells, sometimes with pits, at maturation separating to form catenophyses or compressed by the asci and deliquescing. ***Paraphyses*** absent. Hymenial layer at base of venter, flat or convex. Mature ascospores filling the venter of the ascoma, released singly through ostiole or rarely within the ascus, which swells after dispersal. ***Asci*** fusiform, clavate or rarely subglobose, with or without apical structures, thin-walled, 1-layered, persistent or swelling and deliquescing at or before ascospore maturity. ***Ascospores*** overlapping 2–3 seriate, hyaline or light brown, broad-ellipsoidal, 1-multi septate, mostly with characteristic ornamentations, with appendages or gelatinous sheaths, or both. **Asexual morph**. Hyphomycetes, conidiogensis holoblastic, or obsolete, conidia variable, coiled, terminal, septate or muriform, branched or staurospores, conidia solitary pyriform thick-walled, hyaline or brown, forming chlamydospores or sclerocarps.

#### Type genus.

*Halosphaeria* Linder, Farlowia 1(3): 412 (1944).

#### Notes.

Although around 62% of halosphaeriaceous species have been sequenced, their phylogenetic clading is not supported by morphological features (Sakayaroj et al. 2011). This indicates that certain characters have evolved and been lost several times (Jones et al. 2017). Based on our divergence-time analyses (Fig. 5), Halosphaeriaceae originated in the Paleozoic, with a crown age of ~545 Ma (95% CI: 351–846 MYA), highlighting its ancient evolutionary history.

### *Halosphaeria* Linder

The monotypic genus *Halosphaeria* was introduced by Barghoorn and Linder (1944) typified by *H. appendiculata*. Cribb and Cribb (1956), Kohlmeyer (1960) and Schmidt (1974a) transferred other species to this genus. Kohlmeyer (1972) opined that the borderline of the monotypic genera (*Ceriosporopsis*, *Halosphaeria*, *Lentescospora* and *Remispora*) introduced by Barghoorn and Linder (1944) had become indistinct with the description of other species. Consequently, *Antennospora*, *Arenariomyces*, *Halosphaeriopsis*, and *Remispora* species were transferred to *Halosphaeria*, a move that was not accepted by other mycologists. Ultrastructure studies (SEM and TEM) of ascospore appendage ontogeny by Jones et al. (1983a, 1983b, 1983c, 1984) and Johnson et al. (1984, 1987) reinstated many of the changes introduced by Kohlmeyer (1972).

#### Halosphaeria

Taxon classificationFungiMicroascalesHalosphaeriaceae

Linder, Farlowia 1(3): 412 (1944)

38CD97F8-4487-5426-9D75-368A4E36C793

Index Fungorum: IF2209

##### Description.

Saprobic on wood in marine environments. **Sexual morph. *Ascomata*** solitary, sometimes gregarious, globose, subglobose, obpyriform, ellipsoidal, immersed to superficial, ostiolate, papillate, coriaceous, pale brown to black, necks variable in length, periphysate, centrum breaking up into deliquescing catenophyses. ***Peridium*** comprises 4–6 layers of thick-walled, brown to dark brown cells of *textura angularis*, hyaline towards the inner. ***Asci*** 8-spored, unitunicate, thin-walled, clavate, pedicellate, lacking an apical apparatus, deliquescing early and developing at the base of the ascocarp from a pad of ascogenous hyphae. ***Ascospores*** overlapping 2–3 seriate, hyaline, ellipsoidal, 1-septate, slightly constricted at the septum, with 3–4, equatorial appendages that are obclavate and form deep spoon-shaped structures at the regions of attachment and two morphologically similar polar appendages. ***Appendages*** initially closely wrapped around the spore, expanding in seawater and possess a folded substructure. **Asexual morph**. Undetermined.

##### Type species.

*Halosphaeria appendiculata* Linder, Farlowia 1(3): 412 (1944).

##### Notes.

Other species assigned to the genus by Kohlmeyer (1972) have been transferred to other genera such as *Antennospora*, *Halosphaeriopsis*, *Okeanomyces*, and *Remispora* as a result of ultrastructural and molecular studies (Jones et al. 1984, 2009, 2015). The unique feature of the genus *Halosphaeria* is the inverted or spoon-shaped nature of the polar and equatorial appendages (Jones et al. 1984). Transmission electron microscopy showed that the ascospore wall comprises two layers, a thin electron-dense episporium and a thicker inner, less electron-dense mesosporium without an exosporium (Lutley and Wilson 1972; Jones et al. 1984). Outermost layer of the spore wall and the first to form is the epispore (Kirk 1966). The young spore becomes more angular, forming projections at both poles and at several points of the equator. Appendages are initially closely wrapped around the spore, and unfold in water. At maturity, appendages composed of electron-dense strands with a less electron-dense amorphous component (Hyde et al. 1994). Kirk (1966) suggested that the mesospore may consist largely of glucans and when the spore develops, glycogen builds up in the cytoplasm. Initial phylogenetic analyses with combined 18S and 28S rDNA data of Spatafora et al. (1998) showed *H. appendiculata* and *Corollospora maritima* were placed as a sister group to *Microascus trigonosporus*. Studies by Campbell et al. (2003) described the placement of *H. appendiculata* sister to *Lignincola laevis* within Halosphaeriaceae, this was supported by several later studies by Pang (2001), Jones et al. (2009, 2015), Sakayaroj et al. (2011), and Jones et al. (2017). In our phylogenetic analyses, the two *H. appendiculata* strains formed a well-supported lineage sister to *Lignincola* (Fig. 4).

##### Molecular evaluation.

*Halosphaeria* is a well-established monotypic genus in the Halosphaeriaceae, which diverged from its sister genus *Lignincola* around ~86.8 MYA (95% CI: 29.7–253.5 MYA), while the crown of *Halosphaeria* itself is comparatively young at ~9.3 MYA (95% CI: 1.9–45.6 MYA) (Fig. 5).

#### Halosphaeria appendiculata

Taxon classificationFungiMicroascalesHalosphaeriaceae

Linder, Farlowia 1(3): 412 (1944)

FE850E02-EDA4-5850-ADF3-88E71C566B6D

Index Fungorum: IF286839

Figs 2a, 6

##### Description.

Saprobic on drift wood in marine water. **Sexual morph. *Ascomata*** 140–330 × 140–500 μm, solitary, sometimes gregarious, globose, subglobose, obpyriform, ellipsoidal, immersed to superficial, ostiolate, papillate, coriaceous, pale brown to black, necks variable in length, periphysate, centrum breaking up into deliquescing catenophyses. ***Peridium*** 10–28 μm wide, comprises 4–6 layers of thick-walled, brown to dark brown cells of *textura angularis* hyaline towards the inner. ***Asci*** 50–108 × 10–24 μm, 4 or 8-spored, unitunicate, thin-walled, clavate, pedicellate, lacking an apical apparatus, deliquescing early and developing at the base of the ascocarp from a pad of ascogenous hyphae. ***Ascospores*** 16–29 × 6–12 μm (excluding appendages), overlapping 2–3 seriate, hyaline, ellipsoidal, 1-septate, slightly constricted at the septum, with 3–4, equatorial appendages 7–25 × 1–3 μm that are obclavate and form deep spoon-shaped structures at the regions of attachment and two morphologically similar polar appendages. **Asexual morph**. Undetermined.

**Figure 6.**
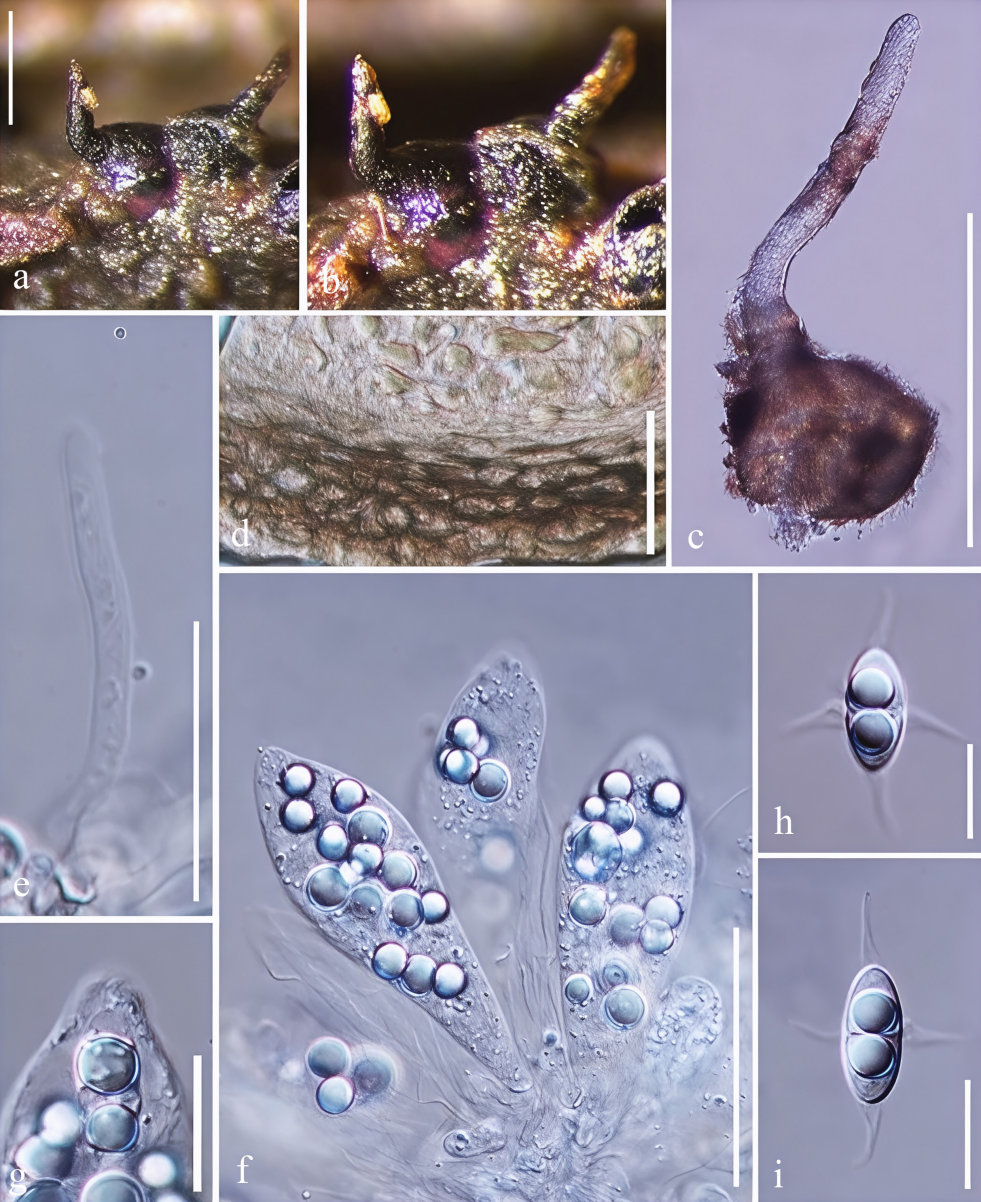
*Halosphaeria appendiculata*. **a, b**. Ascomata superficial to semi-immersed in driftwood; **c**. Long necked ascoma; **d**. Peridium; **e**. Ascogenous hyphae; **f**. Asci along with catenophyses; **g**. Apex of an ascus; **h, i**. Ascospores with polar and equatorial appendages. Scale bars: 200 μm (**c**); 20 μm (**d, e**); 50 μm (**f**); 10 μm (**g–i**).

##### Material examined.

UK, • Hampshire, Portsmouth, Langstone harbour, on unidentified drift wood, 26 January 2018, E.B.G. Jones, GJ457.

##### Notes.

*Halosphaeria appendiculata* occurs most frequently in marine substrates (Abdel-Wahab 2011a), and is a saprobe on decaying, submerged wood in the intertidal, submerged and driftwood, on bark, test panels and other cellulosic materials (Kohlmeyer and Kohlmeyer 1979; Jones et al. 2009, 2015). Although this species is cosmopolitan in its distribution, it tends to occur more frequently in temperate waters than in tropical locations (Cribb and Cribb 1956; Kohlmeyer and Kohlmeyer 1979; Jones 1985; Nakagiri 1989).

##### Distribution.

Argentina, Australia, Canada, Chile, Denmark, France, Germany, Iceland, Portugal, Spain, Sweden, Japan, UK (England, Scotland, Wales), USA (Massachusetts, Rhode Island, North Carolina), USSR.

### *Alisea* J. Dupont & E.B.G. Jones

Dupont et al. (2009) introduced the new genus *Alisea* with *A. longicolla* as the type species, from material collected on small twigs and sugar cane debris trawled from the bottom of the Pacific Ocean off Vanuatu Islands. Named *Alisea* in reference to the research vessel ‘‘Alis’’ used for the collection of material (Dupont et al. 2009). Dupont and Schwabe (2016) made further collections on recently submerged wood in the Northwest Pacific Ocean. Nagano et al. (2019) reported this species from a wood log (*Quercus phillyreoides*) artificially immersed at 495 m depths in the deep sea, off the Nansei Islands, Japan. They opined it “may be an endemic deep-sea fungus, as it grows slowly but is active and reproductive in deep-sea environments”. This genus is currently monotypic.

Occurrence of this fungus on wood panels in the deep sea could confirm their ability to colonize and take an active role in recycling lignocellulose in the deep ocean as saprobes (Nagano et al. 2019). There is only one report on the detection of *A. longicolla* from terrestrial material (organic compost) by 454 pyrosequencing in Oman (Al-Mazroui and Al-Sadi 2016), however, its identity is doubtful. Detection of *A. longicolla* in terrestrial environments is debatable, but its life cycle remains veiled as little is known about the ecology and physiology of *A. longicolla*, due to its rareness of discovery and its difficulty of culture (Nagano et al. 2019).

#### Alisea

Taxon classificationFungiMicroascalesHalosphaeriaceae

J. Dupont & E.B.G. Jones, Mycol. Res. 113(12): 1358 (2009)

27B83D5A-8CDF-5AEC-8CBC-801E9688B4D6

Index Fungorum: IF513289

##### Description.

Saprobic on wood. **Sexual morph. *Ascomata*** globose, coriaceous, black, superficial, solitary or gregarious, with long periphysate necks. ***Peridium*** thick, outer layer composed of polygonal cells; inner layer with flattened cells. ***Asci*** eight spored, clavate, long pedunculate, thin-walled except for the thick-walled apex, unitunicate, persistent to deliquescing. ***Ascospores*** fusiform, slightly curved, one-septate, thick-walled, hyaline, appendages not observed. **Asexual morph**. Undetermined.

##### Type species.

*Alisea longicolla* J. Dupont & E.B.G. Jones, Mycol. Res. 113(12): 1358 (2009).

##### Notes.

Phylogenetic placement of *Alisea* within Halosphaeriaceae has been confirmed by phylogenetic analyses of combined 18S and 28S rDNA data (Dupont et al. 2009). *Alisea* grouped as a distinct lineage apart from other genera within Halosphaeriaceae in different studies (Dupont et al. 2009; Sakayaroj et al. 2011; Jones et al. 2015) but with weak support. In our study the two strains of *A. longicolla* form a highly supported clade with genera in the *Saagaromyces* and *Praelongicaulis* clades (Fig. 4).

This genus is saprobic on wood recovered from the deep sea (Dupont et al. 2009; Nagano et al. 2019), and could be an interesting taxon to investigate wood-degrading enzymes, as they degraded sunken wood in a very different environment from terrestrial habitats (Nagano et al. 2019).

##### Molecular evaluation.

Two strains of *A. longicolla* formed a well-supported clade in the Halosphaeriaceae and is ecologically and morphologically distinct from other genera in the family (Fig. 4). Divergence-time estimates indicate that *Alisea* separated from its closest relatives in the early Cenozoic, around ~15.6 MYA (95% CI: 0–54 MYA) (Fig. 5).

#### Alisea longicolla

Taxon classificationFungiMicroascalesHalosphaeriaceae

J. Dupont & E.B.G. Jones, Mycol. Res. 113(12): 1358 (2009)

303C9C20-277A-59FC-8060-C867AEA84424

Index Fungorum: IF513290

Figs 7, 8

##### Description.

Saprobic on wood. **Sexual morph. *Ascomata*** 600–850 μm diam., globose to obpyriform, coriaceous, black, superficial, solitary or gregarious, carbonaceous. ***Necks*** central, 240–250 mm wide, 200–600 μm long, cylindrical to conical, periphysate. ***Peridium*** 100 μm thick, two layered; outer layer composed of polygonal cells, dark brown; inner layer with flattened, lighter cells. ***Asci*** 40–50 × 16–20 μm, 8-spored, clavate, long pedunculate, thin-walled except for the thick-walled apex, unitunicate, persistent. ***Ascospores*** 30–40 × 3–4 μm, fusiform, slightly curved, one-septate, thick-walled, hyaline. Asci and ascospore characters as described by Dupont et al. (2009) and Nagano et al. (2019). **Asexual morph**. Undetermined.

**Figure 7.**
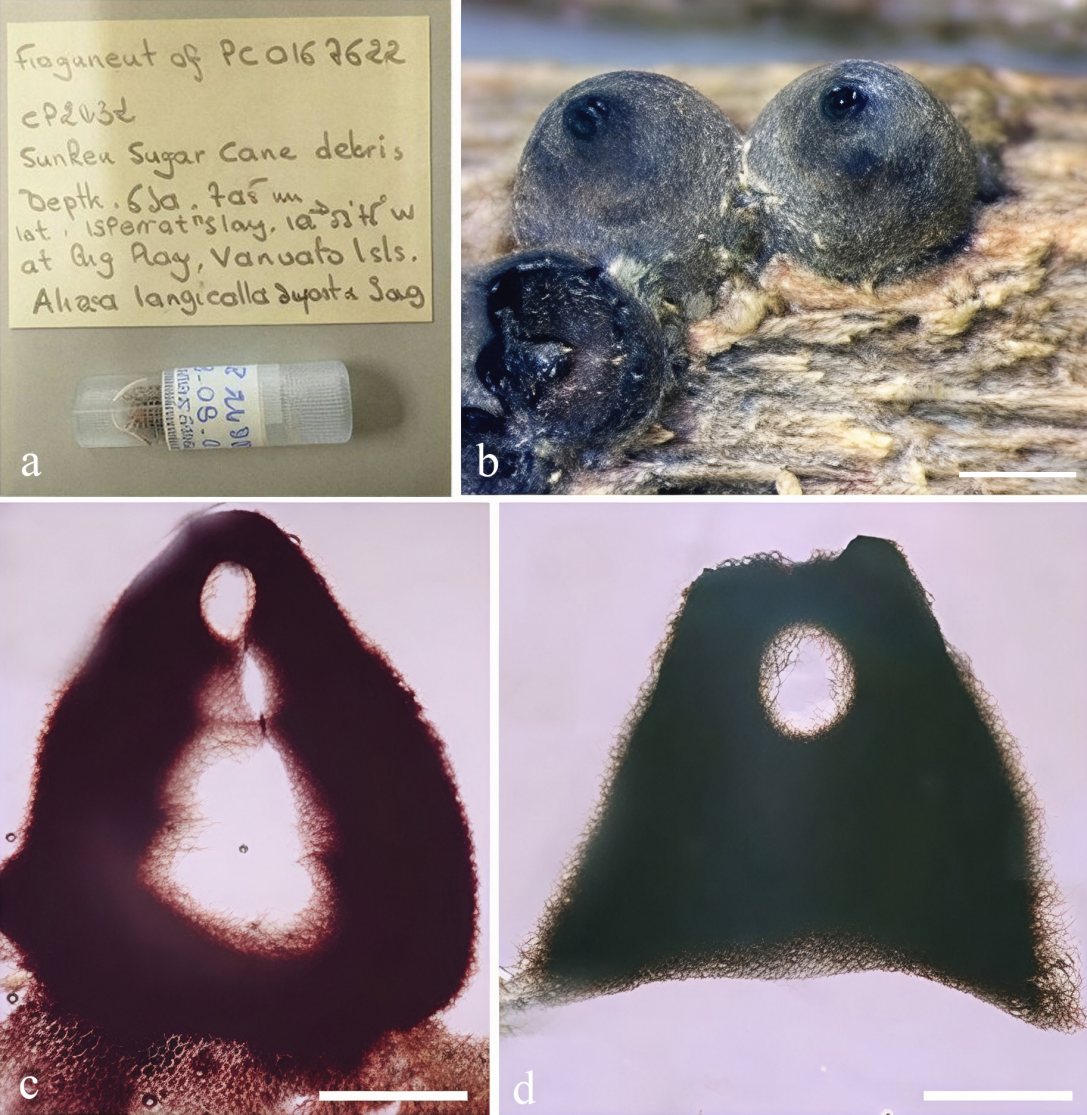
*Alisea longicolla* (PC 0167622, holotype)**. a**. Herbarium material; **b**. Ascomata on host; **c**. Section through ascomata; **d**. Section through neck region. Scale bars: 500 µm (**b**); 100 µm (**c**); 50 µm (**d**).

**Figure 8.**
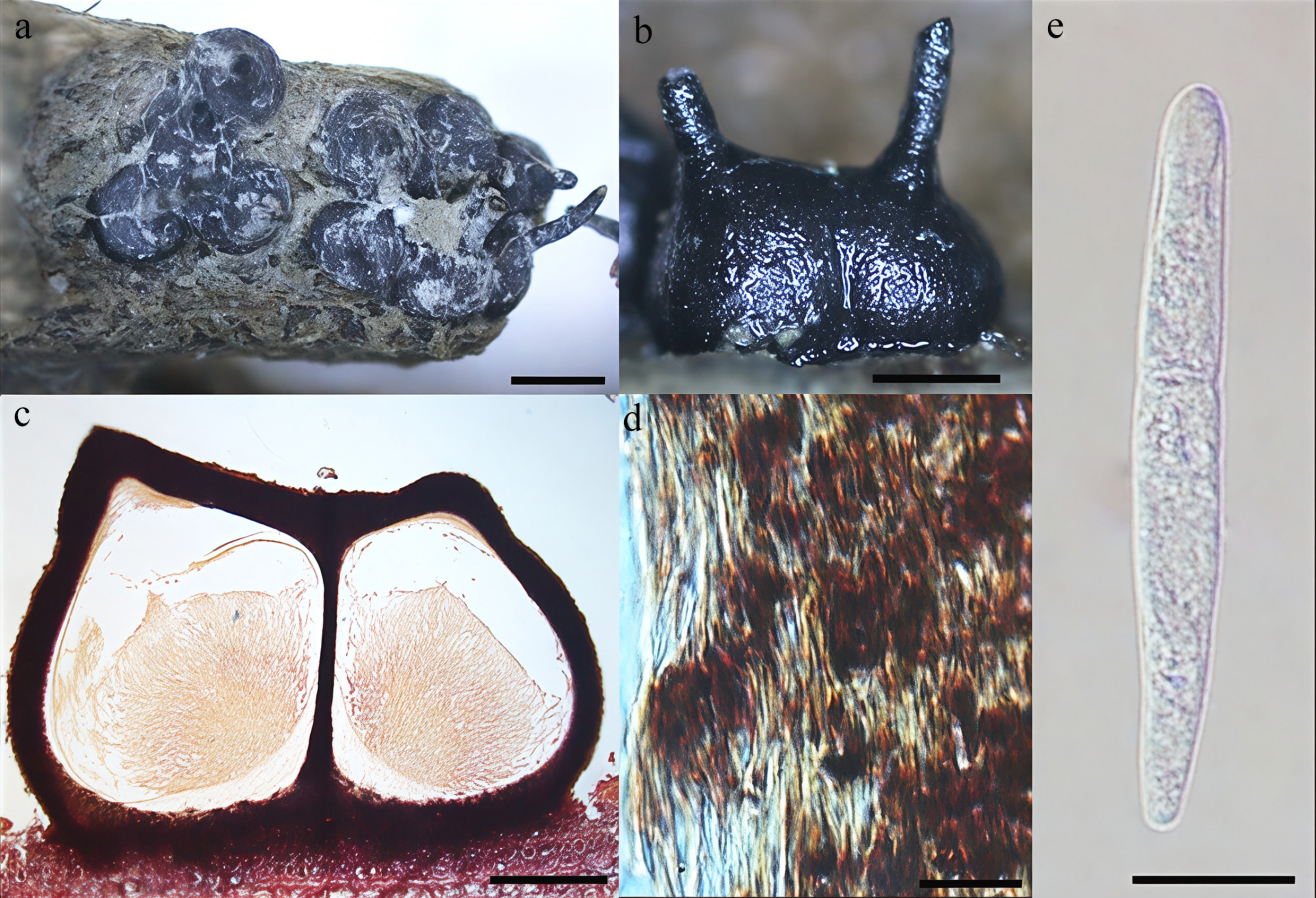
*Alisea longicolla*. **a, b**. Ascomata on wood with long necks; **c**. Section of ascomata; **d**. Peridial wall; **e**. Septate ascospore. Scale bars: 500 mm (**a, b**); 200 mm (**c**); 10 mm (**d, e**).

##### Material examined.

Vanuatu Islands, • Pacific Ocean, from wood twigs submerged in sea, 2005, J. Dupont, PC 0115840 holotype.

##### Notes.

*Alisea longicolla* was originally described as a new deep sea inhabiting ascomycete associated with sunken wood collected at 630–791 m water depths (Dupont et al. 2009) while, further evidence was provided by Nagano et al. (2019) to support that *A. longicolla* as an endemic deep-sea fungus. *Alisea longicolla* is an interesting taxon within Halosphaeriaceae due to its specific habitat, the deep sea. However, no distinct morphological adaptation to deep sea living style has been observed in this species, but the peridium wall is considered thick among members of the family. The holotype of *Alisea longicolla* was examined but lacked asci and ascospores.

Nagano et al. (2019) opined that genome analysis of *A. longicolla* may provide new insights into the ecology of the organism and would be interesting to reveal how the Halosphaeriaceae evolved from terrestrial species and how *A. longicolla* evolved to adapt to deep-sea environments.

##### Distribution.

Vanuatu Islands, Japan, Northwest Pacific Ocean.

### *Amphitrite* S. Tibell

Tibell (2016) introduced the genus *Amphitrite* to accommodate *Amphitrite annulata* which was earlier considered as *Halosphaeria appendiculata*. Tibell (2016) commented that as there were no young or semi-mature ascomata to observe, the morphological description is incomplete.

#### Amphitrite

Taxon classificationFungiMicroascalesHalosphaeriaceae

S. Tibell, Svensk Mykologisk Tidskrift 37(2): 45 (2016)

499C8E54-2C65-5B1E-AF17-57D69A8CC295

Index Fungorum: IF817480

##### Description.

Saprobic on driftwood (oak). **Sexual morph. *Ascomata*** globose to subpyriform, superficial, black, solitary, ostiolate. ***Ostiole*** central. ***Peridium*** consisting of an inner 5–7 layers of strongly flattened, hyaline, thick-walled concentrically arranged cells and the outermost, 1–2 layers of black, elongated hyphae that connect with a dark surrounding tissue reaching into the substrate, surrounding tissue consisting of branched and intertwined hyphae. ***Paraphyses*** hyaline, aseptate, branched. ***Asci*** 8-spored, thin-walled, ellipsoidal, pedicellate, 2–3-seriate. ***Ascospores*** ellipsoid, 1-septate, hyaline, comprising a ring-like, light refractive collar, which encloses the spore at the septum, with two apical appendages at both ends. **Asexual morph**. Undetermined.

##### Type species.

*Amphitrite annulata* S. Tibell, Svensk Mykologisk Tidskrift 37(2): 45 (2016).

##### Notes.

**Figure 9.**
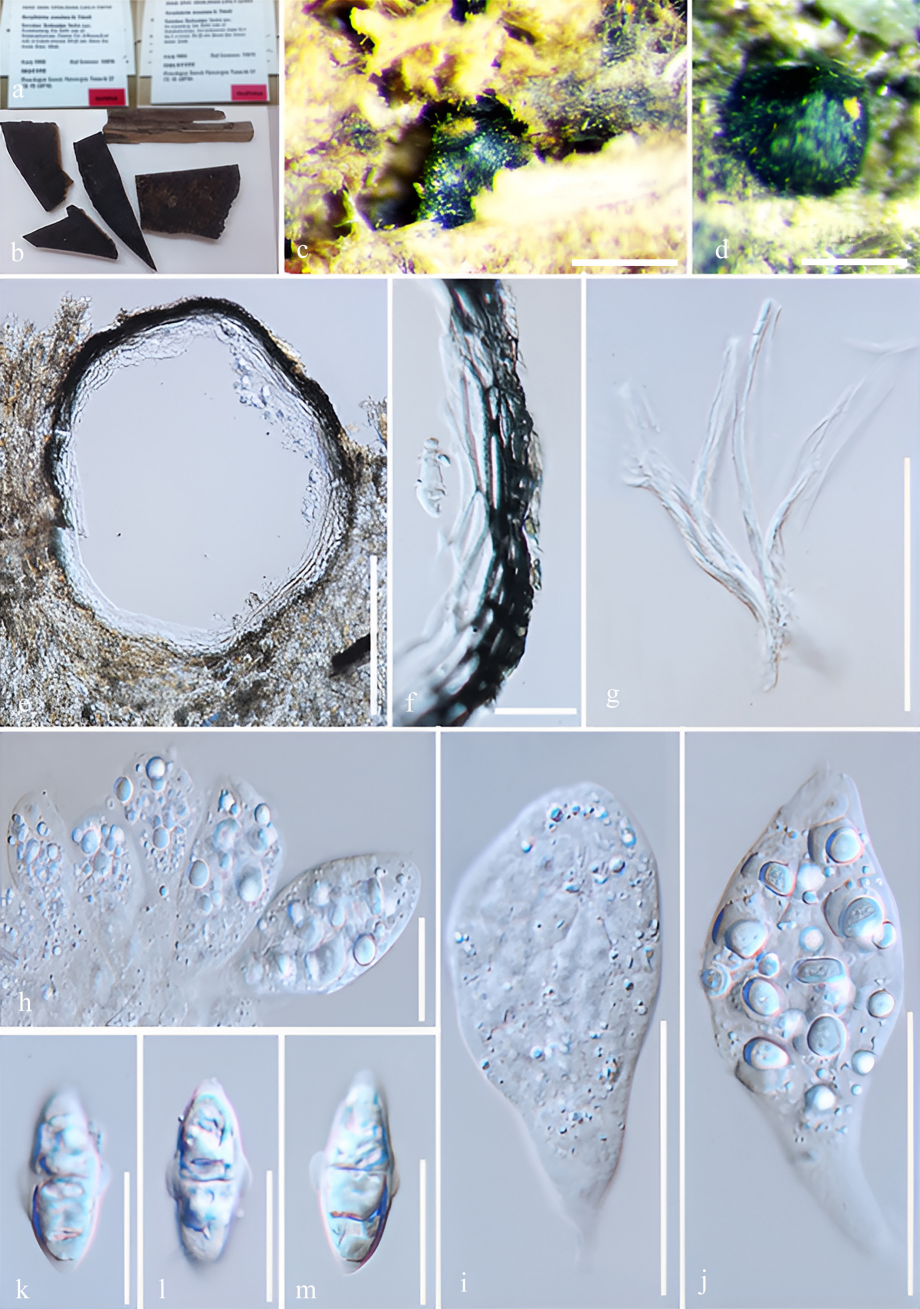
*Amphitrite annulata* (UPS 10870, holotype). **a, b**. Herbarium material; **c, d**. Appearance of ascomata on host surface; **e**. Section through ascoma; **f**. Peridium; **g**. Paraphyses; **h–j**. Asci; **k–m**. Ascospores. Scale bars: 200 μm (**c, d**); 100 μm (**e**); 20 μm (**f–h, k–m**); 50 μm (**i, j**).

*Amphitrite* shares similar morphology to the genus *Ondiniella* (Jones et al. 1984; Tibell 2016). To further confirm the uniqueness of this genus from the rest of genera in the Halosphaeriaceae, morphological observations and molecular phylogenetic data from fresh collections are required. According to our morphological observations of the holotype of *A. annulata*, the genus superficially looks similar to *Ondiniella*. We observed a ring-like, light refractive collar (Fig. 9k–m), which encloses the spore at the septum as well as two apical appendages at both poles. We do not propose synonymizing the genus under *Ondiniella* until fresh material becomes available for sequencing.

##### Molecular evaluation.

Fresh collections, isolation and sequencing of this genus are urgently required to resolve its taxonomic position in the Halosphaeriaceae.

#### Amphitrite annulata

Taxon classificationFungiMicroascalesHalosphaeriaceae

S. Tibell, Svensk Mykologisk Tidskrift 37(2): 45 (2016)

13C25FEB-CE9F-5D34-BBF1-002EA0CD816F

Index Fungorum: IF817492

Fig. 9

##### Description.

Saprobic on wood in marine habitat. **Sexual morph. *Ascomata*** 140–195 μm in diam., globose to subpyriform, superficial, black, solitary, ostiolate. ***Ostiole*** central. ***Peridium*** 15–20 μm thick, consisting of an inner 5–7 layers of strongly flattened, hyaline, thick-walled concentrically arranged cells and the outermost, 1–2 layers of black, elongated hyphae that connect with a dark surrounding tissue reaching into the substrate, surrounding tissue consisting of branched and intertwined hyphae. ***Paraphyses*** hyaline, aseptate, branched. ***Asci*** 52–68 × 16–19 μm, 8-spored, thin-walled, ellipsoidal, pedicellate, 2–3-seriate. ***Ascospores*** 18–21 × 5.5–8 μm, ellipsoid, 1-septate, hyaline, comprising a ring-like light refractive collar, which encloses the spore at the septum, with two apical appendages at both ends. **Asexual morph**. Undetermined.

##### Material examined.

Sweden, • Bohuslän, Skaftö, Kristineberg, (S)W side of Blåbärsholmen, on driftwood (oak) in a dark crevice 20–30 cm above mean water level, 1955 July 04, Santesson, (UPS 10870, holotype) on wooden panels submerged for circa 1 year.

##### Notes.

This species is saprobic on driftwood (oak) trapped in a crevice, 20–30 cm above mean water level (Tibell 2016).

### *Aniptodera* Shearer & M.A. Mill.

The genus *Aniptodera* was introduced by Shearer and Miller (1977) with *Aniptodera chesapeakensis* as the type species. Later, a number of species have been added to this genus, including both marine and freshwater taxa (Tsui et al. 1997; Jones et al. 2019; https://www.marinefungi.org; https://www.freshwaterfungi.org). The genus is characterized by thick- and thin-walled ascomata and species with both appendaged and non-appendaged ascospores, which has led to confusion in delineation of the species (Volkmann-Kohlmeyer and Kohlmeyer 1994).

#### Aniptodera

Taxon classificationFungiMicroascalesHalosphaeriaceae

Shearer & M.A. Mill., Mycologia 69(5): 893 (1977)

A4DB4C79-5943-592A-917D-8F87E0929774

Index Fungorum: IF198

##### Description.

Saprobic on submerged woody substrates in marine and freshwater habitats. **Sexual morph. *Ascomata*** perithecial, superficial or partially immersed, globose to subglobose, hyaline or greyish brown to dark brown, membranous, ostiolate, neck elongated, cylindrical, apical or subapical portion of the neck becoming dark, forming a ring around the neck, periphysate. ***Peridium*** two-layered, outer layer of cells of *textura angularis* and inner layer of elongate cells. ***Paraphyses*** absent. ***Catenophyses*** wide, sparse, hyaline, consisting of elongated cells, septate, slightly constricted at the septa. ***Asci*** 8-spored, in hymenium at base of perithecium, clavate, or becoming balloon-shaped or swollen, unitunicate, wall thickened, plasmalemma retracted below the apex, apically truncate, with a J- apical thickening or with an apical pore, with a short pedicel, asci deliquescing at maturity. ***Ascospores*** hyaline, 2–3-seriate, ellipsoidal, euseptate or 1–2 septate, not constricted or slightly constricted at the septum, thick-walled, guttulate, with or without appendages or with indistinct appendages. Where they occur, appendages are bipolar, thin, hamate, filamentous, cylindrical to conical, extending over the mid-septum, unfurling into fine threads in water, long or short. **Asexual morph**. Undetermined.

##### Type species.

*Aniptodera chesapeakensis* Shearer & M.A. Mill., Mycologia 69(5): 894 (1977).

##### Notes.

Sixteen marine, brackish water and freshwater species are included in *Aniptodera*, and they are commonly found in the tropics and temperate waters as saprobes (Jones et al. 2017). *Aniptodera* species have been reported from decomposing mangrove wood such as *Bruguiera gymnorrhiza*(Nakagiri 1994), and other substrates in the sea, balsa wood, *Juncus roemerianus*, *Nypa fruticans*, *Populus deltoides*, *Platanus occidentalis*, and *Raphia australis* submerged in freshwater habitats (Shearer 1972; Shearer and Miller 1977; Hyde et al. 1999a; Li et al. 2016; Jones et al. 2017). *Aniptodera chesapeakensis* has been shown to cause soft rot decay of wood (Jones 1972, 1975; Mouzouras 1986).

The genus was referred to the Halosphaeriaceae by Kohlmeyer and Kohlmeyer (1979) and this was confirmed by sequence data (Sakayaroj et al. 2011). *Aniptodera* (*A. chesapeakensis*) is most similar to *Lignincola* (*L. laevis*) and *Nais* (*N. inornata*) which produce two-celled ascospores lacking appendages (Shearer 1972; Jones et al. 2017). This was supported by our phylogenetic studies where *A. chesapeakensis* grouped as a sister taxon to *Nais inornata* (Fig. 4). However, *A. chesapeakensis* is distinctly different from both *L. laevis* and *N. inornata* in perithecial pigmentation, ascus morphology and structure and ascospore wall structure (Jones et al. 2017). Ascomata of *L. laevis* and *N. inornata* can be light brown, but often blackish while those of *A. chesapeakensis* are always hyaline with or without some darkening of the neck (Jones et al. 2017). In addition, asci of *L. laevis* are thin-walled and lack an apical pore, while *A. chesapeakensis* are thick-walled and with an apical pore and retraction of plasmalemma (Shearer and Miller 1977; Sakayaroj et al. 2011). Sakayaroj et al. (2011) and Jones et al. (2017) demonstrated that the genus is polyphyletic and later species were referred to new taxa e.g. *Aniptosporopsis* and *Paraaniptodera* (Jones et al. 2017).

##### Molecular evaluation.

Sequence data of 28S and 18S rDNA are available for four species: *A. aquibella*, *A. chesapeakensis*, *A. limnetica* and *A. salsuginosa* (Shearer 1989; Nakagiri and Ito 1994; Volkmann-Kohlmeyer and Kohlmeyer 1994; Li et al. 2016; Jones et al. 2017), and is only available for the holotype of one species (*A. aquibella*), not for the type species. In this study, *A. aquibella*, *A. limnetica* and *A. salsuginosa* were found to be phylogenetically unrelated to the type species *A. chesapeakensis* (Fig. 4), therefore, new genera, *Sheareromyces*, *Pangia* and *Shiiraspora*, are introduced to accommodate these three species in this study, respectively.

The great morphological variability of taxa assigned to *Aniptodera* as stated above, and Nakagiri and Ito (1994), indicates that the genus is weakly delineated and that further taxa may be excluded from the genus. Confusion as to whether the type species has or lacks polar appendages contributes to this. Clearly all taxa assigned to the genus require recollection, isolation and sequencing, before they can be accepted in the Halosphaeriaceae. Our time-calibrated phylogeny further supports this view, showing that the split between *Aniptodera* and *Shiiraspora* dates back to ~301 MYA (95% CI: 123–735 MYA), highlighting their long independent evolutionary histories (Fig. 5).

#### Aniptodera chesapeakensis

Taxon classificationFungiMicroascalesHalosphaeriaceae

Shearer & M.A. Mill., Mycologia 69(5): 894 (1977)

5A3B26AF-DF35-5323-93A9-204353F7DD4A

Index Fungorum: IF308702

Fig. 10

##### Description.

Saprobic on submerged wood in aquatic habitats. **Sexual morph. *Ascomata*** 350–540 µm high, 175–300 µm diam., globose to subglobose, hyaline, greyish brown to dark brown, membranous, superficial or partially immersed, ostiolate. ***Necks*** 100–150 µm long, 40–90 µm diam., apical or subapical portion of the neck becoming dark, forming a ring around the neck, periphysate. ***Peridium*** two-layered, 10–20 µm diam. with an outer layer of cells of *textura angularis* and inner layer of elongate cells. ***Asci*** 70–125 × 15–25 µm, unitunicate, 8-spored, clavate, deliquescing at maturity, with an apical pore, plasmalemma retracted below the apex, short pedicel. ***Catenophyses*** present. ***Ascospores*** 15–25 × 7–10 µm, hyaline, ellipsoidal, thick-walled, 1-septate, not constricted at the septum, appendaged or not. ***Appendages*** bipolar, thin, hamate extending over the mid-septum, unfurling into fine threads in water. **Asexual morph**. Undetermined.

**Figure 10.**
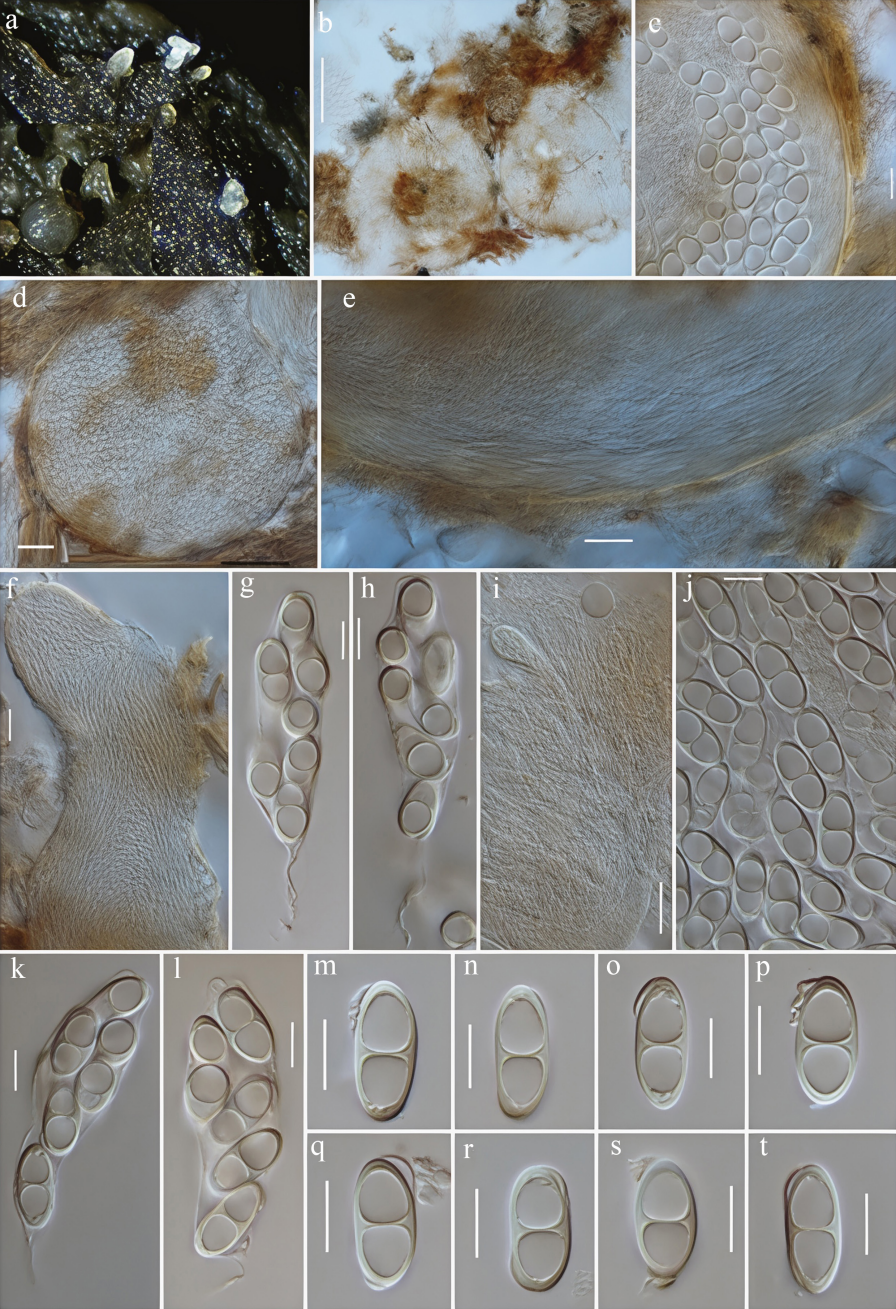
*Aniptodera chesapeakensis*. **a**. Ascomata superficial to semi-immersed in decaying wood; **b, d**. Longitudinal sections of ascomata; **c, e**. Peridium; **f**. Ostiole with periphyses; **i**. Hyaline filamentous paraphyses; **g, h, k, l**. Immature and mature asci; **j, m–t**. Ascospores. Scale bars: 100 μm (**b**); 50 μm (**d, f**); 10 μm (**c, e, g–t**).

##### Material examined.

India, • Tamil Nadu, Tiruvarur, Muthupet mangroves (10.4°N, 79.5°E), on decaying wood of *Avicennia marina* (Acanthaceae), 28 April 2015, B. Devadatha (AMH-10009, holotype).

##### Notes.

The appendage structure and development of *A. chesapeakensis* has not clearly been clarified (Farrant 1986) as it has been reported with and without polar appendages and molecular data is required to determine if this constitutes one taxon (Shearer 1989). However, it is unknown if the isolate of *A. chesapeakensis* in the GenBank was with unfurling appendages (Jones et al. 2017). This species is characterized by pyriform to subglobose ascomata, ostiolate, papillate, hyaline to light brown, catenophyses present, asci clavate to cylindrical, pedunculate, unitunicate, thin-walled, with a refractive pore or plug-like structure at the apex, with a retracting plasmalemma subapically, persistent asci, ascospores ellipsoidal, 1-septate, hyaline, thick-walled, longer than 35 μm (Shearer and Miller 1977). Ascospores are released from the asci through a fissure made at the refractive pore or plug-like structure (Hyde 1990c).

*Aniptodera intermedia* has the shortest asci in this genus (46–62 × 16–19 μm) and smallest ascospores (10.5–13 × 7–8 μm), while *Aniptodera megaloascocarpa* differs distinctly from all other *Aniptodera* species with the largest ascomata (1060–1360 × 430–530 μm) (Raja and Shearer 2008). *Aniptodera margarition* and *A. mangrovei* lack any apical thickening of the ascus while the subapical retraction of cytoplasm of the former also lacks a distinguishable apical pore characteristic of all *Aniptodera* species (Shearer 1989). *Aniptodera triseptata* is the only species with 3-septate ascospores in the genus (Hyde 2002). *Aniptodera aquibella* differs from other species in the genus by conspicuous differences in the size and shape of asci (60–110 × 25–45 μm) and ascospores (25–30 × 7–10 μm) (Li et al. 2016). Further, *Aniptodera mangrovei* is described as having an apical ring with a pore at the ascus apex and appendaged ascospores (Hyde et al. 1986), but their morphology differs from those of *A. salsuginosa* and *A. chesapeakensis*. This species also differs from the latter two taxa by lacking cytoplasmic retraction in the subapical region of ascus (Hyde et al. 1986; Hyde 1992c). *Aniptodera limnetica* and *A. fusiformis* have non-appendaged ascospores, though these two species have similar ascus structure to that of *A. salsuginosa* and *A. chesapeakensis* (Shearer 1989; Hyde 1990c). *Aniptodera margarition* is different from all other *Aniptodera* species in that it lacks an apical apparatus and apical pore in the asci (Nakagiri and Ito 1994). However, concentric undulation of the apical disc is found only in *A. salsuginosa* and ascospore discharge through the fissure between the apical disc and ascus wall is unique to the species. Forcible ejection of ascospores through the apical pore has been recorded for *A. chesapeakensis* (Farrant 1986) and the nonfunctional apical pore in the ascus of *A. salsuginosa* may indicate that this fungus was derived from a terrestrial origin (Nakagiri and Ito 1994).

Jones et al. (2017) reported that *A. lignatilis* and *A. longispora* are distantly positioned from *A. chesapeakensis* based on the combined 28S and 18S rDNA phylogenetic analyses and referred them to the new genera *Aniptosporopsis* and *Paraaniptodera*, respectively (Li et al. 2016; Jones et al. 2017). Furthermore, *A. chesapeakensis* and *A. aquibella* do not group in a single clade (Li et al. 2016; Jones et al. 2017) and this is supported by our combined phylogenetic analysis (Fig. 4). However, further taxon sampling and extended sequence data are required to confirm true *Aniptodera* species among the currently described nineteen taxa.

##### Distribution.

Andaman Island, Australia, Belize, Brunei, China, Egypt, England, Hong Kong, India, Indonesia, Japan, Malaysia, Mauritius, Mexico, Panama, Philippines, Portugal, Seychelles, Singapore, South Africa, Sri Lanka, Taiwan, Thailand, USA, and Venezuela (https://www.marinefungi.org).

### *Aniptosporopsis* K.L. Pang, C.L. Lu, W.T. Ju & E.B.G. Jones

*Aniptosporopsis* was introduced by Jones et al. (2017) to include *Aniptodera lignatilis* which did not group with the type species (*A. chesapeakensis*) in a phylogenetic study. It is a monotypic genus and reported from Australia, Taiwan, Malaysia and Thailand (Pang et al. 2011b).

#### Aniptosporopsis

Taxon classificationFungiMicroascalesHalosphaeriaceae

K.L. Pang, C.L. Lu, W.T. Ju & E.B.G. Jones, Bot. Mar. 60 (4): 459 (2017)

9414485F-046C-5BDB-BF7D-E93421FDE263

Index Fungorum: IF818199

##### Description.

Saprobic on submerged wood in freshwater habitats and mangrove wood. **Sexual morph. *Ascomata*** globose, subglobose to occasionally pyriform, hyaline to cream colored, membranous, immersed to semi-immersed, becoming superficial, solitary or gregarious, ostiolate. ***Necks*** light colored, periphysate. ***Peridium*** composed of 3–6 layers of thick-walled elongate cells. ***Catenophyses*** present. ***Asci*** unitunicate, 8-spored, clavate, persistent with a flattened apex and an indistinct apical pore, plasmalemma retracted below the apex, pedicellate. ***Ascospores*** hyaline, fusiform, thick-walled, septate, not constricted at the septum, appendaged. ***Appendages*** bipolar, initially hamate extending over the central septum, unfurling to form fine threads in water. **Asexual morph**. Undetermined.

##### Type species.

*Aniptosporopsis lignatilis* (K.D. Hyde) K.L. Pang, C.L. Lu, W.T. Ju & E.B.G. Jones, Bot. Mar. 60 (4): 459 (2017).

##### Notes.

Campbell et al. (2003) first obtained the sequence data for *A. lignatilis* only for the 28S rDNA locus. It grouped in the same clade with *Aniptodera chesapeakensis* but as a distinct basal lineage and this was supported by Sakayaroj et al. (2011). Subsequently, Jones et al. (2017) showed that *A. lignatilis* grouped adjacent to *Aniptodera chesapeakensis* but as a separate lineage. In the phylograms of Campbell et al. (2003), Sakayaroj et al. (2011) and Jones et al. (2017), this species did not group with the type species of *Aniptodera*, but formed a sister clade to a clade comprising *Okeanomyces cucullatus*, *Aniptodera chesapeakensis*, *Aniptodera aquibella*, *Naïs inornata*, *Ascosacculus aquaticus* and *Thalespora appendiculata*, a group with few morphological features in common with *A. lignatilis*. Jones et al. (2017) established the genus *Aniptosporopsis* to accommodate *Aniptodera lignatilis*. In our phylogenetic analysis (Fig. 4) *Aniptosporopsis lignatilis* grouped with two strains of *Ascoglobospora marina*, and was distantly placed from the type species of *Aniptodera chesapeakensis*.

##### Molecular evaluation.

*Aniptosporopsis* is a well-established monotypic genus in the Halosphaeriaceae, which represents an independent lineage that diverged from its closest relatives during the mid-Cretaceous to Cenozoic (~36.7 MYA, 95% CI: 11.7–114.8 MYA), supporting its recognition as a distinct genus (Fig. 5).

#### Aniptosporopsis lignatilis

Taxon classificationFungiMicroascalesHalosphaeriaceae

(K.D. Hyde) K.L. Pang, C.L. Lu, W.T. Ju & E.B.G. Jones, Bot. Mar. 60 (4): 459 (2017)

2542A110-FDC4-5489-B4F2-396237943903

Index Fungorum: IF818209

Aniptodera lignatilis K.D. Hyde, Austral. Syst. Bot. 5: 111 (1992). Basionym.

##### Description.

Saprobic on submerged wood in freshwater habitats and mangrove wood. **Sexual morph. *Ascomata*** 225–400 μm high, 160–350 μm diam., globose, subglobose to occasionally pyriform, hyaline to cream colored, membranous, immersed to semi-immersed, becoming superficial, solitary or gregarious, ostiolate. ***Necks*** light-colored, periphysate. ***Peridium*** 18–23 μm thick, 3–6 layers of thick-walled elongate cells. ***Catenophyses*** present. ***Asci*** 128–171 × 28–40 μm, unitunicate, 8-spored, clavate, long pedicellate, persistent with a flattened apex and an indistinct apical pore, plasmalemma retracted below the apex, pedicellate. ***Ascospores*** 33–55 × 12–17 μm, hyaline, fusiform, thick-walled, 1-septate, not constricted at the septum, appendaged, bipolar, initially hamate extending over the central septum, unfurling to form fine threads in water. **Asexual morph**. Undetermined.

##### Material examined.

Australia, • Atherton Tablelands, Millaa Falls, on submerged log in freshwater, 1990, K. D. Hyde, BRIP 17156.

##### Notes.

*Aniptosporopsis lignatilis* is a saprobe occurring on submerged wood in freshwater habitats and on mangrove wood (Hyde 1992a; Pang et al. 2011b; Jones et al. 2017). This species is widely distributed with records from Australia, Hong Kong, Macau, Malaysia, Mauritius, Philippines, Seychelles, South Africa, Taiwan, Thailand, and USA (https://www.marinefungi.org, https://www.freshwaterfungi.org).

### *Anisostagma* K.R.L. Petersen & Jørg. Koch

*Anisostagma* was proposed by Petersen and Koch (1996) for a lignicolous marine ascomycete discovered from a harbour environment at the Bresund coast, Denmark.

#### Anisostagma

Taxon classificationFungiMicroascalesHalosphaeriaceae

K.R.L. Petersen & Jørg. Koch, Mycol. Res. 100: 209 (1996)

95C3B02E-A79B-5528-B01F-DD1D4E2FD6A1

Index Fungorum: IF27558

##### Description.

Saprobic on oak (*Quercus* sp.) wood tidal water region. **Sexual morph. *Ascomata*** single or gregarious, globose to broadly ellipsoidal, immersed to partly immersed, ostiolate with a neck, periphysate, cream-colored to light-brown. ***Peridium*** 2-layered, outer layer forming a *textura angularis*. ***Catenophyses*** present. ***Asci*** 8-spored, clavate, pedunculate, thin-walled, unitunicate, without an apical apparatus, early deliquescing, developing from a small cushion at the base of the ascoma venter. ***Ascospores*** globose to ellipsoidal, aseptate, hyaline, with one large globule surrounded by numerous droplets, without appendages. **Asexual morph**. Undetermined.

##### Type species.

*Anisostagma rotundatum* K.R.L. Petersen & Jørg. Koch, Mycol. Res. 100: 211 (1996).

##### Notes.

*Anisostagma* was assigned to the Halosphaeriaceae (Müller and von Arx 1962; Kohlmeyer 1972) because of the early deliquescing, thin-walled asci, the presence of catenophyses and the occurrence in the marine environment. Peridium of *Anisostagma* is composed of two-layers and asci do not have an apical apparatus. Further, *Anisostagma* has spherical-ellipsoidal ascospores which are hyaline and contain one large globule surrounded by small droplets and no appendages or sheaths at the light microscope level (Petersen and Koch 1996). *Anisostagma* is saprobic on oak (*Quercus* sp.) wood in the tidal zone (Petersen and Koch 1996).

This genus currently comprises a single species *Anisostagma rotundatum* and is only known from Denmark (Petersen and Koch 1996) and is the only record on this taxon hence, this species seems to be rare in the environment.

##### Molecular evaluation.

As there are no cultures or sequence data available for the type material, recollection, isolation and sequencing is required to firmly place this taxon in the Halosphaeriaceae. *Anisostagma rotundatum* is accepted in the Halosphaeriaceae on its morphological features (Jones et al. 2019).

#### Anisostagma rotundatum

Taxon classificationFungiMicroascalesHalosphaeriaceae

K.R.L. Petersen & Jørg. Koch, Mycol. Res. 100: 211 (1996)

033DD701-6B1C-5AFA-8926-A79AF1B62E03

Index Fungorum: IF414610

Fig. 11

##### Description.

Saprobic on wood in marine habitat. **Sexual morph. *Ascomata*** 230–300 × 180–260 µm, solitary or gregarious, globose to broadly ellipsoidal, immersed or partly immersed, coriaceous, light brown, ostiolate with a long neck. ***Peridium*** 35–48 µm thick, 2-layered, inner layer composed of flattened thin-walled cells, outer cells forming a *textura angularis*. ***Necks*** 250–370 × 40–66 µm, cylindrical. ***Catenophyses*** septate with slightly swollen end cells. ***Asci*** 90–120 × 30–42 µm, 8-spored, pedunculate, clavate, unitunicate, thin-walled, early deliquescing, without an apical apparatus, developing from a small cushion at the base of the ascoma venter. ***Ascospores*** 18–22 × 14–20 µm, one-celled, globose to ellipsoidal, hyaline, thin-walled, smooth, without appendages or gelatinous sheaths, with one large globule surrounded by numerous droplets most prominent at the poles. **Asexual morph**. Undetermined.

**Figure 11.**
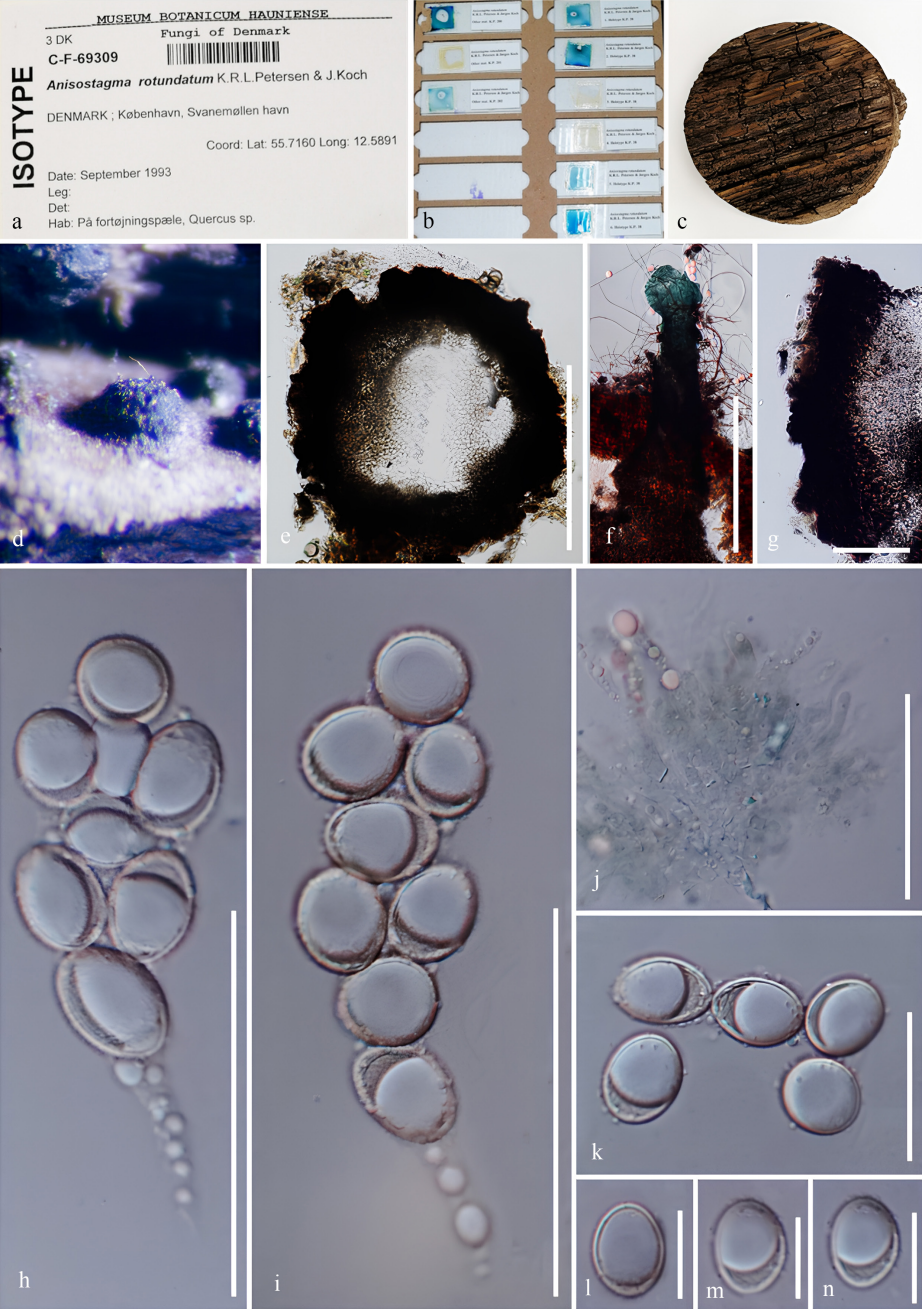
*Anisostagma rotundatum* (C-F-69309, micro-slides from isotype). **a**. Herbarium material; **b**. Micro slides; **c, d**. Ascomata on wood; **e**. Section through ascoma; **f**. Neck region; **g**. Peridium; **h, i**. Asci; **j**. Paraphyses; **k–n**. Ascospores. Scale bars: 100 µm (**e**); 50 µm (**f, h, i**); 20 µm (**g, j–n**).

##### Material examined.

Denmark, • Copenhagen, Svanemollen Harbour, From mooring posts of oak, Aug. 1993, K.R.L. Petersen, C-F-69309, 9 micro slides from isotype.

##### Notes.

*Anisostagma rotundatum* was isolated on wood discs removed from mooring posts of oak (*Quercus* sp.) at Svanemollen Harbour, at the Øresund coast, Copenhagen, Denmark. *Anisostagma rotundatum* shares most similar ascospore morphologies to *Thalassogena sphaerica*, *Iwilsoniella rotunda* and *Hapsidascus hadrus* (Petersen and Koch 1996). These three species have spherical-ellipsoidal ascospores which are hyaline and composed of a one large guttule surrounded by small droplets (Kohlmeyer and Volkmann-Kohlmeyer 1987a; Jones 1991). In addition, ascospores of the four species do not have appendages or sheaths at the light microscope level (Kohlmeyer and Volkmann-Kohlmeyer 1987a; Jones 1991; Petersen and Koch 1996). *Anisostagma rotundatum* can be differentiated from *Thalassogena sphaerica* (the type species) by having two layered peridium while it is undifferentiated in the latter species (Kohlmeyer and Volkmann-Kohlmeyer 1987a). Furthermore, the asci of *Thalassogena sphaerica* has an apical pore (Kohlmeyer and Volkmann-Kohlmeyer 1987a), whereas *Anisostagma rotundatum* asci lack an apical apparatus. When considering the morphological differences of *Anisostagma rotundatum* and *Iwilsoniella rotunda*, ascomata of *I. rotunda* are dark brown and catenophyses and periphyses are lacking, while *A. rotundatum* has light brown ascomata and well distinguishable catenophyses (Jones 1991). *Hapsidascus hadrus* differs from *Anisostagma* in having ascomata that are larger with a wide neck and periphyses-like hyphae and a peridium composed of three layers. Furthermore, it has thin-walled, early deliquescing paraphyses and asci with a net-like apical apparatus inside the ascus wall.

### *Antennospora* Meyers

Cribb and Cribb (1956) described *Halosphaeria quadricornuta* from *Avicennia marina* var. *resiniferae* collected at Redcliffe, Australia. Johnson (1958) transferred *H. quadricornuta* to *Antennospora*, a genus established by Meyers (1957), and synonymized *A. caribbea*. Kohlmeyer (1972) revised the family Halosphaeriaceae and transferred *A. quadricornuta* back to *Halosphaeria*. Later, Jones and Moss (1980) and Jones et al. (1984), based on ultrastructural data, reassigned it to *Antennospora*. Kohlmeyer (1984) had difficulty in accepting the taxonomic status of *Antennospora* as he opined that the similarity in ascospore appendage ontogeny of both *Halosphaeria appendiculata* and *H. quadricornuta* (arising as outgrowths from the mesosporium) did not warrant generic separation. Pang et al. (2008a) suggested that *Antennospora* (*A. quadricornuta*) and *Halosphaeria* (*H. appendiculata*) should be kept as separate genera as inferred from the analyses of 28S rDNA sequence data, although the relationships of the two species with other taxa in the Halosphaeriaceae remain unresolved. *Halosphaeria appendiculata* is always located within a well-supported branch and this result has also been reported in other studies (Pang et al. 2003a, 2003b, 2004a). However, due to the great morphological variations exhibited by these taxa, the apomorphic character(s) supporting this group is (are) unknown.

Yusoff et al. (1994b) transferred *Halosphaeria salina* to *Antennospora* based on morphological similarities, such as, ascospore appendages that are outgrowths of the mesosporium and connected by an isthmus of electron-dense material, cylindrical appendages with a deep spoon-shaped structure at the point of attachment. However, Pang et al. (2008a) excluded *Antennospora salina* based on phylogenetic evidence and referred it to the new genus *Haiyanga*. Therefore, this genus currently comprises a single species *Antennospora quadricornuta* (Pang et al. 2008a).

#### Antennospora

Taxon classificationFungiMicroascalesHalosphaeriaceae

Meyers, Mycologia 49: 501 (1957)

4F09A846-0086-5FF3-BBD4-434974A39E81

Index Fungorum: IF219

##### Description.

Saprobic on submerged marine wood, oceanic. **Sexual morph. *Ascomata*** solitary or gregarious, subglobose to ellipsoidal, immersed or becoming superficial, papillate, ostiolate, without periphyses, subcarbonaceous, dark brown to black. ***Peridium*** composed of 3–4 layers of irregular, polygonal thick-walled cells and thin-walled, large and deliquescing pseudoparenchyma. ***Asci*** 8-spored, clavate, deliquescing early and formed from a pad of ascogenous tissue. ***Ascospores*** ellipsoidal, 1-septate, slightly constricted at the septum, hyaline and appendaged. ***Appendages*** two (rarely 3 or 4) subterminal appendages at each end of the spore, cylindrical, attenuate and stiff. **Asexual morph**. Undermined.

##### Type species.

*Antennospora quadricornuta* (Cribb & J.W. Cribb) T.W. Johnson, J. Elisha Mitchell Sci. Soc. 74: 46 (1958).

##### Notes.

*Antennospora*, initially a monotypic genus, shares morphological similarities to the genus *Halosphaeria* (Jones et al. 1984) but it is distinct by having subterminal appendages, the absence of an episporium at the point of appendage attachment and lacking equatorial appendages. *Antennospora* also shares similar morphologies to the genus *Haiyanga* by having subterminal appendages and parallel striations running the length of appendages (Yusoff et al. 1994b). However, asci of *Haiyanga* species occur on a pad at the base of the ascoma while in *Antennospora* asci are formed from a pad of ascogenous hyphae (Yusoff et al. 1994b). Furthermore, ascospore appendages of *Antennospora* are shorter than those of *Haiyanga* species (Yusoff et al. 1994b).

The ascospore wall of *A. quadricornuta* is composed of a mesosporium and episporium, but without an exosporium (Jones et al. 1984). The episporium is absent at the point of appendage attachment, being replaced by an isthmus of electron-dense material connecting the appendage to the mesosporium (Jones et al. 1984). The appendages are initially closely wrapped around the spore but then uncoil to form the rigid appendages (Jones et al. 1984). Appendages have an electron-dense boundary layer enclosing a peripheral region of electron-dense fibrils running parallel to the long axis of the appendage and an electron-transparent core containing amorphous material (Jones et al. 1984).

##### Molecular evaluation.

*Antennospora quadricornuta* is a well-established genus in the Halosphaeriaceae supported by morphology and sequence data (Fig. 4), and our time-calibrated phylogeny indicates that it diverged from related lineages during the mid-Cretaceous (~111 MYA, 95% CI: 34.8–352.2 MYA), further supporting its independent generic status (Fig. 5).

#### Antennospora quadricornuta

Taxon classificationFungiMicroascalesHalosphaeriaceae

(Cribb & J.W. Cribb) T.W. Johnson, J. Elisha Mitchell Sci. Soc. 74: 46 (1958)

F9C79F9F-F6E4-514C-994D-CC556F7F2F88

Index Fungorum: IF292555

Fig. 12

Halosphaeria quadricornuta Cribb & J.W. Cribb, Pap. Dept. Bot. Univ. Queensland 3 (12): 99 (1956). Basionym.
Antennospora caribbea Meyers, Mycologia 49(4): 503 (1957). Synonymy.

##### Description.

Saprobic on submerged wood. **Sexual morph. *Ascomata*** 130–260(–514) μm high, 140–285 μm diam., solitary or gregarious, subglobose to ellipsoidal, immersed or becoming superficial, papillate, ostiolate, without periphyses, subcarbonaceous, dark-brown to black. ***Necks*** 70–560 μm long, 20–70(–93) μm diam., subconical or cylindrical, centric or eccentric, ostiolar canal indistinctly periphysate. ***Peridium*** 9–12.5 μm thick, composed of 3–4 layers of irregular, polygonal thick-walled cells and thin-walled, large and deliquescing pseudoparenchyma. ***Asci*** 90–120 × 30–42 µm, 8-spored, clavate, deliquescing early and formed from a pad of ascogenous tissue. ***Ascospores*** 20–35 × 6–12 µm (excluding appendages, ellipsoidal, 1-septate, slightly constricted at the septum, hyaline and appendaged. ***Appendages*** 20–37 μm long, 1–2 μm diam., two (rarely 3 or 4) subterminal appendages at each end of the spore, cylindrical, attenuate and stiff. **Asexual morph**. Undetermined.

**Figure 12.**
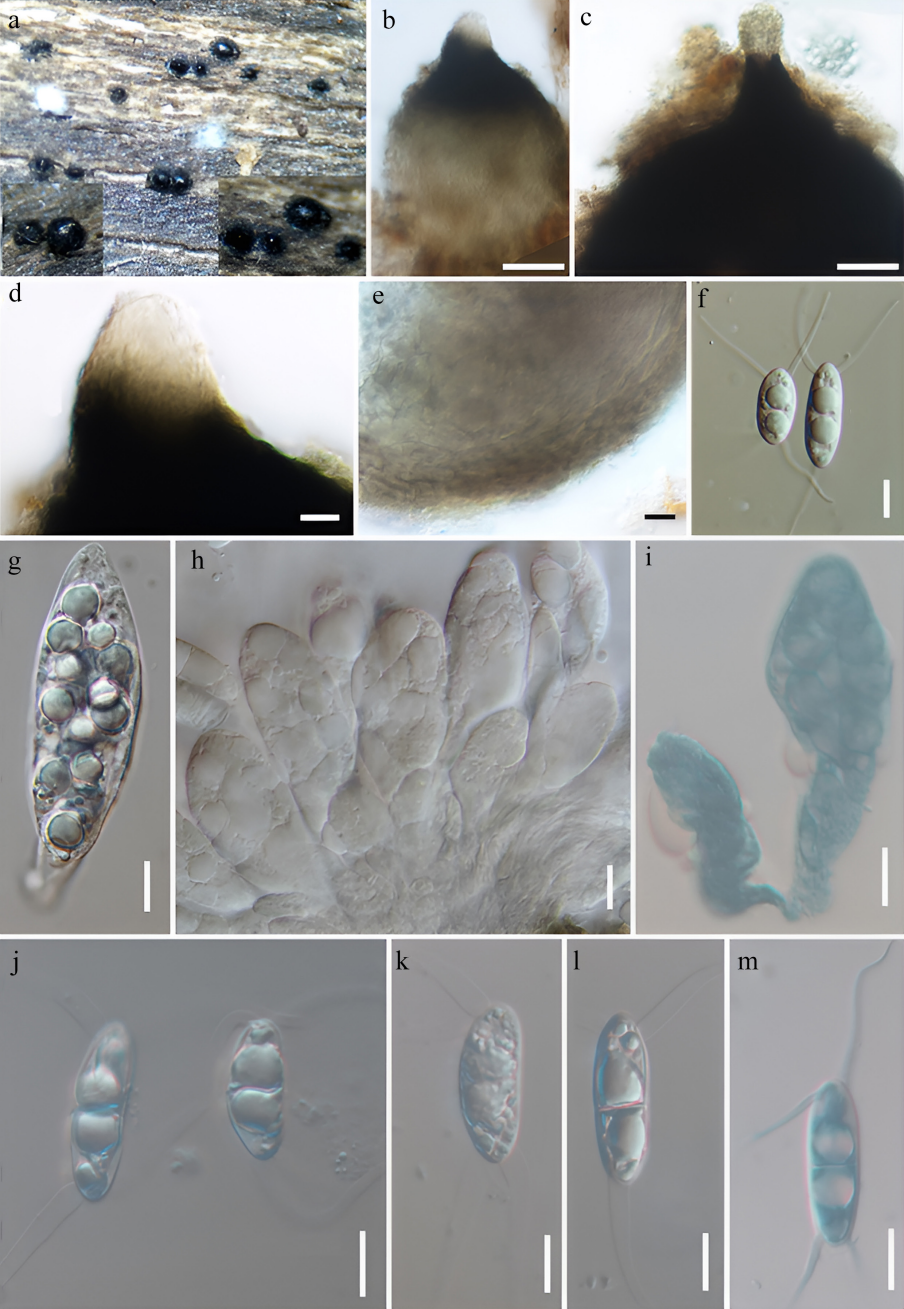
*Antennospora quadricornuta*. **a**. Ascomata superficial to semi-immersed in the decaying wood; **b, c**. Ascomata; **d**. Ostiole; **e**. Peridium; **f, j–m**. Ascospores; **g–i**. Immature and mature asci. Scale bars: 50 μm (**b, c**), 10 μm (**d–m**).

##### Material examined.

India, • Tamil Nadu, Tiruvarur, Muthupet mangroves (10.4°N, 79.5°E), on decaying wood of *Avicennia marina* (Acanthaceae), 28 April 2015, B. Devadatha (AMH-9994).

##### Notes.

*Antennospora quadricornuta* is widely distributed in the tropics and sub-tropics (Pang et al. 2008a). Most collections of *A. quadricornuta* have been reported from mangrove wood (*Avicennia marina*, *Aegiceras corniculatum*, *Bruguiera gymnorrhiza*, *Cocos nucifera*, *Hibiscus tiliaceus*, *Prosopis* sp., *Pithecellobium* sp., *Rhizophora apiculata*, *Rh. mangle*, *Rh. mucronata*, *Sonneratia alba*, *Sonneratia griffithii*, test blocks of *Albizia*) (Kohlmeyer 1969, 1984; Vrijmoed et al. 1982a, 1982b, 1986a, 1986b; Suhirman and Jones 1983; Kohlmeyer and Volkmann-Kohlmeyer 1987b; Hyde 1989; Hyde and Jones 1989; Hyde et al. 1990; Chinnaraj 1993; El-Sharouney et al. 1998; Prasannarai et al. 1999; Poonyth et al. 2001) as saprobes. Vrijmoed and Hodgkiss (1988) showed that it caused decay of wood under laboratory conditions.

Phylogenetic analyses of Pang et al. (2008a) using the 28S rDNA sequence data confirmed its placement within Halosphaeriaceae (Pang et al. 2003a, 2003b, 2004a, 2008a). Pang et al. (2008a) showed that *Antennospora salina* is not related phylogenetically to *A. quadricornuta* but forms a rather well-supported group with *Arenariomyces trifurcatus* hence, excluded from *Antennospora* and referred to the genus *Haiyanga* (Pang et al. 2008a). This was subsequently supported by the studies of Sakayaroj et al. (2011) and Jones et al. (2017). The multi-gene analysis in this study revealed that *Haiyanga salina* grouped within the genus *Arenariomyces* (Fig. 4), and consequently, this species is referred back to *Arenariomyces*.

##### Distribution.

Antigua and Barbuda, Australia, Bahamas, Belize, Bermuda, Brunei, Colombia, Cuba, Haiti, Egypt, Fiji, Galapagos, Hong Kong, India, Japan, Kuwait, Liberia, Malaysia, Martinique, Mexico, Mozambique, Panama, Philippines, Puerto Rico, Republic and Trinidad and Tobago, Seychelles, Singapore, Taiwan, Tahiti, Thailand, USA, Venezuela, Yemen (https://www.marinefungi.org).

### *Appendichordella* R.G. Johnson, E.B.G. Jones & S.T. Moss

This fungus was first described by Kohlmeyer (1962a) and placed in *Sphaerulina*, but since the asci are unitunicate and deliquesce, this was not an appropriate genus. Kohlmeyer and Kohlmeyer (1979) then transferred the species to *Haligena*, but the appendages are attached to the ascospore wall primarily in the polar region. Johnson et al. (1987) introduced the genus *Appendichordella* to accommodate *Haligena amicta*. In *Haligena* the polar appendages arise as outgrowths of the spore wall, while in *H. amicta* they develop from the episporium (Johnson et al. 1984). *Haligena amicta* is also quite distinct from the type of *Haligena* (*H. elaterophora*) by having undifferentiated peridium, lacking catenophyses, and by the absence of polar appendages.

#### Appendichordella

Taxon classificationFungiMicroascalesHalosphaeriaceae

R.G. Johnson, E.B.G. Jones & S.T. Moss, Canad. J. Bot. 65 (5): 941 (1987)

3DAA6FC1-DC76-5FB6-B587-0C4719D38475

Index Fungorum: IF25087

##### Description.

Saprobic on rotten drift wood. **Sexual morph. *Ascomata*** solitary, globose to subglobose, usually immersed, coriaceous, light to reddish brown, ostiolate, papillate, with periphyses. ***Peridium*** composed of 4–5 layers of elongate and thick-walled cells and thin-walled pseudoparenchyma that are deliquescing at maturity. ***Catenophyses*** absent. ***Asci*** 8-spored, clavate, pedunculate, unitunicate, thin-walled, without apical apparatus, deliquescing early and developing at the base of the ascocarp. ***Ascospores*** cylindrical or ellipsoidal, 3-septate, slightly constricted at the septa, hyaline and enclosed in a gelatinous sheath composed of tightly coiled appendages which swell in water to form thread-like looping appendages. **Asexual morph**. Undetermined. (Description adopted from Johnson et al. 1987).

##### Type species.

*Appendichordella amicta* (Kohlm.) R.G. Johnson, E.B.G. Jones & S.T. Moss, Canad. J. Bot. 65 (5): 941 (1987).

##### Notes.

*Appendichordella* most closely resembles *Carbosphaerella* with its gelatinous, persistent, irregular, and sheath-like appendage which becomes fibrillar, the appendages developing by fragmentation of the exosporium (Johnson et al. 1984). However, the phylogenetic position of this genus within Halosphaeriaceae is provisional due to the lack of molecular data.

##### Molecular evaluation.

Sequence data is not available for this species. Recollection, isolation, and sequencing are required to determine its inclusion in the Halosphaeriaceae.

#### Appendichordella amicta

Taxon classificationFungiMicroascalesHalosphaeriaceae

(Kohlm.) R.G. Johnson, E.B.G. Jones & S.T. Moss, Canad. J. Bot. 65 (5): 941 (1987)

FBAD27B4-7579-5EFE-AA55-9A0DBD62A068

Index Fungorum: IF130709

Fig. 13

Sphaerulina amicta Kohlm., Nova Hedwigia 4: 414 (1962). Basionym.
Haligena amicta (Kohlm.) Kohlm. & E. Kohlm., Marine Mycology: The higher fungi: 280 (1979). Synonymy.

##### Description.

Saprobic on rotten drift wood. **Sexual morph. *Ascomata*** 187–360 × 205–340 μm solitary, globose to subglobose, or depressed ellipsoidal, immersed, ostiolate, papillate, coriaceous, light to reddish-brown, solitary. ***Necks*** 47–220 × 36–44 μm, cylindrical, ostiolar canal first filled with hyaline, thin-walled pseudoparenchyma and later with papilliform periphyses. ***Peridium*** 16–19 μm wide, composed of 4–5 layers of elongate and thick-walled cells and thin-walled pseudoparenchyma that deliquesce at maturity. ***Catenophyses*** absent. ***Asci*** 48–72 × 15–28 μm, 8-spored or rarely 2 spored, clavate, pedunculate, unitunicate, thin-walled, without apical apparatus, deliquescing early and developing at the base of the ascocarp. ***Ascospores*** (15–)18–25.5(–27) × (7–)8–11.5 μm, cylindrical or ellipsoidal, 3-septate, slightly constricted at the septa, hyaline and enclosed in a gelatinous sheath, 3–6 μm thick, composed of tightly coiled appendages which swell in water to form thread-like looping appendages. **Asexual morph**. Undetermined. (Description adopted from Kohlmeyer and Kohlmeyer 1979; Johnson et al. 1987).

**Figure 13.**
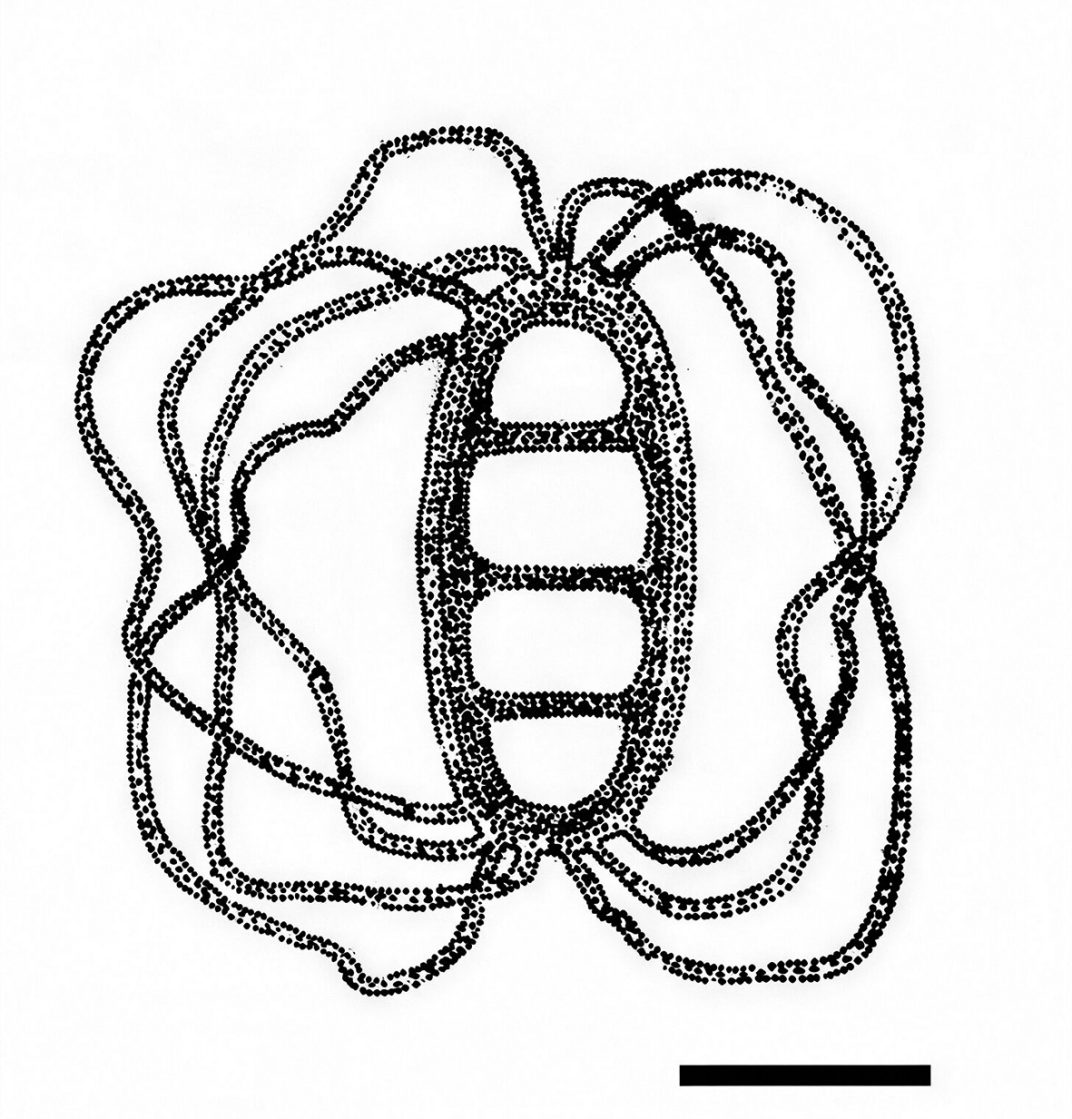
Ascospore of *Appendichordella amicta* (Redrawn from Johnson et al. 1987). Scale bar: 10 mm.

##### Notes.

*Appendichordella amicta* was introduced as *Sphaerulina amicta* (Kohlmeyer 1962a) but we could not locate the type specimen for our study as the original author has not designated a type specimen. This species was first discovered from rotten wood in seawater on the Atlantic coast, France (Kohlmeyer 1962a). Furthermore, there is no authentic specimen to observe from subsequent collections. This species has been subjected to a histochemical and scanning and transmission electron microscope level studies by Johnson et al. (1987) but there are no illustrations of other structures.

The sheath surrounding the ascospores of *A. amicta* is unique in its structure; it is composed of tightly coiled appendages which swell in water to form thread-like looping appendages (Fig. 13) which arise from the episporium. The species lacks polar appendages and catenophyses (Kohlmeyer and Kohlmeyer 1979). The spore wall is composed of a thick-walled electron-transparent mesosporium and a thin-walled electron-dense episporium from which the thread-like appendages arise and it lacks an exosporium (Johnson et al. 1984). This genus is therefore easily differentiated from all other genera in the Halosphaeriaceae (Shearer and Crane 1980; Jones et al. 1983a, 1984; Johnson et al. 1984).

This little reported species is saprobic on usually well-rotted wood such as *Picea* sp., *Populus* sp. in marine water.

##### Distribution.

Chile, Denmark (N. Jutland), Germany (Karlshagen), Sweden.

### *Arenariomyces* Höhnk

*Arenariomyces* was introduced by Höhnk (1954) with two species, *Arenariomyces cinctus* and *Arenariomyces trifurcatus*, but no type material for either the genus or species was designated or deposited. Subsequently, Kohlmeyer (1960) assigned *Arenariomyces trifurcatus* to *Peritrichospora* (Kohlmeyer 1961) and later to *Corollospora* (Kohlmeyer 1962b). Subsequently, Jones et al. (1983a) in an SEM/TEM study showed that the appendages of *Arenariomyces* differed from those of *Corollospora*, and reestablished the genus *Arenariomyces*. Jones et al. (2009) recognized five species *A. majusculus*, *A. parvulus*, *A. trifurcatus*, *A. triseptatus* and *A. truncatellus*. A further *Arenariomyces* species was introduced by Meyers (1957) as *Arenariomyces salinus*, but a subsequent molecular study assigned it to the genus *Haiyanga* (Pang et al. 2008a).

#### Arenariomyces

Taxon classificationFungiMicroascalesHalosphaeriaceae

Höhnk, Veröff. Inst. Meeresf. Bremerhaven 3: 28 (1954)

10CD9DCC-B847-5FC9-9759-48770DB713D4

Index Fungorum: IF303

##### Description.

Saprobic on wood, sand or calcareous substrates. **Sexual morph. *Ascomata*** solitary, sometimes gregarious, globose to subglobose, papillate, periphyses absent, carbonaceous, dark brown to black, superficial or immersed, sometimes fully or slightly covered with scattered brownish black spines, subiculum present on materials such as sand grains and shells. ***Peridium*** variable in arrangement of cells, usually comprised of dark, thick-walled cells, cell spherical to subsphaerical with inconspicuous pit connections. ***Pseudoparenchyma*** thin-walled, and deliquescing at maturity. ***Catenophyses*** absent or deliquescing if present. ***Asci*** 8-spored, fusiform to subclavate or pyriform, short-stalked, unitunicate, thin-walled and deliquescing early. ***Ascospores*** 1–3 septate, fusiform, cylindrical, ellipsoidal or oblong, constricted/slightly constricted at the septa, hyaline and appendaged. ***Appendages*** 3-subterminal appendages at each end of the spore, straight or curved, with a bulbose base and long attenuated arms initially wrapped along the length of the ascospore and unfolding at maturity. **Asexual morph**. Undetermined.

##### Type species.

*Arenariomyces trifurcatus* Höhnk, Veröff. Inst. Meeresf. Bremerhaven 3: 30 (1954).

##### Notes.

*Arenariomyces* species are saprobes on wood and in wood associated with sand grains, the mycelium growing out from the wood into the surrounding sand, and forming fruiting bodies on sand particles (Jones et al. 1983a; Kohlmeyer and Volkmann-Kohlmeyer 1989; Jones et al. 2009; Koch 2013). Also reported on dead mangrove wood, mangrove leaves, seaweeds, seagrasses, sand and sea foam. They are mostly reported from temperate parts of the world (Jones et al. 2009; Koch 2013).

Morphologically, this genus is distinguished from *Corollospora* by the ascospores lacking equatorial appendages, primary polar and secondary exosporial appendages, and presence of subterminal appendages, appendages being outgrowths of the spore wall and composed of a bulbous base and rigid spine terminating in a bifurcated or disc-like structure and lacking an exosporium (Jones et al. 1983a). *Arenariomyces truncatellus* and *A. majusculus* ascomata are formed in the outermost part of the wood substrate and bearing spines in contrast to *A. parvulus*, *A. trifurcatus*, and *A. triseptatus* (Jones et al. 2009). However, the stability of ascomatal characters in the genus is variable (on wood/ on hard surfaces, spines/ no spines). *Arenariomyces majusculus* is lignicolous with ascomata embedded in wood, in contrast to other species which form subicula on grains of sand or calcareous substrates (Kohlmeyer and Volkmann-Kohlmeyer 1989). *Arenariomyces* species are different from each other mainly by ascospore septation and dimensions; *A. majusculus* (33 × 11.5 µm), *A. truncatellus* (21.4 × 10.2 µm), *A. parvulus* (16–25 × 3–6 µm), *A. trifurcatus* 28–32 × 9–11 µm are 1-septate while *A. triseptatus* is 3-septate (Kohlmeyer 1984; Koch 2013). Considering the ascospore appendage ontogeny, ascospore appendages are initially wrapped along the length of the ascospore, the appendages unfolding at maturity. Appendages are outgrowths of the spore wall, with a bulbous base, slender, rigid, round shaft, terminating in an apical thickening, pad or bifurcated structure (Jones et al. 1983a). Fully developed spores lacking an exosporium; the episporium laciniated and absent at the point of appendage attachment. The outer surface of the basal third of the appendage composed of 2–4 laminae, the shaft containing a granular core bounded by an electron-opaque homogeneous layer (Jones et al. 1983a).

Molecular sequence data is available for all the *Arenariomyces* species in the GenBank. Pang et al. (2008a), based only on 28S rDNA sequence data, showed that the taxonomic placement of this genus within Halosphaeriaceae is polyphyletic, while with combined 18S and 28S rDNA analyses Jones at al. (2017) showed that *Arenariomyces* species formed a monophyletic group and suggested that the phylogenetic placement of this genus within Halosphaeriaceae is well-resolved.

In this study, the five *Arenariomyces* species formed a monophyletic group with *Haiyanga salina* in the Halosphaeriaceae (Fig. 4) all possessing 3-subterminal appendages at each end of the ascospore and this is supported by molecular data, all with low support (Jones et al. 2017). *Arenariomyces triseptatus*, with 3-septate ascospores and constricted at the septa, differs from other species in the genus.

##### Molecular evaluation.

The genus grouped well within the Halosphaeriaceae (Sakayaroj et al. 2011; Jones et al. 2015) and this is accepted by Maharachchikumbura et al. (2015) and Jones et al. (2019). Our divergence-time analysis indicates that *Arenariomyces* originated from the mid-Cretaceous (~111 MYA, 95% CI: 34.8–352.2 MYA), supporting its recognition as a distinct and independently evolving lineage within the family (Fig. 5).

#### Arenariomyces trifurcatus

Taxon classificationFungiMicroascalesHalosphaeriaceae

Höhnk, Veröff. Inst. Meeresf. Bremerhaven 3: 30 (1954)

12C65433-9996-5755-91FD-567F8924D783

Index Fungorum: IF292682

Figs 3j

Halosphaeria trifurcata (Höhnk) Cribb and J.W. Cribb, Pap. Dept. Bot. Univ. Queensland 3 (12): 99 (1956). Synonym.
Peritrichospora trifurcata (Höhnk) Kohlm., Nova Hedwigia 3: 89 (1961).
Corollospora trifurcata (Höhnk) Kohlm., Ber. Deutsch. Bot. Ges. 75: 126 (1962).

##### Description.

Saprobic on driftwood. **Sexual morph. *Ascomata*** 100–325 µm in diam., globose or subglobose, brown-black, dull, densely covered by short brown hyphae, with subicula on grains of sand, gregarious or solitary, coriaceous, collapsing in drying, ostiole inconspicuous. ***Peridium*** membranous, outer dark layer composed of flat angular cells. ***Asci*** not seen, early deliquescing. ***Ascospores*** 16–22 × 2–3.5 µm, cylindrical, curved, 1-septate, slightly constricted at septa, hyaline with three terminal appendages at both ends; appendages 10–12 µm long, 0.6 µm wide at the base, tapering towards the end, rigid, curved, mostly oriented towards septum. **Asexual morph**. Undetermined

##### Material examined.

Thailand, • Phuket, Patong beach, on driftwood, 30 Oct. 1983, J. Koch, CP1012577 isotype.

##### Notes.

*Arenariomyces trifurcatus* has been referred to various genera, including *Halosphaeria* (Cribb and Cribb 1956), *Peritrichospora* (Kohlmeyer 1962a), *Corollospora* (Kohlmeyer and Kohlmeyer 1979) and finally referred back to *Arenariomyces* (Jones et al. 1983a).

#### Arenariomyces salinus

Taxon classificationFungiMicroascalesHalosphaeriaceae

Meyers, Mycologia 49: 505 (1957)

75F7BDC6-ECDD-54DE-AB34-CE29E5459B5B

Index Fungorum: IF292681

Haiyanga salina (Meyers) K.L. Pang & E.B.G. Jones, Raffles Bull. Zool. 19: 8 (2008). Synonym.
Antennospora salina (Meyers) Yusoff, E.B.G. Jones & S.T. Moss, Mycol. Res. 98 (9): 1003 (1994).
Halosphaeria salina (Meyers) Kohlm., Canad. J. Bot. 50 (9): 1957 (1972).
Remispora salina (Meyers) Kohlm., Mycologia 60: 262 (1968).

##### Notes.

Taxonomically this species, based on morphological characters, has been assigned to various genera. Introduced by Meyers (1957) from *Pinus palustris* wood in seawater in the Caribbean Sea, with hyaline, 1-septate ascospores with three appendages at each end, then referred to *Remispora* by Kohlmeyer (1968) and subsequently to *Halosphaeria* as Kohlmeyer (1972) did not accept the different types of appendages described for various marine Ascomycota. Based on ultrastructural studies it was referred to *Antennospora* by Yusoff et al. (1994b). With molecular studies the species formed a separate clade in the Halosphaeriaceae and a new genus erected as *Haiyanga* (Pang et al. 2008a). In the multi-gene analysis in this study, Haiyanga salina grouped in a highly supported clade within the *Arenariomyces* species clade, and thus is referred to *Arenariomyces*.

##### World distribution.

Argentina, Australia, Bahamas, Canada, Denmark, France, Hawaii, Germany, India, Japan, Mexico, Portugal, Sierra Leone, Spain, Tobago and Trinidad, Yugoslavia, UK, USA.

### *Ascoglobospora* Abdel-Wahab

*Ascoglobospora* was introduced by Abdel-Wahab (in Liu et al. 2024) from driftwood collected from a rocky beach at Umikaze Park, Yokohama, Japan.

#### Ascoglobospora

Taxon classificationFungiMicroascalesHalosphaeriaceae

Abdel-Wahab, Fungal Diversity 124: 58 (2024)

C4EFE55F-673F-5EF8-A920-5697446DA5D1

Index Fungorum: IF559818

##### Description.

Saprobic on decaying driftwood. **Sexual morph. *Ascomata*** globose to subglobose, erumpent to superficial, membranous, hyaline to light-brown, ostiolate, papillate, neck hyaline to light-brown, cylindrical, periphysate. ***Peridium*** one-layered, hyaline, forming *textura angularis*, cell layers consisted of elongated, thick-walled, polygonal hyaline cells. ***Catenophyses*** present. ***Asci*** 8-spored, subglobose, thin-walled, deliquesce early, without an apical apparatus. ***Ascospores*** ellipsoidal, with rounded ends, multiseriate, 1-septate, not constricted at the septum, hyaline, thick-walled, with bipolar, hamate, apical appendages uncoil in water to form long filaments. **Asexual morph**. Undermined.

##### Type species.

*Ascoglobospora marina* Abdel-Wahab, Fungal Diversity 124: 58 (2024).

##### Notes.

*Ascoglobospora* grouped with the genus *Aniptosporopsis* in a well-supported sister clade with high statistical support (100 ML/100 MP/100 BYPP), and morphologically *Ascoglobospora* has early deliquescing asci that are subglobose, thin-walled, without an apical apparatus while *Aniptosporopsis* has persistent, pedunculate, clavate asci with a flattened thickened tip, with an apical pore, plasmalemma retraction, and active spore release (Hyde 1992a; Jones et al. 2017).

##### Molecular evaluation.

Currently two isolates are registered as *Ascoglobospora marina* with molecular sequence data for the 28S and 18S rDNA loci, forming a distinct sister clade with *Aniptosporopsis* with moderate statistical support in the Halosphaeriaceae. Our divergence-time analysis places this split in the late Miocene (95% CI: 0–11.9 MYA), consistent with a relatively recent divergence, suggesting a long independent history (Fig. 5). Further collections and a wider range of genes sequenced are recommended.

#### Ascoglobospora marina

Taxon classificationFungiMicroascalesHalosphaeriaceae

Abdel-Wahab, Fungal Diversity 124: 58 (2024)

B0444FB2-17D3-5229-9E59-11D07DB6E8E8

Index Fungorum: IF559819

Fig. 15

##### Description.

Saprobic on decaying driftwood. **Sexual morph. *Ascomata*** 110–180 μm in diam., globose to subglobose, erumpent to superficial, membranous, hyaline to light-brown, ostiolate, papillate, surrounded by septate hyphae, neck 22–45 μm long, 18–21 μm wide, hyaline to light-brown, cylindrical, periphysate. ***Peridium*** 15–24 μm thick layered, hyaline, forming *textura angularis*, 9–12 cell layers thick, thick walled. ***Catenophyses*** present. ***Asci*** 42–48 × 32–37 μm, 8-spored, subglobose, thin-walled, deliquesce early, without an apical apparatus. ***Ascospores*** 22–30 × 8–9 μm, ellipsoidal, with rounded ends, multiseriate, 1-septate, not constricted at the septum, hyaline, thick-walled (0.5–0.7 μm), with bipolar, hamate, apical appendages, guttulate. ***Appendages*** 15–20 μm long extend beyond the median septum, uncoil in water to form long filaments. **Asexual morph**. Undetermined.

**Figure 14.**
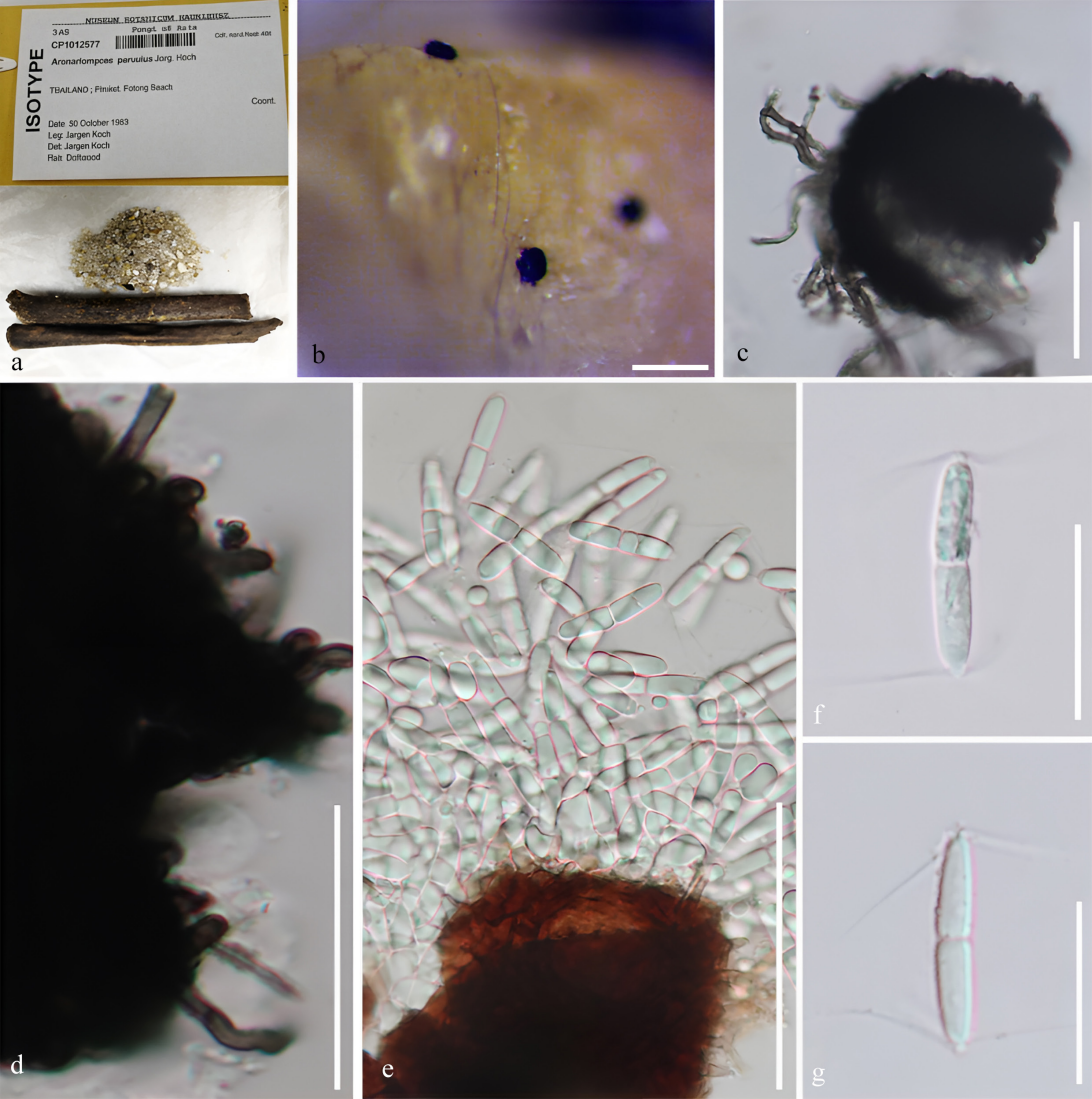
*Arenariomyces parvulus* (CP1012577, isotype). **a**. Herbarium material; **b**. Ascomata on sand particle; **c**. Outer appearance of ascoma; **d**. Short brown hyphae around ascoma; **e**. Squash mount of ascoma (microslide); **f, g**. Ascospores. Scale bars: 200 µm (**b**); 20 µm (**c–g**).

**Figure 15.**
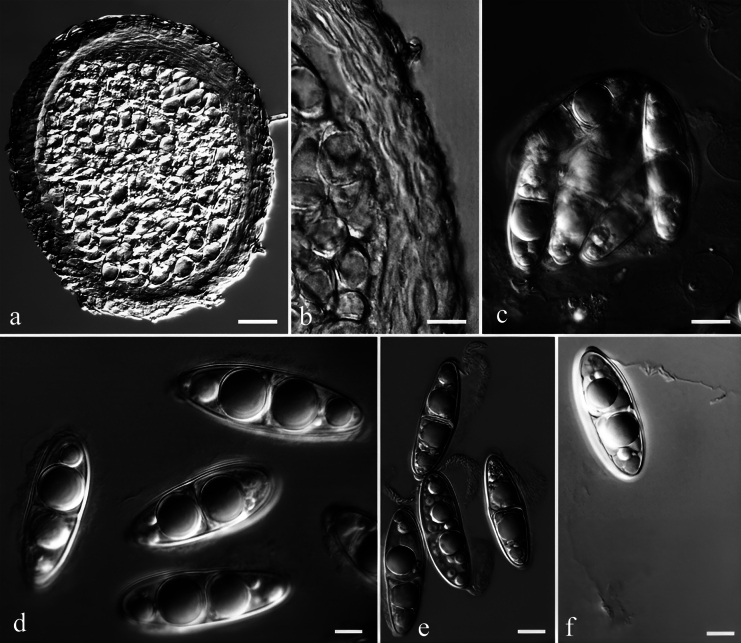
*Ascoglobospora marina*. **a**. Vertical section of ascoma; **b**. Magnified part of the peridium; **c**. Ascus and catenophyses; **d–f**. Variously shaped ascospores with various stages of appendages uncoiling. Scale bars: 25 μm (**a**); 5 μm (**c–f**).

##### Culture characteristics.

On PDA cultures light brown with tufts of aerial mycelium, reaching a 20–25 mm radius after one month at 25 °C, sporulation was not observed.

##### Material examined.

Japan, • Yokohama, Umikaze Park, 35°16'36"N, 139°41'02"E, on decaying driftwood, 12 October 2007, M.A. Abdel-Wahab, CBS-H-23855 (holotype), ex-type living culture, NBRC 105278. GenBank numbers: CBS-H-23855: ITS = OP150939, 28S rDNA = OP151088.

##### Notes.

*Ascoglobospora marina* is saprobic on wood and characterized by globose, thick-walled, hyaline to light brown ascomata, with catenophyses, asci subglobose, lacking apical apparatus or retraction of plasmalemma, ascospores with long unfurling bipolar appendages with prominent guttules. *Ascoglobospora marina* belongs to a group of ascomycetes with hamate appendages that uncoil to form long filamentous threads that aid in the entrapment and attachment to substrates in aquatic environments (Jones 1994, 1995). These include genera such as *Aniptodera*, *Ascosacculus*, *Halosarpheia*, *Magnisphaera*, *Natantispora*, *Praelongicaulis*, *Panorbis*, *Tirispora*, *Trichomaris*, and *Saagaromyces* (Kohlmeyer and Kohlmeyer 1979; Campbell et al. 2003; Sakayaroj et al. 2011; Jones et al. 2019), but phylogenetically distinct and generally disparate within the Halosphaeriaceae. Clearly this type of ascospore appendages plays a significant role in both freshwater and marine habitats.

### *Ascosacculus* J. Campb., J.L. Anderson & Shearer

Campbell et al. (2003) established the genus *Ascosacculus* for two *Halosarpheia* species: *H. aquaticus* and *H. heteroguttulatus*. Both of these taxa differ from the type species of *Halosarpheia* (*H. fibrosa*) in having globose, membranous ascomata; saccate, early deliquescent asci; tapering, fusiform to cylindrical, polyguttulate ascospores and occurrence in freshwater habitats. Phylogenetic analyses of the ribosomal genes placed *Ascosacculus* species in a well-supported clade distant from the *Halosarpheiasensu stricto* clade (Campbell et al. 2003; Pang et al. 2003b). Luo et al. (2019) described *Ascosacculus fusiformis* from decaying submerged wood in freshwater stream in Thailand.

#### Ascosacculus

Taxon classificationFungiMicroascalesHalosphaeriaceae

J. Campbell, J.L. Anderson & Shearer, Mycologia 95 (3): 545 (2003)

A26E1BCA-190F-5517-A869-9F2A072B79A5

Index Fungorum: IF28705

##### Description.

Saprobic on submerged wood in freshwater habitats. **Sexual morph. *Ascomata*** small, globose to subglobose, membranous, brown, ostiolate with long, narrow, thin-walled, hyaline to pale brown periphysate necks. ***Peridium*** forming *textura angularis*. ***Asci*** thin-walled, deliquesce early or persistent, saccate to cylindrical, with or without apical apparatus. ***Ascospores*** fusiform to cylindrical, hyaline, one to 3-septate filled with many small guttules and having a hamate appendage at each apex that unfurls to form long, thread-like, sticky appendages or with a thin mucilaginous sheath (*A. fusiformis*). **Asexual morph**. Undetermined.

##### Type species.

*Ascosacculus aquaticus* (K.D. Hyde) J. Campb., J.L. Anderson & Shearer, Mycologia 95 (3): 545 (2003).

##### Notes.

*Ascosacculus* species were recorded from decaying submerged wood in tropical freshwater streams (Hyde et al. 1999b; Campbell et al. 2003; Pang et al. 2003b; Luo et al. 2019).

##### Molecular evaluation.

Four *Ascosacculus* species grouped in the Halosphaeriaceae in highly supported clade (Fig. 4) adjacent to *Thalespora*, *Okeanomyces* and *Cucurbitinus*. Divergence-time analysis places the split across the Late Cretaceous to Paleogene interval (26 MYA, 95% CI 7.5–92.6 MYA), consistent with a long independent history (Fig. 5).

#### Ascosacculus aquaticus

Taxon classificationFungiMicroascalesHalosphaeriaceae

(K.D. Hyde) J. Campb., J.L. Anderson & Shearer, Mycologia 95 (3): 545 (2003)

9B3A190A-6CC3-59A2-A789-055452A37E56

Index Fungorum: IF458000

Halosarpheia aquatica K.D. Hyde, Austral. Syst. Bot. 5: 407 (1992). Basionym.

##### Description.

Saprobic on submerged wood in freshwater habitats. **Sexual morph. *Ascomata*** 180–205 µm high, 140–164 µm diam., immersed or semi-immersed, subglobose or pyriform, light brown or brown, membranous, ostiolate, beaked, solitary or gregarious. ***Necks*** central, long or short, up to 320 µm long, 30–42 µm diam., hyaline and with periphyses. ***Peridium*** up to 14 µm thick, composed of 2–3 layers of brown polygonal thin-walled cells, in surface view *textura angularis*. *Catenophyses* present. ***Asci*** 56 × 30 µm, 8-spored, saccate, thin-walled, with no apical thickening or pore, deliquescing early. ***Ascospores*** 33.5–64 × 7–10 µm, fusiform or cylindrical, hyaline, 1-septate, thin-walled, highly guttulate, with hamate appendages at each end. The appendages uncoil in water to form long filamentous threads. **Asexual morph**. Undetermined.

##### Material examined.

Australia, • North Queensland, Clohesy River, on submerged wood, August 1990, K. D. Hyde (BRIP 19331, holotype).

##### Notes.

The four *Ascosacculus* species share the following morphological characters: immersed, solitary, dark-brown ascomata with long neck, thin-walled peridium forming *textura angularis* and hyaline, fusiform, guttulate ascospores (Hyde 1992c; Luo et al. 2019). The four freshwater species that vary in their morphological characters from thin-walled, saccate, early deliquescing asci without apical apparatus and apical hamate ascospore appendages that uncoil to long filaments in *A. aquaticus* and *A. heteroguttulatus* to cylindrical, persistent asci with apical ring and 3-septate ascospores surrounded by a thin mucilaginous sheath (Hyde 1992c; Hyde et al. 1999b; Campbell et al. 2003; Pang et al. 2003b; Luo et al. 2019).

Phylogenetically, *Ascosacculus* species clustered together in a separate clade from other genera of Halosphaeriaceae with good bootstrap support (Campbell et al. 2003; Pang and Jones 2004; Luo et al. 2019).

##### Distribution.

Australia, Costa Rica, Thailand.

### *Bathyascus* Kohlm.

Kohlmeyer (1977) introduced the new genus *Bathyascus* with *B. vermisporus* as the type species collected on a submerged wood panel of *Pinus* in deep-sea water in the Pacific Ocean. Further species were introduced by Kohlmeyer (1980), *B. avicenniae* and *B. tropicalis*, on bark of pneumatophores of *Avicennia germinans* and submerged wood, respectively, in the Virgin Islands. Hyde and Jones (1987) described *B. grandisporus* on dead wood of *Rhizophora mucronata* in the Seychelles and Ravikumar and Vittal (1991) discovered *B. mangrovei* on dead *Rhizophora apiculata* wood in Tamil Nadu, India.

#### Bathyascus

Taxon classificationFungiMicroascalesHalosphaeriaceae

Kohlm., Rev. Mycol. (Paris) 41 (2): 190 (1977)

FFBA2D4F-39A6-50AA-BAE5-435509EABB51

Index Fungorum: IF519

##### Description.

Saprobic in marine habitats, mainly on mangrove wood, deep sea. **Sexual morph. *Ascomata*** subglobose or ellipsoidal, immersed, ostiolate, papillate, coriaceous, dark brown, solitary, neck lacking periphyses. ***Catenophyses*** deliquescing. ***Asci*** fusiform to clavate, unitunicate, thin-walled, early deliquescing. ***Ascospores*** filiform, 0–1-septate, hyaline and lacking appendages. **Asexual morph**. Undetermined.

##### Type species.

*Bathyascus vermisporus* Kohlm., Rev. Mycol. (Paris) 41 (2): 190 (1977).

##### Notes.

The genus is characterized as having ascospores that are filiform, straight or curved and without septa or apical chambers. Currently, five species are accepted primarily from tropical locations (Kohlmeyer 1980; Hyde and Jones 1987; Ravikumar and Vittal 1991). The species are saprobic, on intertidal wood and also those in the deep-sea.

*Bathyascus* is most similar to the genus *Thalespora*, as both genera have spathulate, hyaline, unicellular or 1-septate ascospores, however, *Thalespora* possesses apical ascospore appendages. *Bathyascus tropicalis* is the only member of the genus that possesses septate ascospores and ascomata with a thin-walled peridium (6–8 µm thick), and these two features are shared with *Thalespora appendiculata*. *Thalespora appendiculata* differs in possessing apical polar appendages with an asexual morph and its inclusion in the Halosphaeriaceae is supported by molecular data (Jones et al. 2006).

*Bathyascus grandisporus* ascospores are similar to those of *Halonectria* and *Pseudohalonectria* but differ by ascus and spore measurements and assignment to the families Hypocreaceae, and Amphisphaeriaceae, respectively.

##### Molecular evaluation.

Molecular data is not available for any of the *Bathyascus* species. All five species need to be recollected, isolated and sequenced to resolve their taxonomic position to confirm their inclusion in the Halosphaeriaceae, and whether *B. tropicalis* would be better assigned to the genus *Thalespora* (Jones et al. 2006). Numerous collections have been made of *Bathyascus* species, and attempts at their isolation have been made but ascospores did not germinate.

#### Bathyascus vermisporus

Taxon classificationFungiMicroascalesHalosphaeriaceae

Kohlm., Rev. Mycol. (Paris) 41 (2): 191 (1977)

7FAB6C22-CABB-5DA9-938E-CE83C45DA704

Index Fungorum: IF309467

Fig. 16

##### Description.

Saprobic on intertidal wood (deep sea), generally mangrove wood. **Sexual morph. *Ascomata*** 150–260 µm high (including necks), 215–480 µm in diam., solitary, subglobose or ellipsoidal, partly or completely immersed, ostiolate, papillate, coriaceous, dark brown above, light brown to almost hyaline at the base. ***Peridium*** 40–45 µm thick around the ostiole, 20–30 um at the base and sides, composed of 5 or 6 layers of thin-walled, polygonal, elongated cells with large lumina, forming a *textura angularis*, merging into the pseudoparenchyma of the centrum. ***Necks*** 60–380 µm long, 60–70 µm in diam., cylindrical or obtusely conical, dark brown; ostiolar canal about 15 µm in diam., periphyses absent; walls of some necks extend into lumina of wood vessels and, thus, appear warty. ***Catenophyses*** absent, the center of immature ascomata filled with hyaline, thin-walled pseudoparenchymatous cells that become compressed by the developing asci. ***Asci*** 70–80 × 12–14 µm, 8-spored, fusiform to clavate, unitunicate, thin-walled, early deliquescing. ***Ascospores*** 50–72 × 4–5.5 µm, filiform, or rarely elongate fusoid, straight or curved, attenuate, thick-walled, non-septate, hyaline, without appendages. **Asexual morph**. Undetermined. (Description based on Kohlmeyer 1977; Borse et al. 2012).

**Figure 16.**
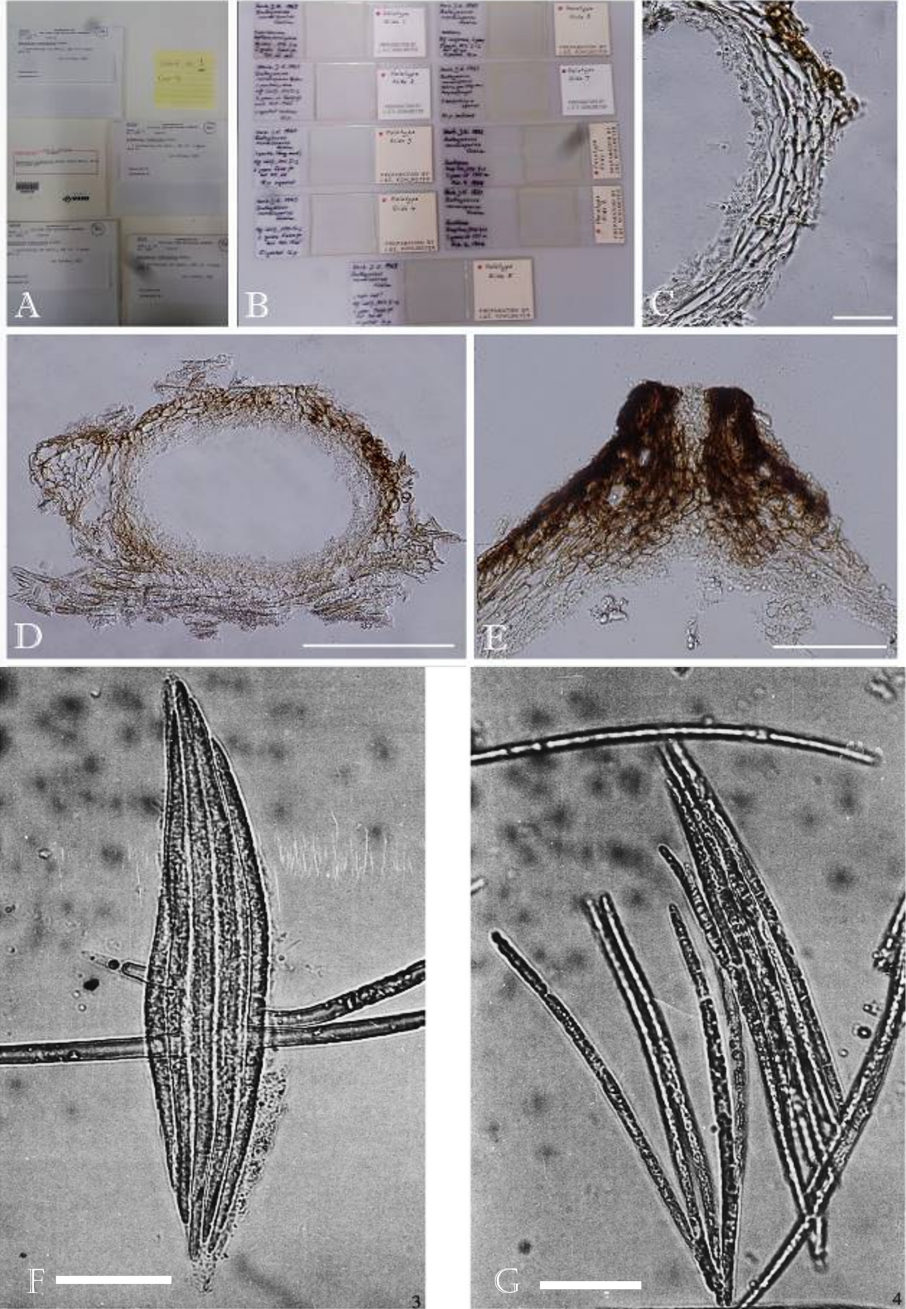
*Bathyascus vermisporus* (NY 966736, holotype; NY 966737, paratype). **a, b**. Herbarium material; **c**. Peridium (NY 966737); **d**. Section through ascomata (NY 966736); **e**. Ostiolate neck (NY 966736); **f**. Ascus; **g**. Ascospores. Scale bars: 10 μm (**c**); 20 μm (**d**); 10 μm (**e–g**).

##### Notes.

There are no sequences available for the type species of this genus.

##### Distribution.

India, USA. Both on wood and in deep-sea sediments.

### *Carbosphaerella* I. Schmidt

Schmidt (1969a) introduced the genus *Carbosphaerella* with *C. pleosporoides* as the type species. A second species *Carbosphaerella leptosphaerioides* was also described by Schmidt (1969b), a species that appears to be more commonly collected. Even so, very few collections of *Carbosphaerella* species have been reported (Kohlmeyer 1971; Koch 1974; Schmidt 1974b; Jones and Moss 1978; Rees et al. 1979; Johnson et al. 1984).

#### Carbosphaerella

Taxon classificationFungiMicroascalesHalosphaeriaceae

I. Schmidt, Feddes Repert. 80 (2–3): 108 (1969)

7B5EFEAF-63A5-5EBA-86B1-5A2D512BA976

Index Fungorum: IF820

##### Description.

Saprobic on marine wood. **Sexual morph. *Ascomata*** solitary of gregarious, globose to subglobose, superficial, sometimes with a subiculum on sand grains, with or without ostioles, papillate or epapillate, eperiphysate, carbonaceous and black. ***Peridium*** composed of 2–3 layers of cells. ***Necks*** short when present, often directed downwards. ***Pseudoparenchyma*** none reported and lacking paraphyses and catenophyses. ***Asci*** 4–8-spored, ovoidal to obpyriform, short pedunculate, unitunicate, thin-walled and deliquescing early. ***Ascospores*** ovoidal to ellipsoidal, 3-many-septate, constricted at the septa, central cells dark brown, end cells small and hyaline, and appendaged. Ascospores enveloped by a gelatinous persistent, irregular and sheath-like appendage which becomes fibrillar. ***Appendages*** formed by fragmentation of an exosporium which is composed of layers of electron-dense fibres wrapped around the spore. An inner layer of the exosporium fibres remains associated with the epispore. The spore wall is composed of an episporium and a mesosporium, the latter with an inner electron-transparent layer and an outer pigmented layer. Histochemistry, appendages composed of protein with neutral and acidic PAS positive carbohydrates. **Asexual morph**. Undetermined.

##### Type species.

*Carbosphaerella pleosporoides* I. Schmidt, Feddes Repert. 80 (2–3): 108 (1969).

##### Notes.

The species in *Carbosphaerella* are saprobic on wood, often associated with sand. The species are distinguished by ascospore color: central large cells dark brown, apical small cells hyaline or light brown and their septation: transverse septa only in *C. leptosphaerioides*, transverse and longitudinal septa in *C. pleosporoides*. The gelatinous sheath plays an important role in the adhesion of the ascospores to substrates (Johnson et al. 1984; Jones 1994).

Molecular data is available for *C. leptosphaerioides* (Jones et al. 2009), which is well placed in the Halosphaeriaceae, grouping with *Remispora pilleata* and *R. maritima* with strong statistical support (Jones et al. 2009, 2015; Maharachchikumbura et al. 2015).

##### Molecular evaluation.

Both species need to be recollected and sequenced for a wide range of gene loci.

#### Carbosphaerella pleosporoides

Taxon classificationFungiMicroascalesHalosphaeriaceae

I. Schmidt, Feddes Repert. Spec. Nov. Regni veg. 80: 108 (1969)

D1747624-B2BC-5EA1-A3B0-22535D548629

Index Fungorum: IF327523

##### Description.

Saprobic on driftwood, especially when in contact with sand. **Sexual morph. *Ascomata*** 100–190 µm, globose, superficial, sometimes seated on a subiculum on sand grains, with or without ostioles, papillate or epapillate. ***Necks*** when present, short, conical, near the basal subiculum and pointing downward. ***Paraphyses*** absent. ***Asci*** 26.5–40 × 23–39 µm, 4-spored, obpyriform to subglobose, short stipitate, unitunicate, thin-walled, without apical apparatus, early deliquescing. ***Ascospores*** 22–30 × 11–13–20 µm, muriform, constricted at the septum, central cells dark brown, apical cells hyaline to light brown, surrounded by a gelatinous, persistent, irregular sheath, appearing striated or dotted with parallel fibres embedded in a matrix; germination from the apical cells only. **Asexual morph**. Undetermined. (Description based on Kohlmeyer and Kohlmeyer 1979).

#### Carbosphaerella leptosphaerioides

Taxon classificationFungiMicroascalesHalosphaeriaceae

I. Schmidt, Natur Naturschutz Mecklenburg 7: 9 (1969)

77AB9D8A-26FC-5120-A357-533E8E235826

Index Fungorum: IF310397

Figs 2d, e, 17

##### Description.

Saprobic on driftwood, especially when in contact with sand, intertidal mangrove wood. **Sexual morph. *Ascomata*** 90–210 µm in diam., globose to subglobose, superficial, sometimes seated on a subiculum on sand grains, with or without ostioles, papillate or epapillate, carbonaceous, black, solitary or gregarious. ***Peridium*** 5–12 µm thick, composed of 2–3 layers of cells, subglobose at the outside, flattened toward the inside. ***Necks*** when present, short, conical, near the basal subiculum and pointing downward. ***Paraphyses*** absent. ***Asci*** 65–90 × 45–60 µm (excluding peduncle), 4–8-spored, ovoidal or obpyriform, short pedunculate, unitunicate, thin-walled, aphysoclastic, without apical apparatus, early deliquescing. ***Ascospores*** 27–42 × 16–24 µm (excluding sheath), ellipsoidal, 3-septate, not or slightly constricted at the septa, central large cells dark brown, apical small cells hyaline or light brown; septa with a central pore, ca. 1 µm in diam., except for the apical cells, surrounded by a gelatinous, persistent, irregular sheath, 4–12.5 µm thick, hyaline or faintly brown, appearing striated or dotted with parallel fibres embedded in a matrix; germination from the apical cells only. **Asexual morph**. Undetermined. (Description based on Kohlmeyer and Kohlmeyer 1979; Borse et al. 2012).

**Figure 17.**
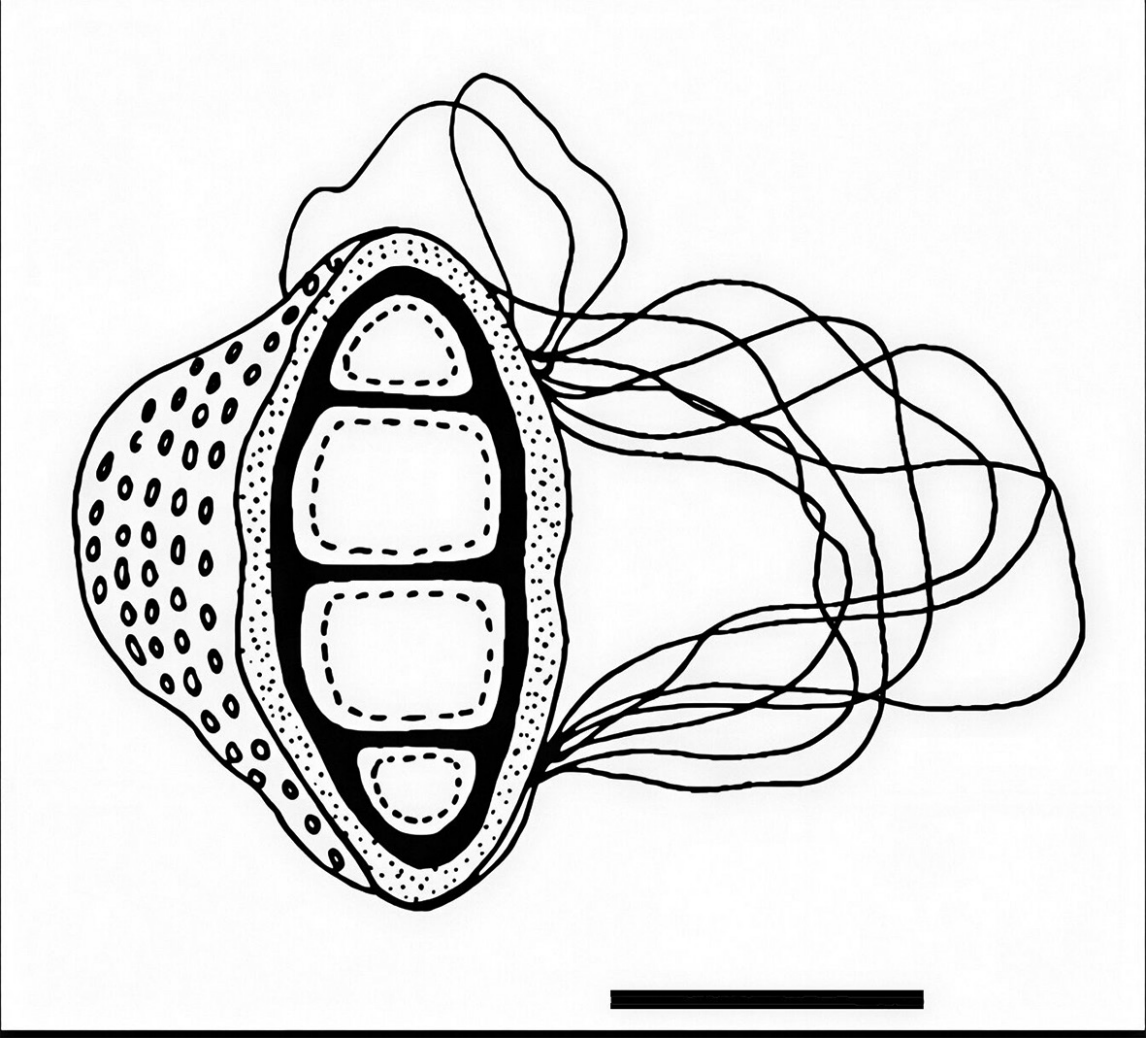
Ascospore of *Carbosphaerella leptosphaerioides* with net-like appendage. Scale bar: 20 mm.

##### Notes.

Ecologically, this species is found superficially on wood generally associated with sand (Koch and Jones 1983; Farrant et al. 1985; Rees and Jones 1985). Mostly found on buried wood in sand, it has also been recorded on mangrove woody litter (Shini et al. 2009–2010) and intertidal wood in India (Borse and Pawar 2001; Prasannarai and Sridhar 2001).

##### Distribution.

Bermuda, Canada, Denmark, Germany, Hawaii, India, Japan, Malaysia, New Zealand, Portugal, Taiwan, UK, USA.

### *Ceriosporopsis* Linder

This genus was introduced by Barghoorn and Linder (1944) with *Ceriosporopsis halima* as the type species. Subsequently, further species have been referred to the genus: *C. cambrensis* (Wilson 1954), *C. barbata* (Höhnk 1955) (= *C. halima*), *C. capillacea* (Kohlmeyer 1981), *C. caduca* (Jones and Zainal 1988), and *C. minuta* (Abdel-Wahab et al. 2017). Kohlmeyer (1972) listed six *Ceriosporopsis* species, while https://www.marinefungi.org lists seven with *Bovicornua intricata* transferred to *Ceriosporopsis* by Sakayaroj et al. (2011). Others have been referred to other genera as the result of ultrastructural and phylogenetic studies: *C. calyptrata* and *C. longissima* to *Marinospora* (Johnson et al. 1984); *C. circumvestita* to *Lautisporopsis* (Yusoff et al. 1994a); *C. sundica* to *Limacospora* (Jones et al. 1995) and *C. tubulifera* to *Toriella* (Sakayaroj et al. 2011).

#### Ceriosporopsis

Taxon classificationFungiMicroascalesHalosphaeriaceae

Linder, Farlowia 1 (3): 408 (1944)

3E9D8757-B771-55D6-999F-75F7653B325B

Index Fungorum: IF915

##### Description.

A group of obligate marine lignicolous fungi, saprobic on wood. **Sexual morph. *Ascomata*** solitary or gregarious, subglobose to cylindrical, immersed or becoming exposed, ostiolate, papillate, coriaceous or subcarbonaceous, light brown to black. ***Pseudoparenchyma*** thin-walled cells filling venter of young ascocarps; deliquescing, or central core developing into catenophyses. ***Asci*** 8-spored, clavate, pedunculate, unitunicate, thin-walled, early deliquescing. ***Ascospores*** ellipsoidal, 1-septate, hyaline, surrounded by an exosporic sheath that forms apical collar which ruptures to release a mucilaginous appendage that stains metachromatically in 0.1% toluidine blue (Adapted from Kohlmeyer and Kohlmeyer 1979). **Asexual morph**. Undetermined.

##### Type species.

*Ceriosporopsis halima* Linder, Farlowia 1(3): 409 (1944).

##### Notes.

*Ceriosporopsis* is a genus of obligate lignicolous marine fungi, often found on decayed wood. The type species (*C. halima*) is a cosmopolitan fungus, occurring in both tropical and temperate waters (Raghukumar 2019).

*Ceriosporopsis* is morphologically close to *Marinospora* and *Toriella*: *Marinospora* differs from *Ceriosporopsis* in the following aspects, ascocarps are carbonaceous to subcarbonaceous, ascocarp wall is composed of up to 30 layers, asci are pedunculate and often apiculate, ascospores have distinct primary polar and equatorial appendages with cup-like structures at their apices formed by fragmentation (or dissolution) of an enveloping exosporic membrane (Johnson et al. 1984). *Toriella* differs from *Ceriosporopsis* in the exosporium which folds to form an annulus-like equatorial appendage and the polar appendage is formed inside an end chamber that consists of two electron-dense layers (Johnson et al. 1987).

##### Molecular evaluation.

Only *Ceriosporopsis halima* (9 isolates) and *Ceriosporopsis intricata* (3 isolates) (still registered as *Bovicornua intricata* in Index Fungorum) are supported by molecular data. Strains of *Ceriosporopsis* formed a highly supported clade in the Halosphaeriaceae (Fig. 4), comprising two highly supported sub-clades corresponding to the two species. *Ceriosporopsis* diverged from allied genera in the early Jurassic (mean 196 MYA; 95% CI 73.1–528.1 MYA), indicating a very old lineage within the family (Fig. 5).

#### Ceriosporopsis halima

Taxon classificationFungiMicroascalesHalosphaeriaceae

Linder, Farlowia 1(3): 409 (1944)

474E2432-5405-5088-A644-A2644450E165

Index Fungorum: IF285108

##### Description.

Saprobic on wood and other cellulosic substrates. **Sexual morph. *Ascomata*** 80–481 µm high, 130–503 µm in diam. or long, subglobose, ellipsoidal or cylindrical, immersed or superficial, ostiolate, papillate, coriaceous, dark brown to black, reddish-brown when empty, solitary or gregarious. ***Peridium*** 8–26 µm thick, composed of three to four (to ten) layers of thin-walled, elongate cells with large lumina, merging into the pseudoparenchyma of the venter. ***Necks*** up to 706 µm long, 16–30(-56) µm in diam., centric or eccentric, cylindrical; ostiolar canal at first filled with a small-celled pseudoparenchyma. ***Pseudoparenchyma*** of thin-walled, polygonal cells filling venter of young ascocarps. ***Asci*** 56–89 × 14–22 µm, 8-spored, ellipsoidal, subclavate or subfusiform, short pedunculate, unitunicate, thin-walled, aphysoclastic, deliquescing before ascospore maturity, without apical apparatuses, developing at the base of the ascocarp venter. ***Ascospores*** 18–27(–35) × 6–12 µm (excluding appendages), ellipsoidal to fusiform-ellipsoidal, 1-septate, slightly or strongly constricted at the septum, hyaline, surrounded by a gelatinous, exosporic sheath that is pierced at each apex by an outward growing appendage; appendages 5–8 µm in diam., of variable length, terminal, simple, subcylindrical, tapering, finally becoming viscous and filamentous. ***Chlamydospores*** from in axenic cultures 6–17 µm in diam., reddish-brown, catenulate; chains up to 90 µm long, up to 13-celled, terminal, simple or rarely ramose, curved, frequently 1/4 or 3/4 times coiled; single cells globose, ellipsoidal or subcylindrical, sometimes increasing in diameter from base to apex. **Asexual morph**. Undetermined (Adapted from Kohlmeyer and Kohlmeyer 1979).

##### Notes.

*Ceriosporopsis halima* is widely collected on intertidal and driftwood, bark, rope, test panels and a range of timbers. *Ceriosporopsis halima* is an early colonizer of wood test panels submerged in the sea, sporulating on the wood after 6–18 weeks of immersion (Jones 1963).

Within *Ceriosporopsis*, the most divergent species is *C. caduca*, the only one with equatorial appendages; *C. capillacea* has the longest polar appendages, other than these two, other species are only discernible from each other by ascospore measurements.

##### Distribution.

Argentina, Australia, Canada, Denmark, France, Germany, Ghana, Iceland, India, Italy, Japan, Norway, Peru, Portugal, Spain, Taiwan, UK (England, Wales, Scotland), USA (California, Hawaii, Washington).

### *Chadefaudia* Feldm.-Maz.

Jean Feldmann (1941) introduced the novel species *Macrophoma gymnogongri* parasitic on the red alga *Gymnogongrus norvegicus* collected in Algeria. It produced pycnidia immersed in the alga with hyaline unicellular conidia. Cribb and Herbert (1954) collected this species on a red alga *Ptilonia australasica* on the north coast of Tasmania, Australia. Geneviève Feldmann (1957) introduced the genus *Chadefaudia* with *Ch. marina* as the type species on the red alga *Palmaria palmata* (=*Rhodymenia palmata*) collected in Roscoff, France and referred to the Ophiostomataceae. Subsequently, Cribb and Cribb (1960a) referred *Macrophoma gymnogongri* to the genus *Mycophycophila* with collections on a range of red algae: *Laurencia* spp., *Gigartina intermedia* and *Galaxaura* sp., in New South Wales, Australia (Cribb and Cribb 1960b). Müller and von Arx (1973) proposed that *Mycophycophila* should be considered as a synonym of *Chadefaudia* and this has been universally accepted.

#### Chadefaudia

Taxon classificationFungiMicroascalesHalosphaeriaceae

Feldm.-Maz., Rev. Gén. Bot. 64: 150 (1957)

749C24D5-708B-5799-9939-42950DC38989

Index Fungorum: IF927

##### Description.

Parasitic, or epiphytic on red algae or saprophytic or symbiotic on calcified microalgae. **Sexual morph. *Ascomata*** solitary or gregarious, subglobose, superficial or immersed, ostiolate, papillate or epapillate, coriaceous to carbonaceous, dark colored. ***Paraphyses*** absent. ***Asci*** 8-spored, subglobose to clavate, unitunicate, thin-walled, early deliquescing. ***Ascospores*** ellipsoidal, 1-septate, hyaline, with an apical gelatinous sheath, cap-like appendage. **Asexual morph**. Coelomycetous.

##### Type species.

*Chadefaudia marina* Feldm.-Maz., Rev. Gén. Bot. 64: 150 (1957).

##### Notes.

*Chadefaudia* species are parasitic, or epiphytic on red algae or saprophytic or symbiotic on calcified microalgae and worldwide in distribution (Kohlmeyer and Kohlmeyer 1979).

A genus with a confusing history and comprising six species: *Chadefaudia balliae*, *Ch. corallinarum*, *Ch. gymnogongri*, *Ch. marina*, *Ch. polyporolithi* and *Ch. schizymeniae*, all on different marine hosts, but mostly on red algae. With exception of *Ch. gymnogongri* and *Ch. marina*, most are poorly known and all require recollection and sequencing to confirm the placement of *Chadefaudia* in the Halosphaeriaceae.

##### Molecular evaluation.

As no molecular data is available, all species need recollection, isolation and sequencing.

#### Chadefaudia marina

Taxon classificationFungiMicroascalesHalosphaeriaceae

Feldm.-Maz., Rev. Gén. Bot. 64: 150 (1957)

F8E4DD17-B6DA-5A9C-AFE6-CB949E92EA41

Index Fungorum: IF294674

##### Description.

Parasitic on red algae. **Sexual morph. *Ascomata*** 90–180 µm high, 90–200 µm diam., subglobose, immersed, ostiolate, papillate or epapillate, coriaceous, dark brown. ***Peridium*** 10–39 µm thick, merging with host tissue. ***Pseudoparenchyma*** cells thin-walled. ***Asci*** 23–34 × 12–16 µm, 8-spored, ellipsoidal to broadly clavate with short pedicel, unitunicate, thin-walled, early deliquescing, lacking apical apparatus. ***Ascospores*** 12.5–16.5 × 4–5.5 µm, ellipsoidal, unicellular, hyaline, with appendages at both ends. ***Appendages*** 2–3 µm high, 4 µm diam., gelatinous and terminally attached. **Asexual morph**. Undetermined.

##### Notes.

*Chadefaudia marina* is only known from a collection made in France by Feldmann (1957) and collected on the red alga *Palmaria palmata* (=*Rhodymenia palmata*) at Roscoff, France. Feldmann describes and illustrates (line drawings) in detail the morphology and development of the ascoma in *Ch. marina*. The most recently described *Chadefaudia* species is *Ch. schizymeniae* by Stegenga and Kemperman (1984) from the red alga *Schizymenia obovata* collected in South Africa.

### *Cirrenalia* Meyers & R.T. Moore

Meyers and Moore (1960) established the genus *Cirrenalia* for *Helicoma macrocephala* that has different conidial morphology to other *Helicoma* species. The original concept of the genus was for marine species with helicoid conidial cells that increase in size and pigmentation from basal to apical cells. Seven *Cirrenalia* species were described from marine habitats occurring primarily on woody tissue and *Juncus roemerianus* (Kohlmeyer 1958; Schmidt 1969a, 1985; Raghukumar et al. 1988; Kohlmeyer et al. 1997). However, the genus concept was broadened by the description of 13 species from terrestrial habitats with a large number of conidial cells that are more or less similar in pigmentation and size (Sutton 1973; Rao and Reddy 1978; Matsushima 1980, 1996; Sugiyama 1981; Mel’nik 1988; Somrithipol et al. 2002; Zhao and Liu 2005; Jiang and Zhang 2007; Zhang et al. 2014; Hernández-Restrepo et al. 2017; Spribille et al. 2020). Phylogenetic analyses of the 28S rDNA placed *Cirrenalia macrocephala* within the Halosphaeriaceae (Tsui and Berbee 2006). Abdel-Wahab et al. (2010) studied the molecular phylogeny of five *Cirrenalia* species based on 18S and 28S rDNA loci, demonstrating the genus was polyphyletic and affiliated with three different orders: Lulworthiales, Pleosporales and Torpedosporales.

#### Cirrenalia

Taxon classificationFungiMicroascalesHalosphaeriaceae

Meyers & R.T. Moore, Amer. J. Bot. 47 (5): 346 (1960)

6B579F20-BA76-52A3-90AD-F8F954016BF4

Index Fungorum: IF7668

##### Description.

Saprobic on plant debris and soil in marine, freshwater and soil habitats. **Sexual morph**. Undetermined. **Asexual morph. *Conidiophores*** present or obsolete, cylindrical, septate or nonseptate, acrogenous or laterally on the hyphae, hyaline or light brown. ***Conidiogenous cells*** monoblastic, integrated, terminal, determinate. ***Conidia*** acrogenous, solitary, helicoid, septate, constricted or not constricted at the septa, brownish, conidial cells increase in diameter and pigmentation from base to apex.

##### Type species.

*Cirrenalia macrocephala* (Kohlm.) Meyers & R.T. Moore, Amer. J. Bot. 47 (5): 347 (1960).

##### Notes.

*Cirrenalia* species were described and recorded from marine, freshwater and terrestrial habitats on plant remains and soil.

Molecular data (Fig. 4) places *Cirrenalia macrocephala* in the new genus *Remisporiopsis* with high support and therefore it has been excluded from *Cirrenalia*. However, other *Cirrenalia* species have been described including two marine species (Kohlmeyer 1958, 1968; Raghukumar et al. 1988) and 10 terrestrial species (Sutton 1973; Rao and Reddy 1978; Matsushima 1980, 1996; Sugiyama 1981; Mel’nik 1988; Somrithipol et al. 2002; Zhao and Liu 2005; Jiang and Zhang 2007; Zhang et al. 2014; Hernández-Restrepo et al. 2017’ Spribille et al. 2020). These are retained in *Cirrenalia* until they have been recollected isolated and sequenced.

Other marine *Cirrenalia* species have been referred to other genera: *Halazoon* for *C. fusca*, *Hydea* for *C. pygmea*, and *Matsusporium* for *C. tropicalis* in the Lulworthiales, *Moheitospora* for *C. adarca* in the Torpedosporales and *Hiogispora* for *C. japonica* in the Pleosporales (Abdel-Wahab et al. 2010; Jones et al. 2019).

##### Molecular evaluation.

Molecular data is not available for 12 *Cirrenalia* species and further collections for those species are required to confirm their phylogenetic placements.

### *Corallicola* Volkm. – Kohlm. & Kohlm.

The genus *Corallicola* was introduced by Volkmann-Kohlmeyer and Kohlmeyer (1992) with *Corallicola nana* as the type species, collected on dead coral slab in Belize, Central America. *Corallicola nana* is a rare species as it was found in only “one sample from hundreds of collected dead coral slabs”. *Corallicola* differs from the genus *Corollospora* in lacking equatorial appendages but shows similarities to *Arenariomyces*. It differs from *Arenariomyces* in lacking a bulbous base to the spores’ appendages (Jones et al. 1983a, 1986).

#### Corallicola

Taxon classificationFungiMicroascalesHalosphaeriaceae

Volkm. – Kohlm. & Kohlm., Mycotaxon 44 (2): 418 (1992)

BA233E6D-3FB6-5FB8-AFC3-2DB73F3C15D5

Index Fungorum: IF25426

##### Description.

Probably saprobic. **Sexual morph. *Ascomata*** are singular or gregarious, subglobose, superficial, ostiolate, short papillate or epipapillate, subiculate, coriaceous, brown. ***Peridium*** is thin-walled, forming a *textura angularis*. The centrum of immature ascomata is filled with pseudoparenchymatous hyaline deliquescent cells. ***Asci*** thin-walled, deliquescing early in development. ***Ascospores*** are ellipsoidal, 1-septate, hyaline, appendiculate; at both apices, with several terminal appendages, gibbous at the base, flattened in the middle and gradually tapering towards the tip (Adapted from Volkman-Kohlmeyer and Kohlmeyer 1992). **Asexual morph**. Undetermined.

##### Type species.

*Corallicola nana* Volkm.-Kohlm. & Kohlm., Mycotaxon 44 (2): 418 (1992).

##### Notes.

*Corallicola* is characterized by its 1-septate, ellipsoidal ascospores with 5–7 appendages, making it similar to members of the genus *Arenariomyces* (Höhnk 1954). They differ in that the pseudoparenchyma of the centrum has pit-like connections in *A. trifurcatus* but lacking in *C. nana*. Furthermore, the 5–7 terminal appendages of *C. nana* are swollen at the base, flat and wide in the middle and tapering to thin rounded tips, while in *A. trifurcatus* its 3 subterminal appendages have bulbous bases at the end (Jones et al. 1983a). Ecologically they differ; *A. trifurcatus* is an intertidal, arenicolous species associated with wood, while *C. nana* grows offshore on dead coral rocks (Volkman-Kohlmeyer and Kohlmeyer 1992).

##### Molecular evaluation.

As no molecular data is available, it requires recollection, isolation and sequencing.

#### Corallicola nana

Taxon classificationFungiMicroascalesHalosphaeriaceae

Volkm.-Kohlm. & Kohlm., Mycotaxon 44 (2): 418 (1992)

CE46ABCF-4DCC-531B-861A-9EFA36DC791C

Index Fungorum: IF358979

##### Description.

Saprobic on dead coral. **Sexual morph. *Ascomata*** are solitary or gregarious 80–95 µm in diam., subglobose, superficial, ostiolate, short papillate or epipapillate, subiculate, coriaceous, dark brown, sometimes covered by short brown hyphae. ***Peridium*** is 5–7 µm thick, composed of one or two layers of polygonal cells, forming a *textura angularis*, dark brown; at the base attached to the substrate with a thin subiculum that is more or less hyphoid (*textura intricata*) or dense (*textura angularis*). ***Pseudoparenchyma*** of thin-walled polygonal cells filling the centrum of young ascomata, deliquescing at ascospore maturity; no indication of pit-connections in the walls. ***Asci*** thin-walled, deliquescing before ascospore maturation. ***Ascospores*** range from 21–26.5 × 7–8.5 µm, ellipsoidal, 1-septate, slightly constricted at the septum, hyaline, appendaged at each end with 5–7 terminal appendages, 15–18 µm long, round and swollen at the base, 1.4–1.7 µm wide and flat in the middle, and tapering to the thin (0.5) µm round tip (Adapted from Volkmann-Kohlmeyer and Kohlmeyer 1992). **Asexual morph**. Undetermined.

##### Notes.

*Corallicola nana* is an obligate marine ascomycete, probably saprophytic as it does not damage living corals, simply growing attached to dead coral (Volkmann-Kohlmeyer and Kohlmeyer 1992).

### *Corollospora* Werderm.

Werdermann (1922) introduced *Corollospora*, with one species, as a coelomycete, while Barghoorn and Linder (1944), unaware of this earlier publication, erected the genus *Peritrichospora* with *P. integra* as the type species and a second species *P. lacera*. Subsequently Kohlmeyer (1960) described two new species and assigned *Arenariomyces trifurcatus* to *Peritrichospora* (Kohlmeyer 1961). Kohlmeyer (1962b) placed *Peritrichospora* in synonymy with *Corollospora*. Kohlmeyer (1968), Kohlmeyer et al. (1967), Schmidt (1969b) and Nakagiri and Tubaki (1982) described further new species of *Corollospora*, bringing the total to nine.

Schmidt (1974a) was the first to suggest (without formal description) that the genus *Corollospora* should be subdivided into two sections: the ‘coronate’, to which she assigned the species *C. maritima*, *C. intermedia*, *C. lacera* and *C. pulchella*; and the ‘cristatiae’ with the species *C. comata* and *C. cristata*. The species *C. trifurcata* and *C. tubulata* were not assigned to either section but she suggested them to be placed in other genera, “possibly *Carbosphaerella*?” Jones and Moss (1980) showed that on the basis of spore ontogeny, four assemblages could be discerned in *Corollospora*. They proposed the following: the species *C. maritima*, *C. intermedia*, *C. lacera* and *C. pulchella* be retained; a new genus be erected for *C. tubulata*, *Kohlmeyeriella*; a new genus be erected for *C. comata* and *C. cristata*, *Nereiospora*; that *C. trifurcata* be referred to *Arenariomyces*. These proposals were confirmed by Jones et al. (1983a) in a TEM study of *Corollospora* species. Subsequently, other species were described and the genus contains over 20 species (https://www.marinefungi.org; Index Fungorum).

A more radical treatment of the genus was proposed by Correia et al. (2023) based on phylogeny and p-distance estimation of predominantly 28S rDNA sequences available for the group, while early sequences were of poor quality. Correia et al. (2023) retained *C. maritima*, *C. quinqueseptata*, *C. mediterranea* and *C. intermedia* in *Corollospora*, while referred the remaining *Corollospora* species to ten new genera based on pairwise distances and phylogenetic analyses: *Ajigaurospora* (*A. pseudopulchella*), *Corollosporella* (*C. anglusa*, *C. ramulosa*), *Corollosporopsis* (*C. portsaidica*), *Garethelia* (*G. parvula*), *Honshuriella* (*H. fusca*), *Keraliethelia* (*K. pulchella*), *Nakagariella* (*N. filiformis*), *Paracorollospora* (*P. angusta*, *P. luteola*, *P. marina*), *Shirahamella* (*Sh. gracilis*), and *Tokurathelia* (*T. colossus*).

We have not adopted the splitting of *Corollospora* into new genera as we regard it a cohesive group of species with characteristic morphological and ecological features: ascospores generally have a primary polar spine, or thorn-like or reduced papilla formed by the mesosporium and episporium layers of the spore wall; secondary sheet-like equatorial and polar appendages formed by fragmentation of the exosporium (variously referred to as peritrichous or frill-like); most are arenicolous with ascomata attached to hard substrata by a subiculum; ostioles tucked in between the subiculum and base of the ascomata to minimize abrasion by sand grains; often mycelium binding sand grains into a thick biofilm (Rees et al. 1979; Jones et al. 1983a; Rees and Jones 1985; Velez et al. 2022). Nakagiri et al. (2025) decided to retain *Corollospora* as single genus for the time being as the proposed new genera were not “clearly defined by the phenotypic characteristics such as morphology, ecology, life cycles and that most were monotypic, so it must be reasonable to treat the differences between them as species level differences rather than generic”.

Hongsanan et al. (2025) introduced *Keraliethelia hydei* for an asexual morphic marine fungus from submerged decaying wood at Thalang district, Phuket Province, Thailand. Here we refer to *Corollospora hydei* as the genus *Keraliethelia* is not accepted. Currently, the genus contains 30 species (Table 1).

**Table 1.** Genera and species of the Halosphaeriaceae. *type species of each genus.

**Taxa**
**1. *Alisea*** J. Dupont & E.B.G. Jones
*A. longicolla J. Dupont & E.B.G. Jones
**2. *Amphitrite*** S. Tibell
*A. annulata S. Tibell
**3. *Aniptodera*** Shearer & M. Miller
*A. aquadulcis* (S.Y. Hsieh, H.S. Chang & E.B.G. Jones) J. Campb., J.L. Anderson & Shearer
*A. chesapeakensis Shearer & M.A. Mill.
*A. fusiformis* Shearer
*A. haispora* Vrijmoed, K.D. Hyde & E.B.G. Jones
*A. inflatiascigera* K.M. Tsui, K.D. Hyde & Hodgkiss
*A. intermedia* K.D. Hyde & Alias
*A. lignicola* K.D. Hyde
*A. mangrovei* K.D. Hyde
*A. margarition* Shearer
*A. mauritaniensis* K.D. Hyde, W.H. Ho & K.M. Tsui
*A. megaloascocarpa* Raja & Shearer
*A. megalospora* K.D. Hyde, W.H. Ho & K.M. Tsui
*A. nypae* K.D. Hyde
*A. palmicola* K.D. Hyde, W.H. Ho & K.M. Tsui
*A. triseptata* K.D. Hyde
**4. *Aniptosporopsis*** (K.D. Hyde) K.L. Pang
*A. lignatilis (K.D. Hyde) K.L. Pang, C.L. Lu, W.T. Ju & E.B.G. Jones
**5. *Anisostagma*** K.R.L. Petersen & Jørg. Koch
*A. rotundatum K.R.L. Petersen & Jørg. Koch
**6. *Antennospora*** Meyers
*A. quadricornuta (Cribb & J.W. Cribb) T.W. Johnson
**7. *Appendichordella*** R.G. Johnson, E.B.G. Jones & S.T. Moss
*A. amicta (Kohlm.) R.G. Johnson, E.B.G. Jones & S.T. Moss
**8. *Arenariomyces*** Höhnk
*A. majusculus* Kohlm. & Volkm.-Kohlm.
*A. parvulus* Jørg. Koch
*A. salinus* Meyers
*A. trifurcatus Höhnk
*A. triseptatus* Kohlm.
*A. truncatellus* Jørg. Koch
**9. *Ascoglobospora*** Abdel-Wahab
*A. marina Abdel-Wahab
**10. *Ascosacculus*** J. Campb., J.L. Anderson & Shearer
*A. aquaticus (K.D. Hyde) J. Campb., J.L. Anderson & Shearer
*A. fusiformis* Z.L. Luo, K.D. Hyde & H.Y. Su
*A. heteroguttulatus* (S.W. Wong, K.D. Hyde & E.B.G. Jones) J. Campb., J.L. Anderson & Shearer
*A. thailandicus* D.F. Bao, K.D. Hyde & Z.L. Luo
**11. *Bathyascus*** Kohlm.
*B. avicenniae* Kohlm.
*B. grandisporus* K.D. Hyde
*B. mangrovei* Ravik. & Vittal
*B. tropicalis* Kohlm.
*B. vermisporus Kohlm.
**12. *Carbosphaerella*** I. Schmidt
*C. pleosporoides I. Schmidt
*C. leptosphaerioides* I. Schmidt
**13. *Ceriosporopsis*** Linder
*C. cambrensis* Wilson
*C. capillacea* Kohlm.
*C. caduca* Jones and Zainal
*C. halima Linder
*C. intricata* (Jørg. Koch & E.B.G. Jones) Sakay., K.L. Pang & E.B.G. Jones
*C. minuta* Abdel-Wahab, Nagah. & E.B.G. Jones
14. ***Chadefaudia*** Feldm.-Maz.
*Ch. balliae* Kohlm.
*Ch. corallinarum* (P. Crouan & H. Crouan) E. Müll. & Arx
*Ch. gymnogongri* (Feldmann) Kohlm.
*Ch. marina Feldm.-Maz.
*Ch. polyporolithi* (Bonar) Kohlm.
*Ch. schizymeniae* Stegenga & Kemperman
**15. *Cirrenalia*** Meyers & R.T. Moore
*C. acericola* Melnik
*C. basiminuta* Raghuk. & Zainal
*C. caffra* Matsush.
*C. donnae* B. Sutton
*C. hunanensis* Y.L. Zhang & T.Y. Zhang
*C. indica* G.V. Rao & A.P. Reddy
*C. lichenicola* Pérez-Ort.
*C. lignincola* P.M. Kirk
*C. longipes* G.Z. Zhao & Xing Z. Liu
*C. pallescens* Y.L. Jiang & T.Y. Zhang
*C. palmicola* Matsush.
*C. pseudomacrocephala* Kohlm.
*C. rhodospora* Y.L. Jiang & T.Y. Zhang
**16. *Corallicola*** Volkm.-Kohlm. & Kohlm.
*C. nana Volkman-Kohlmeyer & Kohlmeyer
**17. *Corollospora* Werderm**.
*C. anglusa* Abdel-Wahab & Nagahama
*C. angusta* Nakagiri & Tokura
*C. armoricana* Kohlm. & Volkm.-Kohlm.
*C. baravispora* Steinke & E.B.G. Jones
*C. besarispora* Sundari
*C. borealis* S. Tibell
*C. californica* Kohlm. & Volkm.-Kohlm.
*C. cinnamomea* Jørg. Koch
*C. clavata* Nakagiri, K. Miyazaki & Shino
*C. colossa* Nakagiri & Tokura
*C. filiformis* Nakagiri
*C. fusca* Nakagiri & Tokura
*C. gracilis* Nakagiri & Tokura
*C. hydei* (Bagacy, Opina & M.S. Calabon) M.S. Calabon
*C. indica* Prasannarai, Ananda & K.R. Sridhar
*C. intermedia* I. Schmidt
*C. lacera* (Linder) Kohlm.
*C. luteola* Nakagiri & Tubaki
*C. marina* Haythorn & E.B.G. Jones
*C. maritima Werderm.
*C. mediterranea* A. Poli, E. Bovio, G.C. Varese & V. Prigione
*C. mesapotamica* Al-Saadon
*C. novo-fusca* Kohlm.
*C. parvula* Zuccaro, J.I. Mitch. & Nakagiri
*C. portsaidica* Abdel-Wahab & Nagahama
*C. prolifera* (Nakagiri) K. Miyazaki & Nakagiri
*C. pseudopulchella* Nakagiri & Tokura
*C. pulchella* Kohlm., I. Schmidt & Nair
*C. ramulosa* Meyers & Kohlm.
*C. quinqueseptata* Nakagiri
**18. *Cucullosporella*** K.D. Hyde & E.B.G. Jones
*C. mangrovei (K.D. Hyde & E.B.G. Jones) K.D. Hyde & E.B.G. Jones
**19. *Cucurbitinus*** L.L. Liu & Z.Y. Liu, in Liu, Liu, Yang, Chen & Liu
*C. constrictus (I. Schmidt) L.L. Liu & Z.Y. Liu, in Liu, Liu, Yang, Chen & Liu
*C. ibericus* (Hern.-Restr. & Gené) L.L. Liu & Z.Y. Liu, in Liu, Liu, Yang, Chen & Liu
**20. *Ebullia*** K.L. Pang
*E. octonae (Kohlm.) K.L. Pang
**21. *Fluviatispora*** K.D. Hyde
*F. boothii* Fryar & K.D. Hyde
*F. reticulata* K.D. Hyde
*F. tunicata K.D. Hyde
**22. *Gesasha*** Abdel-Wahab & Nagahama
*G. peditatus Abdel-Wahab & Nagahama
**23. *Haligena*** Kohlm.
*H. elaterophora Kohlm.
**24. *Halosarpheia*** Kohlm. & E. Kohlm.
*H. australiensis* (K.D. Hyde) McKeown, S.T. Moss & E.B.G. Jones, **comb. nov**.
*H. bentotensis* Jørg. Koch
*H. culmiperda* Kohlm., Volkm.-Kohlm. & O.E. Erikss.
*H. fibrosa Kohlm. & E. Kohlm.
*H. japonica* Abdel-Wahab & Nagahama
*H. minuta* W.F. Leong
*H. phragmiticola* Poon & K.D. Hyde
*H. trullifera* (Kohlm.) E.B.G. Jones, S.T. Moss & Cuomo
*H. unicellularis* Abdel-Wahab & E.B.G. Jones
**25. *Halosphaeria*** Linder
*H. appendiculata Linder
**26. *Halosphaeriopsis*** T.W. Johnson
*H. alopallonella* (Meyers & R.T. Moore) Devadatha, Day. & E.B.G. Jones, **comb. nov**.
*H. mediosetigera (Cribb & J.W. Cribb) T.W. Johnson
**27. *Havispora*** K.L. Pang & Vrijmoed
*H. longyearbyenensis K.L. Pang & Vrijmoed
**28. *Iwilsoniella*** E.B.G. Jones
*I. rotunda E. B. G. Jones
**29. *Jinshana*** K.L. Pang, M.W.L. Chiang & E.B.G. Jones
*Jinshana tangtangiae K.L. Pang, M.W.L. Chiang & E.B.G. Jones
**30. *Kitesporella*** Jheng & K.L. Pang
*K. keelungensis Jheng & K.L. Pang
**31. *Kochiella*** Sakay., K.L. Pang & E.B.G. Jones
*K. crispa (Kohlm.) Sakay., K.L. Pang & E.B.G. Jones
**32. *Lautisporopsis*** E.B.G. Jones, Yusoff & S.T. Moss
*L. circumvestita (Kohlm.) E.B.G. Jones, Yusoff & S.T. Moss
**33. *Lignincola*** Höhnk
*L. laevis Höhnk
*L. conchicola* Jian K. Liu, E.B.G. Jones & K.D. Hyde
*L. nypae* K.D. Hyde & Alias
*L. tropica* Kohlm.
**34. *Limacospora*** Jørg. Koch & E.B.G. Jones
*L. sundica (Jørg. Koch & E.B.G. Jones) Jørg. Koch & E.B.G. Jones
**35. *Luttrellia*** Shearer
*L. estuarina Shearer
*L. guttulata* A. Ferrer & Shearer
*L. halonata* A. Ferrer & Shearer
*L. parvulospora* A. Ferrer & Shearer
**36. *Magnisphaera*** J. Campb., J.L. Anderson & Shearer
*M. spartinae (E.B.G. Jones) J. Campb., J.L. Anderson & Shearer
*M. stevemossago* J. Campb., J.L. Anderson & Shearer
**37. *Marinospora*** A.R. Caval.
*M. calyptrata (Kohlm.) A.R. Caval.
*M. longissima* (Kohlm.) A.R. Caval. (Doubtfull species)
**38. *Moana*** Kohlm. & Volkm.-Kohlm.
*M. turbinulata Kohlm. & Volkm.-Kohlm.
**39. *Morakotiella*** Sakay.
*M. salina (C.A. Farrant & E.B.G. Jones) Sakay.
**40. *Naïs*** Kohlm.
*N. aquatica* K.D. Hyde
*N. inornata Kohlm.
*N. submersa* Huang Zhang & R. Yang
**41. *Natantispora*** J. Campb., J.L. Anderson & Shearer
*N. lotica* (Shearer) J. Campb., J.L. Anderson & Shearer
*N. retorquens (Shearer & J.L. Crane) J. Campb., J.L. Anderson & Shearer
*N. unipolaris* K.L. Pang, S.Y. Guo & E.B.G. Jones
**42. *Naufragella*** Kohlm. & Volkm.-Kohlm.,
*N. spinibarbata (Jørg. Koch) Kohlm. & Volkm.-Kohlm.
**43. *Nautosphaeria*** E.B.G. Jones
*N. cristaminuta E.B.G. Jones
**44. *Neoaniptodera*** Abdel-Wahab, K.L. Pang, P. Correia & E.B.G. Jones, **gen. nov**.
*N. juncicola (Volkm.-Kohlm. & Kohlm.) Abdel-Wahab, K.L. Pang, P. Correia & E.B.G. Jones, **comb. nov**.
**45. *Neogesasha*** Day., Abdel-Wahab, M.F. Caeiro & K.L. Pang, **gen. nov**.
*N. mangrovei (Abdel-Wahab & Nagahama) Day., Abdel-Wahab, M.F. Caeiro & K.L. Pang, **comb. nov**.
**46. *Neohalosarpheia*** Abdel-Wahab, K.L. Pang & P. Correia, **gen. nov**.
*N. marina (Cribb & J.W. Cribb) Abdel-Wahab, K.L. Pang & P. Correia, **comb. nov**.
**47. *Neptunella*** K.L. Pang & E.B.G. Jones
*N. longirostris (Cribb & J.W. Cribb) K.L. Pang & E.B.G. Jones
**48. *Nereiospora*** E.B.G. Jones, R.G. Johnson & S.T. Moss
*N. comata (Kohlm.) E.B.G. Jones, R.G. Johnson & S.T. Moss
*N. cristata* (Kohlm.) E.B.G. Jones, R.G. Johnson & S.T. Moss
**49. *Nimbospora*** Jørg. Koch
*N. effusa Jørg. Koch
*N. bipolaris* K.D. Hyde & E.B.G. Jones
**50. *Nohea*** Kohlm. & Volkm.-Kohlm.
*N. delmarensis* (Kohlm. & Volkm.-Kohlm.) Abdel-Wahab
*N. umiumi Kohlm. & Volkm.-Kohlm.
**51. *Oceanitis*** Kohlm.
*O. abyssalis* Y. Nagano & Abdel-Wahab
*O. cincinnatula* (Shearer & J.L. Crane) J. Dupont & E.B.G. Jones
*O. scuticella Kohlm.
*O. unicaudata* (E.B.G. Jones & Camp.-Als.) J. Dupont & E.B.G. Jones
*O. viscidula* (Kohlm. & E. Kohlm.) J. Dupont & E.B.G. Jones
**52. *Ocostaspora*** E.B.G. Jones, R.G. Johnson & S.T. Moss
*O. apilongissima E.B.G. Jones, R.G. Johnson & S.T. Moss
*O. japonica* Abdel-Wahab & E.B.G. Jones, **sp. nov**.
**53. *Okeanomyces*** K.L. Pang & E.B.G. Jones
*O. cucullatus (Kohlm.) K.L. Pang & E.B.G. Jones
*O. guttulatus* M. Li, Raza & L. Cai
*O. marinus* Calabon, E.B.G. Jones, Boonmee & K.D. Hyde
**54. *Ondiniella*** E.B.G. Jones, R.G. Johnson & S.T. Moss
*O. torquata (Kohlm.) E.B.G. Jones, R.G. Johnson & S.T. Moss
**55. *Ophiodeira*** Kohlm. & Volkm.-Kohlm.
*O. monosemeia Kohlm. & Volkm.-Kohlm.
**56. *Pangia*** Abdel-Wahab, M.F. Caeiro & E.B.G. Jones, **gen. nov**.
*P. limnetica (Shearer) Abdel-Wahab, M.F. Caeiro & E.B.G. Jones, **comb. nov**.
**57. *Panorbis*** J. Campb., J.L. Anderson & Shearer
*P. viscosus (I. Schmidt) J. Campb., J.L. Anderson & Shearer
**58. *Paraaniptodera*** K.L. Pang, C.L. Lu, W.T. Ju & E.B.G. Jones
*P. longispora (K.D. Hyde) K.L. Pang, C.L. Lu, W.T. Ju & E.B.G. Jones
**59. *Phaeonectriella*** R.A. Eaton & E.B.G Jones
*Ph. alba* Abdel-Wahab & Abdel-Aziz
*Ph. appendiculata* K.D. Hyde, W.H. Ho & C.K.M. Tsui
*Ph. lignicola R.A. Eaton & E.B.G. Jones
**60. *Pileomyces*** K.L. Pang & J.S. Jheng
*P. formosanus K.L. Pang & J.S. Jheng
**61. *Praelongicaulis*** E.B.G. Jones, Abdel-Wahab & K.L. Pang
*P. kandeliae (Abdel-Wahab & E.B.G. Jones) E.B.G. Jones, Abdel-Wahab & K.L. Pang
**62. *Pseudolignincola*** Chatmala & E.B.G. Jones
*P. siamensis Chatmala & E.B.G. Jones
**63. *Qarounispora*** Nourel-Din, Abdel-Aziz & Abdel-Wahab
*Q. grandiappendiculata Nourel-Din, Abdel-Aziz & Abdel-Wahab
**64. *Remispora*** Linder
*R. maritima Linder
*R. minuta* E.B.G. Jones, K.L. Pang & Vrijmoed
*R. pilleata* Kohlm.
**65. *Remisporiopsis*** K.L. Pang, E.B.G. Jones, E. Azevedo & M.F. Caeiro, **gen. nov**.
*R. macrocephala* (Meyers & R.T. Moore) K.L. Pang. E.B.G. Jones & M.F. Caeiro, **comb. nov**.
*R. quadri-remis* (Höhnk) K.L. Pang, E.B.G. Jones, E. Azevedo & M.F. Caeiro, **comb. nov**.
*R. spitsbergenensis* (K.L. Pang & Vrijmoed) K.L. Pang & Vrijmoed, **comb. nov**.
*R. stellatus (Kohlm.) K.L. Pang, E.B.G. Jones, E. Azevedo & M.F. Caeiro, **comb. nov**.
*R. submersa* (M. Gonçalves, A. Abreu & A. Alves) K.L. Pang, E.B.G. Jones, E. Azevedo & M.F. Caeiro, **comb. nov**.
**66. *Saagaromyces*** K.L. Pang & E.B.G. Jones
*S. abonnis* (Kohlm.) K.L. Pang & E.B.G. Jones
*S. glitra* (J.L. Crane & Shearer) K.L. Pang & E.B.G. Jones
*S. mangrovei* Abdel-Wahab, Bahkali & E.B.G. Jones
*S. ratnagiriensis (S.D. Patil & Borse) K.L. Pang & E.B.G. Jones
**67. *Sablicola*** E.B.G. Jones, K.L. Pang & Vrijmoed
*S. chinensis E.B.G. Jones, K.L. Pang & Vrijmoed
**68. *Safagamyces*** Bakhit & Abdel-Wahab
*S. marinus Bakhit & Abdel-Wahab **69. *Sheareromyces*** Abdel-Wahab, Maharachch. & E.B.G. Jones, **gen. nov**. *S. aquibella (J. Yang & K.D. Hyde) Abdel-Wahab, Maharachch. & E.B.G. Jones, **comb. nov**.
**70. *Shiiraspora*** P. Correia, Abdel-Wahab & E.B.G. Jones, **gen. nov**.
*S. salsuginosa (Nakagiri & Tad. Ito) P. Correia, Abdel-Wahab & E.B.G. Jones, **comb. nov**.
**71. *Thalassogena*** Kohlm. & Volkm.-Kohlm.
*Th. sphaerica Kohlm. & Volkm.-Kohlm
*Th. unicellularis* (Abdel-Wahab & Nagahama) Abdel-Wahab, **comb. nov**.
**72. *Thalespora*** Chatmala & E.B.G. Jones
*T. appendiculata Chatmala & E.B.G. Jones
**73. *Tinhaudeus*** K.L. Pang, S.Y. Guo & E.B.G. Jones
*T. formosanus K.L. Pang, S.Y. Guo & E.B.G. Jones
**74. *Tirispora*** E.B.G. Jones & Vrijmoed
*T. mandoviana* V.V. Sarma & K.D. Hyde
*T. unicaudata E.B.G. Jones & Vrijmoed
**75. *Toriella*** Sakay., K.L. Pang & E.B.G. Jones
*T. tubulifera (Kohlm.) Sakay., K.L. Pang & E.B.G. Jones
**76. *Trichomaris*** Hibbits, G.C. Hughes & Sparks
*T. invadens Hibbits, G.C. Hughes & Sparks
**77. *Tubakiella*** Sakay., K.L. Pang & E.B.G. Jones
*T. galerita (Tubaki) Sakay., K.L. Pang & E.B.G. Jones

#### Corollospora

Taxon classificationFungiMicroascalesHalosphaeriaceae

Werderm., Notizbl. Bot. Gart. Berl. 8 (73): 248 (1922)

9F1286A5-A22E-541A-80BE-B8646FFE53E3

Index Fungorum: IF1251

##### Description.

Saprobic on wood associated with wood. **Sexual morph. *Ascomata*** solitary or gregarious, globose to ellipsoidal, superficial, rarely submerged, with or without subiculum, ostiolate or nonostiolate, papillate or epapillate, carbonaceous rarely coriaceous, black, often on sand grains, and other calcareous material. ***Asci*** 8-spored, fusiform or clavate, unitunicate, thin-walled, early deliquescing. ***Ascospores*** ellipsoidal to fusiform, 1-septate to multi-septate, hyaline rarely brown, appendaged with or lacking polar spines with secondary appendages formed by fragmentation of an exosporic wall layer. **Asexual morph**. Hyphomycetous, conidia septate, terminal, branched staurospores, muriform, 2-celled with 2–3 polar extensions,

##### Type species.

*Corollospora maritima* Werderm., Notizbl. Bot. Gart. Berl. 8 (73): 248 (1922).

##### Notes.

**Figure 18.**
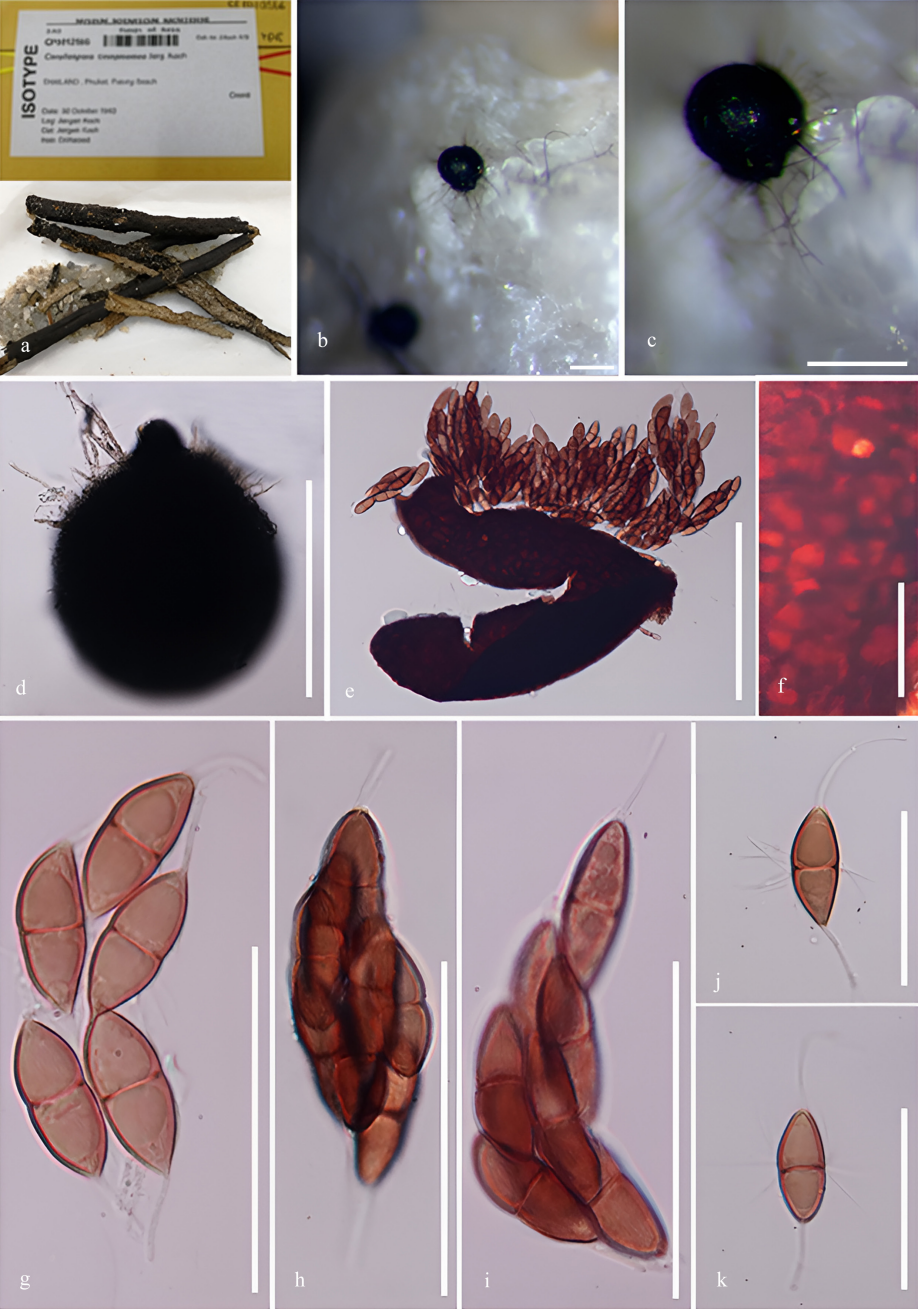
*Corollospora cinnamomea* (CP1012566, isotype and micro slides). **a**. Herbarium material; **b, c**. Ascomata on sand grain; **d**. Ascoma; **e**. Squash mount of ascoma (microslide); **f**. Peridium; **g–i**. Asci; **j–k**. Ascospores. Scale bars: 200 µm (**b, c**); 100 µm (**d**); 50 µm (**e, g–i**); 20 µm (**f, j, k**).

**Figure 19.**
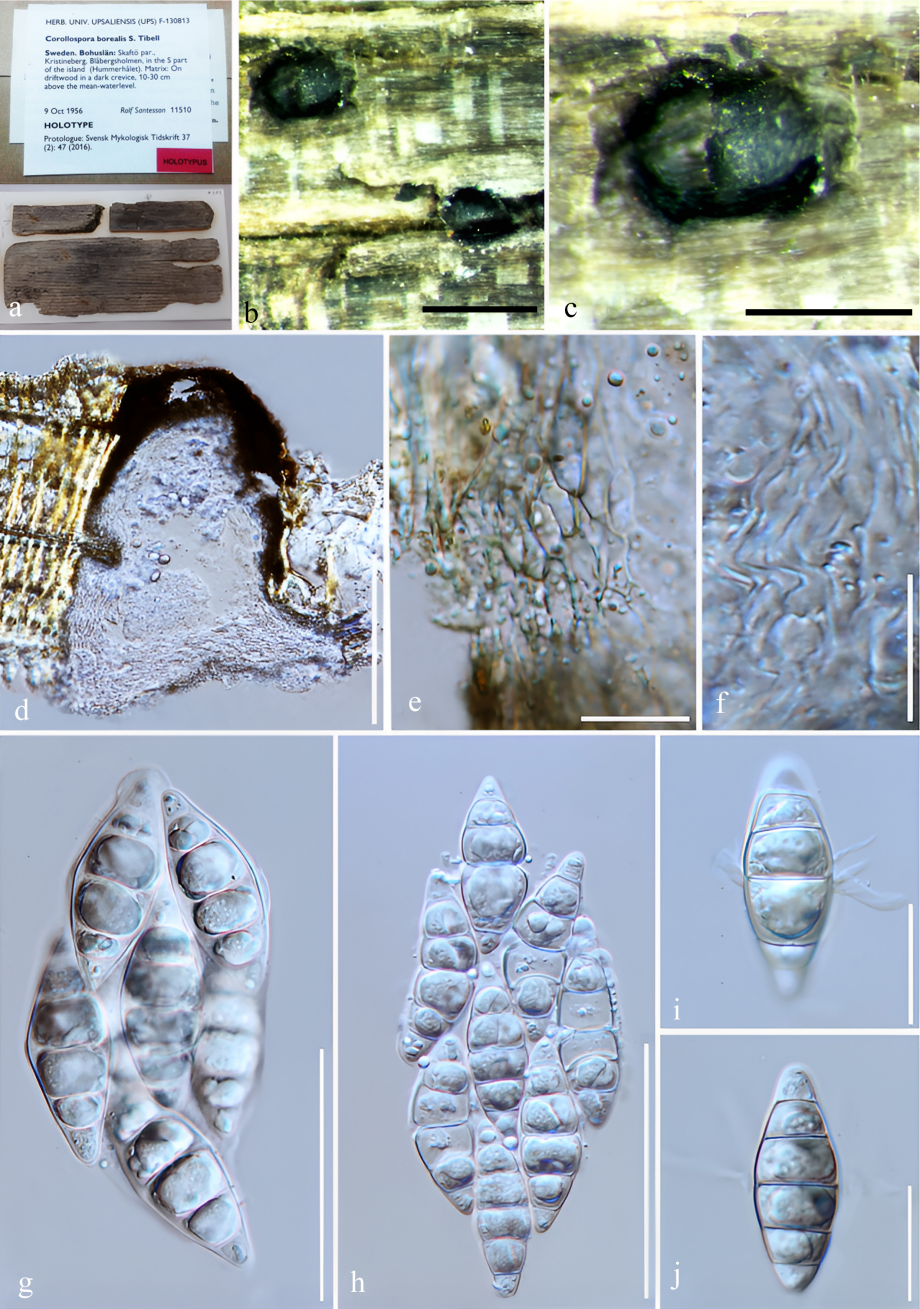
*Corollospora borealis* (UPS 11510, holotype). **a**. Herbarium material; **b, c**. Appearance of ascomata on host surface; **d**. Section through ascoma; **e**. Peridium; **f**. Paraphyses; **g, h**. Asci; **i, j**. Ascospores. Scale bars: 500 μm (**b, c**); 100 μm (**d**); 20 μm (**e, i, j**); 50 μm (**f–h**).

*Corollospora* species are saprobic on a wide range of substrates: driftwood, trapped wood, test panels submerged in the sea, mangrove wood, algae, seagrasses, on beach sand, sea-foam, shell fragments, and algal thalli. Thirty *Corollospora* species have been described, many lack sequence data (Suppl. material 1). Isotype of *C. cinnamomea* and holotype of *C. borealis* were examined and illustrated in Figs 18, 19, respectively.

##### Molecular evaluation.

*Corollospora* species formed a moderately supported monophyletic group in the multi-gene analysis in this study (Fig. 4). A wide range of genes have been sequenced for *Corollospora* species: 28S, 18S, ITS rDNA, rpb1, rpb2, tef-1α, and β-tub (Suppl. material 1), however most studies have been based on the nuclear cistron. Divergence-time analysis places the crown of *Corollospora* in the early Jurassic (~202 MYA, 95% CI: 74–547 MYA), highlighting its long independent evolutionary history within the Halosphaeriaceae (Fig. 5).

#### Corollospora maritima

Taxon classificationFungiMicroascalesHalosphaeriaceae

Werderm., Notizbl. Bot. Gart. Berl. 8 (73): 248 (1922)

EFE31177-CFFF-5A40-AE05-90BF7794EE4B

Index Fungorum: IF270595

Fig. 1e

Peritrichospora integra Linder, Farlowia 1 (3): 414 (1944). Synonym.

##### Description.

**Sexual morph. *Ascomata*** 190 × 230 µm, globose or subglobose, black, smooth, brilliant, with subicula on grains of sand, solitary or gregarious, carbonaceous, ostiolate. ***Peridium***, composed of flat, thick-walled angular cells. ***Asci*** 60–100 × 8–14 µm, 8-spored, fusiform or subclavate, thin-walled, without apical apparatus, deliquescing early. ***Ascospores*** 15–20 × 5–7 µm, fusiform, 1-septate, not or slightly constricted at septum, asymmetric, hyaline, appendaged; appendages of two kinds a) polar spines 12–16 µm long, slender, 0.5–1 µm thick, slightly curved, hyaline, and b) sheet-like, exosporic appendages up to 12 µm long from apical part of polar spine and equatorial appendages forming a double frill of appendages 6–12 µm long. **Asexual morph**. Undetermined.

##### Material examined.

Thailand, • Phuket, Patong beach, on driftwood, 30 Oct. 1983, J. Koch., CP1012566 isotype and micro slides.

##### Notes.

*Corollospora maritima* is characterized by 1-septate, hyaline ascospores with a prominent apical spine and a peridial wall with two layers, outer layer composed of roundish cells with large lumina, inner layer of flat, elongate cells with narrow lumina. This species is a common cosmopolitan species growing on a wide range of substrates, with ascospores common in sea foam. This species has been widely collected and sequenced, but many may not represent *C. maritima* (Pang et al. 2023a).

### *Cucullosporella* K.D. Hyde & E.B.G. Jones

*Cucullospora* was introduced by Hyde and Jones (1986), an invalid name, and changed to *Cucullosporella* by Jones and Hyde (1990), with *C. mangrovei* as the type species, from material collected at Brillant mangrove, Seychelles. It was referred to the Halosphaeriaceae as it had deliquescing asci and polar appendages to the ascospores, characteristics of the family.

#### Cucullosporella

Taxon classificationFungiMicroascalesHalosphaeriaceae

K.D. Hyde & E.B.G. Jones, Mycotaxon 37: 200 (1990)

5EF3575F-EA2F-562B-9D16-8A84CCBC4137

Index Fungorum: IF25506

Cucullospora K.D. Hyde & E.B.G. Jones, Bot. Mar. 29: 491 (1986). Synonym.

##### Description.

Saprobic on submerged and intertidal wood. **Sexual morph. *Ascomata*** solitary or gregarious, subglobose, ostiolate, periphysate, greyish brown to black, immersed and coriaceous. ***Pseudoparenchymatous***, lacking paraphyses but catenophyses present. ***Asci*** 8-spored, unitunicate, thin-walled, clavate, pedunculate, thickened at the ascus tip, persistent, arising from a central region at the base of the ascocarp. ***Ascospores*** ellipsoidal, 1-septate, not constricted at the septum, hyaline, polar appendages arising from an extension of the spore wall. ***Appendages*** initially hamate, uncoiling at maturity to form thread-like polar appendages. **Asexual morph**. Undetermined.

##### Type species.

*Cucullosporella mangrovei* (K.D. Hyde & E.B.G. Jones) K.D. Hyde & E.B.G. Jones, Mycotaxon 37: 200 (1990).

##### Notes.

*Cucullosporella* is saprobic on mangrove substrata (*Rhizophora apiculata*, *Rhizophora stylosa*, *Casuarina* sp., *Xylocarpus granatum*, *Rhizophora mucronata*, *Sonneratia acida*) and driftwood.

*Cucullosporella* is a monotypic genus that belongs to a group of aquatic fungi with ascospores with polar hamate appendages that unfurl to form fine thread–like appendages (Alias et al. 2001; Pang et al. 2003b). It differs from other such genera in having a polar tube–like structure through which the thread–like appendages are extruded (Alias et al. 2001).

##### Molecular evaluation.

Sequence data available for *Cucullosporella mangrovei* confirms its assignment to the Halosphaeriaceae, and in highly supported clade with *Paraaniptodera longispora* (Fig. 4). Divergence-time analysis indicates that this split dates to the late Cretaceous, near the K–Pg boundary (~66.3 MYA; 95% CI 18.8–233.6 MYA, spanning Miocene–Late Triassic), supporting recognition of *Cucullosporella* as an independent lineage within the family (Fig. 5).

#### Cucullosporella mangrovei

Taxon classificationFungiMicroascalesHalosphaeriaceae

(K.D. Hyde & E.B.G. Jones) K.D. Hyde & E.B.G. Jones, Mycotaxon 37: 200 (1990)

99C382B6-6CCB-5DED-ACFD-043D027B1928

Index Fungorum: IF136766

Fig. 3g

Cucullospora mangrovei (K.D. Hyde & E.B.G. Jones) K.D. Hyde & E.B.G. Jones, Mycotaxon 37: 200 (1990). Basionym.

##### Description.

Saprobic on mangrove wood. **Sexual morph. *Ascomata*** 150–220 µm high, 250–380 µm diam., subglobose, ovoid to ellipsoidal, greyish brown to black, coriaceous, immersed, solitary or gregarious, ostiolate. ***Peridium*** 27–45 µm thick, composed of an inner layer of elongate cells and an outer layer of thick–walled rounded cells. ***Necks*** 230–330 µm long, 50–75 µm diam., periphysate. ***Asci*** 180–(212)–250 × 36–(41)–46 µm, thin–walled, unitunicate, 8–spored, clavate, persistent, thickened at the ascus tip with a lens–shaped refractive region, pedicellate. ***Catenophyses*** present. ***Ascospores*** 48–(57)–64 µm × 16–(19)–24 µm, hyaline, fusoid to ellipsoidal, 1–septate, not constricted at the septum, appendaged. ***Appendages*** 6–12 µm × 4–8 µm, arising from an extension of the spore wall, unfurling into fine threads in water. **Asexual morph**. Undetermined.

##### Notes.

Molecular studies place this species in the Halosphaeriaceae forming a sister group to *Aniptodera longispora* (=*Paraaniptodera longispora*) (Sakayaroj et al. 2011; Jones et al. 2017). *Cucullosporella* is characterized by hyaline 1-septate ascospores with uncoiling polar appendages arising from a hood or cap-like structure. Owing to these characters the genus was assigned to the Halosphaeriaceae (Jones 1995). In recent years a number of freshwater and marine ascomycetes have been described with polar, hamate appendages that uncoil in water. Many of these have been studied at the ultrastructural level. *Cucullosporella mangrovei* differs from other species with polar, hamate appendages in that the polar appendages arise from a hood or cap-like structure which is an outgrowth of the mesosporium and bounded by the episporium. Appendages may arise through pores in the episporium within the hood (Hyde and Jones 1986).

### *Cucurbitinus* L.L. Liu & Z.Y. Liu

*Cucurbitinus* genus was introduced by Liu et al. (2020) to accommodate *Trichocladium constrictum* as *Cucurbitinus constrictus* and *Cirrenalia iberica* as *Cucurbitinus ibericus* with morphological illustrations and molecular data. *Trichocladium constrictum* was introduced by Schmidt (1974a) from collections on submerged wood in the Baltic Sea, Germany. *Trichocladium* is a polyphyletic genus with most assigned to the Chaetomiaceae.

#### Cucurbitinus

Taxon classificationFungiMicroascalesHalosphaeriaceae

L.L. Liu & Z.Y. Liu, Phytotaxa 455 (2): 124 (2020)

312BE12C-2811-51A8-A6C2-2B38A42B25A9

Index Fungorum: IF557758

##### Description.

Saprobic on dead decaying wood in aquatic or terrestrial habitats. **Asexual morph. *Colonies*** brown to dark brown, glistening, effuse, hairy, velvety. ***Mycelium*** partly superficial to immersed, septate, composed of hyaline to pale brown, branched hyphae. ***Conidiophores*** solitary, pale brown to brown, micronematous or macronematous, mononematous, cylindrical, straight or slightly flexuous, septate, smooth, thick-walled. ***Conidiogenous cells*** pale brown to brown, smooth, polyblastic or monoblastic, terminal or intercalary, cylindrical. ***Conidia*** mid-brown, acropleurogenous, solitary, straight or slightly curved, 1–4-septate, strongly constricted at the septa, clavate, gourd shaped, guttulate, smooth-walled. **Sexual morph**. Undermined (Adapted from Liu et al. 2020).

##### Type species.

*Cucurbitinus constrictus* (I. Schmidt) L.L. Liu & Z.Y. Liu, in Liu, Liu, Yang, Chen and Liu, Phytotaxa 455(2): 126 (2020).

##### Notes.

*Cucurbitinus* species are saprobic on dead decaying wood in aquatic or terrestrial habitats (Liu et al. 2020).

Morphologically the conidia of *Cucurbitinus* resembles those of *Cirrenalia* and *Trichocladium*, but not phylogenetically. *Cucurbitinus constrictus* clustered with *C. iberica* (= *Cirrenalia iberica*) in a clade including marine Ascomycota and a sister clade comprising two strains of *Cirrenalia macrocephala*.

##### Molecular evaluation.

Molecular analysis for this genus placed it in a distinct clade in the Halosphaeriaceae. Also, the sequencing of new isolates is required. Divergence-time analysis indicates that it separated from allied genera such as *Thalespora* and *Okeanomyces* during the Paleogene–Neogene interval (~ 13 MYA; 95% CI 2.7–59.5 MYA) (Fig. 5). Additional collections and sequencing of new isolates are required to better resolve its phylogenetic placement and confirm its circumscription.

#### Cucurbitinus constrictus

Taxon classificationFungiMicroascalesHalosphaeriaceae

(I. Schmidt) L.L. Liu & Z.Y. Liu, Phytotaxa 455 (2): 126 (2020)

3F430026-597D-58EA-9C88-F7778B85E36F

Index Fungorum: IF557757

Trichocladium constrictum I. Schmidt, Mycotaxon 24: 419 (1985). Basionym.
Trichocladium constrictum I. Schmidt, Natur Naturschutz Mecklenburg 12: 114 (1974). Synonym.
Trichocladium angelicum A. Roldán & Honrubia, Mycotaxon 35(2): 353 (1989).

##### Description.

Saprobic on decaying plant substrates. **Asexual morph. *Colonies*** brown to dark brown, hairy, velvety, effuse, glistening. ***Mycelium*** semi-immersed, composed of hyaline to pale brown, septate, branched hyphae. ***Conidiophores*** pale brown to brown, macronematous, mononematous, solitary, cylindrical, straight or slightly flexuous, 5–6 septate, even, thick-walled, cylindrical, 40–80.5(–90) × 1.5–3 μm. ***Conidiogenous cells*** cylindrical, monoblastic or polyblastic, terminal, sympodial, smooth. ***Conidia*** acropleurogenous, mid-brown, solitary, straight or slightly curved, 2–4-septate, constricted at the septa, clavate, gourd-shaped, guttulate, smooth-walled, 26.5–43 × 9–12.5 μm, with cells commonly different in size from the base to the apex; basal cells hyaline to subhyaline, subglobose to cuneiform, tubulate, 4–7.5 × 2.7–4.5 μm; middle and apical cells globose to subglobose, apical cell 8.5–12.5 × 8–12.5 μm. Conidial secession schizolytic. **Sexual morph**. Undetermined (Adapted from Liu et al. 2020).

##### Notes.

*Trichocladium constrictum* is distinguishable from other *Trichocladium* species by long conidiophores, gourd-shaped conidia which are strongly constricted at the septa, the apical cell larger than the other cells in the conidium (Schmidt 1974b). Liu et al. (2020) showed that *T. constrictum* was distantly placed from other *Trichocladium* species (Chaetomiaceae) and introduced the new genus *Cucurbitinus*. Liu et al. (2020) also synonymized *Trichocladium angelicum* under *Cucurbitinus constrictus* based on morphological and phylogenetic data.

##### Distribution.

Germany (Baltic Sea), China (Baihua Lake), Portugal.

#### Cucurbitinus ibericus

Taxon classificationFungiMicroascalesHalosphaeriaceae

(Hern.-Restr. & Gené) L.L. Liu & Z.Y. Liu, Phytotaxa 455 (2): 126 (2020)

2CFF7B5E-7690-584D-8A6A-391ADD44E8D1

Index Fungorum: IF557756

Cirrenalia iberica Hern.-Restr. & Gené, Stud. Mycol. 86: 86 (2017). Basionym.

##### Notes.

987 registered at NCBI, one ITS rDNA sequence of *C. macrocephala*, strain FMR 12418, while Hernández-Restrepo et al. (2017) described the new species *Cirrenalia iberica* based on two strains and *Cirrenalia iberica*, FMR 12418 was one of them.

### *Ebullia* K.L. Pang

*Ebullia* is a monotypic genus created to accommodate *Nimbospora octonae* (Kohlmeyer 1985), a marine fungus described from driftwood in Hawaii. Phylogenetically, *Ebullia* was related to *Haligena* but phylogenetically unrelated to the type species of *Nimbospora*, *N. effusa* and *N. bipolaris* (Chu et al. 2015).

#### Ebullia

Taxon classificationFungiHalosphaerialesHalosphaeriaceae

K.L. Pang, Mycoscience 56: 40 (2014)

473ECAB5-5B2A-5659-B9A7-919EF8194CBE

Index Fungorum: IF803444

##### Description.

Saprobic on wood. **Sexual morph. *Ascomata*** subglobose to ampulliform, immersed, ostiolate, with long necks, coriaceous, dark brown to black above, hyaline to brown below. ***Peridium*** two-layered, much thicker at the base. ***Necks*** periphysate. ***Asci*** 8-spored, clavate, pedunculate, unitunicate, thin-walled, early deliquescing, developing at the base of the ascoma venter on a convex cushion of ascogenous cells. ***Ascospores*** ellipsoidal, 1-septate, not or slightly constricted at the septum, hyaline, with a gelatinous sheath enclosing 6–7 subulate equatorial appendages evenly distributed around the septum and one similar appendage at each end; swells in water. **Asexual morph**. Undetermined.

##### Type species.

*Ebullia octonae* (Kohlm.) K.L. Pang, Mycoscience 56: 40 (2014).

##### Notes.

*Ebullia* (*E. octonae*) is similar to *Nimbospora* (*N. effusa*, *N. bipolaris*) by having a spore sheath but differs from them in lacking the tuft-like equatorial appendages, but having subulate appendages at equatorial and polar positions of the ascospores after the sheath has dissolved (Kohlmeyer 1985).

##### Molecular evaluation.

The two isolates of *E. octonae* formed a branch, separate from other taxa in the Halosphaeriaceae in the multi-gene analysis (Fig. 4), distantly placed from the *Nimbospora* clade. Divergence-time estimates indicate that *Ebullia* diverged from its nearest relatives around 203 MYA (95% CI 74.3–552.7 MYA), underscoring its long and independent evolutionary history within the family (Fig. 5).

#### Ebullia octonae

Taxon classificationFungiHalosphaerialesHalosphaeriaceae

(Kohlm.) K.L. Pang, Mycoscience 56: 40 (2014)

710E637C-00D7-55E1-ADC2-DAD56C9BEAA1

Index Fungorum: IF805905

Nimbospora octonae Kohlm., Canad. J. Bot. 63 (6): 1122 (1985). Basionym.

##### Description.

Saprobic on wood. **Sexual morph. *Ascomata*** 850–1000 µm high, 600–1250 µm in diam., subglobose to ampulliform, immersed, ostiolate, with long necks, coriaceous, dark brown to black above, hyaline to brown below. ***Peridium*** 70–100 µm thick, two-layered; outer layer 40–60 µm thick, dark brown, irregular, growing into the tracheids and fibers of the substrate; inner layer 30–40 µm thick, hyaline, composed of flat cells, forming a *textura angularis* or *prismatica*, merging with the pseudoparenchyma of the centrum. ***Necks*** 200–500 µm long, 80–150 µm in diam., dark brown to black, ostiolar canal periphysate; periphyses surrounded by a gelatinous mass. ***Pseudoparenchyma*** thin-walled, polygonal cells filling the centrum of young ascomata, dissolving before ascospore maturity. ***Asci*** 110–135 × 35 µm, 8-spored, clavate, pedunculate, unitunicate, thin-walled, aphysoclastic, maturing successively. ***Ascospores*** 22–29(–31) × 12–16(–18) µm, ellipsoidal, 1-septate, not or slightly constricted at the septum, hyaline, with a gelatinous sheath enclosing 6 subulate equatorial, 12–20 µm long appendages and one similar appendage at each end; the equatorial appendages are evenly distributed around the septum; the sheath swells in water from 3–4 to 22 µm; the sheath shrink completely onto appendages and the spore wall and the formerly hidden appendages become visible. **Asexual morph**. Undetermined.

##### Notes.

*Ebullia octonae* is saprobic on wood (Kohlmeyer 1985; Chu et al. 2015).

##### Distribution.

Brunei, Hawaiian Islands, India, Taiwan.

### *Fluviatispora* K.D. Hyde

*Fluviatispora* was introduced by Hyde (1994) to accommodate two taxa, *F. reticulata* and *F. tunicata* which were collected from freshwater habitats and the later species designated as the type. A third species, *F. boothii* was described from submerged wood in brackish water. The genus is characterized by hyaline, unicellular ascospores surrounded by a mucilaginous sheath, thin-walled and clavate to saccate, early deliquescing unitunicate asci and immersed thin-walled ascomata (Hyde 1994).

#### Fluviatispora

Taxon classificationFungiMicroascalesHalosphaeriaceae

K.D. Hyde, Mycol. Res. 98: 720 (1994)

0B4C057E-D4A4-5FEE-85ED-8E75A883A3E1

Index Fungorum: IF22416

##### Description.

Saprobic on submerged wood in fresh or brackish water. **Sexual morph. *Ascomata*** globose to subglobose or ellipsoidal, immersed in wood, light-brown, ostiolate, membranous and solitary. ***Asci*** 8-spored, clavate or saccate, thin-walled, unitunicate, pedunculate and deliquescing early. ***Ascospores*** 2–3-seriate, unicellular, ellipsoidal, hyaline, with a thin mucilaginous sheath. **Asexual morph**. Undetermined.

##### Type species.

*Fluviatispora tunicata* K.D. Hyde, Mycol. Res. 98: 722 (1994).

##### Notes.

This genus is represented by two freshwater (Hyde 1994) and one brackish water species occurring on submerged wood in the tropics (Hyde 1994; Fryar and Hyde 2004). *Fluviatispora* possess characters that are common in Halosphaeriaceae and includes: asci are clavate, thin-walled and deliquesce early and hyaline ascospores with either a thin or wide spreading gelatinous sheath.

##### Molecular evaluation.

Molecular data is not available for the three *Fluviatispora* species. These species need to be recollected, isolated and sequenced to determine their placement in the Halosphaeriaceae.

#### Fluviatispora tunicata

Taxon classificationFungiMicroascalesHalosphaeriaceae

K.D. Hyde, Mycol. Res. 98: 722 (1994)

B5F554FA-E7D1-599D-ABD4-2040CF543791

Index Fungorum: IF362329

##### Description.

Saprobic on submerged wood in fresh or brackish water. **Sexual morph. *Ascomata*** immersed, up to 150 µm diam., globose, subglobose or ellipsoidal, light-brown, ostiolate, thin-walled, membranous and solitary. ***Necks*** short, periphyses not seen. ***Peridium*** 10–14 µm thick, comprising thin-walled, compressed cells internally and brown-walled angular cells externally. ***Catenophyses*** not seen. ***Asci*** 62–102 × 24–45 µm, 8-spored, clavate to saccate, thin-walled, unitunicate, pedunculate and deliquescing early. ***Ascospores*** 24–33 × 12–15 µm, 2–3-seriate, unicellular, ellipsoidal, hyaline, with two large lipid globules, smooth walled, with an irregular spreading mucilaginous sheath. **Asexual morph**. Undetermined.

##### Notes.

Sequence data is not available for the three *Fluviatispora* species and they are only known from the type localities. *Fluviatispora reticulata* has larger ascospores dimensions than the other two species with an irregular spreading mucilaginous sheath; *F. reticulata* has longer and wider ellipsoidal ascospores than *F. boothii*. The latter species has fusiform ascospores with a narrow sheath. In *F. reticulata* the ascospores are ovoid and wider with radiating striations on the wall as well as a sheath.

##### Distribution.

Papua New Guinea.

### *Gesasha* Abdel-Wahab & Nagah.

The genus *Gesasha*, typified by *G. peditatus*, was described from Gesashi mangroves, Okinawa, Japan (Abdel-Wahab and Nagahama 2011), and a further two species described were *G. unicellularis* and *G. mangrovei*. In this study, *Gesasha* was shown to be polyphyletic based on a multi-gene phylogenetic analyses.

#### Gesasha

Taxon classificationFungiMicroascalesHalosphaeriaceae

Abdel-Wahab & Nagah., Nova Hedwigia 92 (3–4): 501 (2011)

A8389228-1EDF-5D08-A767-0D2CE8E8DE36

Index Fungorum: IF518670

##### Description.

Saprobic on decaying mangrove wood. **Sexual morph. *Ascomata*** subglobose, elongate, ovate, hyaline to light brown, coriaceous, immersed or erumpent, solitary, ostiolate with a long hyaline neck. ***Necks*** hyaline, cylindrical, periphysate. ***Peridium*** thick-walled cells with large lumina, forming *textura angularis*, cells hyaline except the first outside layer which is brown in color. ***Catenophyses*** present. ***Asci*** unitunicate, clavate with a long stalk, with apical thickening and pore, apically truncate with cytoplasmic retraction, persistent, with eight overlapping biseriate ascospores, developing in a hymenium at base of ascoma venter. ***Ascospores*** 1-septate, subglobose, ellipsoidal or foot-like, with sub-median septum, distoseptate, not constricted at the septa, thick-walled, the walls being 2–2.5 μm thick, hyaline becoming light brown with age, with polar to sub-apical amorphous, ephemeral appendages, smooth. **Asexual morph**. Undetermined.

##### Type species.

*Gesasha peditatus* Abdel-Wahab & Nagah., Nova Hedwigia 92 (3–4): 502 (2011).

##### Notes.

*Gesasha* is distinguished from other morphologically similar genera in the family Halosphaeriaceae by its ascospore morphology which includes: distoseptate, thick-walled ascospores with sub-median septum, foot-like shape and the amorphous apical to sub-apical ephemeral appendages. Several genera of marine ascomycetes have bicelled ascospores with ephemeral polar appendages, e.g. *Aniptodera* and *Halosarpheia*. Fungi belonging to the *Aniptodera*/*Halosarpheia* complex have polar unfurling appendages.

In the multigene phylogenetic analyses in this study (Fig. 4), the three species of *Gesasha* formed a monophyletic group with *Moana* (type species *M. turbinulata*) with high Bayesian support. *Gesasha mangrovei* is transferred to the new genus *Neogesasha* while *G. unicellularis* is transferred to the monotypic genus *Thalassogena*. There is no molecular data available for *Thalassogena sphaerica*, however, both taxa have common morphological characters and we believe that both fungi are better placed in the same genus.

Morphologically the four genera are clearly delineated: *Gesasha peditatus* ascospores 1-septate, thick-walled with polar to sub-apical amorphous, ephemeral appendages, asci clavate with a long stalk, with apical thickening and pore and cytoplasmic retraction, persistent; *Moana turbinulata* asci clavate, pedunculate, apically rounded, without a pore, more or less persistent, ascospores unicellular, with a top-like polar appendage, ribbon-like which uncoils in water; *Neogesasha mangrovei* asci clavate with an apical thickening and pore, apically truncate with cytoplasmic retraction, persistent, ascospores 1-septate, thick-walled, not constricted at the septum, and lacking appendages; and *Thalassogena unicellularis* asci with apical thickening and ring, apically truncate with cytoplasmic retraction, persistent, ascospores subglobose, rarely ellipsoidal, unicellular hyaline, lacking sheaths or appendages, with one large lipid globule surrounded by numerous small non-lipoid droplets.

The phylogenetic analyses also confirmed that *Gesasha peditatus* is unrelated to the complex with unfurling ascospore appendages.

##### Molecular evaluation.

Only the 28S rDNA of *G. peditatus* is available for phylogenetic analysis. This species needs to be recollected, isolated and sequenced for multiple genes. Divergence-time analysis shows that *Gesasha* separated from *Neogesasha* around ~68 MYA (95% CI: 21–222 MYA), supporting its recognition as a distinct but poorly sampled lineage within the Halosphaeriaceae (Fig. 5).

#### Gesasha peditatus

Taxon classificationFungiMicroascalesHalosphaeriaceae

Abdel-Wahab & Nagah., Nova Hedwigia 92 (3–4): 502 (2011)

67C0D0E0-7DC9-5CAF-BB37-0511A0FA627B

Index Fungorum: IF518681

Fig. 20

##### Description.

Saprobic on decaying, intertidal mangrove wood. **Sexual morph. *Ascomata*** 200–320 × 260–320 μm, subglobose, elongate, ovate, hyaline to light brown, coriaceous, immersed or erumpent, solitary, ostiolate with a long hyaline neck. ***Necks*** 300–800 × 60–90 μm, hyaline, cylindrical, periphysate, periphyses up to 22 μm in length and 0.5 μm in width. ***Peridium*** 20–24 μm wide, consisting of 7–9 layers of thick-walled cells with large lumina, forming *textura angularis* cells, hyaline except the first outside layer which is brown in color. ***Catenophyses*** present. ***Asci*** 100–150 × 20–30 μm, unitunicate, clavate with a long stalk, with apical thickening and pore, apically truncate with cytoplasmic retraction, persistent, with eight overlapping biseriate ascospores. ***Ascospores*** 18–30 × 9–16 μm, 1-septate, subglobose, ellipsoidal or foot-like, with sub-median septum, distoseptate, not constricted at the septa, thick-walled, the walls 2–2.5 μm thick, hyaline becoming light brown with age, with polar to sub-apical amorphous, ephemeral appendages, smooth. **Asexual morph**. Undetermined.

**Figure 20.**
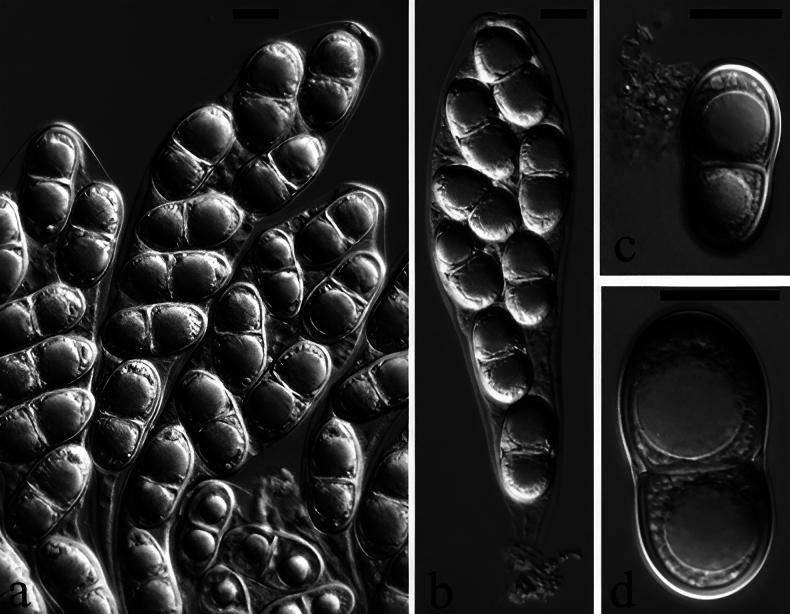
*Gesasha peditatus***. a, b**. Mature asci; **c, d**. Ascospores. Scale bars: 5 µm (**a–d**).

##### Material examined.

Japan, • Okinawa, Higashi-son, Gesashi mangroves, on decaying mangrove wood collected from the intertidal zone, July 2008, coll. M.A. Abdel-Wahab (IMI 397958, holotype).

##### Notes.

After the taxonomic revision proposed above, *Gesasha* becomes a monotypic marine genus, occurring on decaying mangrove wood (Abdel-Wahab and Nagahama 2011).

### *Haligena* Kohlm.

The genus *Haligena* was introduced by Kohlmeyer (1961) with *Haligena elaterophora* as the type species. Other taxa were referred to this genus: *Haligena spartinae* (Jones 1962), *H. viscidula* (Kohlmeyer and Kohlmeyer 1965), *H. unicaudata* (Jones and Le Campion-Alsumard 1970), *H. amicta* (Kohlmeyer and Kohlmeyer 1979), and *H. salina* (Farrant and Jones 1986). As the result of further morphological studies and sequence data, *Haligena* species were referred to other genera: *Haligena amicta* to *Appendichordella* (Johnson et al. 1987), *H. spartinae* to *Magnisphaera* (Anderson et al. 2001), *H. unicaudata* and *H. viscidula* to *Oceanitis* (Dupont et al. 2009), and *H. salina* to *Morakotiella* (Sakayaroj et al. 2005). Currently the genus *Haligena* only has one accepted species.

#### Haligena

Taxon classificationFungiMicroascalesHalosphaeriaceae

Kohlm., Nova Hedwigia 3: 87 (1961)

B38448FA-2C20-503A-8656-B62818957092

Index Fungorum: IF2204

##### Description.

Saprobic on well decayed wood in marine environments. **Sexual morph. *Ascomata*** are solitary or gregarious, globose to ovoid, immersed or superficial, sub carbonaceous, black, ostiolate, papillate, periphysate. ***Peridium*** composed of two cell layers, the outer thick-walled with irregular cells and the inner with elongate cells and a thin-walled pseudoparenchyma that forms catenophyses. ***Asci*** develop at the base of the ascocarp, 8-spored, clavate, apiculate, pedunculate, unitunicate, thin-walled, and deliquescing early. ***Ascospores*** are oblong ellipsoidal, 3–5 septate, constricted at the septa, hyaline with polar strap or tube-like appendages, that arise as outgrowths of the spore wall and are composed of an inner amorphous component and a narrower outer more compact material, with a discontinuous episporium beneath the area of attachment; the spore wall is composed of the episporium and mesosporium, the exosporium is absent (Adapted from Johnson et al. 1987). **Asexual morph**. Undetermined.

##### Type species.

*Haligena elaterophora* Kohlm., Nova Hedwigia 3: 87 (1961).

##### Notes.

*Haligena* is frequently encountered in marine habitats especially on decayed wood in temperate regions (Sakayaroj et al. 2005). This genus is characterized by having 3–5-septate ascospores, with long, strap-like polar appendages that arise as outgrowths of the spore wall, lacking an exosporium to the spore wall and with no equatorial appendages (Johnson et al. 1987), which resemble those of *Ceriosporopsis*, but differ in that the latter has ascospores with an exosporium (Johnson et al. 1987).

##### Molecular evaluation.

Previous studies showed that *Haligena* formed a sister clade with the genus *Ebullia* (Chu et al. 2015). However, in our analysis (Fig. 4), *Haligena* occupied a basal position, forming the earliest diverging lineage in the Halosphaeriaceae. Divergence-time estimates place the crown of *Haligena* at ~27 MYA (95% CI: 10–75 MYA), indicating that although morphologically distinctive, its diversification is comparatively recent within this ancient family (Fig. 5).

#### Haligena elaterophora

Taxon classificationFungiMicroascalesHalosphaeriaceae

Kohlm., Nova Hedwigia 3: 87 (1961)

67CE03C4-F7F7-59AA-A982-BE32B4ED95F8

Index Fungorum: IF331644

##### Description.

Saprobic on submerged wood in marine environment. **Sexual morph. *Ascomata*** 205–580 µm in diam., globose or ovoid, immersed or superficial, ostiolate, papillate or epapillate, subcoriaceous or subcarbonaceous, black, solitary or gregarious. ***Peridium*** 13–29 µm thick, composed on the outside of thick-walled irregular cells, forming a *textura epidermoidea*. ***Papillae*** short or absent, conical; ostiolar canal periphysate. ***Pseudoparenchyma*** of thin-walled cells filling the venter of young ascocarps; eventually breaking up into catenophyses. ***Asci*** 82–184 × 22.5–49.5 µm, 8-spored (rarely four), clavate and somewhat apiculate, pedunculate, unitunicate, thin-walled, aphysoclastic, without an apical apparatus, early deliquescing; developing at the base of the ascocarp venter on a small-celled ascogenous tissue. ***Ascospores*** 24–54.5 × 10–17.5 µm (excluding appendages), biseriate, oblong ellipsoidal, 3-septate (rarely up to five), constricted at the septa, hyaline, appendaged; immature ascospores surrounded by a gelatinous sheath that expands at maturity, remaining attached with a cone-shaped or semi globose protrusion to both apices; expanded elater-like appendages, curved, attenuate, channeled, spoon-shaped at the base. **Asexual morph**. Undetermined (Description based on Kohlmeyer 1961).

##### Notes.

*Haligena elaterophora* has been widely collected in the temperate waters of the Atlantic and Pacific Oceans, with collections from Denmark, Germany, Spain, Sweden, UK and the USA

### *Halosarpheia* Kohlm. & E. Kohlm.

The genus *Halosarpheia* was introduced by Kohlmeyer and Kohlmeyer (1977) to accommodate a marine fungus *Halosarpheia fibrosa*, with coriaceous, brown to black ascomata, long necks; persistent, clavate asci and 1-septate, hyaline ascospores with polar cap-like appendages that uncoil in water into long filaments (Kohlmeyer and Kohlmeyer 1977). Later, a number of species with similar type of ascospore appendages have been added from freshwater and marine habitats or were transferred from other genera. The genus was referred to the Halosphaeriaceae by Kohlmeyer and Kohlmeyer (1977) and this was supported by molecular data (Campbell et al. 2003; Jones et al. 2015). Molecular studies confirmed that the genus is polyphyletic (Kong et al. 2000; Abdel-Wahab et al. 2001; Anderson et al. 2001; Pang et al. 2003b), and this led to the introduction of a number of new genera including: *Ascosacculus* (*A. aquaticus*, *A. heteroguttulatus*), *Magnisphaera* (*M. spartinae*), *Natantispora* (*N. lotica*, *N. retorquens*), *Oceanitis* (*O. cincinnatula*, *O. unicaudata*, *O. viscidula*), *Panorbis* (*P. viscosus*), *Praelongicaulis* (*P. kandeliae*) and *Saagaromyces* (*S. abonnis*, *S. ratnagiriensis*) (Campbell et al. 2003; Pang et al. 2003a, 2003b; Dupont et al. 2009; Jones et al. 2015). *Halosarpheia aquadulcis* was transferred to the genus *Aniptodera* (Campbell et al. 2003).

#### Halosarpheia

Taxon classificationFungiMicroascalesHalosphaeriaceae

Kohlm. & E. Kohlm., Trans. Brit. Mycol. Soc. 68 (2): 208 (1977)

CB22E2BB-A8FC-59D4-90FC-50FC22884B7D

Index Fungorum: IF2208

##### Description.

Saprobic on submerged woody substrates in marine habitats. **Sexual morph. *Ascomata*** perithecial, coriaceous, brown to black, solitary or gregarious, globose, subglobose, obpyriform, ellipsoidal, immersed to superficial, ostiolate, papillate or with long necked, multi-layered peridial wall. ***Catenophyses*** present. ***Asci*** 8-spored, clavate, unitunicate, pedicellate, thin-walled with or without apical thickening, persistent or deliquesce early. ***Ascospores*** ellipsoidal, globose, subglobose, ovate, 0–1-septate, with one or two polar appendages. ***Appendages*** coiled filaments enclosed in gelatinous membranes or amorphous material enclosed in cellular sheath that dissolves in water and the appendage swells to form long thread-like appendages. **Asexual morph**. Undetermined.

##### Type species.

*Halosarpheia fibrosa* Kohlm. & E. Kohlm., Trans. Brit. Mycol. Soc. 68 (2): 208 (1977).

##### Notes.

*Halosarpheia* species have been reported from decaying mangrove wood such as *Avicennia germinans*, *Rhizophora mangle* and *Tamarix gallica* (Kohlmeyer and Kohlmeyer 1977), pneumatophores of *Laguncularia racemosa* (Kohlmeyer 1984), *Bruguiera parviflora*, *Kandelia candel* and *Sonneratia alba* (Lu et al. 2000), *Avicennia marina* (Abdel-Wahab et al. 2001), *Rhizophora mucronata* (Pande 2008), and other substrates e.g., senescent culms of *Juncus roemerianus*, leaf sheaths and stem of *Phragmites australis*, decaying wood in the sea (Koch 1982; Leong et al. 1991; Kohlmeyer et al. 1995; Poon and Hyde 1998; Abdel-Wahab and Nagahama 2012).

The polyphyletic nature of *Halosarpheia* has been reported based on the phylogeny of 18S and 28S rDNA sequences in early reports (Kong et al. 2000; Abdel-Wahab et al. 2001; Anderson et al. 2001; Pang et al. 2003b). In this study, four *Halosarpheia* species (*H. fibrosa*, *H. trullifera*, *H. japonica* and *H. unicellularis*) formed a monophyletic group with *Tunicatispora australiensis*. *Tunicatispora australiensis* is transferred to *Halosarpheia*. *Halosarpheia marina* was placed in a clade phylogenetically distant from *Halosarpheia* and grouped with species of *Nimbospora* but with no significant bootstrap support. Morphologically, *Halosarpheia marina* has characteristics of *Halosarpheia* and *Aniptodera*, but the phylogenetic analysis indicated it is not closely related to species in either genus. It was characterized by having clavate to subcylindrical asci with short pedicel and ascospores 18–23(–26) × 9–12 μm with appendages cap-like, then filamentous (Monod 1983; Kohlmeyer 1984). A new genus, *Neohalosarpheia*, is established from *H. marina*.

Currently, nine species are recognized in the genus *Halosarpheia*, recorded from marine habitats (Calabon et al. 2022). *Halosarpheia australiensis*, *H. fibrosa*, *H. japonica*, *H. trullifera*, *H. unicellularis* with molecular data, while *H. bentotensis*, *H. culmiperda*, *H. minuta* and *H. phragmiticola* lack molecular data (Jones et al. 2009, 2015, 2019). The five *Halosarpheia* species with molecular data share the following morphological features: large, dark brown to black, coriaceous ascomata with long periphysate necks; thick multi-layered peridial wall and globose, ovoid, widely ellipsoidal ascospores. These species differ from one another in ascomal shape and pigmentation, peridial cell wall thickening, presence or absence of a beak in immature asci, degree of ascus deliquescence, and ascospore size and septation (Abdel-Wahab and Nagahama 2012). *Halosarpheia japonica* differs from other *Halosarpheia* species in having an asexual morph with brown, helicoid conidia. Conidia are globose to subglobose, similar in color, generally increase in size from base to apex and tightly joined and appeared muriform (Abdel-Wahab and Nagahama 2012). The asexual stage of *H. japonica* is similar to other marine helicoid species such as *Cirrenalia*, *Cumulospora* and *Zalerion*.

##### Molecular evaluation.

In this study, *Halosarpheia* sensu stricto clade includes five species: *H. fibrosa*, *H. trullifera*, *H. japonica*, *H. australiensis* and *H. unicellularis* (Fig. 4). Four *Halosarpheia* species (*H. bentotensis*, *H. culmiperda*, *H. minuta* and *H. phragmiticola*) lacks molecular data, and need to be recollected, isolated and sequenced to determine if they belong in the genus and in the Halosphaeriaceae. Divergence-time estimates place the crown of *Halosarpheia* at ~40 MYA (95% CI: 10–160 MYA), supporting its recognition as a distinct and long-standing lineage within the family (Fig. 5).

#### Halosarpheia fibrosa

Taxon classificationFungiMicroascalesHalosphaeriaceae

Kohlm. & E. Kohlm., Trans. Brit. Mycol. Soc. 68 (2): 208 (1977)

01E3DB7C-2E94-5FA6-82C8-3832D4CC25F5

Index Fungorum: IF314854

Figs 3e, 21

##### Description.

Saprobic on submerged wood in aquatic habitats. **Sexual morph. *Ascomata*** 380–440 µm high, 340–450 µm diam., obpyriform to subglobose, immersed or partly immersed, ostiolate, papillate, coriaceous, brown to black, immersed part lighter-colored than the exposed neck and top. ***Necks*** 140–530 μm long, 130–200 μm diam. at the base, 90–110 μm diam. at the apex, periphysate. ***Peridium*** 40–60 μm thick, two-layered, forming a *textura angularis*, outer layer composed of 2–3 layers of polygonal or subglobose dark or light brown cells with large lumina, inner layer composed of 7–10 layers of elongated, hyaline cells with narrow lumina. ***Asci*** 160–220 × 34–46 µm, 8-spored, clavate, persistent, unitunicate, thick-walled below the apex, pedicellate, without apical apparatuses. ***Catenophyses*** present. ***Ascospores*** 32–44 × 18–24 µm, hyaline, 1-septate, broad ellipsoidal, not constricted at the septum, with bipolar appendages. ***Appendages*** 3–5 µm thick, 6–8(–11) µm diam., uncoils into a fine thread in water, 0.5–1 µm diam. **Asexual morph**. Undetermined.

##### Notes.

*Halosarpheia fibrosa* is most morphologically similar to *H. trullifera*, and both have ellipsoidal, 1-septate ascospores with broadly rounded apices and two polar appendages that uncoil in water to form long filaments. However, *H. fibrosa* differs from *H. trullifera* in having larger ascospores (Kohlmeyer 1963; Kohlmeyer and Kohlmeyer 1977).

**Figure 21.**
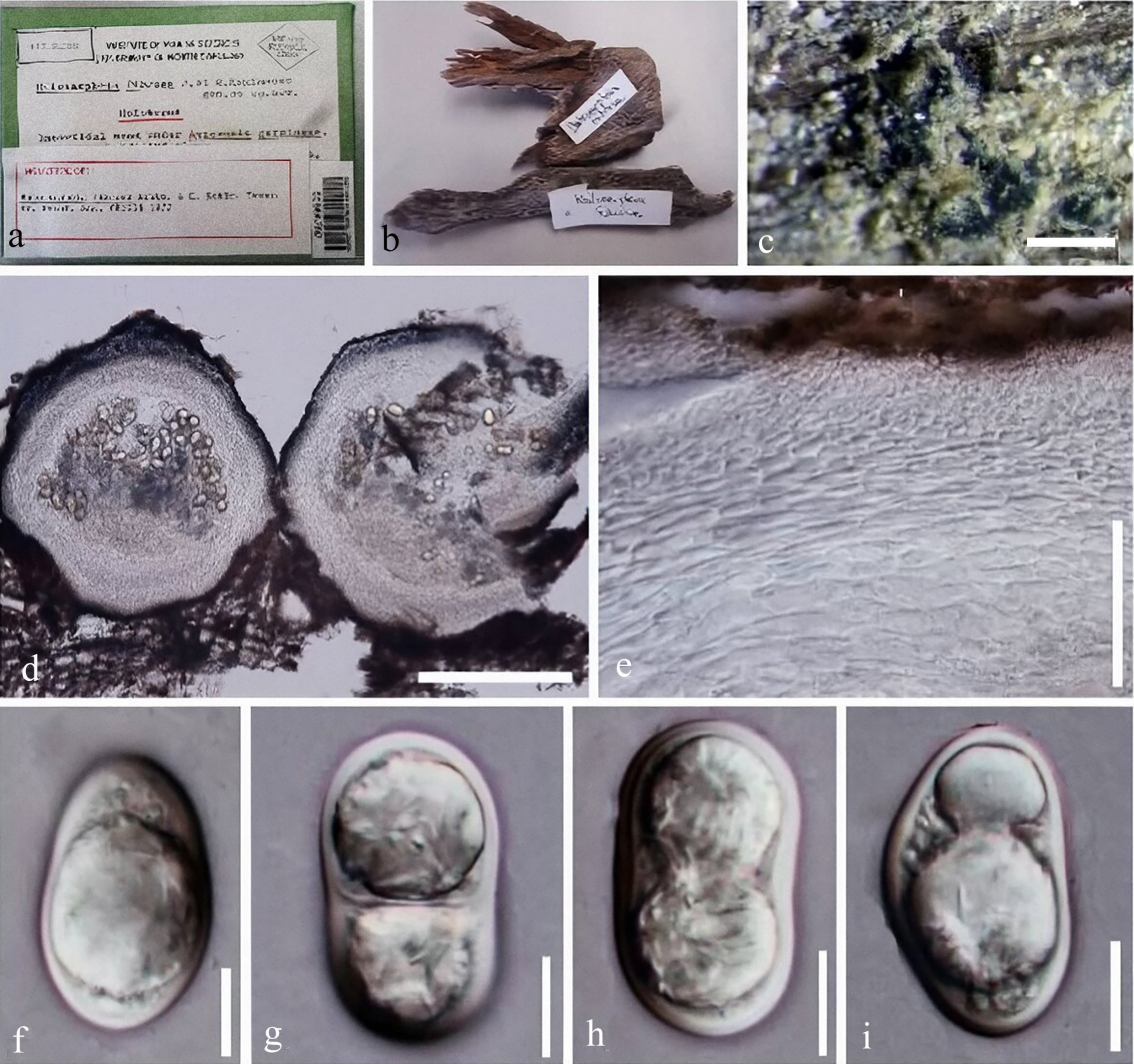
*Halosarpheia fibrosa* (NY 00966760, holotype). **a, b**. Herbarium material of *Halosarpheia fibrosa*; **c**. Ascomata on host surface; **d**. Section through ascomata; **e**. Peridium; **f–i**. Ascospores. Scale bars: 500 μm (**c**); 100 μm (**d, e**); 20 μm (**f–i**).

#### Halosarpheia trullifera

Taxon classificationFungiMicroascalesHalosphaeriaceae

(Kohlm.) E.B.G. Jones, S.T. Moss & Cuomo, Trans. Brit. Mycol. Soc. 80 (2): 200 (1983)

4963D356-3B28-5FDD-B34E-6E3AAEE4546A

Index Fungorum: IF108901

Remispora trullifera Kohlm., Nova Hedwigia 6: 321 (1963). Basionym.
Halosphaeria trullifera (Kohlm.) Kohlm., Canad. J. Bot. 50 (9): 1957 (1972). Synonym.

##### Notes.

This species has a chequered history, initially introduced as *Remispora trullifera* (Kohlmeyer 1963) but subsequently transferred to *Halosphaeria* based on his interpretation of ascospore appendage morphology (Kohlmeyer 1972). *Halosarpheia trullifera* has ascospore with a polar cap-like appendages closely adpressed to the spore wall but following release from the ascus become fibrillar as in *Halosarpheia*. Jones et al. (1983b) undertook an SEM study of the ascospore appendages which appeared to be composed of amorphous material, which on exposure to seawater unraveled to form thread-like polar appendages. Based on these observations, Jones et al. (1983b) transferred the species to *Halosarpheia*.

##### Distribution.

Australia, Denmark, Portugal.

#### Halosarpheia australiensis

Taxon classificationFungiMicroascalesHalosphaeriaceae

(K.D. Hyde) McKeown, S.T. Moss & E.B.G. Jones comb. nov.

E972272D-13F4-5462-AFAE-832B7268C1CC

Index Fungorum: IF904208

Tunicatispora australiensis K.D. Hyde, Austral. Syst. Bot. 3: 712 (1990). Basionym.

##### Description.

Saprobic on decaying intertidal wood of *Avicennia marina*. **Sexual morph. *Ascomata*** 240–360 µm high, 220–320 µm diam., globose to subglobose, immersed or semi-immersed, hyaline below, hyaline to light brown on exposed upper surface, ostiolate, papillate, thin-walled, membranous, solitary or gregarious. ***Necks*** 180–220 × 45–64 µm, light brown, periphysate. ***Peridium*** up to 50 µm thick, composed of several layers of cells, pigmented, rounded and thick-walled towards the outside, elongate and thinner-walled towards the venter. ***Centrum*** breaking up into catenophyses. ***Asci*** 70–102 × 22–26 µm, ovoid to clavate, thin-walled, unitunicate, 8-spored, deliquescing early. ***Ascospores*** 18–25 × 10–14 µm, 2–3-seriate, 1-septate, ellipsoidal, hyaline, thick-walled, and appendaged. ***Appendages*** of two types: a thin skin-like sheath which surrounds the ascospore with openings at each pole; and a viscous pad arising from these openings at each spore pole. The pad is attached to the spore apex by a single thread but the pad was seldom seen to unravel. **Asexual morph**. Undetermined.

##### Material examined.

Australia, • Cannons Creek Coastal Reserve (33°38'S, 151°23'E), near Melbourne, on decaying intertidal wood of *Avicennia marina*, February 1988, K.D. Hyde (BRIP 16783, holotype); Denmark, • On driftwood (*Quercus* sp.) collected at Srandegaard Dyrehave, Jutland.

##### Notes.

McKeown et al. (1990) undertook a detailed ultrastructural study (SEM and TEM) of ascospore appendage development showing that the viscous pad described by Hyde (1990b) was composed of a tightly coiled thread. *Halosarpheia australiensis* can easily be confused with *H. trullifera* as both polar appendages that unfurl to form thread-like structures which protrude through a layer enveloping the ascospore (McKeown et al. 1990). The outer layer, termed a sheath in *H. trullifera* (Jones et al. 1983b; Farrant 1986) is thinner than that of *H. australiensis*. *Halosarpheia trullifera* differs from *T. australiensis* in that it has a thickened ascus apex and the polar appendage arise from a collar-like structure. In *T. australiensis*, the appendage protrudes through a pore in the exosporium and is formed prior to the development of the mesosporium (McKeown et al. 1990). Morphologically *H. australiensis* has all the attributes of the genus *Halosarpheia*, and we therefore transfer it to that genus.

##### Distribution.

Australia, Denmark

### *Halosphaeriopsis* T.W. Johnson

The genus *Halosphaeriopsis* was introduced by Johnson (1958) to accommodate a species transferred from the genus *Halosphaeria*, with a single species *Halosphaeriopsis mediosetigera*. Earlier collections of this fungus led researchers to believe that there was a large spore variant known as *Halosphaeria mediosetigera* var. *grandispora*, which was reduced as a synonym of *H. mediosetigera* by Johnson and Sparrow (1961). In this monograph a second species, *Halosphaeriopsis alopallonella*, is included as the asexual fungus *Trichocladium alopallonella*, which has been shown to group with *H. mediosetigera* in a well-supported clade (Calabon et al. 2023, Fig. 4).

#### Halosphaeriopsis

Taxon classificationFungiMicroascalesHalosphaeriaceae

T.W. Johnson, J. Elisha Mitchell Sci. Soc. 74: 44 (1958)

01A19C85-19AE-5206-8F68-5F29B16FD5B7

Index Fungorum: IF2210

##### Description.

Saprobic marine fungus. **Sexual morph. *Ascomata*** subglobose or ellipsoidal, immersed or partly immersed, ostiolate, papillate, subcarbonaceous, black, solitary or gregarious. ***Peridium*** composed of five to eight (to twenty) layers of irregular, polygonal, thick-walled cells, forming a *textura angularis*, merging into the pseudoparenchyma of the venter. ***Papillae*** or necks are conical or subcylindrical, centric or eccentric, dark; ostiolar canal filled with a small-celled pseudoparenchyma, composed of thin-walled, large cells filling venter of young ascocarps, early deliquescing. ***Asci*** 8-spored, clavate, pedunculate, unitunicate, thin-walled, aphysoclastic, without apical apparatus, early deliquescing; developing at the base of the ascocarp venter on a small-celled ascogenous tissue. ***Ascospores*** ellipsoidal or subfusiform, 1-septate, not or slightly constricted at the septum, hyaline, appendaged; at both ends with a small cap that may invert; around the septum three (rarely four) crescent-shaped, rigid, attenuate appendages, obliquely attached to the septum, developing by fragmentation of the exospore. **Asexual morph. *Conidiophores*** absent or short, zero- to four-septate, simple, formed laterally on hyphae, hyaline to light brown or fuscous. ***Conidia*** (blastoconidia) are clavate, ovoid or obpyriform, two- to five-septate, constricted at the septa, straight or slightly curved, increasing in diameter from base to apex, formed singly on the conidiophores; apical cells subglobose, dark brown; basal cells conical or subcylindrical, subhyaline to light brown or fuscous. ***Chlamydospores*** catenulate, dark brown; chains up to 35-celled, terminal, rarely intercalary, simple; cells increasing in diameter from base to apex, globose or ellipsoidal (Description adapted from Kohlmeyer and Kohlmeyer 1979).

##### Type species.

*Halosphaeriopsis mediosetigera* (Cribb & J.W. Cribb) T.W. Johnson, J. Elisha Mitchell Sci. Soc. 74: 44 (1958).

##### Notes.

The two *Halosphaeriopsis* species are saprobic fungi, capable of colonizing a wide variety of plant substrates and algae, also having a wide distribution around the world, displaying a cosmopolitan distribution range (Kohlmeyer and Kohlmeyer 1979).

##### Molecular evaluation.

Both *Halosphaeriopsis* species formed a monophyletic clade outside the major clade of *Corollospora* spp. with weak support (Fig. 4). Divergence-time analysis indicates that *Halosphaeriopsis* separated from *Corollospora* during the Triassic–Jurassic (~243 MYA, 95% CI: 95–624 MYA), supporting its recognition as an independent lineage despite limited molecular resolution (Fig. 5).

#### Halosphaeriopsis mediosetigera

Taxon classificationFungiMicroascalesHalosphaeriaceae

(Cribb & J.W. Cribb) T.W. Johnson, J. Elisha Mitchell Sci. Soc. 74: 44 (1958)

BF9EC682-5B35-5924-BF7C-CC6DC82DACA1

Index Fungorum: IF298100

Fig. 1f

Halosphaeria mediosetigera Cribb & J.W. Cribb, Pap. Dept. Bot. Univ. Queensland 3 (12): 100 (1956). Basionym.
Culcitalna achraspora Meyers & R.T. Moore, Amer. J. Bot. 47 (5): 348 (1960). Synonym.
Trichocladium achrasporum (Meyers & R.T. Moore) M. Dixon, Trans. Brit. Mycol. Soc. 51 (1): 163 (1968).
Trichocladium achrasporum (Meyers & R.T. Moore) M. Dixon ex Shearer & J.L. Crane, Mycologia 63 (2): 244 (1971).

##### Description.

Saprobic marine fungus. **Sexual morph. *Ascomata*** 138–590 µm, subglobose or ellipsoidal, immersed or partly immersed, ostiolate, papillate, subcarbonaceous, black, solitary or gregarious. ***Peridium*** 12–32 µm thick, composed of five to eight (to twenty) layers of irregular, polygonal, thick-walled cells, forming a *textura angularis*, merging into the pseudoparenchyma of the venter. ***Papillae*** or necks 35–320 µm long, 35–80(–140) µm in diam., conical or subcylindrical, centric or eccentric, dark, ostiolar canal periphysate. ***Asci*** 62.5–168(–200) × 20–48 µm, 8-spored, clavate, pedunculate, unitunicate, thin-walled, aphysoclastic. ***Ascospores*** 24–44.5 × 8–17 (–20) µm (excluding appendages), ellipsoidal or subfusiform, 1-septate, not or slightly constricted at the septum, hyaline, appendaged; at both ends with a small cap that may invert; around the septum three (rarely four) crescent-shaped, rigid, attenuate appendages, (12–)24.5–40 µm long, up to 1.3 µm in diam. at the base, obliquely attached to the septum, developing by fragmentation of the exospore. **Asexual morph. *Conidiophores*** absent or short, zero- to four-septate, simple, formed laterally on hyphae, hyaline to light brown or fuscous. ***Conidia*** (blastoconidia) (15–)20–34(–45) × (8–) 10–24 µm clavate, ovoid or obpyriform, two- to five-septate, constricted at the septa, straight or slightly curved, increasing in diameter from base to apex, formed singly on the conidiophores; apical cells subglobose, dark brown; basal cells conical or subcylindrical, subhyaline to light brown or fuscous. ***Chlamydospores*** are 6–16, catenulate µm, dark brown; chains up to 500 µm, long chains up to 35-celled, terminal, rarely intercalary, simple; cells increasing in diameter from base to apex, 7.5–19 µm long, globose or ellipsoidal (Description adapted from Kohlmeyer and Kohlmeyer 1979).

##### Notes.

*Halosphaeriopsis mediosetigera* is an interesting species as it was the first marine ascomycete to be sequenced and its position in the Halosphaeriaceae confirmed (Spatafora and Blackwell 1994). It also was the first to be studied at the SEM level by Moss and Jones (1977) who demonstrated that the polar and equatorial appendages were formed by fragmentation of an exosporic sheath. Shearer and Crane (1977) showed that the asexual morph of *H. mediosetigera* was *Trichocladium achrasporum* based on cultural observations. Subsequently, Shearer (1986) opined that the sexual morph of this species did not produce the asexual morph in culture, and no isolate of the asexual state had been shown to produce the sexual stage.

##### Distribution.

Australia, Canada, Bulgaria, Denmark, France, Iceland, Germany, Japan, Liberia, Mexico, Portugal, Senegal, Spain, UK (England, Scotland, Wales), USA (California, Florida, Maryland, Washington).

#### Halosphaeriopsis alopallonella

Taxon classificationFungiMicroascalesHalosphaeriaceae

(Meyers & R.T. Moore) Devadatha, Day. & E.B.G. Jones comb. nov.

CE8D53B7-6693-5E7C-AECE-49D26E89C304

Index Fungorum: IF849677

Humicola alopallonella Meyers & R.T. Moore, Amer. J. Bot. 47 (5): 346 (1960). Basionym.
Trichocladium alopallonellum (Meyers & R.T. Moore) Kohlm. & Volkm.-Kohlm., Mycotaxon 53: 352 (1995). Synonymy.
Halosphaeriopsis alopallonella (Meyers & R.T. Moore) Devadatha & E.B.G. Jones, Bot. Mar. 66 (4): 224 (2023).

##### Description.

Saprobic on wood. **Sexual morph**. Undetermined. **Asexual morph**. Hyphomycetous. ***Hyphae*** hyaline to light brown, septate, branched. ***Conidiophores*** micronematous, mononematous, resemble non-specialized short lateral vegetative hyphae or conidiophore indistinct, conidia developing directly on hyphae. ***Conidia*** 14–24 μm × 7–11 μm, solitary, thick-walled, smooth, straight, 2–4 septate, strongly constricted at the septa, apical cell large subglobose, ellipsoidal, ovoidal to obpyriform, fuscous, reddish-brown to dark brown, basal cell smaller, obconical, light brown (Description based on Pang et al. 2011).

##### Material examined.

Usa, • *Tilia americana*, wood, in seawater; Culture isolated by S.P. Meyers, No. F-123, Sept. 1960 (Holotype); USA Florida, Marine Laboratory, Univ. Miami.

##### Notes.

In Fig. 4, *Trichocladium alopallonella* formed a monophyletic group with *H. mediosetigera* with strong branch support. Therefore, *T. alopallonella* is transferred to *Halosphaeriopsis* in this study.

##### Distribution.

Australia, Bahamas, Bermuda, Cameron, Canada, France, Germany, India, Italy, Ivory Coast, Japan, Liberia, Norway, UK (England, Scotland, Wales), USA (Alaska, Hawaii, Washington), Yemen.

### *Havispora* K.L. Pang & Vrijmoed

A monotypic genus described from coastal driftwood collected at Longyearbyen inside the Arctic circle.

#### Havispora

Taxon classificationFungiMicroascalesHalosphaeriaceae

K.L. Pang & Vrijmoed, Mycologia 100 (2): 293 (2008)

BBACD972-31A0-53AA-8762-D4015592620E

Index Fungorum: IF506744

##### Description.

Saprobic on driftwood. **Sexual morph. *Ascomata*** black, ellipsoidal or subglobose, coriaceous. ***Periphyses*** absent. ***Peridium*** dark-colored, 2-layered, outer stratum of cells ex *textura angularis*, inner stratum of elongated cells. ***Asci*** clavate, thin-walled, unitunicate, 8-spored, persistent, developing at the base of ascoma venter. ***Catenophyses*** present. ***Ascospores*** ellipsoidal, thin-walled, with one appendage at each pole and four equatorial appendages of equal dimension. ***Appendages*** one tuft at polar and four tufts at equatorial positions, string-like composed of intertwining strands which split in seawater. **Asexual morph**. Undetermined.

##### Type species.

*Havispora longyearbyenensis* K.L. Pang & Vrijmoed, Mycologia 100(2): 293 (2008).

##### Notes.

*Havispora* (*H. longyearbyenensis*) belongs to the Halosphaeriaceae with the presence of catenophyses, persistent asci with no apical structure and hyaline ascospores with appendages. Its ascospore appendage morphology and ontogeny are unique, i.e. string-like appendages composed of intertwining strands at polar and equatorial positions (Pang et al. 2008b). Among the genera in the Halosphaeriaceae, polar and equatorial appendages are also present in the following genera: *Corollospora*, *Halosphaeria*, *Halosphaeriopsis*, *Marinospora*, *Nautosphaeria*, *Nereiospora*, *Ocostaspora* and *Sablicola*. Appendages in *Havispora* are string-like and thus differ from *Nereiospora* species which have central brown cells and in spore measurements. Transmission electron micrographs showed that appendages of *N. comata* are attached to the spore wall by a pad, which is not present in *Havispora* (Jones and Moss 1980). Also, *Nautosphaeria*, *Nereiospora* and *Sablicola* lack catenophyses.

##### Molecular evaluation.

A partial 28S rDNA sequence of *H. longyearbyenensis* is available in the GenBank, and formed a weakly supported sister relationship with *Nereiospora comata* and *N. cristata* phylogenetically (Sakayaroj et al. 2011). In this study Fig. 4, *H. longyearbyenensis* formed a weakly supported clade with *Nereiospora* spp. and *Magnisphaera* spp. Further collections are required to establish its relationship with other taxa in the Halosphaeriaceae. Divergence-time analysis places this relationship in the ~182 MYA; 95% CI: 57.7–516.3 MYA, but the limited data highlight the need for further collections and multi-gene sequencing to clarify its position in the Halosphaeriaceae (Fig. 5).

#### Havispora longyearbyenensis

Taxon classificationFungiMicroascalesHalosphaeriaceae

K.L. Pang & Vrijmoed, Mycologia 100(2): 293 (2008)

608000D2-395F-57A2-82D3-C95025E0FAAD

Index Fungorum: IF506745

##### Description.

Saprobic on driftwood. **Sexual morph. *Ascomata*** 442–(616)–787 × 306–(422)–607 µm, solitary, black, ellipsoidal to subglobose, immersed, coriaceous. ***Peridium*** 29–(37)–44 µm, dark-colored, 2-layered, outer stratum with 5–7 rows of cells of *textura angularis*, inner stratum with 3–4 rows of elongated cells. ***Necks*** 43–(61)–80 × 33–(69)–88 µm, periphyses absent. ***Asci*** 88–(103) –114 × 20–(25)–33 µm, clavate, pedunculate, thin-walled, unitunicate, 8-spored, persistent, developing at the base of the ascoma venter. ***Catenophyses*** present, 105–(132)–165 × 5–(6)–8 µm. ***Ascospores*** 24–(30)–36 × 8–(11)–14 µm, ellipsoidal, hyaline, thin-walled, 3-septate constricted at the septa, with tufts of appendages. ***Appendages*** 4–(10)–14 µm long, at one polar tuft, and four tufts in an equatorial position, string-like composed of intertwining strands that split in seawater. **Asexual morph**. Undetermined.

##### Notes.

*Havispora longyearbyenensis* is saprobic on driftwood (Pang et al. 2008b).

##### Distribution.

Sweden, Norway.

### *Iwilsoniella* E.B.G. Jones

The genus *Iwilsoniella* was introduced by Jones (1991) to accommodate the species *Iwilsoniella rotunda* and the genus remains monophyletic.

#### Iwilsoniella

Taxon classificationFungiMicroascalesHalosphaeriaceae

E.B.G. Jones, Syst. Ascomycetum 10: 8 (1991)

0C8EA5F5-1BE7-532B-BAF1-812A03D2AF3B

Index Fungorum: IF25574

##### Description.

Saprobic on wood. **Sexual morph. *Ascomata*** subglobose, dark brown, membranous, superficial or immersed, solitary or gregarious, ostiolate, papillate. ***Peridium*** is composed of two types of cells, an outer layer of rectangular, thick-walled and melanized cells, the inner layer has thin-walled, pseudoparenchymatous cells. ***Necks*** (100-)250–300 × 50–60 µm, periphyses absent. ***Asci*** 55–75 × 20–28 µm, thin-walled, unitunicate, 8-spored, clavate, deliquescing early, pedicellate. ***Catenophyses*** absent. ***Ascospores*** 12–18 µm, hyaline, round, thin-walled, one-celled with no appendages. **Asexual morph**. Undetermined (Adapted from https://www.marinefungi.org).

##### Type species.

*Iwilsoniella rotunda* E.B.G. Jones, Syst. Ascomycetum 10: 8 (1991).

##### Notes.

As a marine fungus in the Halosphaeriaceae, *Iwilsoniella* (*I. rotunda*) is unusual as it lacks ascospores with elaborate appendages and resembles the genera *Anisostagma*, *Moana* and *Thalassogena*, which have spherical-ellipsoidal hyaline, unicellular ascospores. *Anisostagma* differs from *Iwilsoniella* in the dark brown color of the ascomata, as well as possessing catenophyses and periphyses. *Moana* is distinguished from *Iwilsoniella* by possessing cream colored ascomata, and ascospores with a single polar, uncoiling strap-like appendage. *Iwilsoniella* has deliquescing asci without apical structures, which differentiates it from *Thalassogena*. Despite lacking appendages, the spore wall appears to have a membranous material that peels off the spore wall (Hyde et al. 1989; Jones 1991).

##### Molecular evaluation.

There are currently no available sequences and cultures. Recollecting and sequencing are required to determine the placement of *Iwilsoniella* in the Halosphaeriaceae.

#### Iwilsoniella rotunda

Taxon classificationFungiMicroascalesHalosphaeriaceae

E.B.G. Jones, Syst. Ascomycetum 10: 8 (1991)

7934FC26-7485-5D4C-B9FD-23E870F21FC4

Index Fungorum: IF128203

Fig. 22

##### Description.

Saprobic on wood. **Sexual morph. *Ascomata*** 250–400 µm high × 200–230 µm diam., subglobose, dark brown, membranous, superficial or immersed, solitary or gregarious, ostiolate, papillate. ***Peridium*** 10–24 µm thick, composed of two types of cells, an outer layer of rectangular, thick-walled and melanized cells, the inner layer has thin-walled, pseudoparenchymatous cells. ***Periphyses*** absent. ***Asci*** thin walled, unitunicate, 8-spored, clavate, deliquescing early, pedicellate. ***Catenophyses*** absent. ***Ascospores*** are hyaline, round, thin-walled, one-celled with no appendages. **Asexual morph**. Undetermined (Adapted from https://www.marinefungi.org).

**Figure 22.**
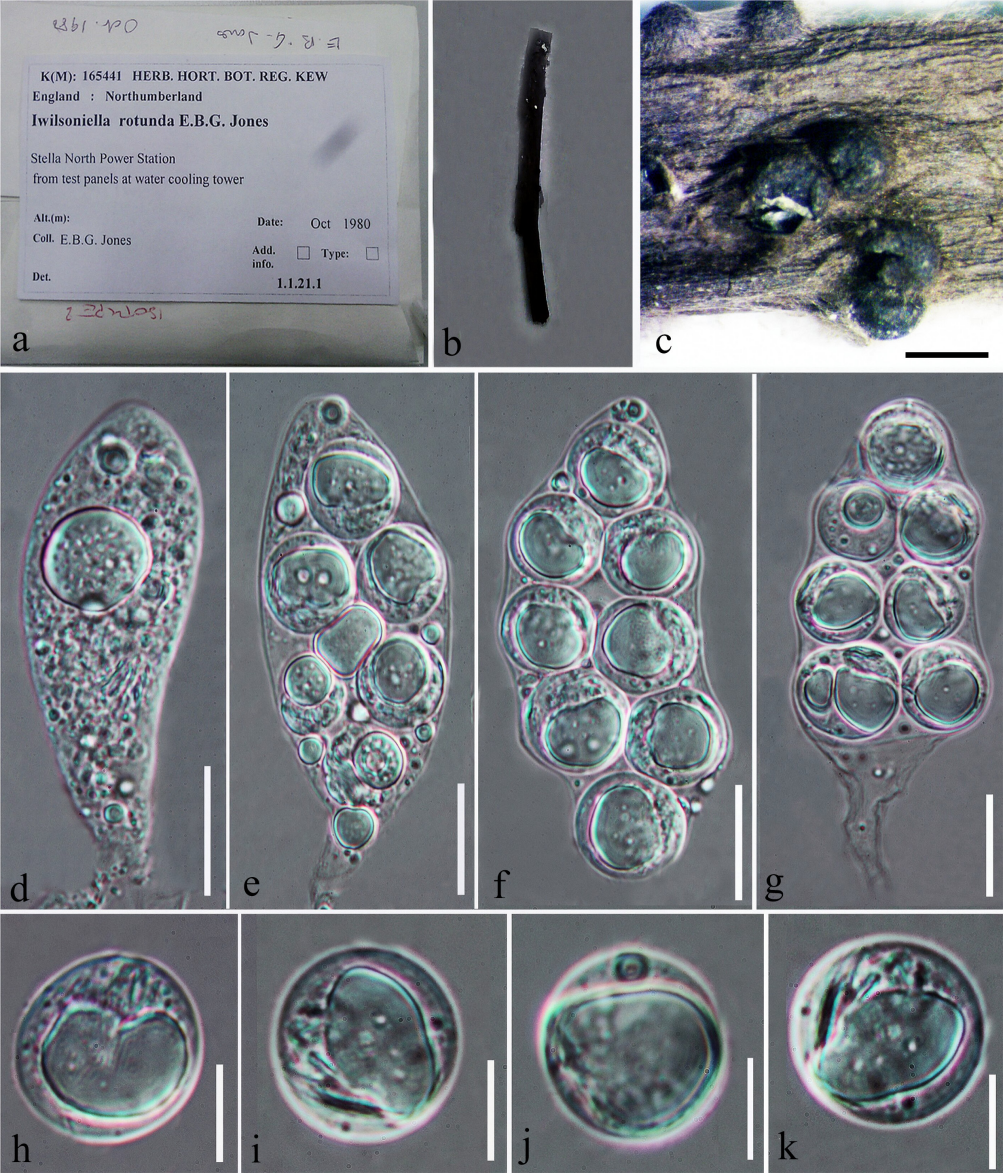
*Iwilsoniella rotunda* (K(M): 165441, holotype). **a, b**. Herbarium material; **c**. Ascomata; **d–g**. Asci; **h–k**. Ascospores. Scale bars: 500 μm (**d, e**); 20 μm (**f–g**); 10 μm (**h–k**).

##### Notes.

*Iwilsoniella rotunda* is a saprobic fungus, capable of tolerating brackish to full seawater, initially found on wood test blocks that were exposed in a cooling tower at the Stella North power station (Jones 1991).

### *Jinshana* K.L. Pang, S.Y. Guo & E.B.G. Jones

The genus *Jinshana* was introduced by Manawasinghe et al. (2024) to accommodate the species *Jinshana tangtangiae*.

#### Jinshana

Taxon classificationFungiMicroascalesHalosphaeriaceae

K.L. Pang, M.W.L. Chiang & E.B.G. Jones, Fungal Diversity 130: 118 (2024)

CCB2D923-A621-5DD3-A99C-562D2E74D6E2

Index Fungorum: IF902396

##### Description.

Saprobic on trapped wood on a rocky shore. **Sexual morph. *Ascomata*** superficial, globose to subglobose, solitary to gregarious, coriaceous, yellow to brown, ostiolate, papillate. ***Necks*** short, cylindrical, white to pale yellow. ***Periphyses*** not observed. ***Peridium*** comprising an outer stratum (5–8 layers) of hyaline cells of *textura angularis* with large lumina and an inner stratum (3–4 layers) of hyaline and elongated cells. ***Catenophyses*** present. ***Asci*** 8-spored, unitunicate, thin-walled, clavate with a flattened apex, pedunculate. ***Ascospores*** ellipsoidal, 1-septate, with one large oil globule in each cell, not constricted at the septum, thick-walled, hyaline, appendaged. ***Appendages*** bipolar, initially adpressed to the ascospore wall, unravelling in sea water to form a long thin filament. **Asexual morph**. Undetermined.

##### Type species.

*Jinshana tangtangiae* K.L. Pang, M.W.L. Chiang & E.B.G. Jones, Fungal Diversity 130: 118 (2024).

##### Notes.

Morphologically, *Jinshana* is related to the complex with unfurling ascospore appendages, including *Aniptodera*, *Halosarpheia* and *Phaeonectriella*. However, *Jinshana* did not group with any genera in the Halosphaeriaceae with unfurling ascospore appendages, but with the genus *Qarounispora* (Fig. 4). *Jinshana* differs in having clavate asci with a flattened apex, and the presence of bipolar ascospore appendages which unravel in seawater, in contrast to the broad clavate asci without an apical apparatus and broadly ellipsoidal ascospores with a unipolar appendage which swells in water to form an irregular amorphous structure in *Qarounispora* (Nourel-Din et al. 2022).

##### Molecular evaluation.

*Jinshana tangtangiae* and *Qarounispora grandiappendiculata* formed a highly supported clade, adjacent to *Nimbospora*, *Phaeonectriella* and the new genus *Neohalosarpheia*. Divergence-time analysis indicates that this lineage originated in the ~4 MYA; 95% CI: 1.3–10.5 MYA, supporting its recognition as a distinct evolutionary branch within the Halosphaeriaceae (Fig. 5).

#### Jinshana tangtangiae

Taxon classificationFungiMicroascalesHalosphaeriaceae

K.L. Pang, M.W.L. Chiang & E.B.G. Jones, Fungal Diversity 130: 118 (2024)

9072566B-05A1-5654-8DCA-A6513253FF9D

Index Fungorum: IF902397

##### Description.

Saprobic on trapped wood on a rocky shore. ***Sexual morph*. *Ascomata*** 181–245 μm high, 181–266 μm diam., superficial, globose to subglobose, solitary to gregarious, coriaceous, yellow to brown, ostiolate, papillate. ***Necks*** short, 53–117 μm long, 32–64 μm diam., cylindrical, yellow to brown. ***Periphyses*** not observed. ***Peridium*** 21–53 μm, comprising an outer stratum (5–8 layers) of hyaline cells of *textura angularis* with large lumina and an inner stratum (3–4 layers) of hyaline and elongated cells. ***Catenophyses*** present. ***Asci*** 105–142 × 19–28 μm, 8-spored, unitunicate, thin-walled, clavate with a flattened apex, pedunculate. ***Ascospores*** 21–28 × 10–11 μm, ellipsoidal, 1-septate, with one large oil globule in each cell, not constricted at the septum, thick-walled, hyaline, appendaged. ***Appendages*** bipolar, initially adpressed to the ascospore wall, unravelling in sea water to form a long thin filament. **Asexual morph**. Undetermined.

##### Material examined.

Taiwan, • Jin-Shan (New Taipei City, Taiwan). On a piece of unidentified trapped wood, 1 June 2022, S.Y. Guo and K.L. Pang, F0036020 (National Museum of Natural Science, Taiwan, holotype), dried wood.

##### Notes.

*Jinshana tangtangiae* is saprobic on unidentified trapped wood at a rocky shore in Taiwan.

### *Kitesporella* Jheng & K.L. Pang

A monotypic genus described from coastal trapped wood between rocks in northern Taiwan (Pang and Jheng 2012a).

#### Kitesporella

Taxon classificationFungiMicroascalesHalosphaeriaceae

Jheng & K.L. Pang, Bot. Mar. 55: 462 (2012)

D1F1F938-453D-593E-889E-A00599288DF7

Index Fungorum: IF563899

##### Description.

Saprobic on wood. **Sexual morph. *Ascomata*** globose to subglobose, hyaline, solitary, immersed, coriaceous, ostiolate. ***Necks*** short, periphyses absent. ***Peridium*** thick, two layers, composed of an inner layer of elongate cells with large lumina and an outer layer of cells of *textura angularis*. ***Asci*** clavate, unitunicate, thin-walled, short pedunculate, 8-spored, persistent or semi-persistent, developing at the base of ascoma venter. ***Catenophyses*** absent. ***Ascospores*** kite or rhomboid shape, unicellular, hyaline, smooth, thin-walled. **Asexual morph**. Undetermined.

##### Type species.

*Kitesporella keelungensis* Jheng & K.L. Pang, Bot. Mar. 55: 462 (2012).

##### Notes.

*Kitesporella* belongs to the Halosphaeriaceae with its perithecioid ascomata, pseudoparenchymatous centrum, clavate asci, and hyaline ascospores and its marine occurrence (Pang and Jheng 2012a). The kite-/rhomboid-shape ascospores of *K. keelungensis* are unique in the family and can be differentiated from the closely related genera (*Anisostagma*, *Iwilsoniella*, *Thalassogena*) which possess globose/subglobose ascospores (Pang and Jheng 2012a).

##### Molecular evaluation.

No sequences are available for *K. keelungensis* as ascospores did not germinate in culture for molecular analysis and direct extraction of DNA from spore mass also did not yield any DNA (Pang and Jheng 2012a). The species needs to be recollected, isolated and sequenced.

#### Kitesporella keelungensis

Taxon classificationFungiMicroascalesHalosphaeriaceae

Jheng & K.L. Pang, Bot. Mar. 55: 462 (2012)

69245A73-D692-56AB-9E41-1A1893FCFBA9

Index Fungorum: IF563900

##### Description.

Saprobic on wood. **Sexual morph. *Ascomata*** 182–(244)–271 × 318–(360)–382 μm, pyriform, globose to subglobose, hyaline, solitary, immersed, coriaceous, ostiolate. ***Necks*** 44 × 162 μm, periphyses absent. ***Peridium*** 15–(52)–85 μm thick, composed of an inner layer of elongate cells with large lumina and an outer layer of cells of *textura angularis*. ***Asci*** 75–(99)–144 × 27–(35)–45 μm, clavate, unitunicate, thin-walled, short pedunculate, 8-spored, persistent or semi-persistent, developing at the base of ascoma venter. ***Catenophyses*** absent. ***Ascospores*** 16–(20)–26 × 11–(13)–15 μm, kite or rhomboid shape, unicellular, hyaline, smooth, thin-walled. **Asexual morph**. Undetermined.

##### Notes.

*Kitesporella keelungensis* is saprobic on wood.

##### Distribution.

Taiwan.

### *Kochiella* Sakayaroj, K.L. Pang & E.B.G. Jones

Kohlmeyer (1981) introduced *Remispora crispa* from material collected in Martinique (French Antilles), also from Hawaii and Liberia, on dead tree wood, and driftwood. Molecular sequence data showed clearly that the genus *Remispora* was polyphyletic and the genus *Kochiella* was proposed to accommodate *R. crispa* (Sakayaroj et al. 2011).

#### Kochiella

Taxon classificationFungiMicroascalesHalosphaeriaceae

Sakayaroj, K.L. Pang & E.B.G. Jones, Fungal Diversity 46: 96 (2011)

2920E278-7F1B-51EA-BD2A-628286435AFA

Index Fungorum: IF518769

##### Description.

Saprobic on wood. **Sexual morph. *Ascomata*** subglobose to ellipsoidal, superficial to immersed, ostiolate, papillate, periphysate, coriaceous, thin-walled, hyaline to cream colored. ***Asci*** ellipsoidal to clavate, thin-walled, deliquescing at maturity. ***Ascospores*** 1-septate, hyaline, appendages pleomorphic. **Asexual morph**. Undetermined.

##### Type species.

*Kochiella crispa* (Kohlm.) Sakay., K.L. Pang & E.B.G. Jones, Fungal Diversity 46: 96 (2011).

##### Notes.

*Kochiella* is saprobic, described from submerged and intertidal wood (Kohlmeyer and Kohlmeyer 1979).

##### Molecular evaluation.

*Kochiella crispa* formed a weakly clade with *Ocostaspora apilongissima* (Fig. 4). Divergence-time estimates place this association in the Miocene (~16 MYA, 95% CI: 4–72 MYA) (Fig. 5).

#### Kochiella crispa

Taxon classificationFungiMicroascalesHalosphaeriaceae

(Kohlm.) Sakay., K.L. Pang & E.B.G. Jones, Fungal Diversity 46: 96 (2011)

52D214A1-49BA-5165-BE1A-F4C7EE014C6B

Index Fungorum: IF518770

Fig. 23c, d

Remispora crispa Kohlm., Canad. J. Bot. 59 (7): 1317 (1981). Basionym.

##### Description.

Saprobic on wood and other cellulosic materials. **Sexual morph. *Ascomata*** subglobose to ellipsoidal, superficial to immersed, ostiolate, papillate, periphysate, coriaceous, thin-walled, hyaline to cream colored, solitary. ***Peridium*** composed of 4 to 5 layers of flat, thin-walled cells, merging into the pseudoparenchyma of the center. ***Necks*** cylindrical; ostiolar canal with periphyses. ***Paraphyses*** absent; center of immature ascomata filled with a hyaline, thin-walled deliquescing pseudoparenchyma. ***Asci*** 8-spored, ellipsoidal to clavate, short stipitate, unitunicate, thin-walled, dissolving at spore maturity. ***Ascospores*** ellipsoidal, 1-septate, slightly or not constricted at the septum, hyaline, at first at both apices with a wide cap that extends along one side to the middle of each cell. ***Appendages*** initially sub-gelatinous, upon release into the water becoming detached from the side of the spore, remaining attached to the spore apices; the lower part of the appendages swells and parallel fibers become apparent; fibers are straight near the point of attachment, but become wavy above; developed appendages are at first yoke-like, later scoop-shaped when they become detached from the lateral spore wall and invert. **Asexual morph**. not observed.

**Figure 23.**
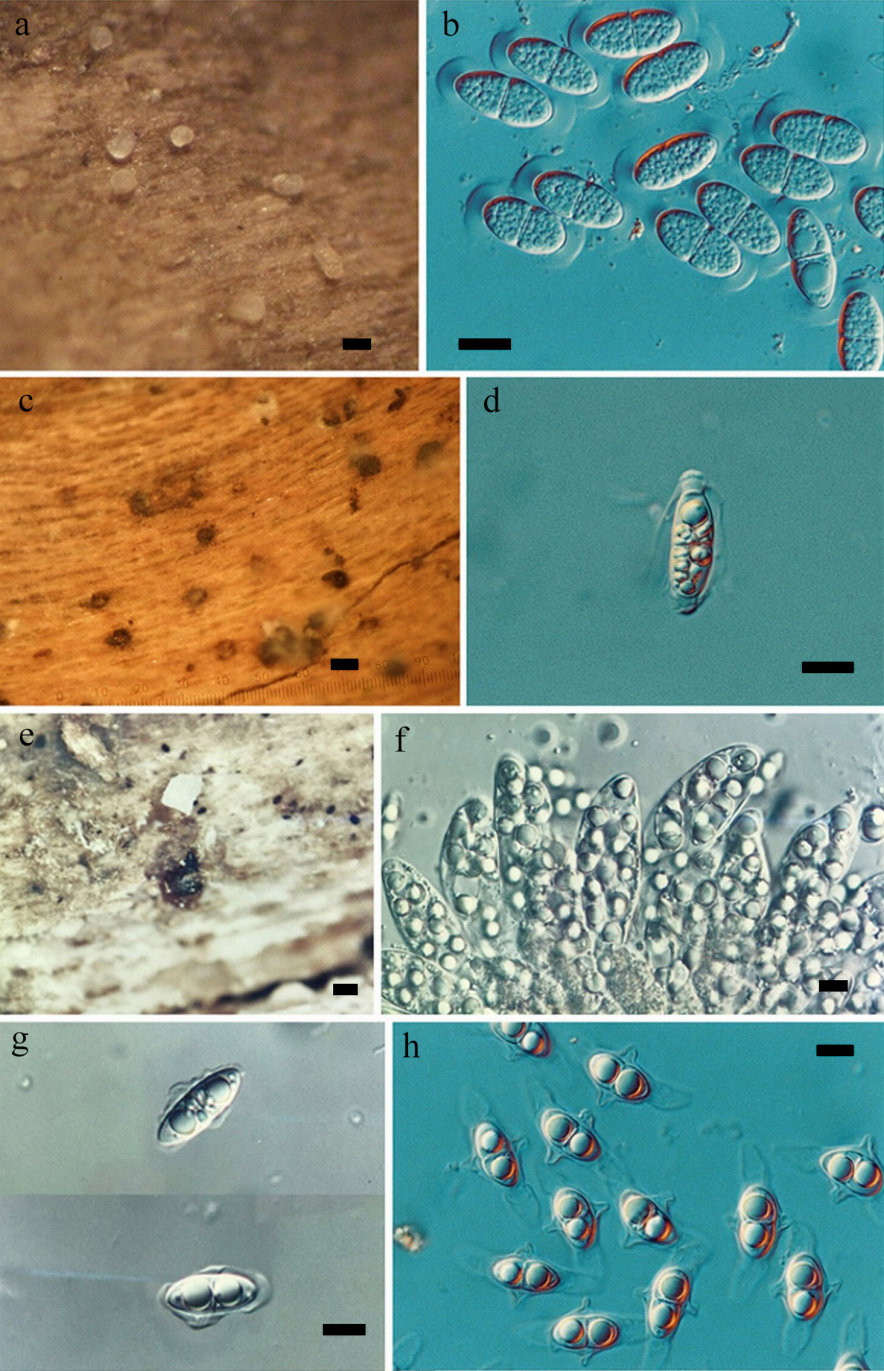
*Tubakiella galerita*. **a**. Immersed ascomata with hyaline spore mass oozing on the wood surface; **b**. Ornamented ascospores full of oil globules with subglobose compact cap-like polar appendages. *Kochiella crispa*; **c**. Superficial black ascomata on the wood surface; **d**. Ascospore with wide cap polar appendages oriented to one side of the middle of the spore. *Morakotiella salina*; **e**. Superficial black ascomata; **f**. Cylindrical-clavate asci; **g**. Ascospores with tightly coiled appendages around the spores. *Toriella tubulifera*; **h**. Ascospores with polar end chamber and equatorial appendages. Scale bars: 200 μm (**a, c, e**); 10 μm (**b, d, f–h**).

##### Material examined.

Denmark, • unidentified intertidal wood (BCC33502, BCC33504, BCC33507, living culture).

##### Note.

Ascospores germinate readily on agar plates and grow slowly but do not sporulate in culture. It is not widely collected but reported frequently from Denmark and India.

##### Distribution.

Hawaii, Japan, Liberia, Martinique, Portugal, Taiwan.

### *Lautisporopsis* E.B.G. Jones, Yusoff & S.T. Moss

This genus was introduced to accommodate *Ceriosporopsis circumvestita* as a study of its ascospore appendage ontogeny and development differed from that of the type species *Ceriosporopsis halima* (Yusoff et al. 1994a).

#### Lautisporopsis

Taxon classificationFungiMicroascalesHalosphaeriaceae

E.B.G. Jones, Yusoff & S.T. Moss, Mycotaxon 67: 1 (1998)

FA25ADB8-9E66-5D61-831D-9B5A177C1CC4

Index Fungorum: IF27443

##### Description.

Saprobic on driftwood and intertidal wood. **Sexual morph. *Ascomata*** subglobose, rarely globose, immersed or superficial, pale to dark brown, coriaceous, solitary or gregarious. ***Necks*** papillate with periphyses. ***Catenophyses*** absent. ***Asci*** 8-spored, clavate, pedunculate, unitunicate, thin-walled, deliquescing early. ***Ascospores*** ellipsoidal, 1-septate, hyaline, enveloped by a mucilaginous sheath, which is mesosporial in origin, and forming irregular polar and equatorial appendages surrounded by an episporium. Exosporium absent (Adapted from Yusoff et al. 1994a). **Asexual morph**. Undetermined.

##### Type species.

*Lautisporopsis circumvestita* (Kohlm.) E.B.G. Jones, Yusoff and S.T. Moss, in Jones, Mycotaxon 67: 1 (1998).

##### Notes.

*Halosphaeria circumvestita* was introduced by Kohlmeyer (1960), but transferred to *Ceriosporopsis* by Kohlmeyer (1972) as he regarded that many marine ascomycetes lacked clear generic limits. Subsequently, Jones and Moss (1978) and Jones et al. (1983a) undertook a major revision of genera in the Halosphaeriaceae by studying ascospore appendage ontogeny and development at the TEM and SEM level. This resulted in the introduction of *Lautisporopsis* for the species *Ceriosporopsis circumvestita* as ascospore appendage ontogeny and morphology was totally different. In *L. circumvestita* ascospores are enveloped in a mucilaginous sheath, which is mesosporial in origin, and forming irregular polar and equatorial appendages (Yusoff et al. 1994a). *Ceriosporopsis* species have only polar appendages.

##### Molecular evaluation.

There are no sequences available on any database, and no previous study evaluated this taxon phylogenetically, new collections, isolation and sequencing required.

#### Lautisporopsis circumvestita

Taxon classificationFungiMicroascalesHalosphaeriaceae

(Kohlm.) E.B.G. Jones, Yusoff & S.T. Moss, in Jones, Mycotaxon 67: 1 (1998)

28FBE82F-F5E8-5D3A-AE86-C61801889F78

Index Fungorum: IF363593

Index Fungorum: IF443610

Halosphaeria circumvestita Kohlm., Nova Hedwigia 2: 307 (1960). Basionym.
Ceriosporopsis circumvestita (Kohlm.) Kohlm., Canad. J. Bot. 50 (9): 1953 (1972). Synonym.

##### Description.

Saprobic on intertidal, lignocellulosic materials, test panels. **Sexual morph. *Ascomata*** 135–325 high, 150–450 µm diam., subglobose to ovoid, generally immersed, sometimes superficial, hyaline to dark brown, coriaceous, solitary. ***Peridium*** 12–17 µm wide, comprises two cell types, an outer zone of 4–5 layers of cells that are thick-walled and roundish to ellipsoidal, and an inner layer of elongate, hyaline cells. ***Necks*** usually short, 102–850 × 41–55 µm, with 4–5 layers of thick-walled cells, the canal lined with single-celled periphyses. ***Catenophyses*** not observed. ***Asci*** 62.5–87.5 × 15–25 µm, 8-spored, clavate, pedunculate, unitunicate, thin-walled, deliquescing early. ***Ascospores*** 14.5–25 × 9–13 µm, ellipsoidal, 1–septate, hyaline and appendaged. Appendages originate from the outer mesosporial layer, forming chambers, the equatorial and polar appendages being larger chambers and surrounded by the episporium. Polar and equatorial chambers contain no inclusions. **Asexual morph**. In culture (CMA), chlamydospores 3.5–7.5 µm in diam. are formed in chains (Adapted from Yusoff et al. 1994a).

##### Notes.

*Lautisporopsis circumvestita* is saprobic on intertidal wood and driftwood, found in temperate or arctic waters, not widely reported (Kohlmeyer and Kohlmeyer 1979).

##### Distribution.

Canada, Chile, Denmark, France, Iceland, Portugal, Sweden, UK, USA (Washington) Also at Grytviken, South Georgia, Antarctic where it occurred on a Ramin (*Gonystylus* sp.) test panel (Pugh and Jones 1986).

### *Lignincola* Höhnk

The genus *Lignincola* was introduced by Höhnk (1955) with *Lignincola laevis* as the type species, which was characterized by hyaline to dark ascomata, semi-persistent clavate to fusiform, thin-walled asci and 1-septate, hyaline, thin-walled ascospores without appendages. Kohlmeyer (1984) transferred *Gnomonia longirostris* to *Lignincola*. However, *L. longirostris* was later transferred to a new genus *Neptunella* based on phylogenetic analyses (Pang et al. 2003a). Another three species, *Lignincola conchicola*, *L. nypae*, *L. tropica*, were added to this genus (Kohlmeyer 1984; Hyde et al. 1999b; Liu et al. 2011). The genus was referred to the Halosphaeriaceae by Kohlmeyer and Kohlmeyer (1979) and this was confirmed by sequence data (Jones et al. 2009, 2017).

#### Lignincola

Taxon classificationFungiMicroascalesHalosphaeriaceae

Höhnk, Veröff. Inst. Meeresf. Bremerhaven 3: 216 (1955)

E41C69B7-C56F-59BB-9D6B-FA26242B3BCF

Index Fungorum: IF2864

##### Description.

Saprobic on submerged woody substrates in marine and freshwater habitats. **Sexual morph. *Ascomata*** perithecial, subglobose to ellipsoidal, solitary or gregarious, immersed to superficial, coriaceous, ostiolate, papillate, light brown to black, catenophyses deliquescing. ***Asci*** 8-spored, unitunicate, clavate or sub-fusiform, pedunculate, thin-walled, apiculate, persistent; developing at the base of the ascocarp venter; asci released from the ascoma through the ostiole. ***Ascospores*** ellipsoidal, 1-septate, hyaline, lacking appendages. **Asexual morph**. Undetermined.

##### Type species.

*Lignincola laevis* Höhnk, Veröff. Inst. Meeresf. Bremerhaven 3: 216 (1955).

##### Notes.

*Lignincola* species have been found in freshwater, brackish and marine habitats (Jones et al. 2017). *Lignincola* species have been recorded from decaying mangrove wood, intertidal wood, bark, roots of shoreline trees (e.g., balsa, bamboo, *Fagus sylvatica*, *Phragmites australis*, *Pinus massoniana*, *Hibiscus tiliaceus*, *Liquidambar styraciflua*, *Pachira aquatica*, *Rhizophora mangle*, *Pluchea fosbergi*, *Avicennia alba*, *A. germinans*, *A. marina*, *Laguncularia racemosa*), litter of *Agropyron pungens* and *Spartina townsendii*, petiole of intertidal *Nypa fruticans*, fronds of *Phoenix paludosa*, submerged branch with attached leaf of *Thalassia testudinum*, subtidal wood with shipworms and *Limnoria* under *Rhizophora mangle* (Kohlmeyer and Kohlmeyer 1979; Raabe et al. 1981; Kohlmeyer 1984; Hyde et al. 1999b; Lu et al. 2000; Pande 2008; Liu et al. 2011).

Jones et al. (2009) suggested that the genus *Lignincola* has only one unifying character, the hyaline 1-septate ascospores without appendages with considerable variation in the structure of the asci and ascospores. The type species *L. laevis* was characterized by black ascomata, persistent clavate to fusiform, thin-walled asci lacking an apical apparatus and have a distinct thimble-like apex (Höhnk 1955). Kohlmeyer (1984) introduced two other species, *L. tropica* and *L. longirostris* the latter initially described as *Gnomonia longirostris* (Cribb and Cribb 1956) with thick-walled, clavate asci with a refractive apical thickening and a pore and ascospores with a thin mucilaginous sheath (Kohlmeyer 1984; Pang et al. 2003a). Subsequently, Pang et al. (2003a) transferred the species to a new genus *Neptunella* based on morphological and molecular evidence.

*Lignincola tropica*, was described from mangrove wood in Mexico (Kohlmeyer 1984) with semi-persistent asci with a refractive apex with a pore and has larger ascospores (22–36 × 12–16 μm) than *L. laevis* (13–24 × 5–8 μm). Initially, the ascus was described as possessing an apical pore (Kohlmeyer 1984), but subsequently Kohlmeyer and Volkmann-Kohlmeyer (1988) emended the description, stating it lacked a pore. In addition, molecular studies show that it does not belong in *Lignincola* (Pang et al. 2003a). Jones et al. (2009) also considered that this species does not belong in the genus, but lack of a consensus on the morphology of its ascus prevents its transfer to a new genus. Sakayaroj et al. (2011) also showed that *L. tropica* grouped with *Halosarpheiasensu stricto*, *H. marina* and *Panorbis viscosus*. Sequence data in Figs 4, 5 confirm earlier findings, but the erection of a new genus is problematic: there are no sequences of the type material and Kohlmeyer (1984) did not illustrate the ascospores. Sequence PP7777, isolated from material collected at Boracay, Philippines, lacks supporting herbarium material and a photoplate. Therefore, further collections, isolation and sequencing is required to enable its transfer to a new genus.

*Lignincola nypae* was described from the intertidal petiole base of *Nypa fruticans* from Malaysia and has clavate asci with a refractive apical thickening with a pore and cylindrical, 13.5–20 × 3.5–4.5 μm ascospores (Hyde et al. 1999b). They considered the taxonomic status of *L. nypae* problematic because ascospores are cylindrical with an indistinct septum and asci are clavate with an apical pore. They suggested *Aniptodera* as an alternative genus for *L. nypae* but dismissed this as the ascospores lacked appendages. *Pseudolignincola siamensis* is morphologically similar to *L. nypae* in terms of ascus shape, but differs in the dimensions of ascomata, 1–4-septate ascospores and with an asexual morph (Jones et al. 2006).

*Lignincola conchicola* was described from submerged fronds of *Phoenix paludosa* with asci persistent and thin-walled and lacking an apical apparatus (Liu et al. 2011). Morphologically, this species is similar to the type species in ascal and ascospore morphology shape occurring on the adhesive pads of a marine invertebrate. *Lignincola laevis* also differs from *L. conchicola* by larger ascospores (12.5–24 × 5–8 μm) vs. (10.5–16 × 5–7 μm) and the ascomata have a neck (Höhnk 1955; Liu et al. 2011).

##### Molecular evaluation.

*Lignincola laevis* formed a moderately supported group with *Halosphaeria appendiculata* (Fig. 4). Divergence-time estimates place this association in the Miocene (~16 MYA, 95% CI: 4–72 MYA) (Fig. 5). However, no type material is available for the type species, and no sequence has been designated as the isotype of the genus. *Lignincola tropica* formed a separate branch outside the genus *Halosarpheia*. Further collections and molecular study are required to resolve the placement of *L. conchicola*, *L. tropica* and *L. nypae*.

#### Lignincola laevis

Taxon classificationFungiMicroascalesHalosphaeriaceae

Höhnk, Veröff. Inst. Meeresf. Bremerhaven 3: 216 (1955)

904C9170-87D3-54DA-B07A-72F584C350EB

Index Fungorum: IF299797

##### Description.

Saprobic on submerged wood in aquatic habitats. **Sexual morph. *Ascomata*** 125–250(–386) μm in diam., subglobose or ellipsoidal, immersed or superficial, ostiolate, papillate, coriaceous, hyaline, light brown, fuscous or blackish, solitary or gregarious. ***Necks*** up to 4 mm long, 25–40 μm in diam., cylindrical, centric or eccentric. ***Peridium*** 13–16 μm thick, composed of 2 to 5 layers of elongate, thick-walled cells with large lumina, forming a *textura angularis*. ***Catenophyses*** deliquescing. ***Asci*** 49–69 × 15–20 μm, 8-spored, clavate or sub-fusiform, short pedunculate, unitunicate, thin-walled, sometimes slightly thickened at the apex, without apical apparatuses, persistent. ***Ascospores*** (12.5–)16–24 × (5–)6–8 μm, irregularly biseriate, ellipsoidal, 1-septate, slightly constricted at the septum, hyaline, without appendages. **Asexual morph**. Undetermined.

##### Notes.

*Lignincola laevis* is a cosmopolitan fungus, reported from diverse habitats (freshwater, brackish, marine) and substrata (wood, marsh grasses) (Jones 1993; Sarma and Hyde 2001; Schmit and Shearer 2003; Jones and Pang 2012a).

Pang et al. (2013) concluded that *L. laevis* was a species complex comprising at least three different taxa with subtle morphological differences. Twenty-eight isolates from marine and brackish water collected on driftwood, mangrove and submerged test blocks and worldwide in distribution were sequenced and grouped in three major clades with pairwise distance of the ITS marker between 0% and 10.6%. However, no geographical assemblages could be detected.

### *Limacospora* Jørg. Koch & E.B.G. Jones

The genus *Limacospora* was introduced by Koch and Jones (1985) to accommodate the type species *Limacospora sundica* and originally assigned to the genus *Ceriosporopsis* (*Ceriosporopsis sundica*). Jones et al. (1995) undertook an ultrastructural (TEM, SEM) study of ascospore appendage development of *C. sundica* and showed that its morphology and structure was different from that of the type species of *Ceriosporopsis* (*C. halima*) and referred it to the new genus *Limacospora*.

#### Limacospora

Taxon classificationFungiMicroascalesHalosphaeriaceae

Jørg. Koch & E.B.G. Jones, Canad. J. Bot. 73 (7): 1011 (1995)

C1979D7D-2CFA-52AF-A919-1B85ED2FCE47

Index Fungorum: IF27610

##### Description.

Saprobic on intertidal wood. **Sexual morph. *Ascomata*** deeply embedded in wood, pale brown, long necks with no periphyses. ***Peridium*** two-layered: an outer layer of prismatic, nearly isodiametric cells and an inner layer of long, thin-walled often pointed cells. ***Asci*** clavate, tapering, pedunculate, unitunicate, early deliquescing, 8-spored in a hymenial layer at the base of the ascomata. Interthecial filaments present. ***Ascospores*** 1-septate, hyaline, surrounded by a mucilaginous sheath, bounded by the delimiting membrane, which is constricted at the septum. ***Appendages*** variable in morphology, initially in water elliptical and “sluglike” but becoming ovoid-oblong, then spore surrounded by fibrillar mucilage drawn into long polar threads. The ascospore wall comprises a mesosporium and an electron-dense episporium. The sheath arises as fibrillar extensions of the mesosporium through discontinuities in the episporium, initially highly condensed but swelling in water and rupturing the delimiting membrane. At maturity the ascospore is surrounded by interconnecting threads of mucilaginous material, with further extensions forming long fibrillar threads (Description based on Jones et al. 1995). **Asexual morph**. Undetermined.

##### Type species.

*Limacospora sundica* (Jørg. Koch & E.B.G. Jones) Jørg. Koch & E.B.G. Jones, Canad. J. Bot. 73 (7): 1013 (1995).

##### Notes.

This genus shares many characteristics with *Ceriosporopsis*, however it differs in the following ontological and ultrastructural characteristics: the polar appendage arises as an outgrowth of the mesosporium and is initially surrounded by an exosporial layer (Johnson et al. 1987). This is ruptured by the expansion of the polar appendage and forms a collar at the base of the polar appendage (Johnson et al. 1987; Yusoff et al. 1994a).

##### Molecular evaluation.

There is no available molecular data for this genus. *Limacospora sundica* needs to be recollected, isolated and sequenced to determine its relationship to other genera in the Halosphaeriaceae.

#### Limacospora sundica

Taxon classificationFungiMicroascalesHalosphaeriaceae

(Jørg. Koch & E.B.G. Jones) Jørg. Koch & E.B.G. Jones, Canad. J. Bot. 73 (7): 1013 (1995)

B8FD289B-2BC6-5E48-9D97-449D608AC58C

Index Fungorum: IF413404

Fig. 24

Ceriosporopsis sundica Jørg. Koch & E.B.G. Jones, Nordic J. Bot. 6 (3): 339 (1986). Basionym.

##### Description.

Saprobic on intertidal wood. **Sexual morph. *Ascomata*** gregarious, immersed, deep seated, subglobose-ellipsoid, 570–1080 µm broad, 270–675 µm high, ostiolate with a long neck extending to the surface of the wood. Outer layer of peridium consisting of hyaline, prismatic, nearly isodiametric cells, 16–20 µm thick at the base of the perithecium but thinning towards the apical region. Inner layer of the peridium composed of long, hyaline, thin-walled, often pointed cells, 20–40 × 8–10 µm, which are longer and broader towards the base of the ostiole. ***Necks*** cylindrical, brown-black, 46–76 µm in diam. and up to 1500 µm long. ***Asci*** 90–140 × 24–28 µm, clavate, tapering, pedunculate, unitunicate, thin-walled, early deliquescing, 8-spored, in different stages of maturity in a hymenium on a sub-hymenial layer formed as a broad, flat cushion or a flat cup in the lower part of the perithecial cavity. ***Paraphyses*** absent. ***Ascospores*** 19.0–21.1–24.0 × 8.0–8.9–10.0 µm, 1-septate, ellipsoidal or broadly fusiform, slightly constricted around septum, hyaline and appendaged. ***Ascospores*** enveloped in a granular sheath with polar appendages at either end which appear as outgrowths of the spore wall. Polar appendages initially short (circa 28 µm) but can be long and drawn out, sometimes forming fine threads reminiscent of *Halosarpheia* species. The sheath and polar appendages swell when placed in water to form a diffuse mass, are extremely sticky and may adhere spores to substrata. The sheath is slightly constricted at the septum with radiating striations extending outwards from the spore wall (Based on the description of *Ceriosporopsis sundica* in Jones and Koch 1985). **Asexual morph**. Undetermined.

**Figure 24.**
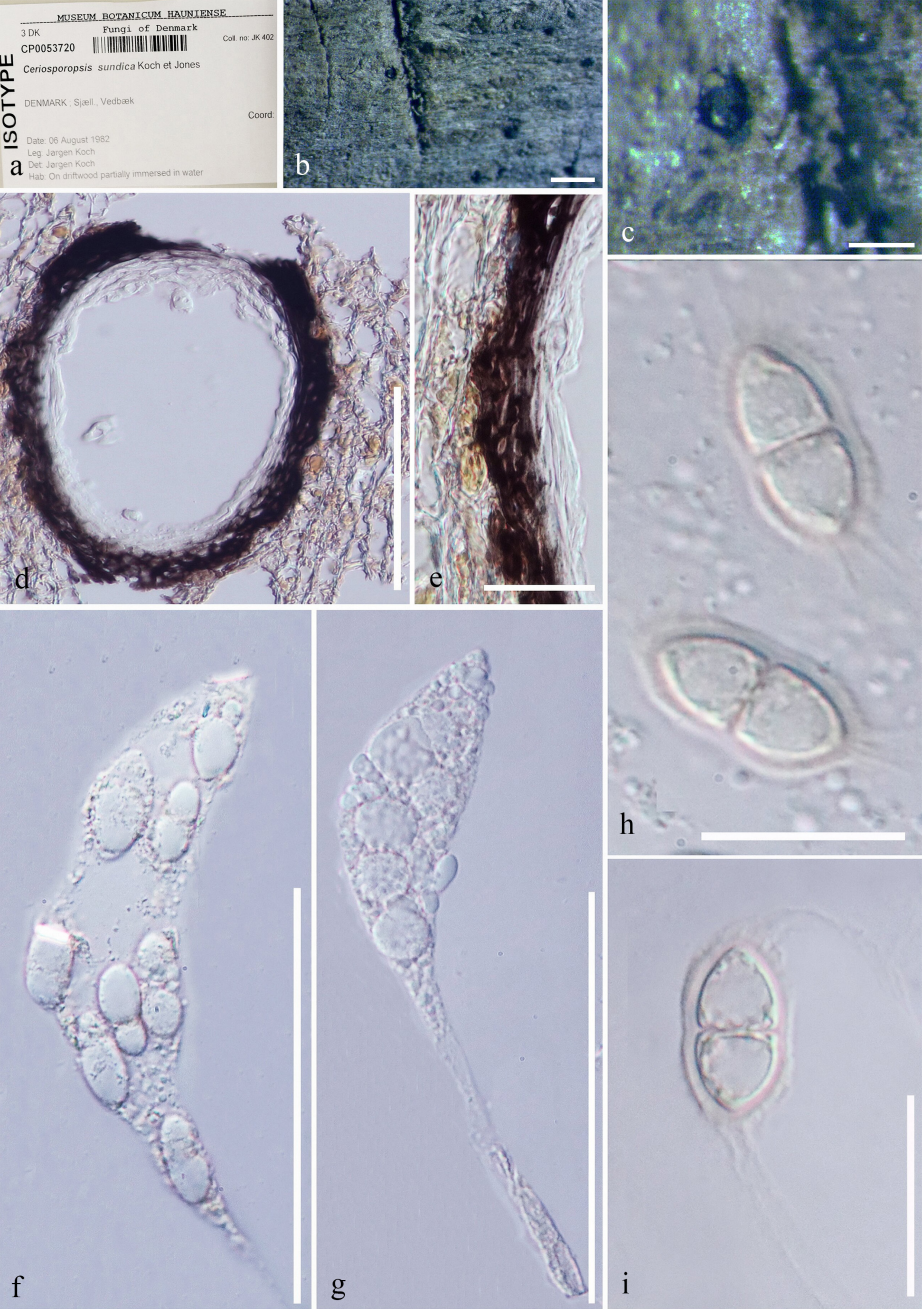
*Limacospora sundica* (CP0053720, isotype and micro-slides). **a**. Herbarium material; **b, c**. Ascomata on wood; **d**. Section through ascoma; **e**. Peridium; **f, g**. Asci; **h, i**. Ascospores. Scale bars: 200 µm (**d**); 20 µm (**e**); 100 µm (**f, g**); 20 µm (**h, i**).

##### Material examined.

Denmark, • Sealand, Vedbaek, on driftwood partially immersed in water, 6 Aug. 1982, Jorgen Koch, CP0053720 isotype and micro slides.

##### Notes.

This species is saprobic on wood and isolated from the coastal waters of Denmark (Jones et al. 1995).

##### Distribution.

Denmark, and India on intertidal and mangrove wood (Maria and Sridhar 2002, 2003; Nambiar and Raveendran 2007), however, this needs confirmation as it is originally known from temperate habitats (Koch and Jones 1985).

### *Luttrellia* Shearer

Shearer (1978) introduced the genus *Luttrellia* with *L. estuarina* as the type species from balsa wood submerged in the Patuxent River, an estuary of the Chesapeake Bay, Maryland, with water salinity of 0.5–8.5 PSU. However, Khokhr. and Gornostaĭ (1978) also used the epithet *Luttrellia* for the taxon *Luttrellia turcica* (Pleosporaceae = *Exserohilum turcicum*), unaware that this name was already occupied, and is therefore a *nomen rejectum*. Three further *Luttrellia* species, found on decorticated submerged wood were also introduced: *L. guttulata* (river in Panama), *L. halonata* (river in Ecuador) and *L. parvulospora* (Mississippi river) (Ferrer and Shearer 2007). Currently, four *Luttrellia* species are listed in Index Fungorum (May 2024), but only *L. estuarina* is a marine species. All assigned to the Halosphaeriaceae.

#### Luttrellia

Taxon classificationFungiMicroascalesHalosphaeriaceae

Shearer, Mycologia 70 (3): 692 (1978)

FD81E66D-BA01-5524-A996-EA4CEB5FB68E

Index Fungorum: IF2954

##### Description.

Saprobic on submerged balsa test panels. **Sexual morph. *Ascomata*** superficial, globose to subglobose membranous, becoming pale brown ostiolate, neck long, cylindrical hyaline and periphysate. ***Asci*** clavate to cylindrical, thin-walled and deliquescing. ***Catenophyses*** present. ***Ascospores*** hyaline, multiseptate and lacking appendages. The only genus in the Halosphaeriaceae with tetrasporic asci. **Asexual morph**. Undermined.

##### Type species.

*Luttrellia estuarina* Shearer, Mycologia 70 (3): 693 (1978).

##### Notes.

Shearer (1978) assigned *Luttrellia* to the Halosphaeriaceae as it compared with hyaline, unappendaged ascosporous genera in the family: *Lignincola*, *Nais* and *Aniptodera*, but chose not to emend the generic concept of *Nais*. *Luttrellia* species are saprobic on wood in freshwater and brackish water.

##### Molecular evaluation.

There are no DNA sequences available for any *Luttrellia* species. Recollection, isolation and sequencing are required to determine the taxonomic position of the four *Luttrellia* species in the Halosphaeriaceae.

#### Luttrellia estuarina

Taxon classificationFungiMicroascalesHalosphaeriaceae

Shearer, Mycologia 70 (3): 693 (1978)

F79C096A-8974-5563-8F92-135365DFD6EC

Index Fungorum: IF317023

##### Description.

Saprobic on wood. **Sexual morph. *Ascomata*** 277–327 × 416–455 µm, superficial, globose to subglobose membranous, becoming pale brown, ostiolate, neck long (248–822 × 35.6–40 µm), cylindrical hyaline and periphysate. ***Asci*** 103–158 × 12–26 µm, 4-spored, clavate to cylindrical, thin-walled and deliquescing. ***Catenophyses*** present. ***Ascospores*** 42–55 × 125.6–21.6 µm, hyaline, multiseptate and lacking appendages. **Asexual morph**. Undetermined.

##### Notes.

*Luttrellia estuarina* is a brackish water species on submerged wood. This species was infrequently collected, and its position in the Halosphaeriaceae requires verification.

### *Magnisphaera* J. Campbell, J.L. Anderson & Shearer

*Magnisphaera* was erected by Campbell et al. (2003) to accommodate two species; *Magnisphaera spartinae* that was phylogenetically shown as a clade distinct from *Halosarpheia* and a new species *Magnisphaera stevemossago*. *Magnisphaera* is characterized by black, globose to subglobose ascomata, ellipsoid, thin-walled, early deliquescent asci which lack an apical pore or apical apparatus and ellipsoidal to fusiform, hyaline ascospores (Campbell et al. 2003). *Magnisphaera* differs from *Halosarpheia* in having multi-septate ascospores with a rough ascospore wall (Jones 1962; Campbell et al. 2003). *Magnisphaera spartinae* was originally described by Jones (1962) from drift *Spartina townsendii*. *Matsusphaeria spartinae* was described by Pang et al. (2003b), unaware that Campbell et al. (2003) had transferred the species to *Magnisphaera* and thus has generic priority. The genus was referred to the Halosphaeriaceae by Shearer and Crane (1980) and this was confirmed by sequence data (Campbell et al. 2003; Pang et al. 2003b).

#### Magnisphaera

Taxon classificationFungiMicroascalesHalosphaeriaceae

J. Campbell, J.L. Anderson & Shearer, Mycologia 95 (3): 546 (2003)

45D9D862-59DF-5D28-9AD5-ADF98EA5394C

Index Fungorum: IF28707

##### Description.

Saprobic on submerged woody substrates in marine and freshwater habitats. **Sexual morph. *Ascomata*** perithecial, immersed, ostiolate, globose to subglobose, solitary or gregarious, dark brown to black, with a central neck, periphysate. ***Peridium*** two-layered, composed of about 8–10 large cells forming a *textura angularis*. ***Asci*** unitunicate, pedunculate, thin-walled, clavate, early deliquescent and lack an apical pore and apical apparatus. ***Ascospores*** ellipsoid to fusiform, phragmoseptate, hyaline, verruculose, constricted at the septa, uniguttulate in each of the central cells, with polar appendages. ***Appendages*** coiled into short, flat, hamate structures that unfurl to form long ribbon-like structures. **Asexual morph**. Undetermined.

##### Type species.

*Magnisphaera spartinae* (E.B.G. Jones) J. Campb., J.L. Anderson & Shearer, Mycologia 95 (3): 547 (2003).

##### Notes.

This genus is represented by two species, *M. spartinae* from marine environment and *M. stevemossago* from freshwater habitats (Campbell et al. 2003). *Magnisphaera* species are saprobic on *Spartina patens*, *Spartina alterniflora*, intertidal wood, balsa wood, *Agropyron junceiforme*, *Agropyron pungens*, *Spartina townsendii*, *Typha* sp., driftwood, herbaceous debris, and test panels (balsa, *Betula pubescens*) (Jones 1962; Campbell et al. 2003; https://www.marinefungi.org).

*Magnisphaera* is morphologically similar to *Halosarpheia*, but differs in having multi-septate ascospores with a rough ascospore wall (Jones 1962; Campbell et al. 2003; Pang et al. 2003b).

##### Molecular evaluation.

Sakayaroj et al. (2011) demonstrated that the genus grouped in the Halosphaeriaceae in a sister clade to *Havispora* and *Nereiospora*. A similar result was recorded in a further study of the Halosphaeriaceae by Jones et al. (2017). In this study (Fig. 4), *Magnisphaera* was monophyletic, forming a sister clade with *Nereiospora* spp. and *Havispora longyearbyenensis* with weak support. Divergence-time analysis places this relationship in the ~182 MYA; 95% CI: 57.7–516.3 MYA (Fig. 5).

#### Magnisphaera spartinae

Taxon classificationFungiMicroascalesHalosphaeriaceae

(E.B.G. Jones) J. Campb., J.L. Anderson & Shearer, Mycologia 95 (3): 547 (2003)

70446C1C-2202-5066-BDD4-BF0E4E001F71

Index Fungorum: IF373835

Haligena spartinae E.B.G. Jones, Trans. Brit. Mycol. Soc. 45: 245 (1962). Basionym.
Halosarpheia spartinae (E.B.G. Jones) Shearer & J.L. Crane, Bot. Mar. 23: 608 (1981). Synonymy.
Matsusphaeria spartinae (E.B.G. Jones) K.L. Pang & E.B.G. Jones, Nova Hedwigia 77 (1–2): 15 (2003).

##### Description.

Saprobic on *Spartina townsendii* and submerged wood in marine habitats. **Sexual morph. *Ascomata*** 190–310 µm high, 310–350 µm diam., solitary, globose, dark brown to black, coriaceous, immersed. ***Necks*** periphysate. ***Asci*** 124–214 × 32–60 µm, unitunicate, 8-spored, clavate, early deliquescing, pedicellate. ***Ascospores*** 60–76(–90) × 15–22 µm, hyaline, ellipsoidal, 2–7 (–8–9) septate, predominantly 5-septate, constricted at the septum, appendaged. ***Appendages*** bipolar, 7–18 × 1–2 µm, unfurling into fine ribbons in water. **Asexual morph**. Undetermined.

##### Notes.

Jones (1995) suggested that *M. spartinae* is different from *Halosarpheia* species based on the ontogeny of the appendages, that arise from a single polar pore in the ascospore wall as opposed to a pore field in *Halosarpheia* species (Alias et al. 2001; Baker et al. 2001). Campbell et al. (2003) introduced two species: *Magnisphaera spartinae* characterized by 60–90 × 15–22 µm, 2–9 septate, predominantly 5-septate ascospores; *M. stevemossago* has ascospores that are 3-septate, shorter and broader (45–64 × 16–31 µm) than those of *M. spartinae* (Campbell et al. 2003). In addition, *M. spartinae* is found from marine and brackish water habitats while *M. stevemossago* is known only from freshwater habitats (Calabon et al. 2022).

### *Marinospora* A.R. Caval.

This genus was introduced by Cavaliere (1966a) as *Ceriosporella*, but the name was illegitimate according to article 53.1 and renamed *Marinospora* (Cavaliere 1966b). The type species (as *Ceriosporopsis calyptrata*) was described by Kohlmeyer (1960), a second species *C. longissima* was introduced by Kohlmeyer (1962а), but later reduced to synonymy with *C. calyptrata*. Subsequently, Kohlmeyer (1972) referred both species to *Ceriosporopsis* as he considered that delineation based on appendage morphology to be insufficient and should be based on ontogeny and chemical composition. Kohlmeyer and Kohlmeyer (1979) retained the name *Ceriosporopsis calyptrata*. Johnson et al. (1984) examined the two species at the transmission and scanning electron microscope level and accepted the generic name *Marinospora*. Although *Ceriosporopsis* closely resembles *Marinospora*, spore appendage development is fundamentally different. Ascospores in *Marinospora* have distinct primary polar and equatorial appendages with cup-like structures at their apices, derived by fragmentation of an enveloping exosporic membrane. There are other differences in the structure of the ascomata (Johnson et al. 1984).

#### Marinospora

Taxon classificationFungiMicroascalesHalosphaeriaceae

A.R. Caval., Nova Hedwigia 11: 548 (1966)

6549CF5C-7601-56EF-9D27-CB904C2ECA0F

Index Fungorum: IF3002

##### Description.

Saprobic on submerged wood. **Sexual morph. *Ascomata*** are solitary or gregarious, carbonaceous to subcarbonaceous, ellipsoidal ovoidal to subglobose, immersed, ostiolate, papillate, periphysate and brown to black in color. ***Peridium*** composed of 7–30 cell layers, which are thick-walled with large lumens, the necks are long and periphysate. ***Pseudoparenchyma*** is thin-walled with polygonal or subglobose cells, forming catenophyses. ***Asci*** 8-spored, clavate, slightly apiculate, pedunculate, unitunicate, thin-walled, deliquescing early and form at the base of the ascocarp. ***Ascospores*** are broadly ellipsoidal, 1-septate, constricted at the septum, hyaline and appendaged. Both the polar and equatorial appendages are obclavate to subcylindrical, tapering, each primary appendage terminating in a cup-like secondary appendage. The ascospores may initially be enveloped by mucilage. The spore wall is composed of a two layered mesosporium, episporium and an outer exosporium which is ridged, or corrugated and covered to the outside by a thin exosporium which may deliquesce or peel away. The exosporium also gives rise to the cup-like “calyptra” at the tip of the polar and equatorial primary appendages, these primary appendages arise as outgrowths of the spore wall (Description adapted from Johnson et al. 1984). **Asexual morph**. Undetermined.

##### Type species.

*Marinospora calyptrata* (Kohlm.) A.R. Caval., Nova Hedwigia 11: 548 (1966).

##### Notes.

This genus closely resembles *Ceriosporopsis*, but differs in the following aspects: ascocarps are carbonaceous to subcarbonaceous, and ascocarp wall is composed of up to 30 layers; the asci are pedunculate and often apiculate; the ascospores have distinct primary polar and equatorial appendages with cup-like structures at their apices formed by fragmentation (or dissolution) of an enveloping exosporic membrane while the outer spore was corrugated or ridged. *Marinospora* has both ultrastructural and developmental differences from *Ceriosporopsis* (Johnson et al. 1984). Sequence data confirm that the genus is distinct from *Ceriosporopsis*, although closely related (Sakayaroj et al. 2004, 2011).

The original specimens were found on decayed driftwood (Cavaliere 1966a), with the type species classified as a temperate water species (Panebianco 1994).

##### Molecular evaluation.

In this study (Fig. 4) *Marinospora* species formed well supported clades with the genera *Toriella* and *Ondiniella*. *Marinospora longissima* was born on a long branch. There are no meaningful studies on the phylogeny of this genus, so further collections are warranted, including isolated individuals and a wider range of gene loci sequenced.

#### Marinospora calyptrata

Taxon classificationFungiMicroascalesHalosphaeriaceae

(Kohlm.) A.R. Caval., Nova Hedwigia 11: 548 (1966)

A4F636DC-97DD-563D-A5A6-E57876AC1B3D

Index Fungorum: IF333792

Fig. 3i

Ceriosporopsis calyptrata Kohlm., Nova Hedwigia 2: 301 (1960). Basionym.
Ceriosporella calyptrata (Kohlm.) A.R. Caval., Nova Hedwigia 10: 394 (1966). Synonym.

##### Description.

Saprobic on wood, driftwood, test panels. **Sexual morph. *Ascomata*** 295–605 µm high, 480–770 µm diam., ellipsoidal, subglobose, immersed often deep in the wood, ostiolate, papillate, subcarbonaceous or carbonaceous, brown to black, solitary or gregarious. ***Peridium*** 12–80 µm thick, composed of 7–30 layers of thick-walled cells, with large lumina merging with wood particles. ***Necks*** up to 1050 µm, wide, periphysate. ***Catenophyses*** present. ***Asci*** 104–190 × 22–37 µm, 8-spored, clavate, long pedicellate, unitunicate, thin-walled, early deliquescing, lacking an apical apparatus. ***Ascospores*** 30–36 × 9–19 µm, hyaline, broadly ellipsoidal, 1-septate, constricted at the septum, with apical and equatorial appendages, terminating in a cup-shaped caps, ends rounded, one large guttule per cell. ***Appendages*** gelatinous, surrounded by an exosporic membrane which breaks down, straight or curved. **Asexual morph**. Undermined (Adapted from Cavaliere 1977; Johnson et al. 1984).

##### Notes.

*Marinospora calyptrata* is widely distributed in temperate zones, on a wide variety of timbers: *Fagus sylvatica*, *Larix* sp., *Picea* sp. *Quercus* sp. and others.

Panebianco et al. (2002) examined the effect of pre-inoculation of balsa wood by the marine fungi *Ceriosporopsis halima*, *Corollospora maritima*, *Halosphaeriopsis mediosetigera* and *Marinospora calyptrata* and the subsequent colonization by other marine fungi over periods 2, 6, 9 and 15 months. In all cases, the climax/dominant fungus at 15 months was *M. calyptrata*, highlighting its ecological role in the succession of fungi on wood. Its success may be attributed to its ability to penetrate deeper into the wood (Panebianco et al. 2002).

##### Distribution.

Argentina, Belgium, Canada, Denmark, Germany, Italy, Portugal, Sweden, UK, and USA.

### *Moana* Kohlm. & Volkm.-Kohlm.

The genus *Moana* was introduced by Kohlmeyer and Volkman-Kohlmeyer (1989), with the type species *Moana turbinulata*.

#### Moana

Taxon classificationFungiMicroascalesHalosphaeriaceae

Kohlm. & Volkm.-Kohlm., Mycol. Res. 92 (4): 418 (1989)

54696EB6-25DA-5D93-9CC1-77522E946F8A

Index Fungorum: IF 25325

##### Description.

Saprobic on wood. **Sexual morph. *Ascomata*** are single, cream-colored, subglobose, immersed, ostiolate, periphysate, papillate, coriaceous. ***Peridium*** is thin, forming a *textura angularis*. ***Catenophyses*** develop from the thin-walled pseudoparenchyma. ***Asci*** 8-spored, clavate, pedunculate, rounded at their apex, without a pore, non-amyloid, thin-walled, unitunicate, more or less persistent, maturing successively on an ascogenous tissue at the bottom of the locule. ***Ascospores*** are subglobose, one-celled, hyaline, with a single, top-shaped appendage that unwinds in water to produce a long ribbon. **Asexual morph**. Undetermined.

##### Type species.

*Moana turbinulata* Kohlm. & Volkm.-Kohlm., Mycol. Res. 92 (4): 418 (1989).

##### Notes.

Among members of the Halosphaeriaceae, *Moana* bears a striking resemblance to *Thalassogena* (*T. sphaerica*), the latter has asci flat at the tip with a pore, while *M. turbinulata* has apically rounded, aporous asci. The ascospores in both species are almost identical, except for the long unfurling appendage in *M. turbinulata*.

##### Molecular evaluation.

*Moana turbinulata* grouped with *Gesasha peditatus* (Fig. 4) in highly supported clades, but have few morphological features in common. Divergence-time analysis indicates that this relationship dates back to the Paleogene (~5268 MYA, 95% CI: 1821.1–221.8136 MYA), suggesting an extended independent history despite their close phylogenetic affinity (Fig. 5). Further collections and genes are required to confirm phylogenetic placement of this genus.

#### Moana turbinulata

Taxon classificationFungiMicroascalesHalosphaeriaceae

Kohlm. & Volkm.-Kohlm., Mycol. Res. 92 (4): 418 (1989)

6CE9FC55-D8CB-5D05-9E8E-451E9F4E36D7

Index Fungorum: IF135942

Fig. 25

##### Description.

Saprobic on wood. **Sexual morph. *Ascomata*** 300–450 µm in diam., single, cream-colored, subglobose, immersed, ostiolate, papillate, coriaceous. ***Necks*** 250–800 µm long, 80–115 µm wide, the ostiolar canal filled with periphyses. ***Peridium*** 20–27 µm thick, composed of about six layers of polygonal cells forming a *textura angularis*, merging with the thin-walled pseudoparenchymatous cells. ***Catenophyses*** developing from the pseudoparenchyma that originally fills the central cavity. ***Asci*** 70–95 × 25–35 µm, 8-spored, clavate, pedunculate, apically rounded, without a pore, non-amyloid, thin-walled, unitunicate, more or less persistent, maturing successively on an ascogenous tissue at the bottom of the locule. ***Ascospores*** 12.5–16 µm, subglobose, one-celled, hyaline, containing one large lipid body, surrounded by numerous small non-lipoid droplets with a single, top-shaped appendage, 4–5 µm high, 6.5–8 µm diam., unfurling in water to form long tapering ribbons, 250–550 µm long, 1.5–3 µm wide, staining blue in methylene and cotton blue. **Asexual morph**. Undetermined.

**Figure 25.**
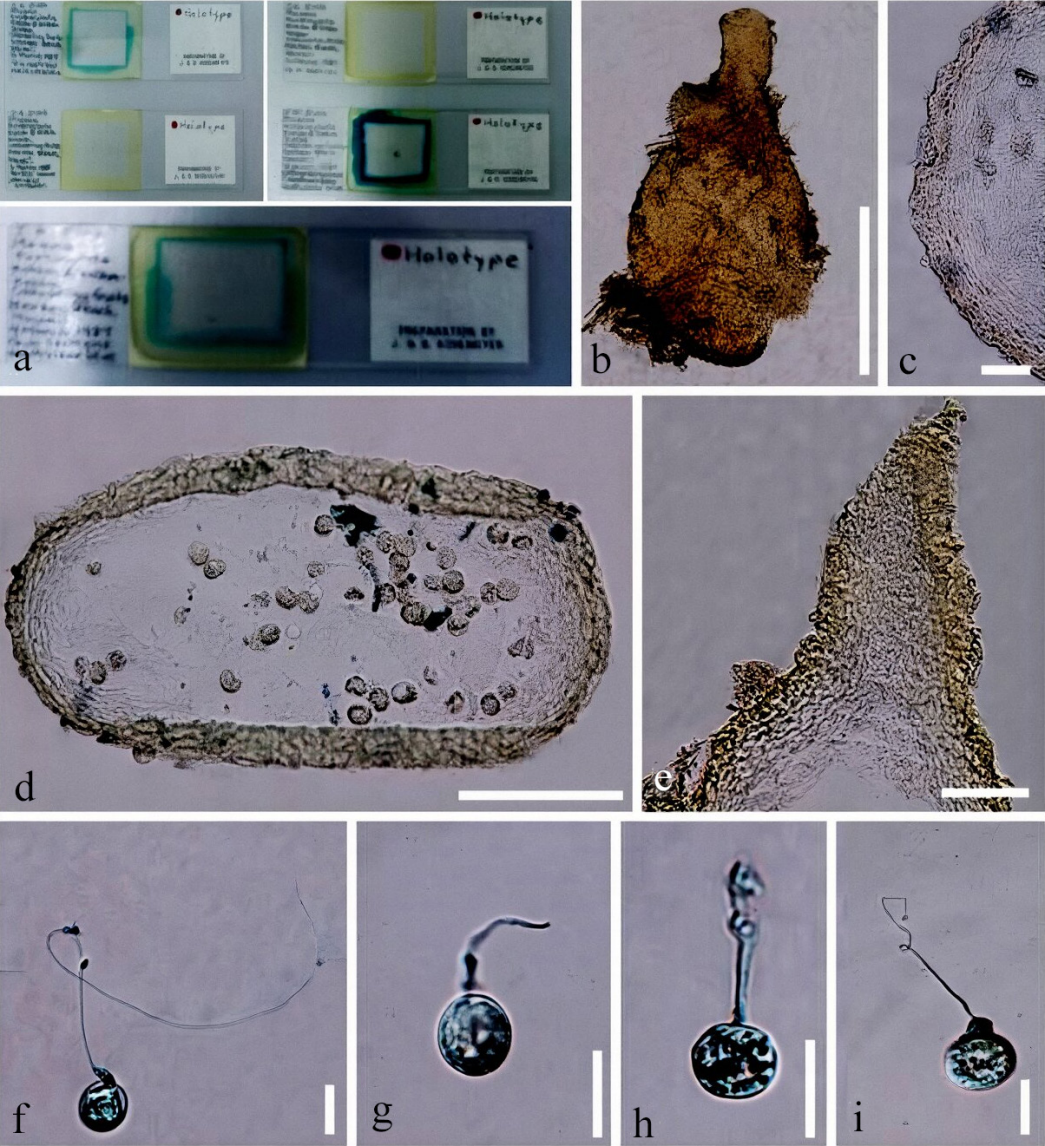
*Moana turbinulata* (NY347770, NY1347771, NY1347773, NY1347779 & NY1347782, holotypes). **a**. Herbarium material; **b**. Surface view of ascoma; **c**. Peridium; **d**. Section through ascoma; **e**. Ostiolar long neck; **f–i**. Ascospores. Scale bars: 100 µm (**b, d**); 50 µm (**c**); 20 µm (**e**); 10 µm (**f–i**).

##### Notes.

The single species of this genus is a saprobic obligate marine species, that was collected in Hawaii and Taiwan, on intertidal wood samples with other marine fungi.

### *Morakotiella* Sakay.

This fungus originally was identified as *Haligena salina* (Farrant and Jones 1986), but Sakayaroj et al. (2005) showed that it did not group with the type species *Haligena elaterophora*, forming a basal clade to the family in a phylogenetic analysis of the partial 28S rDNA. The genus *Morakotiella* was proposed to accommodate *H. salina*.

#### Morakotiella

Taxon classificationFungiMicroascalesHalosphaeriaceae

Sakay., Mycologia 97 (4): 806 (2005)

49C82F55-0E73-5AF0-8764-BE4EF0EED11E

Index Fungorum: IF28969

##### Description.

Saprobic on woody material, mangrove wood. **Sexual morph. *Ascomata*** globose to subglobose, immersed or superficial, ostiolate, black, perithecial wall coriaceous. ***Necks*** short, cylindrical and periphysate. ***Asci*** thin-walled, unitunicate, 8-spored, pedunculate, fusiform to clavate, deliquescing early. ***Catenophyses*** present or absent. ***Ascospores*** 1-septate, ellipsoidal, long thread-like polar appendages. **Asexual morph**. Undetermined.

##### Type species.

*Morakotiella salina* (C.A. Farrant & E.B.G. Jones) Sakayaroj, in Sakayaroj et al. (2005).

##### Notes.

*Morakotiella* differs from other genera in the Halosphaeriaceae with uncoiling appendages by the mode of attachment of the appendage to the ascospore wall; appendages are coiled around the spore, spoon-shaped at the point of attachment, channeled along its length; amorphous with distinct striations running the length of the appendage and arising as an outgrowth of the spore wall (at SEM level). *Morakotiella* is a monotypic genus typified by *Morakotiella salina*.

##### Molecular evaluation.

Phylogenetically *M. salina* is poorly resolved (Fig. 4) and further collections, isolation and sequencing of a wider range of gene loci.

#### Morakotiella salina

Taxon classificationFungiMicroascalesHalosphaeriaceae

(C.A. Farrant & E.B.G. Jones) Sakay., Mycologia 97 (4): 806 (2005)

B6EF087A-1995-5FD6-8392-981558290602

Index Fungorum: IF344553

Figs 23e–g, 26

Haligena salina C.A. Farrant & E.B.G. Jones, Bot. J. Linn. Soc. 93 (4): 406 (1986). Basionym.

##### Description.

Saprobic on woody material, drift and mangrove wood. **Sexual morph. *Ascomata*** immersed or partly immersed or superficial, globose, subglobose, ostiolate, black, perithecial wall coriaceous. ***Necks*** short, cylindrical and periphysate. ***Asci*** thin-walled, unitunicate, pedunculate, 8-spored, fusiform to clavate, deliquescing early. ***Catenophyses*** present or absent. ***Ascospores*** 1-septate, ellipsoidal, slightly constricted, hyaline, appendaged. ***Appendages*** polar, initially wrapped around the ascospore wall, later separating to form long filaments that are spoon-shaped at the place of attachment to the spore wall, attenuate, channeled. *Appendage* origin not determined but bounded by a thin electron-dense delimitating membrane attached to ascospore apices by fine threads. Appendage substructure fibrillar to amorphous, electron-dense. **Asexual morph**. Undermined.

**Figure 26.**
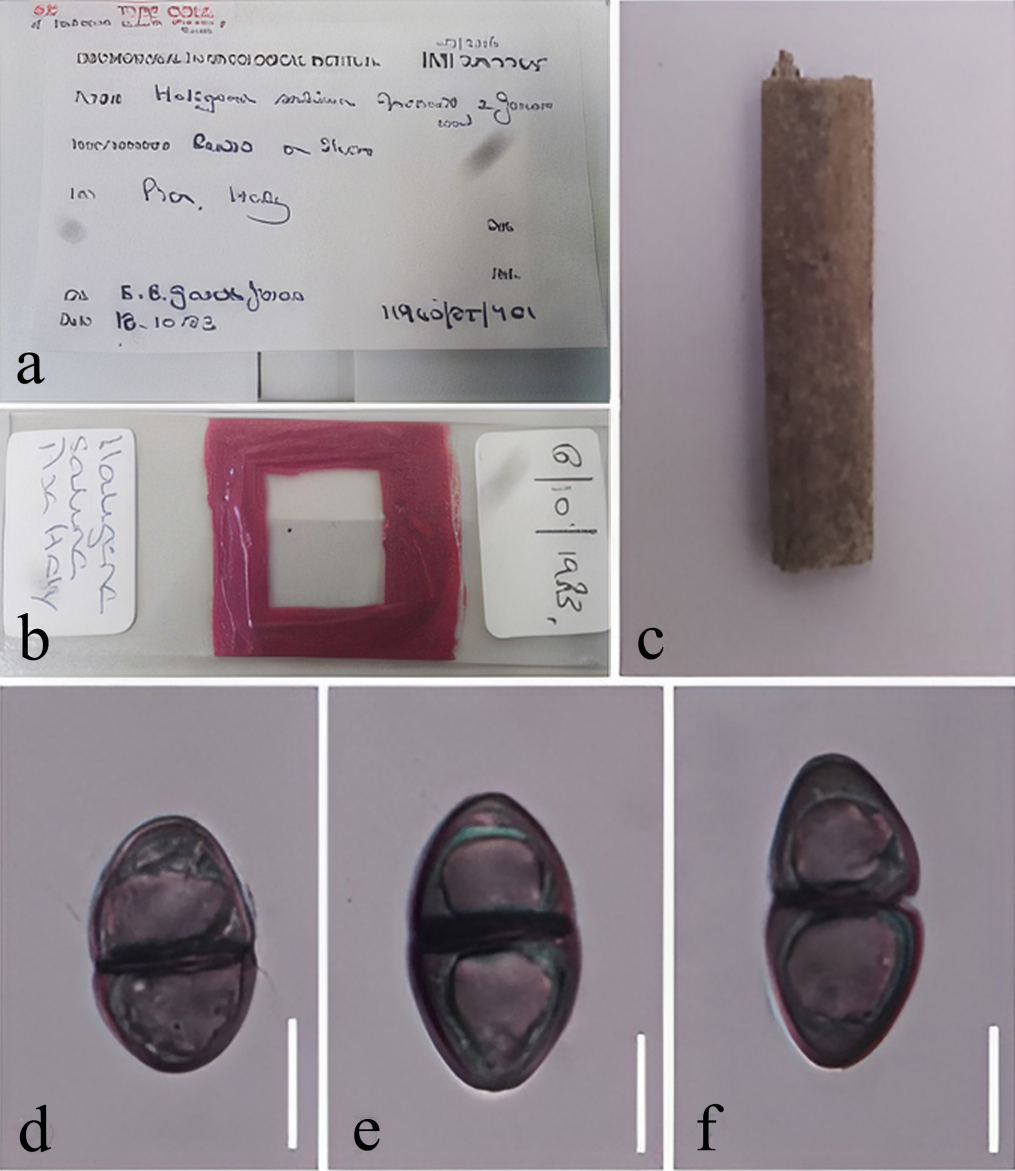
*Morakotiella salina* (IMI 297765, holotype). **a, c**. Herbarium material of Morakotiella*salina*; **b**. Microslide; **d–f**. Ascospores. Scale bars: 5 µm (**d–f**).

##### Material examined.

South Wales, • Marloes, on unidentified intertidal wood (BCC12781, type specimen).

##### Notes.

*Morakotiella salina* is a saprobic fungus described from submerged and intertidal wood. The ascospores germinate readily on agar plate and grow slowly but do not sporulate in culture. It is not widely collected but has been collected from Friday Harbour (USA) and Marloes (South Wales) (Jones 1985; Farrant and Jones 1986; Sakayaroj et al. 2005).

### *Naïs* Kohlm.

The genus *Naïs* was introduced by Kohlmeyer (1962a) with *Naïs inornata* as the type species. Later, two species were added to this genus, *Naïs glitra* from marine habitats and *N. aquatica* from freshwater (Crane and Shearer 1986; Hyde 1992b). *Naïs glitra* was transferred to the genus *Saagaromyces* based on sequence data (Pang et al. 2003a). The genus was referred to the Halosphaeriaceae by Kohlmeyer (1962a) and this was confirmed by sequence data (Chen et al. 1999; Pang et al. 2003a; Sakayaroj et al. 2011). Zhang and Yang in (Yang et al. 2024) introduced *Nais submersa* from wood in freshwater from Jianshui City, Yunnan province, China which grouped with *Nais inornata* in clade including *Natantispora unipolaris* and *Nat. retorquens*. However, *Naïs submersa* has an equatorial band of granular globose bodies in the ascospores which excludes it from *Natantispora*. Yang et al. (2024) query whether to combine *Natantispora* and *Naïs* but deferred action until further evidence becomes available. Pang et al. (2003a) demonstrated that the genus is polyphyletic.

#### Naïs

Taxon classificationFungiMicroascalesHalosphaeriaceae

Kohlm., Nova Hedwigia 4: 409 (1962)

0C11FC39-7BCC-5EFE-AE7B-CA08B293146E

Index Fungorum: IF92064

##### Description.

Saprobic on submerged woody substrates in marine and freshwater habitats. **Sexual morph. *Ascomata*** perithecial, immersed or superficial, subglobose, ostiolate, papillate, coriaceous, dark brown to black, gregarious, long necks, periphysate, catenophyses present. ***Asci*** 8-spored, clavate, unitunicate, short pedunculate, thin-walled, no apical apparatus, early deliquescing. ***Ascospores*** ellipsoidal to broadly ellipsoidal, 1-septate, hyaline, slightly constricted at the septum, lacking appendages or a sheath. **Asexual morph**. Undetermined.

##### Type species.

*Naïs inornata* Kohlm., Nova Hedwigia 4: 409 (1962).

##### Notes.

*Naïs* comprises both marine and freshwater species and are widely reported and worldwide in distribution (Jones et al. 2017; Calabon et al. 2022). *Naïs* species have been found from driftwood, *Spartina alterniflora*, test blocks e.g. *Acanthus ilicifolius*, *Albizia falcata*, *Al. procera*, *Avicennia alba*, *Av. lanata*, *balsa*, *Bruguiera cylindrica*, *Castanopsis javanica*, *Dicorynia paraensis*, *Fagus sylvatica Laphira procera*, *Ocotea rodiaei*, *Pinus sylvestris*, *Pinus* sp. and *Rhizophora apiculata*, *Phragmites australis*, intertidal wood, teak blocks, milfoil, *Spartina* spp., mangrove wood, freshwater wood (beech, Scots pine, balsa), herbaceous substrata, woody panels (beech, Scots pine) (Kohlmeyer 1962a; Churchand and McClaren 1973; Gessner and Goos 1973; Shearer and Crane 1978; Tan et al. 1989; Hyde 1990b, 1992b; Leong et al. 1991; Lintott and Lintott 2002; Abdel-Aziz 2008, 2010, 2016; Raja et al. 2009; Alias et al. 2010; Samón-Legrá et al. 2014; https://www.marinefungi.org).

Currently three *Naïs* species are known, *N. inornata* from marine habitats and *N. aquatica* and *N. submersa* from freshwater habitats. The equatorial band of granular globose bodies distinguishes *Naïs* from the closely related genera *Aniptodera*, *Halosarpheia*, and *Lignincola* (Hyde 1992b).

##### Molecular evaluation.

Molecular data places *Nais* in the Halosphaeriaceae grouping with genera with polar unfurling appendages (Jones et al. 2015), e.g. *Aniptodera* species (Sakayaroj et al. 2011). In Fig. 4, *Naïs inornata* groups with *Aniptodera chesapeakensis* in a well-supported clade. Divergence-time estimates indicate that this relationship originated in the Jurassic–Early Cretaceous (~150 MYA, 95% CI: 112.6–195.2 MYA) (Fig. 5). *Nais* species need to be recollected, isolated and sequenced, along with *Natantispora* species to resolve their generic relationship.

#### Nais inornata

Taxon classificationFungiMicroascalesHalosphaeriaceae

Kohlm., Nova Hedwigia 4: 409 (1962)

1F7EB8BE-004E-5B89-85F0-2F90519D912E

Index Fungorum: IF631135

##### Description.

Saprobic on submerged wood in aquatic habitats. **Sexual morph. *Ascomata*** 135–250 µm high, 200–370 µm diam., dark brown to black, subglobose or depressed, immersed or superficial, ostiolate, papillate, coriaceous, gregarious. ***Peridium*** 12–15 µm thick, composed of two or three layers of thick-walled, oblong or ellipsoidal cells. ***Necks*** 100–620 × 30–44 µm, periphysate. ***Catenophyses*** present, 55–240 × 5.5–36 µm, including up to 15 cells per chain. ***Asci*** 85–160 × 25–33 µm, 8–spored, unitunicate, clavate, thin-walled, early deliquescing, without apical apparatuses, short pedicellate. ***Ascospores*** 22–32 × 12–16 µm, hyaline, broadly ellipsoidal, 1-septate, slightly or not constricted at the septum, with one large oil globule in each cell and many small ones near the septum and the apices, lacking appendages. **Asexual morph**. Undetermined. (Description based on Kohlmeyer 1962a).

##### Notes.

*Nais inornata* grows in marine, brackish and freshwater habitats. *Nais inornata* has thin-walled asci, deliquesce early and 22–32 × 12–16 µm, broadly ellipsoidal ascospores (Kohlmeyer 1962a). Type material of *N. inornata* is lacking material of asci and ascospores.

##### Distribution.

Canada, France, German, UK (England, Wales), USA (Maryland, Mississippi. Rhode Island).

### *Natantispora* J. Campbell, J.L. Anderson & Shearer

The genus *Natantispora* was introduced to accommodate two species which were originally introduced as *Halosarpheia lotica* and *H. retorquens* (Campbell et al. 2003). A new species *Natantispora unipolaris* was described on a dead stem of *Phragmites australis* from an estuary in southern Taiwan (Liu et al. 2015), and characterised by having only one appendage instead of two as in *N. lotica* and *N. retorquens*. The genus was referred to the Halosphaeriaceae by Campbell et al. (2003) and this was confirmed by additional sequence data (Jones et al. 2009; Liu et al. 2015).

#### Natantispora

Taxon classificationFungiMicroascalesHalosphaeriaceae

J. Campbell, J.L. Anderson & Shearer, Mycologia 95 (3): 543 (2003)

27571CB3-D0CC-5056-8044-285C08177658

Index Fungorum: IF28703

##### Description.

Saprobic on submerged woody substrates in marine and freshwater habitats. **Sexual morph. *Ascomata*** perithecial, superficial to immersed, globose, black, membranous, ostiolate, papillate, long, cylindrical, necks, periphysate, dark-pigmented, gradually lightening toward the apex. ***Peridium*** thin-walled, composed of pseudoparenchyma in longitudinal section, of *textura angularis* in surface view. ***Paraphyses*** absent, catenophyses present. ***Asci*** 8-spored, clavate, unitunicate, thin-walled, lacking an apical apparatus, early deliquescing. ***Ascospores*** hyaline, fusiform to ellipsoidal, 1-septate. ***Appendages*** are unipolar or bipolar, coiled into a hamate structure, initially closely adpressed to the spore wall, separating and unfurling in water to form a long, fine, sticky, thread-like structure. **Asexual morph**. Undetermined.

##### Type species.

*Natantispora retorquens* (Shearer & J.L. Crane) J. Campb., J.L. Anderson & Shearer, Mycologia 95 (3): 543 (2003).

##### Notes.

**Figure 27.**
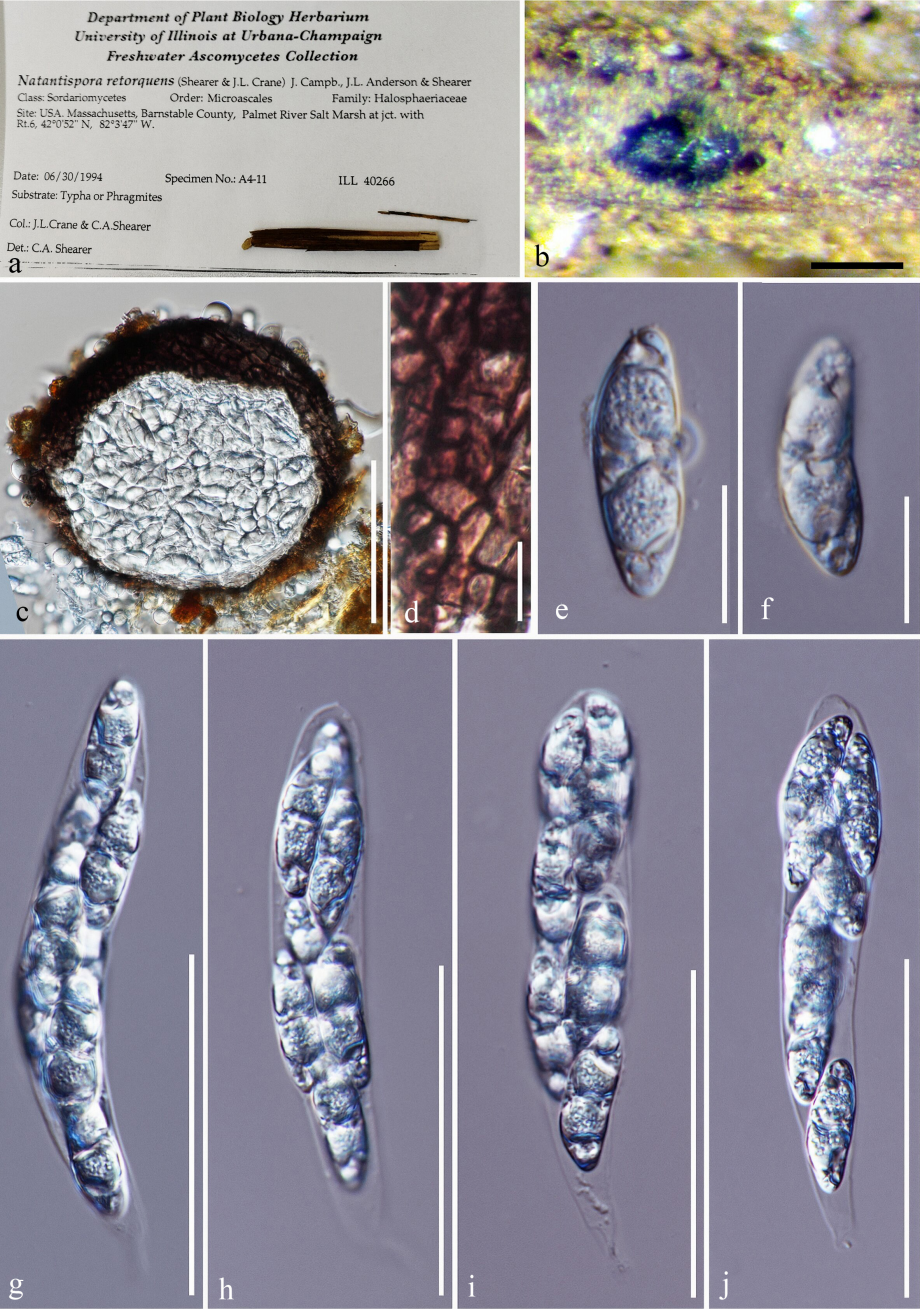
*Natantispora retorquens*. **a**. Herbarium material; **b**. Ascoma on wood; **c**. Section of ascoma; **d**. Wall of ascoma; **e–h**. Asci with short pedicel; **i, j**. Ascospores. Scale bars: 100 µm (**b, c**); 50 µm (**e–h**); 10 µm (**d, i, j**).

**Figure 28.**
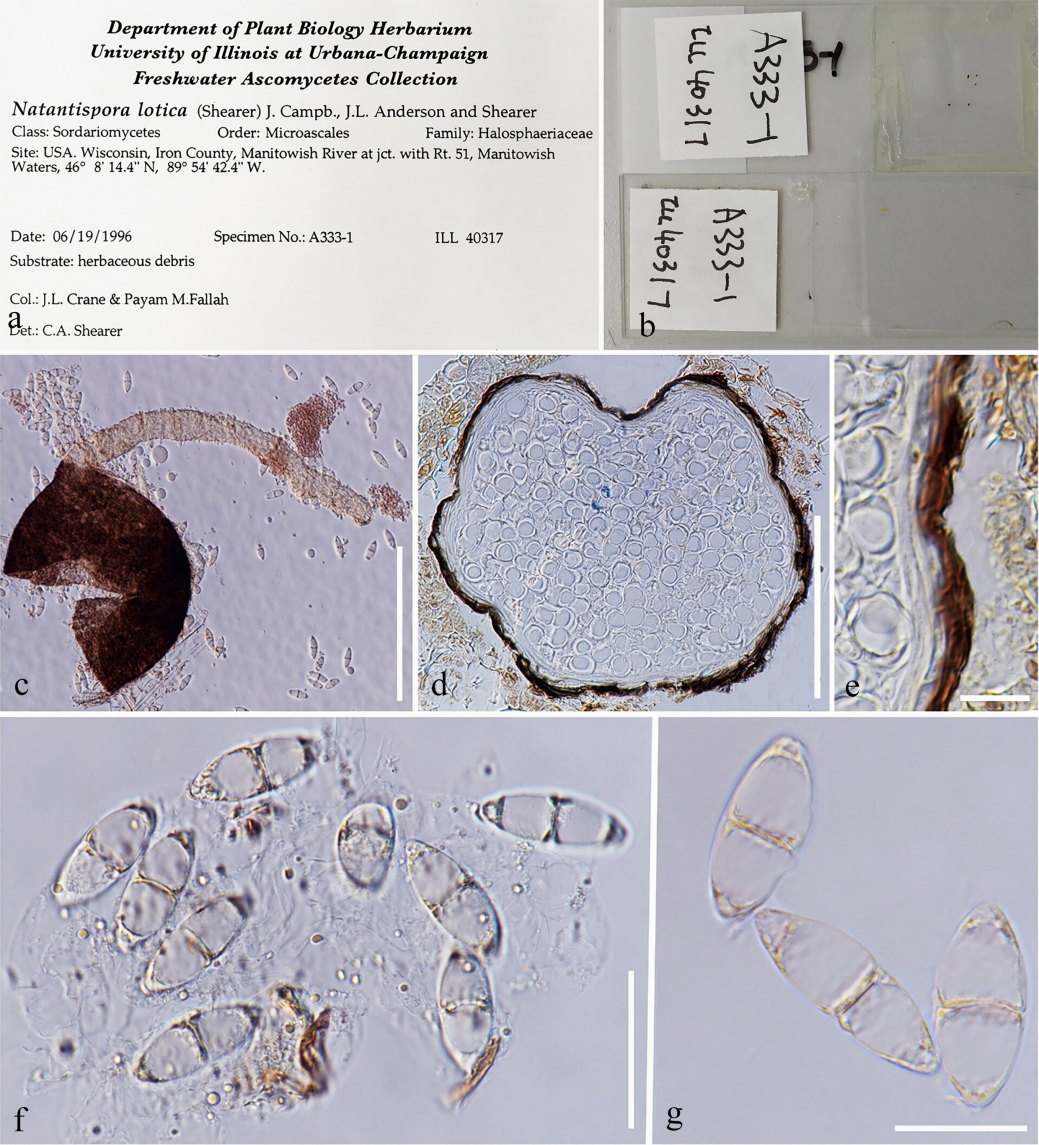
*Natantispora lotica*. **a, b**. Herbarium details; **c**. Ascoma with long neck; **d**. Section of ascoma, full of ascospores; **e**. Close up of ascoma wall; **f**. Squash preparation ascus and ascospores; **g**. Ascospores. Scale bars: 100 µm (**c**); 50 µm (**d**); 10 µm (**e, f**); 5 µm (**g**).

Campbell et al. (2003) introduced the genus *Natantispora* based on sequence data of two *Halosarpheia* species, although distinguishing morphological features at the generic level are not well established. The type species *N. retorquens* (Fig. 27) was described from balsa wood submerged in freshwater habitat from Illinois (USA), with globose to subglobose ascomata, hyaline, bicelled, ellipsoidal ascospores with a polar appendage pad extending over the mid-septum which uncoils to form a long thread (Shearer and Crane 1980). *Natantispora lotica* (Fig. 28) is morphologically similar to *N. retorquens* but differs by having larger ascospores (26–38 × 10–14 μm vs. 20–33 × 7–11 μm) and appendages not extending to the middle septum (Shearer 1984). *Natantispora unipolaris* is morphologically similar to other *Natantispora* species, but differs in having only one ascospore appendage, smaller ascospores with many small oil globules and one large in each cell (Liu et al. 2015). Some marine genera also have a single polar appendage e.g., *Moana*, *Oceanitis*, *Okeanomyces*, *Ophiodeira* and *Tirispora*, but phylogenetically they are distant from *N. unipolaris*. Campbell et al. (2003) stated that more genera with polar unfurling appendages need to be sequenced to determine the validity of this genus.

This genus is represented by two species; *N. retorquens* and *N. unipolaris* which have been reported from freshwater and marine habitats (Jones et al. 2019; Luo et al. 2019). *Natantispora* species are worldwide in their distribution (Jones et al. 2017; Luo et al. 2019). *Natantispora* species have been reported from decomposing mangrove wood such as *Avicennia alba*, *A. marina*, *Rhizophora mucronata*, *Bruguiera cylindrica*, *Spartina maritima* (Pande 2008; Calado et al. 2015; Devadatha et al. 2021), and other woody and herbaceous debris e. g. *Acanthus ilicifolius*, bamboo, *Ficus hispida* baits, palm, *Phragmites australis*, *Raphia australis* and *Typha* sp., submerged in aquatic habitats (Shearer 1984; Hyde et al. 1999b; Lu et al. 2000; Wong and Hyde 2001; Cai et al. 2003; Campbell et al. 2003; Luo et al. 2004; Raja et al. 2009; Liu et al. 2015).

##### Molecular evaluation.

*Natantispora* species formed a well-supported monophyletic group and a sister clade with *Halosphaeria appendiculata* and *Lignincola laevis* (Fig. 4). Divergence-time estimates indicate that *Natantispora* separated from this lineage in the mid-Cretaceous (~110 MYA, 95% CI: 38.5–313.3 MYA), underscoring its independence within the family (Fig. 5). *Natantispora lotica* grouped in a sister clade to *N. retorquens* and *N. unipolaris* and may belong to a different genus. However, there are no sequences derived from the type material and new collections, isolation and sequencing of this species is necessary. *Natantispora* is phylogenetically distinct from the type species of *Halosarpheia* and from other clades with halosarpheia-like species.

#### Natantispora retorquens

Taxon classificationFungiMicroascalesHalosphaeriaceae

(Shearer & J.L. Crane) J. Campb., J.L. Anderson & Shearer, Mycologia 95 (3): 543 (2003)

B1A28D87-CFF5-5371-A493-EF3C118623D2

Index Fungorum: IF373730

Fig. 27

Halosarpheia retorquens Shearer & J.L. Crane, Bot. Mar. 23: 608 (1981). Basionym.

##### Description.

Saprobic on submerged wood in aquatic habitats. **Sexual morph. *Ascomata*** 150–366 µm diam., globose to subglobose, membranous, hyaline at first, becoming black, superficial or immersed, solitary to gregarious, ostiolate. ***Necks*** 108–564 long, 13.5–50 µm diam., cylindrical, periphysate, dark at base, hyaline at apex. ***Peridium*** multi-layered, the outer layer brown to black, inner layer hyaline of laterally compressed cells. ***Asci*** 53–144 × 14.4–24 µm, unitunicate, 8-spored, clavate, thin-walled, deliquescing before or at maturity. ***Catenophyses*** present. ***Ascospores*** 20–33 × 7–11 µm, hyaline, ellipsoidal, 1-septate, not constricted at the septum, appendaged. ***Appendages*** bipolar, composed of a single, coiled or folded filament, at first hamate, finally unwinding in water to produce a long, fine filament. **Asexual morph**. Undetermined.

##### Notes.

Hyde et al. (1987) showed that *N. retorquens* sporulated in culture.

##### Distribution.

Denmark, Portugal, Taiwan, UK (England), USA (Chesapeake, Massachusetts, Minnesota).

### *Naufragella* Kohlm. & Volkm.-Kohlm.

*Naufragella* was introduced by Kohlmeyer and Volkmann-Kohlmeyer (1998) to accommodate the type species *N. delmarensis*, along with *N. spinibarbata* (basionym *Remispora spinibarbata*). Subsequently, Abdel-Wahab (2011b) transferred *N. delmarensis* and *N. spinibarbata* to the genus *Nohea* introduced by Kohlmeyer and Volkmann-Kohlmeyer (1991b), based on a phylogenetic study.

#### Naufragella

Taxon classificationFungiMicroascalesHalosphaeriaceae

Kohlm. & Volkm.-Kohlm., Syst. Ascomycetum 16: 10 (1998)

7C9570AE-1006-54F0-857B-A5144D8E3240

Index Fungorum: IF27779

##### Description.

Saprobic on wood. **Sexual morph. *Ascomata*** ovoid, erumpent, ostiolate with a short neck. coriaceous, hyaline-cream colored. ***Peridium*** thick, one-layered of elongate angular cells. ***Necks*** conical. ***Paraphyses*** absent, catenophyses in young perithecia. ***Asci*** 8-spored, unitunicate, thin-walled, no apical apparatus, broadly clavate, pedunculate, deliquescing at maturity. ***Ascospores*** 1-sepate, ellipsoidal-broadly fusiform, slightly constricted at the septum, hyaline, appendaged. ***Appendages*** of two types: 1). Polar appendages spine-like, flattened, elongating, forming long sheet-like extensions, 2). Subpolar appendages bristle to hair-like developing from a cushion-like pad and surrounding the polar appendage. **Asexual morph**. Not determined.

##### Type species.

*Naufragella spinibarbata* (Jørg. Koch) Kohlm. & Volkm.-Kohlm., Syst. Ascomycetum 16: 11 (1998).

##### Notes.

*Nohea* and *Naufragella* are morphologically closely related and can be distinguished by their ascospore/appendages: in *Nohea*, one appendage is flat and attached to the spore by refractive subapical pads, while on the other side two bundles of fibres form tufts when mounted in water; in *Naufragella* (*N. spinibarbata*) the main appendage is apical with hair-like appendages surrounding the base of the main appendage.

Appendage origin and development of the ascospores of *Naufragella* was not described and lacked the clarity of scanning electron micrographs as used by Jones et al. (1983a, 1983b) and Nakagiri and Tokura (1987), so vital in characterizing the range of appendages found in the Halosphaeriaceae.

##### Molecular evaluation.

No sequences of *Naufragella*/*Nohea* species are derived from the type species and this has created confusion as to the identity of the taxa is concerned. In Fig. 4, *Nohea umiumi* and *Nohea delmarensis* formed a highly supported clade, sistering with two *Tinhaudeus formosanus* strains, while two strains of *Nohea spinibarbata* were placed in an adjacent clade. There are no ITS rDNA sequences for any of these strains. *Nohea delmarensis* and *N. umiumi* certainly belong to the same genus, while *Nohea spinibarbata* strain clearly does not belong to *Nohea* and is best referred back to *Naufragella* until further strains are collected, isolated and sequenced, especially the ITS rDNA.

#### Naufragella spinibarbata

Taxon classificationFungiMicroascalesHalosphaeriaceae

(Jørg. Koch) Kohlm. & Volkm.-Kohlm., Syst. Ascomycetum 16: 11 (1998)

4368A96E-5656-5693-A27E-7BFFAE46BAA7

Index Fungorum: IF442614

Fig. 3c

Remispora spinibarbata Jørg. Koch, Nordic J. Bot. 8 (5): 517 (1989). Basionym.
Nohea spinibarbata (Jørg. Koch) Abdel-Wahab, Mycotaxon 115: 448 (2011). Synonym.

##### Description.

Saprobic on wood. **Sexual morph. *Ascomata*** 325–330 µm high, 245–300 µm wide, ovoid, erumpent, ostiolate with a short neck, coriaceous, hyaline-cream colored. ***Peridium*** 17–24 µm thick, one-layered of elongate angular cells. ***Necks*** 32–55 µm, conical. ***Paraphyses*** absent, catenophyses in young perithecia. ***Asci*** 72–115 × 24–26 µm, 8-spored, unitunicate, thin-walled, no apical apparatus, broadly clavate, pedunculate, deliquescing at maturity. ***Ascospores*** 18–22 × 8–9.7–12 µm,1-septate, ellipsoidal-broadly fusiform, slightly constricted at the septum, hyaline, appendaged. ***Appendages*** of two types: 1). Polar appendages spine-like, flattened, elongating, forming long sheet-like extensions, 2). Subpolar appendages bristle to hair-like developing from a cushion-like pad and surrounding the polar appendage. **Asexual morph**. Not determined.

##### Notes.

*Naufragella spinibarbata* is saprobic on driftwood and bark, forming light-colored ascomata, which can be easily overlooked. *Naufragella spinibarbata* was previously referred by Kohlmeyer and Volkm.-Kohlmeyer (1998) in *Remispora*. It is phylogenetically distantly placed from *Remispora* (Fig. 4) and ascospores have secondary subpolar bristle to hair-like appendages.

### *Nautosphaeria* E.B.G. Jones

*Nautosphaeria* was introduced by Jones (1964) to accommodate the species *Nautosphaeria cristaminuta* based on a study of the ascospore color, number of cells and the characteristic tuft-like appendages apically and at the central septum. The genus is monophyletic.

#### Nautosphaeria

Taxon classificationFungiMicroascalesHalosphaeriaceae

E.B.G. Jones, Trans. Brit. Mycol. Soc. 47 (1): 97 (1964)

2425D5FB-9751-5ACD-BC2E-A6E99511F946

Index Fungorum: IF3428

##### Description.

Saprobic on submerged wood. **Sexual morph. *Ascomata*** immersed in the substratum, hyaline or cream, solitary, membranous, neck long. ***Asci*** 8-spored, clavate, unitunicate, soon deliquescing. ***Ascospores*** ellipsoid, apically and laterally appendiculate, grey or fuscous, unicellular. **Asexual morph**. Undetermined. (Adapted from Jones 1964).

##### Type species.

*Nautosphaeria cristaminuta* E.B.G. Jones, Trans. Brit. Mycol. Soc. 47 (1): 97 (1964).

##### Notes.

*Nautosphaeria* is saprobic on submerged and driftwood along the shore (Jones 1964). Type material is lacking details of asci and ascospores.

Sequence data placed it in the Halosphaeriaceae as a sister clade to *Tubakiella galerita* with high statistical support (Sakayaroj et al. 2011; Jones et al. 2017). The two genera share a few morphological features in common, with the polar and equatorial hair–like appendages in tufts in *N. cristaminuta* (Koch and Jones 1983), while in *T. galerita* there is only polar gelatinous appendages that are composed of fibrillar material and formed by fragmentation of an exosporic layer of the ascospore (Johnson et al. 1984). The tuft-like appendages of *N. cristaminuta* bare a resemblance to those of *Nereiospora* species (Jones et al. 1983a). However, phylogenetically they are distantly placed.

##### Molecular evaluation.

Although its placement in the Halosphaeriaceae as a sister clade to *Tubakiella galerita* with good statistical support (Sakayaroj et al. 2011; Jones et al. 2017; Fig. 4), the two genera were recently excluded from Halosphaeriaceae and placed in Microascales genera *incertae sedis* (Bao et al. 2023). Divergence-time analysis indicates that the crown of *Nautosphaeria* is relatively young (0–9.4 MYA, 95% CI: 0–9.4 MYA), while its split from *Tubakiella* dates back much deeper (~146 MYA, 95% CI: 58.0–370.3 MYA), highlighting its independent evolutionary trajectory (Fig. 5). Further collections and sequencing are required to confirm its placement in the Halosphaeriaceae and further establish its relationship with other genera.

#### Nautosphaeria cristaminuta

Taxon classificationFungiMicroascalesHalosphaeriaceae

E.B.G. Jones, Trans. Brit. Mycol. Soc. 47 (1): 97 (1964)

7ACA1100-2800-588D-B648-962F07D75156

Index Fungorum: IF335090

##### Description.

Saprobic on submerged wood. **Sexual morph. *Ascomata*** (144–)180–288 × 108–198 µm diam., globose to subglobose, rarely elongate, hyaline or yellowish-white, membranous, thin-walled, aparaphysate, necks hyaline, 108–396 × 20–30 µm. ***Asci*** 8-spored, clavate, unitunicate, soon deliquescing. ***Ascospores*** hyaline to grey or fuscous at maturity, 14–18 × 7–8.5 µm, ellipsoidal, one-celled, 1–2 large guttules, with tufts of hair-like appendages at both ends, and four equatorial tufts each at right angles to one another. Apical and lateral appendages measure 4.5–11.5 µm. **Asexual morph**. Undetermined. (Adapted from Jones 1964).

##### Notes.

The fungus was first found growing on timber submerged in the sea for 48 weeks. Other fungi found growing in association with it were *Ceriosporopsis cambrensis*, *C. halima*, *Lulwoana uniseptata* (=*Zalerion maritimum*), *Lulworthia opaca*, *Halazoon purpurea*, *Halosphaeriopsis alopallonella*, *Remispora hamata* and *Sporidesmium salinum* (Jones 1964).

##### Distribution.

Denmark, France, Germany, Italy, Japan, Spain, Taiwan, UK (Isle of Man) and known from coastal temperate areas, on drift and trapped wood, especially on gymnospermous wood.

#### Neoaniptodera

Taxon classificationFungiMicroascalesHalosphaeriaceae

Abdel-Wahab, K.L. Pang, P. Correia & E.B.G. Jones gen. nov.

470B0F05-7A7A-5BBF-A2F5-96FF27A29D5F

Index Fungorum: IF903245

##### Etymology.

‘*Neo*’ similar to *Aniptodera*.

##### Description.

Saprobic on culms of *Juncus roemerianus*. **Sexual morph. *Ascomata*** subglobose to pyriform, immersed, ostiolate, papillate, coriaceous, greyish-brown, solitary. ***Necks*** cylindrical to conical, periphysate. ***Peridium*** two layered, outer layer composed of polygonal, fuscous cells, inner layer flattened hyaline cells forming a *textura angularis*. ***Catenophyses*** present. ***Asci*** 8-spored, clavate, pedunculate with an apical pore, thin-walled, unitunicate, persistent. ***Ascospores*** 1-septate, ellipsoidal, not constricted at the septum, thick-walled, lacking appendages. **Asexual morph**. Undetermined.

##### Type species.

*Neoaniptodera juncicola* (Volkm.-Kohlm. & Kohlm.) Abdel-Wahab, K.L. Pang, P. Correia & E.B.G. Jones.

##### Notes.

*Neoaniptodera* has the typical characteristics of *Aniptodera*, including light-colored ascomata, asci with an apical pore and thick-walled ascospores with no appendages. Phylogenetically, this genus was not monophyletic with *Aniptodera* (*A. chesapeakensis*) (Fig. 4).

##### Molecular evaluation.

*Neoaniptodera juncicola* did not group with the type species of *Aniptodera* but with *Panorbis viscosus* in a sister clade to *Neohalosarpheia marina*, *Nimbospora effusa*, and *Nohea umiumi* (Abdel-Wahab and Nagahama 2011). In our study (Fig. 4) *Neoaniptodera juncicola* grouped with the *Kochiella* clade and *Ocostaspora apilongissima* in an unsupported clade. Divergence-time analysis suggests that the split between *Neoaniptodera* and *Ocostaspora* occurred relatively recently (~15.1 MYA, 95% CI: 3.5–65.1 MYA) (Fig. 5). A recollection of this species with the sequencing of multiple genes is required to resolve the taxonomy of this species.

#### Neoaniptodera juncicola

Taxon classificationFungiMicroascalesHalosphaeriaceae

(Volkm.-Kohlm. & Kohlm.) Abdel-Wahab, K.L. Pang, P. Correia & E.B.G. Jones comb. nov.

7EA898C3-0658-5DA2-B604-88B70FBFD5AC

Index Fungorum: IF903246

Aniptodera juncicola Volkm.-Kohlm. & Kohlm., Bot. Mar. 37 (2): 109 (1994). Basionym.

##### Description.

Saprobic on culms of *Juncus roemerianus*. **Sexual morph. *Ascomata*** 175–280 µm high, 195–315 µm in diam., subglobose to pyriform, immersed, ostiolate, papillate, coriaceous, greyish-brown, solitary. ***Necks*** 145–255 µm long, 65–105 µm diam., cylindrical to conical, periphysate. ***Peridium*** 16–23 µm thick, two layered, outer layer composed of polygonal, fuscous cells, inner layer flattened hyaline cells forming a *textura angularis*. ***Catenophyses*** present. ***Asci*** 145–175 × 18.5–20.5 µm, 8-spored, clavate, pedunculate with an apical pore, thin-walled, unitunicate, persistent. ***Ascospores*** 23.8–30.7 × 8.2–11.6 µm, 1-septate, ellipsoidal, not constricted at the septum, thick-walled, lacking appendages. **Asexual morph**. Undetermined.

##### Type material.

On *Juncus roemerianus* collected Broad Creek, Carteret County, North Carolina, 25 July 1993, J. Kohlmeyer J.K. 5516 (Holotype).

##### Distribution.

North Carolina, USA.

##### Notes.

This species is rarely collected and may be host-specific on *Juncus*.

#### Neogesasha

Taxon classificationFungiMicroascalesHalosphaeriaceae

Day., Abdel-Wahab, M.F. Caeiro & K.L. Pang gen. nov.

D6ED5907-FA05-5820-B888-8D8DC1430B59

Index Fungorum: IF903286

##### Etymology.

‘*Neo*’ similar to *Gesasha*.

##### Description.

Saprobic on decaying mangrove wood. **Sexual morph. *Ascomata*** globose to subglobose, hyaline to light brown, coriaceous, immersed or erumpent, solitary, ostiolate with a long, hyaline neck. ***Necks*** hyaline, cylindrical, single or branched, periphysate. ***Peridium*** consists of 7–10 of thick-walled, hyaline cells, forming *textura angularis*, the first two layers to the outside are yellow brown in color. ***Catenophyses*** present. ***Asci*** unitunicate, clavate with an apical thickening and pore, apically truncate with cytoplasmic retraction, persistent, with eight overlapping biseriate ascospores. ***Ascospores*** hyaline, ellipsoidal, 1-septate, not constricted at the septum, lacking appendages. **Asexual morph**. Undetermined.

##### Type species.

*Neogesasha mangrovei* (Abdel-Wahab & Nagahama) Day., Abdel-Wahab, M.F. Caeiro & K.L. Pang.

##### Notes.

*Neogesasha* differs from *Gesasha* (*G. peditatus*) by having non-appendaged ascospores with median septa and a thinner wall. *Gesasha peditatus* has thick-walled, foot-like ascospores with sub-median distosepta and amorphous polar appendages. *Neogesasha* closely resembles species of *Aniptodera* in having a persistent ascus with apical thickening and cytoplasmic retraction. However, *Neogesasha* differs from the type species of *Aniptodera* in having hyaline to light brown ascomata, asci with ring and thin-walled ascospores.

##### Molecular evaluation.

*Neogesasha* was phylogenetically distant from the type species of *Aniptodera*, *A. chesapeakensis*, and grouped in highly supported clades with *Moana*, *Thalassogena* and *Gesasha* (Fig. 4). Divergence-time analysis indicates that *Neogesasha* and *Gesasha* separated around ~68 MYA (95% CI: 21.1–221.8 MYA), supporting their recognition as distinct genera within the Halosphaeriaceae (Fig. 5).

#### Neogesasha mangrovei

Taxon classificationFungiMicroascalesHalosphaeriaceae

(Abdel-Wahab & Nagahama) Day., Abdel-Wahab, M.F. Caeiro & K.L. Pang comb. nov.

443D8AA6-5B1F-5218-AB06-F055875F676B

Index Fungorum: IF903285

Fig. 29

Gesasha mangrovei Abdel-Wahab & Nagah., Nova Hedwigia 92 (3–4): 507 (2011). Basionym.

##### Description.

Saprobic on decaying mangrove wood. **Sexual morph. *Ascomata*** 200–300 × 210–360 μm, globose to subglobose, hyaline to light brown, coriaceous, immersed or erumpent, solitary, ostiolate with a long, hyaline neck. ***Necks*** 400–720 × 48–65 μm, hyaline, cylindrical, single or branched, periphysate. ***Periphyses*** up to 12 μm long and 0.5 μm in width, neck wall 20 μm thick. ***Peridium*** 16–24 μm thick, consists of 7–10 of thick-walled, hyaline cells, forming *textura angularis*, the first two layers to the outside are yellow brown in color. ***Catenophyses*** present. ***Asci*** 62–82 × 14–22 μm, unitunicate, clavate with an apical thickening and pore, apically truncate with cytoplasmic retraction, persistent, with eight overlapping biseriate ascospores. ***Ascospores*** 16–18 × 8–10 μm, hyaline, ellipsoidal, one-septate, not constricted at the septum, appendages absent. **Asexual morph**. Undetermined.

**Figure 29.**
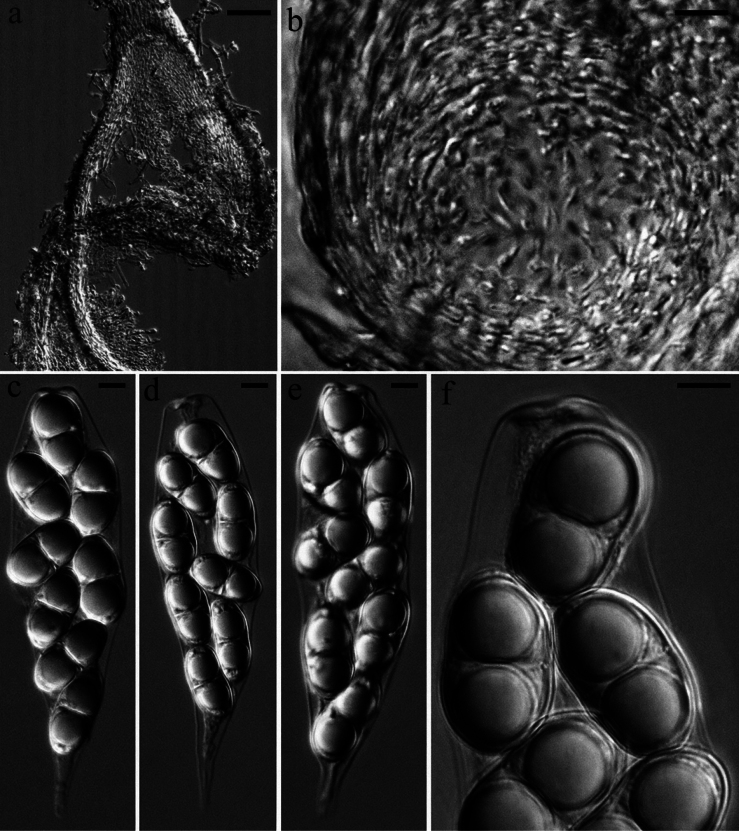
*Neogesasha mangrovei*. **a**. Vertical section through ascoma; **b**. Horizontal section through periphysate neck; **c–e**. Variously shaped mature asci; **f**. Magnified part of the ascus apex showing the ring and the cytoplasmic retraction. Scale bars: 50 μm (**a**); 5 μm (**b–f**).

##### Material examined.

Japan, • Okinawa, Higashi-son, Gesashi mangroves, on decayed wood in the intertidal zone, July 2008, coll. M. A. Abdel-Wahab (holotype, IMI 397960).

##### Notes.

*Neogesasha mangrovei* is a saprobe occurring on mangrove wood.

#### Neohalosarpheia

Taxon classificationFungiMicroascalesHalosphaeriaceae

Abdel-Wahab, K.L. Pang & P. Correia gen. nov.

2E3C3E93-8643-54BF-B119-BEEDAAC76248

Index Fungorum: IF903288

##### Etymology.

*Neo* similar to the genus *Halosarpheia*.

##### Description.

Saprobic on decaying mangrove wood. **Sexual morph. *Ascomata*** bottle-shaped, subhyaline, light brown or fuscous, membranous, mostly immersed, solitary or gregarious, ostiolate, papillate. ***Necks*** cylindrical, periphysate, hyaline to light-brown. ***Peridium*** one-layered, forming *textura angularis* with large lumina. ***Catenophyses*** present. ***Asci*** thin-walled, unitunicate, 8-spored, clavate to subcylindrical, persistent, apically truncate, thickened at the apex, with an apical pore, short-pedicellate. ***Ascospores*** cylindrical-ellipsoidal, apically rounded, 1-septate at the middle, not or slightly constricted at the septum, hyaline, with a gelatinous appendage at each apex, round or apiculate, sometimes deciduous or deliquescing. **Asexual morph**. Undetermined.

##### Type species.

*Neohalosarpheia marina* (Cribb & J.W. Cribb) Abdel-Wahab, K.L. Pang & P. Correia.

##### Notes.

*Neohalosarpheia* was distantly related with *Halosarpheia* (*H. fibrosa*) (Campbell et al. 2003; Sakayaroj et al. 2011; Abdel-Wahab and Nagahama 2012; Fig. 4), and is therefore referred to the new genus *Neohalosarpheia*. *Neohalosarpheia* is saprobic on mangrove dead roots of *Avicennia marina*.

##### Molecular evaluation.

Multi-gene phylogeny of Halosphaeriaceae in this study as well as previous studies (e.g. Campbell et al. 2003; Sakayaroj et al. 2011; Abdel-Wahab and Nagahama 2012), showed that *Halosarpheia marina* did not group in the *Halosarpheiasensu stricto* clade, but was distantly placed, and grouped in a clade with *Nimbospora* with no support (Fig. 4). Divergence-time analysis indicates that *Neohalosarpheia marina* diverged from *Nimbospora* in the late Paleogene (~38 MYA, 95% CI: 29.9–46.4 MYA), supporting its recognition as a distinct genus (Fig. 5).

#### Neohalosarpheia marina

Taxon classificationFungiMicroascalesHalosphaeriaceae

(Cribb & J.W. Cribb) Abdel-Wahab, K.L. Pang & P. Correia comb. nov.

B82BACE2-C083-5ACE-85E3-3FE2B6894189

Index Fungorum: IF903287

Fig. 30

Gnomonia marina Cribb & J.W. Cribb, Pap. Dept. Bot. Univ. Queensland 3 (12): 100 (1956). Basionym.
Halosarpheia marina (Cribb & J.W. Cribb) Kohlm., Mar. Ecol. 5 (4): 345 (1984). Synonym.

##### Description.

Saprobic on decaying mangrove wood. **Sexual morph. *Ascomata*** 140–300 μm in diameter, bottle-shaped, subhyaline, light brown or fuscous, membranous, mostly immersed, solitary or gregarious, surrounded by brown hyphae 3–5 μm thick, ostiolate, papillate. ***Necks*** 100–560 µm long, 42–140 µm diam., lighter colored than the ascomata, cylindrical, simple or rarely bifurcate, immersed or projecting over the surface of the substrate, periphysate. ***Peridium*** 10–20 μm, one-layered, composed of cells of *textura angularis* with large lumina. ***Asci*** 95–132 × 18–28 µm, 8-spored, thin-walled, unitunicate, clavate to subcylindrical, persistent, apically somewhat truncate, thickened at the apex, with an apical pore, short-pedicellate. ***Ascospores*** 18–23(–26) × 9–12 µm, hyaline, cylindrical-ellipsoidal, apically rounded, 1-septate, not or slightly constricted at the septum, with a gelatinous appendage at each apex; appendages 1.5–3 µm long, apical or rarely sub-apical, cap-like, round or apiculate, unfurl in water into long filaments. **Asexual morph**. Undetermined.

**Figure 30.**
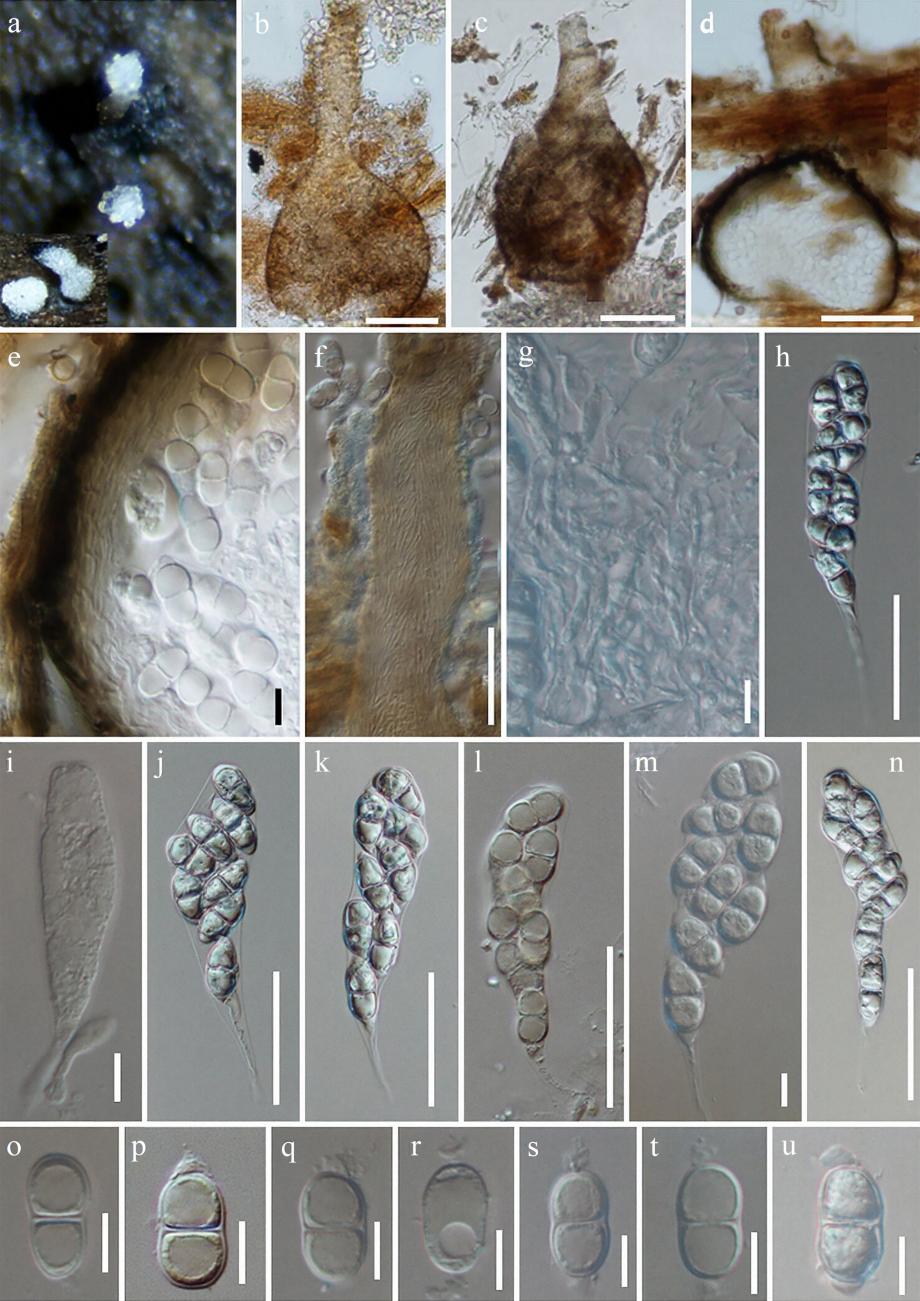
*Neohalosarpheia marina*. **a**. Ascomata superficial to semi-immersed on decaying wood; **b–d**. Ascomata; **e**. Peridium; **f**. Ostiole with periphyses; **g**. Paraphyses; **h, i**. Immature and mature asci; **j–u**. Hyaline ascospores with apical cap. Scale bars: 50 μm (**b–d, f**); 10 μm (**e, g–u**).

##### Material examined.

Australia, • Queensland, Redcliffe, decaying roots of *Avicennia marina*, 27°15'S, 153°04'E, 3 Dec. 1955, A. B. Cribb, holotype; American Samoa, • Pago Pago, 14°22'S, 170°39'W, 1 Dec. 1963, R. A. Barkley (K. 1688); U.s.a., • Florida, Buttonwood Creek near Flamingo, prop root of *Rhizophora mangle*, 25°09'N, 80°55'W, 9 Jan. 1964, J. Kohlmeyer (J.K. 1711b); Liberia, • Mesurado River near Monrovia, prop root of *Rhizophora racemosa*, 6°18'N, 10°49'W, 28 Feb. 1965. J. Kohlmeyer (J. K. 1823).

##### Notes.

Cribb and Cribb (1956) described *Gnomonia marina* from dead roots of *Avicennia marina* from Redcliffe, Queensland, Australia. Later, Kohlmeyer (1984) transferred the taxon to *Halosarpheia* because it has unfurling appendages that is characteristic of the genus. *Halosarpheia marina* differs from *Halosarpheia* species by having asci with thickened apex and with apical pore with active ascospore discharge (Cribb and Cribb 1956).

### *Neptunella* K.L. Pang & E.B.G. Jones

Cribb and Cribb (1956) introduced the species *Gnomonia longirostris* from mangrove wood collected in Queensland, Australia. Later, Kohlmeyer (1984) opined that it was not related to this genus and referred it to *Lignincola*. However, it differs from *Lignincola laevis* (type species) in possessing asci that have an apical pore and plasmalemma which is retracted apically from the ascus wall and the ascospores have a thin exosporial wall layer (Jones 1995). Pang et al. (2003a) revised the genus *Lignincola* based on phylogenetic analysis and found that both *L. laevis* and *L. longirostris* were closely related taxa, although not monophyletic. Hence, a novel genus *Neptunella* typified by *Neptunella longirostris* was erected to accommodate it (Pang et al. 2003a).

#### Neptunella

Taxon classificationFungiMicroascalesHalosphaeriaceae

K K.L. Pang & E.B.G. Jones, Mycol. Progr. 2 (1): 35 (2003)

640A2232-615D-5171-9BDC-6CB5EAE8D2B6

Index Fungorum: IF28749

##### Description.

Saprobic on mangrove wood. **Sexual morph. *Ascomata*** subglobose or bottle-shaped, immersed or semi-immersed, ostiolate, membranous, brownish or hyaline, solitary or gregarious. ***Necks*** periphysate. ***Catenophyses*** present. ***Asci*** unitunicate, 8-spored, ellipsoid-clavate or subfusiform, thin-walled, with short stalk, apically truncated, cytoplasm retracted below the apex, with an apical pore, semi-persistent. ***Ascospores*** ellipsoidal, hyaline, 1-septate, thin-walled, lacking appendages but with a thin ascospore sheath or exosporium. **Asexual morph**. Undetermined.

##### Type species.

*Neptunella longirostris* (Cribb & J.W. Cribb) K.L. Pang & E.B.G. Jones, Mycol. Progr. 2(1): 35 (2003).

##### Notes.

*Neptunella* is a monophyletic genus in the Halosphaeriaceae in a highly supported clade (Fig. 4) distant from *Lignincola*.

##### Molecular evaluation.

In Fig. 4, the two *Neptunella longirostris* strains formed a highly supported clade in the Halosphaeriaceae. Divergence-time analysis places the crown of *Neptunella* in the late Miocene to Pleistocene (~1 MYA, 95% CI: 0.1–6.2 MYA) (Fig. 5).

#### Neptunella longirostris

Taxon classificationFungiMicroascalesHalosphaeriaceae

(Cribb & J.W. Cribb) K.L. Pang & E.B.G. Jones, Mycol. Progr. 2 (1): 35 (2003)

CB5C8E38-F5E2-571A-A55A-2D517C63491A

Index Fungorum: IF373063

Fig. 31

Gnomonia longirostris Cribb & J.W. Cribb, Pap. Dept. Bot. Univ. Queensland 3 (12): 101 (1956). Basionym.
Lignincola longirostris (Cribb & J.W. Cribb) Kohlm., Mar. Ecol. 5 (4): 353 (1984). Synonym.

##### Description.

Saprobic on mangrove wood. **Sexual morph. *Ascomata*** 120–1000 µm high, 120–270 µm diam., subglobose to bottle-shaped, hyaline or brown, membranous, immersed or partly immersed, solitary or gregarious, ostiolate. ***Necks*** 150–650 µm long, 50–70 µm diam., periphyses absent. ***Peridium*** 15–25 µm diam., one stratum, composed of a few layers of elongate cells. ***Catenophyses*** absent. ***Asci*** 40–110 × 12–25 µm, unitunicate, 8-spored, cylindrical-clavate, ellipsoidal-clavate to sub-fusiform, irregularly deliquescing, at first thick–walled apically, becoming thin–walled, with an apical pore, short pedicellate. ***Ascospores*** 17–23 × 4–10 µm, hyaline, elongate-ellipsoidal to irregularly ellipsoidal, 1-septate, slightly constricted at or near the septum, not appendaged. **Asexual morph**. Undetermined.

**Figure 31.**
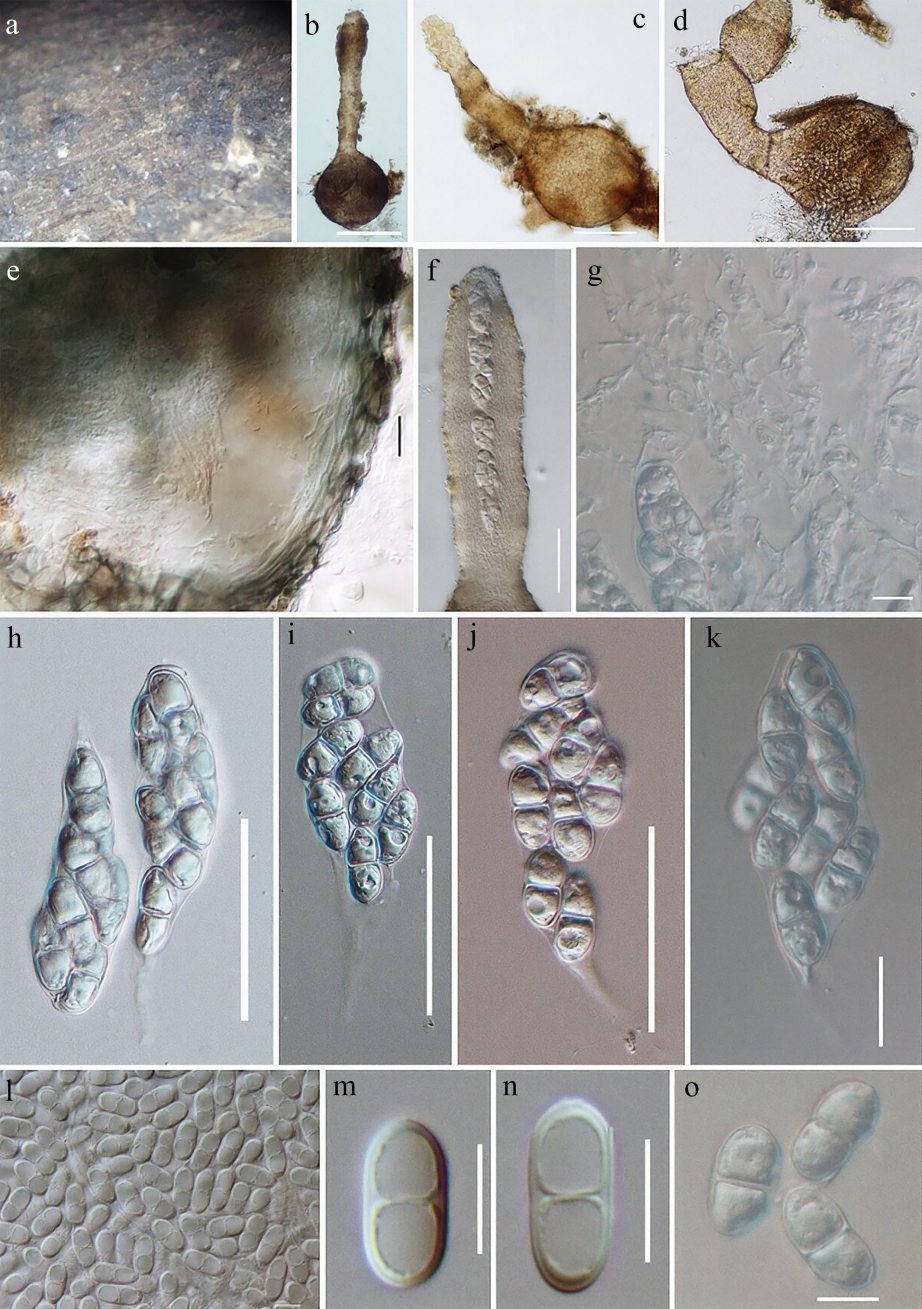
*Neptunella longirostris*. **a**. Ascomata semi-immersed in the decaying wood; **b–d**. Ascomata with long necks; **e**. Peridium with polygonal cells; **f**. Ostiole with periphyses and ascospores dehiscence; **g**. Paraphyses; **h–k**. Asci; **l–o**. Ellipsoidal ascospores. Scale bars: 100 μm (**b–d**); 10 μm (**e, g, l–o**); 50 μm (**f, h–k**).

##### Material examined.

India, • Tamil Nadu, Tiruvarur, Muthupet mangroves, on decaying wood of *Avicennia marina* (Acanthaceae), 8 October 2017, B. Devadatha (AMH-9988). New geographical and substrate record.

##### Notes.

Pang et al. (2003a) showed that *Neptunella longirostris* grouped with *Halosphaeria appendiculata* in a sister clade to *Nohea umiumi* and *N. effusa*. A widely collected taxon, on dead mangrove wood and leaves. *Neptunella longirostris* can be differentiated morphologically from *L. laevis* by: (a) asci having retraction of the plasmalemma and an apical thickening and pore, (b) an extra ascospore wall (exosporium) layer in *N. longirostris* (Jones et al. unpubl.), and (c) asci of *L. laevis* separate from the ascogenous tissue at maturity, are released through the ostiole while still containing the ascospores, and swell in the middle when they enter the water.

##### Distribution.

Australia, Bermuda, Brunei, China, Hong Kong, India, Japan, Malaysia, Mauritius, Philippines, Republic of Trinidad and Tobago, Singapore, Society Islands, South Africa, Taiwan, Thailand, UK (England).

### *Nereiospora* E.B.G. Jones, R.G. Johnson & S.T. Moss

Originally the type species of this genus was *Peritrichospora* (Kohlmeyer 1960) but transferred to *Corollospora* (Kohlmeyer 1962b), as *Peritrichospora* was an invalid name proposed by Barghoorn and Linder (1944) who unaware of the earlier name. *Nereiospora* was introduced by Jones et al. (1983a) to accommodate two species *N. comata* (type species) and *N. cristata* based on a study of the ultrastructure and ontogeny of ascospore appendaged development.

#### Nereiospora

Taxon classificationFungiMicroascalesHalosphaeriaceae

E.B.G. Jones, R.G. Johnson & S.T. Moss, Bot. J. Linn. Soc. 87 (2): 204 (1983)

22EF740F-E73C-5A11-A676-712B1907CC16

Index Fungorum: IF25825

Peritrichospora Linder in Barghoorn and Linder, Farlowia 1: 414 (1944). Synonym.
Corollospora Werdermann Notizbl. Bot. Gart. Berlin, 8: 248 (1922).

##### Description.

Saprobic on wood, bark, and in foam. **Sexual morph. *Ascomata*** solitary and gregarious, subglobose to ellipsoidal, immersed or partly immersed, with or without ostioles, papillate or epapillate, without periphyses, carbonaceous, dark-brown to black. ***Peridium*** six to twelve cell layers, usually thick-walled, black, ellipsoidal, cuboidal to rhomboidal. ***Pseudoparenchyma*** thin-walled, without pit connections and deliquescing at maturity. ***Asci*** fusiform to clavate, pedunculate, unitunicate, 8-spored, thin-walled and deliquescing early. ***Ascospores*** 3–5-spetate, ellipsoidal to fusiform, constricted at the septa, central cells are fuscous and apical cells are hyaline. ***Appendages***, polar and equatorial tufts of rod-like appendages. ***Appendage ontogeny*** outgrowths of the spore wall, initiated prior to septation, rod-like with an outer electron-opaque layer and an electron-transparent core. Spore wall composed of an electron-transparent mesosporium and an electron-opaque episporium continuous beneath the appendages. Exosporium absent. Ascospores and appendages bounded by the membrane complex. ***Histochemistry*** appendage tufts composed of neutral PAS positive carbohydrates (Adapted from Jones et al. 1983a) **Asexual morph**. Hyphomycetous, conidia dark brown to fuscous, basal cell hyaline, muriform, pyriform, oblong ovoid, spherical, slightly constricted at the septa (Mouzouras and Jones 1985).

##### Type species.

*Nereiospora comata* (Kohlm.) E.B.G. Jones, R.G. Johnson & S.T. Moss, Bot. J. Linn. Soc. 87 (2): 206 (1983).

##### Notes.

Originally part of the genus *Corollospora*, members of the genus *Nereiospora* differ in the following aspects: pseudoparenchyma without pit connections; ascospore fuscous with hyaline polar cells; polar spines absent; equatorial and polar appendages are outgrowths of the spore, rod-like, in tufts, and each composed of an outer electron-opaque layer and an electron-transparent core; and secondary exosporial appendages lacking, exosporium absent (Jones et al. 1983a).

The two species are saprobic on intertidal and drifting wood, bark and foam along the shore (Kohlmeyer and Kohlmeyer 1979).

##### Molecular evaluation.

*Nereiospora comata* and *N. cristata* were monophyletic and formed a weakly supported clade with *Havispora longyearbyenensis* (Fig. 4). Both *N. comata* isolates had long branches while no phylogenetic analyses with focus on this genus were found. New isolates should be collected and sequenced.

#### Nereiospora comata

Taxon classificationFungiMicroascalesHalosphaeriaceae

(Kohlm.) E.B.G. Jones, R.G. Johnson & S.T. Moss, Bot. J. Linn. Soc. 87 (2): 206 (1983)

7F723684-C331-5005-AA31-E4EA0542F50D

Index Fungorum: IF108270

Peritrichospora comata Kohlm., Nova Hedwigia 2: 323 (1960). Basionym.
Corollospora comata (Kohlm.) Kohlm., Ber. Deutsch. Bot. Ges. 75: 126 (1962). Synonym.

##### Description.

Saprobic on wood, often associated with sand. **Sexual morph. *Ascomata*** solitary and gregarious, subglobose to ellipsoidal, immersed or partly immersed, with or without ostioles, papillate or epapillate, without periphyses, carbonaceous, dark-brown to black. ***Peridium*** six to twelve layers, cells are usually thick-walled, black, ellipsoidal, cuboidal to rhomboidal. ***Pseudoparenchyma*** is thin-walled, without pit connections and deliquescing at maturity. ***Asci*** fusiform to clavate, pedunculate, unitunicate, 8-spored, thin-walled and deliquescing early. ***Ascospores*** are (32–)35–54 × 12–17 µm typically 5-septate. Ellipsoidal to fusiform, constricted at the septa, central cells are fuscous and apical cells are hyaline. ***Appendages*** polar and equatorial tufts of rod-like appendages. Spore appendage ontogeny: appendages outgrowths of the spore, initiated prior to septation, rod-like with an outer electron-opaque layer and an electron-transparent core. (Adapted from Jones et al. 1983a and Hyde and Sarma 2000). **Asexual morph**. Undetermined.

##### Notes.

*Nereiospora cristata* distinguishes itself from *Nereiospora comata*, by having smaller ascospores (24–38(–41) × 8–16 µm), having fewer septa (typically 3-septate) with the asexual morph *Monodictys pelagica* (Mouzouras and Jones 1985).

##### Distribution.

Canada, Denmark, Germany, Japan, Sweden, UK, USA, fairly frequently collected, and has been recorded from coastal temperate areas of both the eastern and western parts of the North Atlantic Ocean.

### *Nimbospora* Jørg. Koch

Koch introduced the genus *Nimbospora* to accommodate the species *N. effusa*, isolated from intertidal wood (Koch 1982). One of the members of this genus was transferred to a new genus, *Ebullia* based on a phylogenetic study (Chu et al. 2015). Currently *Nimbospora* has two accepted species, the other being *Nimbospora bipolaris* (Fig. 3f).

#### Nimbospora

Taxon classificationFungiMicroascalesHalosphaeriaceae

Jørg. Koch, Nordic J. Bot. 2 (2): 166 (1982)

050EBD8A-A351-525F-BDFE-961122408733

Index Fungorum: IF3509

##### Description.

Saprobic on decayed intertidal wood. **Sexual morph. *Ascomata*** solitary or gregarious, immersed, partly immersed or superficial, globose to subglobose, ostiolate, hyaline. ***Peridium*** membranous, pseudoparenchymatous, surface cells angular, inner cells large, thin walled. ***Asci*** thin-walled, clavate, deliquescing at maturity, 8-spored. ***Ascospores*** ellipsoidal, 1-septate, surrounded by an eccentrically developed sheet and laterally bearing a mucilaginous appendage (Adapted from Koch, 1982). **Asexual morph**. Undetermined.

##### Type species.

*Nimbospora effusa* Jørg. Koch, Nordic J. Bot. 2 (2): 166 (1982).

##### Notes.

The type was originally isolated from strongly decayed intertidal wood from the Indian Ocean (Sri Lanka) (Koch 1982), and has been isolated from Hawaii (Pacific Ocean) and *N. bipolaris* was isolated from Hawaii, as well as Brunei (Pacific Ocean) (Kohlmeyer and Volkmann-Kohlmeyer 1989; Fryar et al. 2004).

##### Molecular evaluation.

*Nimbospora effusa* and *N. bipolaris* formed a monophyletic group, sistering with *Neohalosarpheia marina* (Fig. 4). Divergence-time estimates place the split between *Nimbospora* and *Neohalosarpheia* in the late Paleogene (~38 MYA, 95% CI: 29.9–46.4MYA), supporting their recognition as distinct genera within the Halosphaeriaceae (Fig. 5).

#### Nimbospora effusa

Taxon classificationFungiMicroascalesHalosphaeriaceae

Jørg. Koch, Nordic J. Bot. 2 (2): 166 (1982)

80CD719C-0718-518F-BA0F-8CB8DBDA4CAC

Index Fungorum: IF110841

Figs 1c, 3a

##### Description.

Saprobic on decayed intertidal wood. **Sexual morph. *Ascomata*** solitary or gregarious, immersed, partly immersed or superficial, globose to subglobose 180–225 × 225–240 µm, ostiolate, hyaline. ***Peridium*** membranous, pseudoparenchymatous, surface cells angular, 2–3 outer cells with slightly thickened walls, inner cells thin-walled gradually enlarging becoming large and inflated. ***Necks*** cylindrical, often bifurcate or multifurcate, periphysate, length up to 750 µm, width 70–90 µm. ***Asci*** at different stages of maturity in hymenium in venter of ascocarp, thin-walled, broadly clavate, deliquescing at maturity, 8-spored, 64–84 × 32–44 µm. ***Ascospores*** 1-septate, ellipsoidal, slightly constricted around septum, appendaged, hyaline, 17.0–20.5–24.0 × 8.0–8.5–9.5 µm (60 spores). ***Appendages*** of two types: 1).one is eccentrically placed, more or less an inflated sheet surrounding the spore, in young stages smooth later slightly folded and wavy except in terminal parts, diameter at spore ends up to 9 µm and at septum up to 14 µm, 2). the other is a lateral mucilaginous appendage at first a cushion, later developing into thick and thin strands up to 15 µm extending from the side of the spore. When fresh spores are mounted in water, the lateral mucilaginous appendage will mostly unfold out in more or less, apparently stiff, slightly curved strands (Adapted from Koch 1982). **Asexual morph**. Undetermined.

##### Notes.

*Nimbospora effusa* clearly belongs in the Halosphaeriaceae because it possesses deliquescing asci, appendaged ascospores and is marine (Koch 1982).

### *Nohea* Kohlm. & Volkm.-Kohlm.

The genus *Nohea* was established by Kohlmeyer and Volkmann-Kohlmeyer (1991b) with *N. umiumi* as the type species. Two further species of *Naufragella*, *N. spinibarbata* and *N. delmarensis* have been transferred to this genus based on morphology and molecular phylogeny (Abdel-Wahab 2011b). However, molecular studies conducted by Sakayaroj et al. (2011) and Chu et al. (2015) did not support this conclusion. Jones et al. (2015) considered the transfer of *Naufragella delmarensis* and *N. spinibarbata* to *Nohea* as doubtful. *Nohea* is characterised by ascospores with two types of appendages: (a) gelatinous, forming a band or veil at each apex and (b) a crown of delicate filaments below each apex (Kohlmeyer and Volkmann-Kohlmeyer 1991b). *Nohea spinibarbata* is transferred back to *Naufragella* in this study. The genus was referred to the Halosphaeriaceae by Kohlmeyer and Volkmann-Kohlmeyer (1991b) and this was confirmed by sequence data (Jones et al. 2009; Abdel-Wahab 2011b).

#### Nohea

Taxon classificationFungiMicroascalesHalosphaeriaceae

Kohlm. & Volkm.-Kohlm., Syst. Ascomycetum 10: 121 (1991)

20D07236-6A42-58F5-9CE5-388A98E53811

Index Fungorum: IF25883

##### Description.

Saprobic on intertidal wood. **Sexual morph. *Ascomata*** subglobose, immersed to superficial, ostiolate, papillate, coriaceous, cream colored, solitary. ***Peridium*** thin-walled, forming *textura angularis*. ***Catenophyses*** present, deliquescing early. ***Asci*** 8-spored, clavate, pedunculate, unitunicate, thin-walled, deliquescing. ***Ascospores*** ellipsoidal, one-septate, hyaline, subapically and laterally attached appendages that gelatinize into delicate filamentous threads, also two lateral narrow thin group of hairs. **Asexual morph**. Undetermined.

##### Type species.

*Nohea umiumi* Kohlm. & Volkm.-Kohlm., Syst. Ascomycetum 10: 122 (1991).

##### Notes.

*Nohea* (*N. umiumi*) is characterized by having two kinds of appendages; a subapical gelatinous pad wrapped around the equator and unfurls in water to form long sticky filaments and two bundles of fibers attached to the spore wall at polar positions (Kohlmeyer and Volkmann-Kohlmeyer 1991b). Morphologically, the genus *Naufragella* is similar to *Nohea* in having two types of ascospore appendages: gelatinous and fibrous. However, the two genera differ only in the position of the ascospore appendages. *Naufragella* has apical gelatinous appendages and a subapical crown of filaments at each apex below the gelatinous appendages (Kohlmeyer and Volkmann–Kohlmeyer 1998; Abdel-Wahab 2011b). Abdel-Wahab (2011b) transferred the two taxa in *Naufragella*, *N. delmarensis* and *N. spinibarbata* to *Nohea*. These two species are closely similar and differ only in ascospore measurements and appendage shape and length. In this monograph, *Nohea spinibarbata* is best referred to *Naufragella*, as it also grouped in a sister clade to the *Nohea* clade, albeit with unsupported sequence data.

*Nohea* species have been reported on submerged intertidal wood, driftwood, bark and bamboo submerged in marine waters (Kohlmeyer and Volkmann-Kohlmeyer 1991b, 1998; Abdel-Wahab 2011b).

##### Molecular evaluation.

*Nohea umiumi* and *N. delmarensis* formed a monophyletic group with a good support (Fig. 4 ). Taxa related were *Tinhaudeus formosanus* and *Naufragella spinibarbata*.

#### Nohea umiumi

Taxon classificationFungiMicroascalesHalosphaeriaceae

Kohlm. & Volkm.-Kohlm., Syst. Ascomycetum 10: 122 (1991)

CDAE4972-7A9C-5639-BFA3-C7E5F34E0E56

Index Fungorum: IF355162

Fig. 3b

##### Description.

Saprobic on submerged wood in aquatic habitats. **Sexual morph. *Ascomata*** 160–300 µm diam., subglobose, cream-colored, coriaceous, solitary, superficial or immersed, ostiolate, papillate. ***Necks*** 50–300 µm long, 40–90 µm diam., cylindrical or conical, periphysate. ***Peridium*** 4–6 layers, 12–19 µm diam., composed from elongated cells, forming a *textura angularis*. ***Asci*** 60–75 × 22–28 µm, unitunicate, 8-spored, clavate, pedunculate, thin-walled, deliquescing at maturity. ***Catenophyses*** present. ***Ascospores*** 17–23 × 8–10.5 µm, hyaline, ellipsoidal, 1-septate, with two types of appendages. ***Appendages*** on one side of the ascospore, 1.1–1.7 µm thick, gelatinous, attached to flat, refractive, subapical pads, with tips wrapped around the equator of the spore, unfurling in water to form long sticky filaments. On the other side of the ascospore are two bundles of fibers, attached to the wall below the apices; fibers at first joined together, lying flat against the wall and directed toward the tip; in water spreading out and forming tufts of hair-like appendages. **Asexual morph**. Undetermined.

##### Distribution.

Sarushima Island, Japan. USA (Hawaiian Islands).

##### Notes.

Molecular data placed *N. umiumi* in the Halosphaeriaceae grouping with *Aniptodera* species and *Nimbospora effusa* (Spatafora et al. 1998; Campbell et al. 2003), and formed a sister group to *Neptunella longirostris* (Jones et al. 2009, 2017).

### *Oceanitis* Kohlm.

*Oceanitis* was introduced by Kohlmeyer (1977) as a monotypic genus to accommodate the species *Oceanitis scuticella* and currently includes five species: *Oceanitis scuticella*, *O. cincinnatula* (=*Halosarpheia cincinnatula*), *O. unicaudata* (= *Haligena unicaudata*), and *O. viscidula* (= *Haligena viscidula*) and *O. abyssalis* (Nagano et al. 2024).

#### Oceanitis

Taxon classificationFungiMicroascalesHalosphaeriaceae

Kohlm., Rev. Mycol. (Paris) 41 (2): 193 (1977)

E31A680E-88F7-5399-A538-EE3A9CAABF59

Index Fungorum: IF532552

##### Description.

Saprobic on dead wood. **Sexual morph. *Ascomata*** gregarious, subglobose to ellipsoidal, coriaceous, may be dimorphic, ostiolate, periphysate, epapillate, fleshy, brownish to dull orange-colored. ***Peridium*** thick, forming a *textura angularis*. ***Paraphyses*** absent; the center of immature ascocarps is filled with thin-walled pseudoparenchymatous cells. ***Asci*** 8-spored, clavate, unitunicate, thin-walled, ripening successively on ascogenous tissue at the bottom of the ascocarp venter, becoming detached at maturity at the base and pushed upward by young asci, eventually dissolving in the upper part of the ascocarp venter. ***Ascospores*** filamentous, 1-septate, hyaline, with a filiform apical appendage (based on Kohlmeyer and Kohlmeyer 1979). **Asexual morph**. Undetermined.

##### Type species.

*Oceanitis scuticella* Kohlm., Rev. Mycol. (Paris) 41 (2): 194 (1977).

##### Notes.

*Oceanitis* is morphologically similar to other halosphaeriaceous species which possess ascospores with a single polar appendage, e.g. *Moana*, *Ophiodeira*, *Tirispora*, *Trichomaris*, but differ morphologically and phylogenetically.

Species of *Oceanitis* are saprobic on submerged decayed wood both from shore water and the deep-sea.

##### Molecular evaluation.

In Fig. 4, *Oceanitis* species formed a highly supported clade with *Ophiodeira monosemeia*. Divergence-time analysis suggests that this relationship dates back to the mid-Cretaceous (~105 MYA, 95% CI: 26.6–414 MYA), supporting the recognition of *Oceanitis* as a distinct evolutionary lineage within the Halosphaeriaceae (Fig. 5).

#### Oceanitis scuticella

Taxon classificationFungiMicroascalesHalosphaeriaceae

Kohlm., Rev. Mycol. (Paris) 41 (2): 194 (1977)

614B3A20-705C-5D55-965A-EE11615217C0

Index Fungorum: IF532553

Fig. 32

##### Description.

Saprobic on wood. **Sexual morph. *Ascomata*** 1.4–2.03 µm high, 1.4–1.78 µm in diam., sub globose to ellipsoidal, round at the top, flat at the base, stalked or aggregated in a stroma, hypostroma 30–135 µm, ostiolate, papillate, fleshy, brown to dull orange-colored, gregarious. ***Hypostroma*** is 30–120 µm thick, light-colored, composed of small, polygonal to rounded cells with large lumina; masses of lilac-colored hyphae can be found in the wood vessels under the stromata. ***Peridium*** is 450–470 µm thick at the apex, 240–260 µm at the sides, composed of 17–35 layers of hyaline, thick-walled, polygonal, isodiametric cells with large lumina, forming a *textura angularis*; merging toward the center into flattened cells; outer 3–7-cells filled with small yellowish globules. ***Ostioles*** 25–30 µm in diam., periphysate. ***Paraphyses*** absent. Ascomata filled with hyaline, thin-walled, polygonal, isodiametric, pseudoparenchymatous cells, which eventually compressed by the asci. ***Asci*** are 70–90 × 12–17 µm, 8-spored, clavate, unitunicate, thin-walled, without apical apparatus, deliquescing. ***Ascospores*** 60–80 × 4–6 µm, filiform to elongate fusiform, 1-septate, hyaline, with one apical appendage: appendages 32–50 µm long, 2(-3) µm in diam. at the base, filamentous, tapering toward the apex, wavy, whip-like, hamate, closely adpressed to the spore wall, becoming detached from the wall, but adhering with its base to the apex of the ascospore (Based on Kohlmeyer and Kohlmeyer 1979; Dupont and Schwabe 2016). **Asexual morph**. Undetermined.

**Figure 32.**
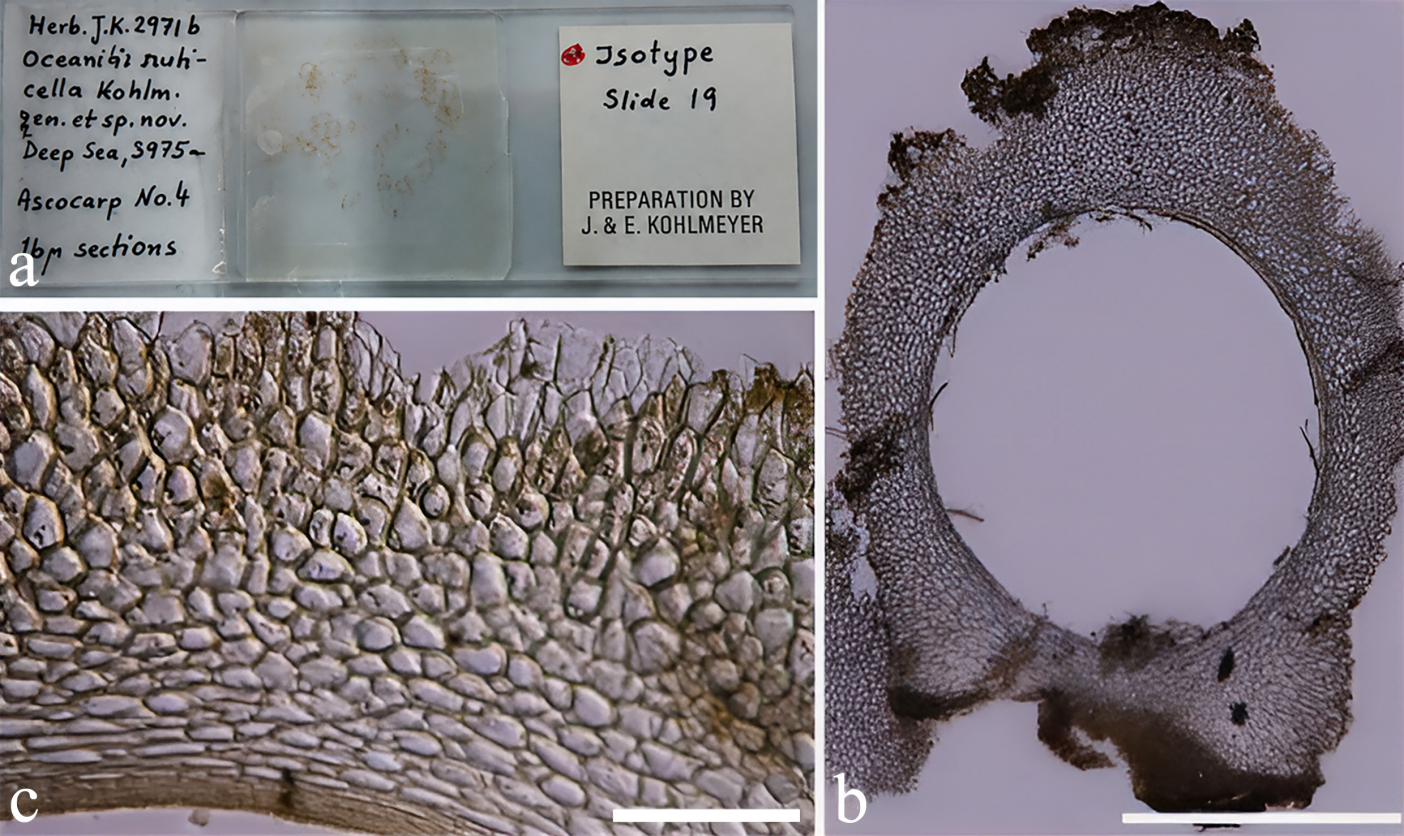
*Oceanitis scuticella* (NY 1348289, isotype). **a**. Herbarium material of *Oceanitis scuticella*; **b**. Section through ascomata; **c**. Peridium. Scale bars: 20 μm (**b**); 10 μm (**c**).

##### Notes.

**Figure 33.**
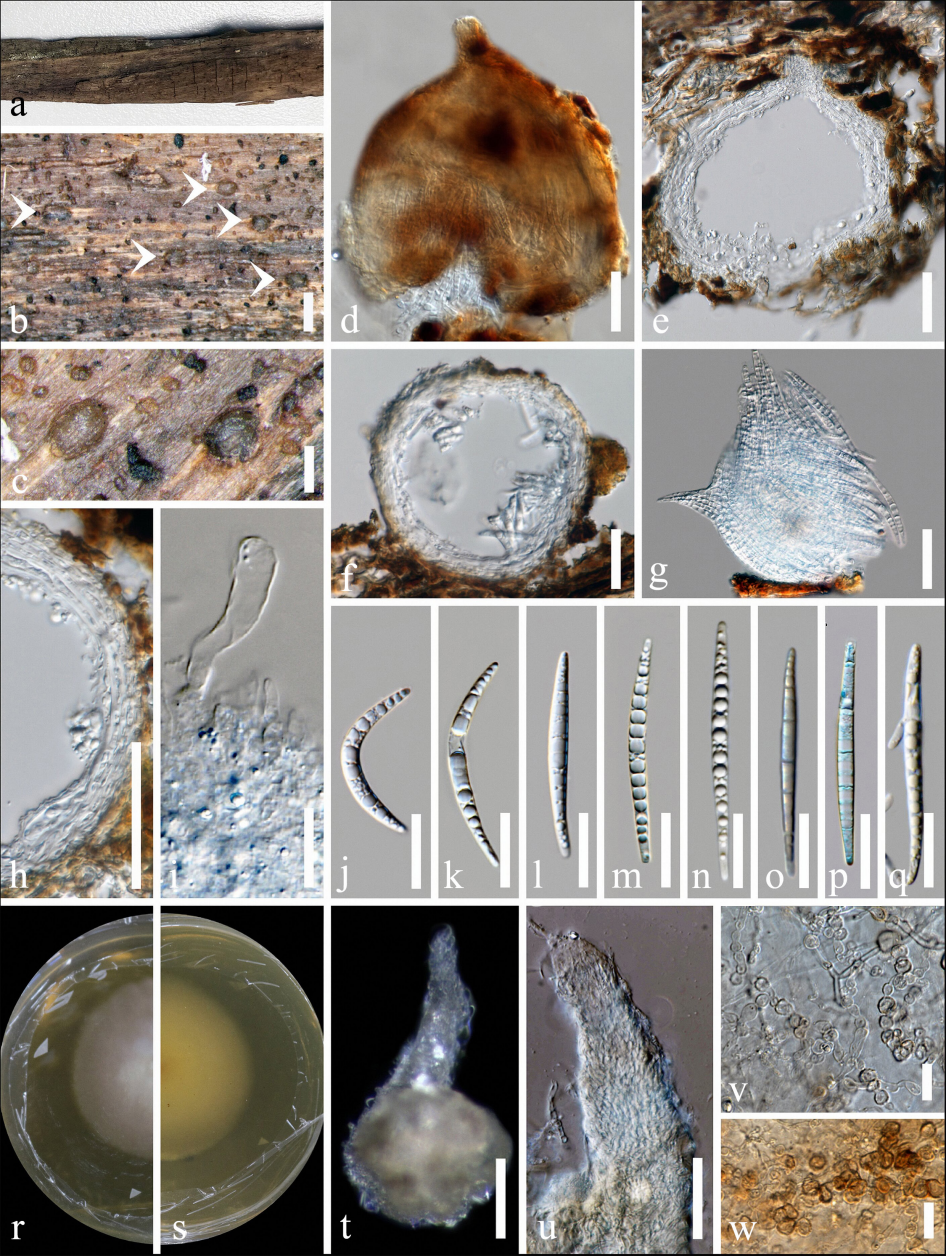
*Oceanitis cincinnatula*. **a–c**. Herbarium material; **d**. Ascoma; **e, f**. Section of ascomata; **g**. Mass of released ascospores; **h**. Enlarged ascoma wall; **i**. Hamathecium; **j–q**. Septate ascospores; **r–s**. Front/back of culture. *Oceanitis* sp.; **t**. Hyaline perithecium; **u**. Close up of perithecial neck; **v–w**. Chlamydospores. Scale bars: 10 µm (**b, c, j–q**); 25 µm (**d**); 20 μm (**e–g**); 1 µm (**h, i**); 100 µm (**t**); 5 µm (**u–w**).

*Oceanitis scuticella* is characterised by the thick-walled ascomata which are dimorphic (stalked or aggregated in a stroma), seated on a thin hypostroma on the surface of the woody substrate (this is not a feature of other *Oceanitis* species) and ascospores with a single polar appendage, similar to that of *Halosarpheia* species. There is no evidence that the appendage unfurls to form long thread-like filaments, as in other *Oceanitis* species (Fig. 33). No asci and ascospores were observed in the holotype. *Oceanitis scuticella* differs from other *Oceanitis* species by the thick-walled ascomata, 1-septate ascospores and occurring in deep waters.

Dupont and Schwabe (2016) regard it as a true abyssal species as it has been reported from three deep water geographical areas: Atlantic Ocean: 3975 m depth; Pacific Ocean: 5229–5217 m depth; South China Sea 1205–1397 m depth.

##### Distribution.

Angola (Vanuatu archipelago, Pacific Ocean), South China Sea (Taiping Island, and Dongsha Island), Kuril-Kamchatka Trench (Pacific Ocean, Kamchatka Peninsula, the Kuril Islands, and Hokkaido island, Japan).

### *Ocostaspora* E.B.G. Jones, R.G. Johnson & S.T. Moss

*Ocostaspora* was introduced by Jones et al. (1983c) to accommodate *Ocostaspora apilongissima*, a monophyletic genus. Initially introduced by Johnson and Cavaliere (1963) as *Remispora ornata* on rotten wood of *Arbutus menziesii* (Washington State, USA), subsequently referred to *Halosphaeria appendiculata* (Kohlmeyer and Kohlmeyer 1979). However, new collections showed that ascospore appendage development was different from the type species of *H. appendiculata* (Jones et al. 1983c) and it was transferred to the new genus *Ocostaspora*. Sakayaroj et al. (2011), in a subsequent phylogenetic study of the family Halosphaeriaceae, confirmed that it did not group with the type species *H. appendiculata*.

#### Ocostaspora

Taxon classificationFungiMicroascalesHalosphaeriaceae

E.B.G. Jones, R.G. Johnson & S.T. Moss, Bot. Mar. 26: 353 (1983)

D04156C5-6631-5393-BB2D-FEDC69B5AF22

Index Fungorum: IF25829

##### Description.

Saprobic on wood. **Sexual morph. *Ascomata*** globose to subglobose, hyaline, exposed parts dark brown to black, solitary sometimes gregarious, immersed to partly immersed in the substrate, membranous, ostiolate, papillate, and lacking periphyses. ***Peridium*** 4–5 layers of elongate, thin-walled cells. ***Asci*** 8-spored, clavate to subcylindrical, apiculate, pedunculate, unitunicate, thin-walled, deliquescing early, asci formed on a cushion of pseudoparenchymatous cells at the base of the ascocarp. ***Ascospores*** ellipsoidal, 1-septate, hyaline and appendaged. ***Appendages*** polar and equatorial, which arise by fragmentation of the exosporium, and histochemically positive stained for protein and neutral carbohydrates. **Asexual morph**. Undetermined. (Description based on Jones et al. 1983c).

##### Type species.

*Ocostaspora apilongissima* E.B.G. Jones, R.G. Johnson & S.T. Moss, Bot. Mar. 26: 354 (1983).

##### Notes.

*Ocostaspora* is most similar to the type species of the genus *Halosphaeria* (*H. appendiculata*), with its polar and equatorial spoon-shaped appendages. *Halosphaeria appendiculata* has larger ascospores and the polar and equatorial appendages are of the same length, however the main difference is in the ontogeny of the spores and appendages. In *Halosphaeria* the appendages arise as outgrowths of the ascospore wall, and the exosporium is absent, while in *Ocostaspora* the appendages are formed by the fragmentation of the exosporium (Jones et al. 1983c). In this monograph, we introduce the new species *Ocostaspora japonica* with two polar and two equatorial appendages.

##### Molecular evaluation.

Phylogenetically, *O. apilongissima* formed a highly supported clade with *Kochiella* species (Sakayaroj et al. 2011; Fig. 4), but are morphologically distinct with polar appendages only in *Kochiella*.

#### Ocostaspora apilongissima

Taxon classificationFungiMicroascalesHalosphaeriaceae

E.B.G. Jones, R.G. Johnson & S.T. Moss, Bot. Mar. 26: 354 (1983)

7D0589E7-5EFA-5F85-A06A-BD29D372DBEC

Index Fungorum: IF108277

##### Description.

Saprobic on wood. **Sexual morph. *Ascomata*** 87–285 µm high, 87–270 µm wide, globose to subglobose, hyaline with short neck and upper part of centrum dark brown to black, solitary or gregarious, immersed to partly immersed in the substrate, membranous, ostiolate, papillate, and lacking periphyses. ***Peridium*** 4–5 layers, cells thin-walled, elongate, cells 25–98 µm long and 25–86 µm wide. ***Asci***: 46.5–54 × 10–12.3 µm, 8-spored, unitunicate, thin-walled, clavate to subcylindrical, short pedunculate, lacking apical pore and deliquescing early. ***Paraphyses*** absent. ***Asci*** formed on a cushion of pseudoparenchymatous cells at the base of ascomata. ***Ascospores*** 15–22 × 5–7.4 µm, ellipsoidal, 1-septate, not or slightly constricted at the septum, hyaline and appendaged. *Appendages* polar appendages 10–49 µm long, awl-like up to 7.4 µm wide and tapering, 6–8 equatorial appendages awl-like 2.4–8 µm long. **Asexual morph**. Undetermined. (Description based on Jones et al. 1983c).

##### Notes.

The type strain was isolated from driftwood collected at Eagle Cove, San Juan Island. Other isolates were obtained from a broad spectrum of locations, from both temperate (Canada, Sweden, USA) and tropical (Australia, India, Mexico, Seychelles) climates (https://www.marinefungi.org).

##### Distribution.

Denmark, Spain, Sri Lanka, UK (England), USA (Friday Harbour, Maine).

#### Ocostaspora japonica

Taxon classificationFungiMicroascalesHalosphaeriaceae

Abdel-Wahab & E.B.G. Jones sp. nov.

8EBB7697-BF4C-5375-B117-BB503023C8A6

Index Fungorum: IF904210

Fig. 34

##### Etymology.

From Japan where the fungus was collected.

**Figure 34.**
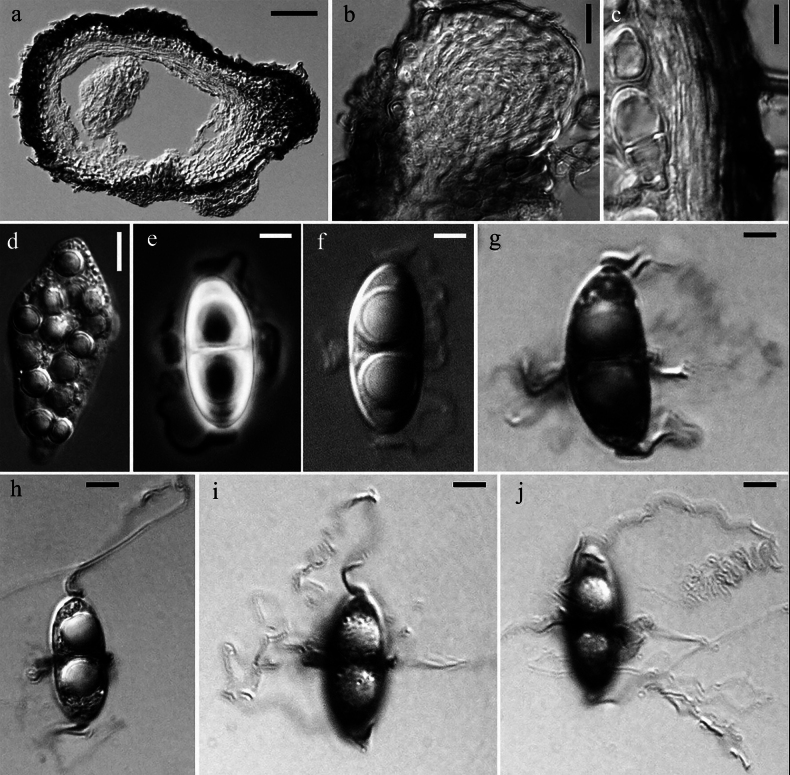
*Ocostaspora japonica* (CBS-H-23863, holotype). **a**. Vertical section of the ascomata; **b**. Magnified part of a vertical section of the neck shows the pseudoparenchymatous cells that fill the ostiolar canal; **c**. Magnified vertical section of the peridium shows the two-layered wall that form *textura-angularis*; **d**. Immature ascus; **e–j**. Variously shaped ascospores with polar and equatorial appendages (**g–j**. Stained in Toluidine blue). Scale bars: 40 μm (**a**); 15 μm (**b, c**); 10 μm (**d**); 5 μm (**e–j**).

##### Description.

Saprobic on decaying driftwood in the intertidal zone in marine habitat. **Sexual morph. *Ascomata*** 140–260 μm high, 105–180 μm wide, subglobose to pear-shaped, horizontal, yellow-brown to reddish-brown upper side, hyaline base, immersed to erumpent, single, membranous, ostiolate and papillate. ***Necks*** 56–110 μm long, 30–45 μm wide, lateral, bending upward, hyaline to light-brown in color, cylindrical, ostiolar canal filled with polygonal pseudoparenchymatous cells. ***Peridium*** 10–38 μm thick, two-layered, forming *textura angularis*; outer layer 8–25 μm thick. ***Catenophyses*** absent. ***Asci*** 64 × 31 μm, 8-spored, clavate, thin-walled, deliquesce early, without an apical apparatus. ***Ascospores*** 16–19 × 7–8 μm (mean = 17.6 × 7.8 μm, n=50), ellipsoidal, 1-septate, not constricted at the septum, hyaline, with bipolar apical appendages, with wide base and tapering tip, 10–22 μm long, 1–2.5 μm wide, two equatorial appendages 5–12 μm long, 1–2.5 μm wide. Polar and equatorial appendages rapidly expand in water into long, thin filaments. **Asexual morph**. Unknown.

##### Culture characteristics.

Single spore isolates of *Ocostaspora japonica* growing on PDA are light brown with tufts of aerial mycelium, reaching a 25–30 mm radius after one month at 25 °C. No sporulation structures were observed.

##### Material examined.

Japan, • Sarushima Island, decaying driftwood, 28^th^ April 2008, M.A. Abdel-Wahab, holotype CBS-H-23863. Ex-type living culture: NBRC 105291.

##### Notes.

Abdel-Wahab (2011b) documented 27 marine fungi from decaying driftwood collected from the intertidal zone of Sarushima Island, Japan. Among the identified 27 taxa, seven were possibly new to science, and of those seven taxa, *Savoryella sraushimana* was recently described (Zhang et al. 2019). In this paper, we describe a second species, *Ocostaspora japonica* (listed as *Morakotiella* sp. NBRC 105291), which had a 2.2% frequency of occurrence. *Ocostaspora japonica* can be placed in the genus *Ocostaspora* because it shares morphological characters with the type species, *O. apilongissima*, by having membranous ascomata with hyaline immersed part and yellow-brown to dark-brown upper part with non-periphysate neck; no catenophyses; asci are deliquescing early and ascospores with long polar and short equatorial appendages with spoon-shaped bases to the appendages. Both species were phylogenetically related and formed a monophyletic clade with *Kochiella crispa*, *Morakotiella salina*, *Neoaniptodera juncicola* and *Remispora hamata* (Fig. 4). The identity of the *R. hamata* CBS 145.60 that was deposited by Jan Kohlmeyer on 4 December 1959 is questionable as the morphology of the isolate is unknown and it is not quoted in any publication. Also, Kohlmeyer and Volkmann-Kohlmeyer (1991a) rejected the taxon *Remispora hamata* because there is no type material of the fungus and stated that the collection most probably belongs to the genus *Halosarpheia*. Furthermore, they regarded that the original protologue and illustrations given by Höhnk (1955) was not sufficient to differentiate it from other taxa and it must be considered a nomen dubium. *Remispora hamata*-like taxa might represent a complex of species with unfulring polar appendages in the temperate region.

*Ocostaspora japonica* differs from *O. apilongissima* by having two equatorial appendages while the later species have 6 to 8 awl-like equatorial appendages. Polar appendages in *O. apilongissima* are awl-like that are much longer (10–49 µm) and wider (up to 7.4 µm) than polar appendages in *O. japonica* which are shorter and narrower (10–22 μm long, 1–2.5 μm wide).

### *Okeanomyces* K.L. Pang & E.B.G. Jones

A genus created to accommodate *Halosphaeria cucullata* based on its morphological differences and phylogenetic disparity with *Halosphaeria appendiculata*, type species of *Halosphaeria* (Pang et al. 2004a). A second species, *Okeanomyces marinus*, is an asexual species later added to the genus (Hyde et al. 2020a), while *Okeanomyces guttulatus* has been described from sediments in Guangdong, China (Li et al. 2023).

#### Okeanomyces

Taxon classificationFungiMicroascalesHalosphaeriaceae

K.L. Pang & E.B.G. Jones, Bot. J. Linn. Soc. 146 (2): 228 (2004)

3C179192-B2C2-53A8-8343-C63494C912FC

Index Fungorum: IF28838

##### Description.

Saprobic on wood. **Sexual morph. *Ascomata*** subglobose or ellipsoidal, immersed or semi-immersed, brown or black, coriaceous or subcarbonaceous. ***Periphyses*** absent. ***Asci*** clavate, with short peduncles, thin-walled, early deliquescing. ***Catenophyses*** present. ***Ascospores*** cylindrical, thin-walled, with or without a single cap-like appendage. **Asexual morph. *Conidiophores*** hyphomycetous, cylindrical, septate, simple or branched, hyaline, often forming pustules on the surface of the substrate. ***Conidiogenous cell*** ellipsoidal or ovoid, hyaline, produced acrogenously. ***Conidia*** one-celled, subglobose or ovoid, smooth, thick-walled, light brown with a reddish tint or dark brown, developing basipetally, catenulate, cells finally separating (asexual stage *Periconia prolifica*).

##### Type species.

*Okeanomyces cucullatus* (Kohlm.) K.L. Pang & E.B.G. Jones, Bot. J. Linn. Soc. 146 (2): 228 (2004).

##### Notes.

Kohlmeyer (1972) revised the taxonomy of various marine genera, synonymizing *Remispora* under *Halosphaeria*, thus *Remispora cucullata*, introduced by Kohlmeyer (1964), was referred to *Halosphaeria*. Johnson et al. (1984) in an ultrastructural study of ascospore appendage ontogeny, accepted the genus *Remispora*. Subsequently, based on sequence data, *R. cucullata* did not group within *Remispora* and the genus *Okeanomyces* was introduced to accommodate *R. cucullata* (Pang et al. 2004a). The sexual/Asexual connection (*Periconia prolifica*) was made by Pang et al. (2004a) based on cultural and molecular evidence. Currently, there are three species in the genus: the cosmopolitan species *O. cucullatus*, *O. marinus* and *O. guttulatus* with a restricted distribution in Thailand and China, respectively.

Species of the genus are saprobic on wood and in sediment (Kohlmeyer 1964; Pang et al. 2004a; Hyde et al. 2020a).

##### Molecular evaluation.

Pang et al. (2004a) found that *Okeanomyces* was related to *Aniptodera* and *Nais* based on partial 28S rDNA sequences. After additional species/genes of the Halosphaeriaceae became available, *Okeanomyces* was found to be related to *Thalespora* phylogenetically based on a combined analysis of the 18S and 28S rDNA (Hyde et al. 2020a). In Fig. 4, *Okeanomyces* species also formed a highly supported clade with *Thalespora appendiculata*. Divergence-time estimates indicate that *Okeanomyces* and *Thalespora* separated during the Paleogene–Neogene interval (~10 MYA, 95% CI: 2.5–43.7 MYA), supporting their recognition as distinct genera within the Halosphaeriaceae (Fig. 5).

#### Okeanomyces cucullatus

Taxon classificationFungiMicroascalesHalosphaeriaceae

(Kohlm.) K.L. Pang & E.B.G. Jones, Bot. J. Linn. Soc. 146 (2): 228 (2004)

31CA1C6D-E751-5F14-8512-FD71CA6E4C02

Index Fungorum: IF369437

Remispora cucullata Kohlm., Mycologia 56: 770 (1964). Basionym.
Halosphaeria cucullata (Kohlm.) Kohlm., Canad. J. Bot. 50 (9): 1956 (1972). Synonym.
Periconia prolifica Anastasiou, Nova Hedwigia 6 (3–4): 260 (1963).

##### Description.

Saprobic on wood. **Sexual morph. *Ascomata*** 150–245 high, 150–250 µm in diam., subglobose or ampulliform, immersed, ostiolate, papillate, coriaceous, brownish-black or brownish-red, solitary or gregarious. ***Peridium*** 7.5–11.5 µm thick, composed of four to six layers of thick-walled, mostly ellipsoidal cells, forming a *textura angularis*, merging toward the center into the pseudoparenchyma. ***Necks*** 55–100 µm long, 28–75 µm in diam., cylindrical or conical; ostiolar canal at first filled with a small-celled pseudoparenchyma. ***Pseudoparenchyma*** of thin-walled, polygonal or rounded cells filling venter of young ascomata; eventually breaking up into catenophyses; cells 9–23 µm, 6–15 µm in diameter. ***Asci*** 45–70 × 15–30 µm, 8-spored, clavate, short pedunculate, unitunicate, thin-walled, early deliquescing, developing at the base of the ascoma venter. ***Ascospores*** 20–68.5 × 6–11.5 µm, cylindrical or rarely ellipsoidal, 1-septate, upon germination often becoming two- to four-septate, slightly or not constricted at the septa, hyaline, with a cap-like, subglobose, terminal, deciduous appendage at one end, 5–5.8 µm in diameter. **Asexual morph. *Conidiophores*** 5–200 × 2.5 μm, cylindrical, septate, simple or branched, hyaline, often forming pustules on the surface of the substrate. ***Conidiogenous cell*** ellipsoidal or ovoid, hyaline, produced acrogenously. ***Conidia*** 6–13(–20) μm in diam., one-celled, subglobose or ovoid, smooth, thick-walled, light brown with a reddish tint or dark brown, developing basipetally, catenulate, cells finally separating (*Periconia prolifica*).

##### Notes.

*Okeanomyces cucullatus* is a widely collected species (as the asexual morph) on a variety of substrata. The sexual stage of *O. cucullatus* is always associated with the mangrove environment, while *P. prolifica* can be found in a wide range of marine habitats (Pang et al. 2004a). Germination of ascospores of *O. cucullatus* always results in its asexual stage (*P. prolifica*) (Pang et al. 2011b).

##### Distribution.

*Okeanomyces cucullatus* is a widely collected species with records from: Australia, Bahamas, Belize, Brazil, Brunei, Great Abaco, Guatemala, Hawaii, India, Japan, Malaysia, Mauritius, Mexico, South Africa, Thailand, United States. The asexual stage *Periconia prolifica* is widely documented from tropical locations: Bahamas, Bermuda, Brazil, Belize, East Mexico, US (Florida, Hawaii), Ghana, Martinique, Sierra Leone, Republic of Trinidad and Tobago, Andaman Islands, India (Andhra Pradesh, Lakshadweep Islands, Maharashtra, Tamil Nadu), East South Africa, Indonesia (Java, Sumatra), Maldives, Mauritius, Nicobar Islands, Seychelles, Kuwait, Saudi Arabia, Brunei, China (Hong Kong, Shenzhen), Malaysia, Australia (New South Wales, Queensland, Victoria), Philippines, Singapore, Society Islands, Thailand, West Guatemala, West Mexico, and Taiwan.

#### Okeanomyces marinus

Taxon classificationFungiMicroascalesHalosphaeriaceae

Calabon, E.B.G. Jones, Boonmee & K.D. Hyde, Fungal Diversity 103: 246 (2020)

1184BDB7-99FF-5615-BC14-6DABE4991119

Index Fungorum: IF557251

##### Description.

Saprobic on decaying wood submerged in intertidal rocky shore. **Sexual morph**. Undetermined. **Asexual morph**. Colonies on the substratum superficial, dark brown to black. Conidiophores reduced to conidiogenous cells. Conidiogenous cells erect, aggregated in clusters on hyphae, hyaline to brown, smooth, spathulate to ampulliform. Conidia 7–15 × 7–11 µm, globose, subglobose, irregular, hyaline to brown, thick and smooth-walled.

##### Distribution.

Thailand.

##### Notes.

*Okeanomyces marinus* differs from *P. prolifica* in having conidiophores reduced to conidiogenous cells, while *P. prolifica* has pale brown erect conidiophores (Kohlmeyer and Kohlmeyer 1979; Hyde et al. 2020a).

### *Ondiniella* E.B.G. Jones, R.G. Johnson & S.T. Moss

The monotypic genus *Ondiniella* was introduced by Jones et al. (1984) to accommodate *O. torquata* as ascospore appendage development was different from that of the type species of *Halosphaeria* (*H. appendiculata*). This was later confirmed by molecular studies (Sakayaroj et al. 2011).

#### Ondiniella

Taxon classificationFungiMicroascalesHalosphaeriaceae

E.B.G. Jones, R.G. Johnson & S.T. Moss, Bot. Mar. 27: 136 (1984)

931A08A2-E6EE-575D-9859-1233E4E800DE

Index Fungorum: IF25830

Fig. 2g

##### Description.

Saprobic on wood: **Sexual morph. *Ascomata*** elongate-cylindrical, immersed ostiolate, membranous, pale to light brown. ***Peridium*** thick, neck long. ***Catenophyses*** present. ***Asci*** 8-spored, clavate, pedunculate, unitunicate, thin-walled, early deliquescing. ***Ascospores*** 1-septate, not constricted at the septum, hyaline, ellipsoidal, with polar and equatorial appendages. **Asexual morph**. Undetermined.

##### Type species.

*Ondiniella torquata* (Kohlm.) E.B.G. Jones, R.G. Johnson & S.T. Moss, Bot. Mar. 27: 136 (1984).

##### Notes.

*Ondiniella* shares some characteristics with *Lautosporopsis*, both with polar and equatorial appendages, but differ in their development. *Ondiniella* is saprobic on intertidal and driftwood, test panels (*Fagus sylvatica*, *Tsuga heterophylla*) in temperate marine environments.

##### Molecular evaluation.

In Fig. 4, *Ondiniella torquata* formed a highly supported clade with *Toriella tubulifera* but their ascospores differ morphologically and at the ultrastructural level (Jones et al. 1984). No molecular data for the type material, and further collections are required to confirm the present assignment. Divergence-time analysis indicates that *Ondiniella* and *Toriella* separated across the Triassic–Neogene interval (~72 MYA, 95% CI: 21.1–248 MYA), supporting their recognition as independent genera within the Halosphaeriaceae (Fig. 5).

#### Ondiniella torquata

Taxon classificationFungiMicroascalesHalosphaeriaceae

(Kohlm.) E.B.G. Jones, R.G. Johnson & S.T. Moss, Bot. Mar. 27: 136 (1984)

485C8352-783F-5BAC-AAA8-5BDF11E6A90D

Index Fungorum: IF107067

Halosphaeria torquata Kohlm., Nova Hedwigia 2: 311 (1960). Basionym.

##### Description.

Saprobic on wood. **Sexual morph. *Ascomata*** 225–336 µm high, 253–363 µm diam., elongate cylindrical or subglobose, immersed, ostiolate, papillate, membranous, pale brown. ***Peridium*** 12–26 µm thick, composed of 4–8 layers of thin-walled cells with large lumina forming a *textura angularis*. ***Necks*** 200–396 µm long, 60–100 µm wide, centric or eccentric. ***Pseudoparenchyma*** breaking up into catenophyses. ***Asci*** 80–150 × 20–33 µm, 8-spored, clavate or subfusiform, pedunculate, unitunicate, thin-walled, without apical apparatus, early deliquescing. ***Ascospores*** 20–331 × 10–16 µm, broadly ellipsoidal, 1-septate, hyaline, slightly constricted at the septum, with apical and equatorial appendages. ***Appendages*** polar subcylindrical, rigid and a collar-like appendage around the central septum. **Asexual morph**. Undetermined. (Based on Kohlmeyer and Kohlmeyer 1979).

##### Notes.

*Ondiniella torquata* has distinctive appendages with a collar-like equatorial appendage, sticky and forming adhesive attachment to substrates (Jones et al. 1984; Jones 1994). Appendages arise as outgrowths of the episporium, and the exosporium is absent. Histochemically the ascospore appendages are composed of protein and carboxylated periodic acid-Schiff positive carbohydrates (Jones et al. 1984).

##### Distribution.

Canada, Chile, Iceland, UK (Scotland) UD (Connecticut, Washington).

### *Ophiodeira* Kohlm. & Volkm.-Kohlm.

The monotypic genus *Ophiodeira* was introduced by Kohlmeyer and Volkmann-Kohlmeyer (1988) for a marine sexual species *Ophiodeira monosemeia*. The genus is characterised by a thin stroma, a curved neck and hyaline, ellipsoidal, 1-septate ascospores with a single polar appendage which unfurls at maturity forming thin threads. The genus was referred to the Halosphaeriaceae by Kohlmeyer and Volkmann-Kohlmeyer (1988) and this was confirmed by sequence data (Jones et al. 2015; Maharachchikumbura et al. 2015). Campbell et al. (2003) argued against the transfer of *Ascosalsum* species to *Ophiodeira*, because the ascomata of the latter are formed under a thin stroma, ascospores are ellipsoidal and smaller than those of *Ascosalsum* species. Based on phylogenetic analysis and morphological characters, Dupont et al. (2009) agree with the separation of the two genera.

#### Ophiodeira

Taxon classificationFungiMicroascalesHalosphaeriaceae

Kohlm. & Volkm.-Kohlm., Canad. J. Bot. 66 (10): 2062 (1988)

98F2C4D9-9A9E-5770-BFFD-89760C96E13A

Index Fungorum: IF25267

##### Description.

Saprobic on submerged woody substrates in marine habitats. **Sexual morph. *Ascomata*** ellipsoidal, immersed under a thin black stroma, ostiolate, light brown, solitary or gregarious, neck elongated, periphysate. ***Peridium*** thin, forming *textura angularis*. ***Catenophyses*** absent. ***Asci*** 8-spored, clavate, pedunculate, thin-walled, unitunicate, deliquescing early, lacking an apical apparatus. ***Ascospores*** hyaline, 1-septate, ellipsoidal, not constricted at the septum, with a single polar appendage, initially hamate, separating from the spore wall and eventually uncoiling to form long, sticky filaments. **Asexual morph**. Undetermined.

##### Type species.

*Ophiodeira monosemeia* Kohlm. & Volkm.-Kohlm., Canad. J. Bot. 66 (10): 2062 (1988).

##### Notes.

The monotypic genus, *Ophiodeira* is characterized by ascospores with a single polar appendage uncoiling to form sticky filaments, as found in the genera: *Halosarpheia*, *Ascosacculus* and *Magnisphaera*. *Ophiodeira* differs from the three previous genera by having ascomata that are immersed under a thin black stroma, deliquescing asci, absence of catenophyses and a single ascospore appendage that is cap-like, attached to the apex and side of the ascospore, at first stiff and homogenous, in water becoming soft and banner-like, eventually transforming into a coil of delicate fibers that uncoil and form long, sticky filaments (Kohlmeyer and Volkmann-Kohlmeyer 1988; Jones et al. 2009).

*Ophiodeira* closely resembles *Tirispora* in that both genera have a single polar appendage that unfurls in water. However, both genera differ in that *Ophiodeira* has ascomata produced under a thin, black stroma, lacks catenophyses, asci deliquesce at maturity and lacks an apical pore in the ascus (Jones et al. 1994).

##### Molecular evaluation.

*Ophiodeira* formed a highly supported monophyletic clade sister to *Oceanitis* species (Campbell et al. 2003; Pang et al. 2003b; Sakayaroj et al. 2011), and this is confirmed in the present study (Fig. 4). Further collections and sequences are required as only one strain (JK 5164A) from the type material, has been sequenced. Divergence-time analysis places the split between *Ophiodeira* and *Oceanitis* near the mid-Cretaceous (~105 MYA, 95% CI: 26.6–414 MYA), underscoring their independent generic status (Fig. 5).

#### Ophiodeira monosemeia

Taxon classificationFungiMicroascalesHalosphaeriaceae

Kohlm. & Volkm.-Kohlm., Canad. J. Bot. 66 (10): 2062 (1988)

DEA76677-FD97-57C8-BACF-50F35B433686

Index Fungorum: IF134493

##### Description.

Saprobic on submerged wood in marine habitats. **Sexual morph. *Ascomata*** 125–270 µm high, 210–320 µm wide, ellipsoidal, immersed under a thin black stroma, ostiolate, with long necks, light brown, solitary or gregarious. ***Necks*** 60–300 µm long, 25–35 µm in diam., more or less laterally inserted, curved, dark brown, ostiolar canal filled with short periphyses, necks piercing through the stroma. ***Peridium*** 15–20 µm thick, composed of 5–7 layers of polygonal flat cells with thick walls, forming a *textura angularis*, merging with the thin-walled pseudoparenchymatous cells. ***Pseudoparenchyma*** composed of thin-walled, large polygonal cells, filling the centrum of young ascomata, finally collapsing without forming catenophyses. ***Asci*** 45–65 × 15–18 µm, 8-spored, clavate, pedunculate, thin-walled, unitunicate, early deliquescing, lacking an apical apparatus, developing successively on an ascogenous tissue at the bottom of the locule, mostly arranged horizontally, parallel to the wood surface. ***Ascospores*** 15.8–20.7 × 5.9–7.7 µm, ellipsoidal, 1-septate, not constricted at the septum, hyaline, with a single appendage. ***Appendages*** 1 µm thick, 7–11 µm long, cap-like, probably originating from an apical pore, attached to the apex and side of the ascospore, at first stiff and homogenous, in water becoming soft and banner-like, eventually transforming into a coil of delicate fibers that uncoil and form long, sticky filaments, remaining attached to the ascospore apices with their bases. **Asexual morph**. Undetermined.

##### Notes.

*Ophiodeira monosemeia* was found from marine habitats on decaying wood of *Avicennia germinans*, *Rhizophora apiculata*, *Rh. mangle*, *Sonneratia griffithii* and *Xylocarpus granatum* (Kohlmeyer and Volkmann-Kohlmeyer 1988; Hyde 1990a, 1990b; Hyde et al. 1990, 1993; Sarma and Vittal 2000). No asci or ascospores were found in examination of the type material, only fragments of wood remain.

##### Distribution.

Virgin Islands, St. Cruiz.

### *Pangia* Abdel-Wahab, M.F. Caeiro & E.B.G. Jones, gen. nov.

A new genus is introduced for *Aniptodera limnetica* which does not belong in *Aniptodera* based on sequencing data.

#### Pangia

Taxon classificationFungiColeopteraHalosphaeriaceae

Dayar., Abdel-Wahab & E.B.G. Jones, g en. nov.

BF3A44EC-9947-5534-A393-46F983531D6A

Index Fungorum: IF903247

##### Etymology.

In honor of marine mycologist Ka-Lai Pang and dominative *ella*.

##### Description.

Saprobic on wood. **Sexual morph. *Ascomata*** superficial to partly immersed, globose to subglobose, ostiolate, hyaline. ***Peridium*** membranous, hyaline, of *textura angularis*. ***Necks*** cylindrical, long, periphysate. ***Asci*** cylindrical to clavate, unitunicate, thin-walled, containing eight ascospores, with apical pore and cytoplasm retracted below the ascus apex, deliquescent. ***Ascospores*** 1-septate, hyaline, ellipsoidal, frequently allantoid, with a thick wall. **Asexual morph**. Undetermined.

##### Type species.

*Pangia limnetica* (Shearer) Abdel-Wahab, M.F. Caeiro & E.B.G. Jones.

##### Notes.

The genus *Aniptodera* has been shown to be repeatedly polyphyletic with *Aniptodera lignatilis* referred to *Aniptosporopsis lignatilis* and *Aniptodera longispora* to *Paraaniptodera longispora*, as well as others proposed in this monograph. *Pangia limnetica* has smaller ascocarps, asci and ascospores than *A. chesapeakensis* with ascospores that are allantoid with rounded tips. Ascospores do not possess polar appendages that uncoil to form long filamentous threads. Sequence data indicates that *A. limnetica* belongs in a new genus and this introduced as *Pangia* here.

##### Molecular evaluation.

In Fig. 4, *Pangia limnetica* formed a clade with *Tirispora unicaudata*, a genus with a single polar appendage. Divergence-time analysis indicates that *Pangia* originated very recently, with a crown age near the present (~0 MYA, 95% CI: 0–12.3 MYA) (Fig. 5).

#### Pangia limnetica

Taxon classificationFungiColeopteraHalosphaeriaceae

(Shearer) Abdel-Wahab, M.F. Caeiro & E.B.G. Jones comb. nov.

DB635DA4-AA3C-5B53-8BE8-054DD10006BF

Index Fungorum: IF903248

Aniptodera limnetica Shearer, Mycologia 81 (1): 140 (1989). Synonym.

##### Description.

Saprobic on wood in freshwater. **Sexual morph. *Ascomata*** superficial to partly immersed, globose to subglobose, ostiolate, hyaline, 139–208 × 129–198 µm; peridium membranous, hyaline, composed of *textura angularis*. ***Necks*** cylindrical, long, periphysate, 59–178 × 40–69 µm. ***Catenophyses*** present. ***Asci*** in a hymenium at base of ascoma, cylindrical to clavate, 50–70 × 12–17 µm, unitunicate, thin-walled, 8-spored, with an apical pore and cytoplasm retracted below the ascus apex, deliquescent. ***Ascospores*** 1-septate, hyaline, ellipsoidal, frequently allantoid, 18–21.9–24 × 8–9.5–11 µm, thick-walled, (1.5–)2–2.8(–3) µm thick. **Asexual morph**. Undetermined.

##### Notes.

*Pangia limnetica* was initially collected on woody twigs in a freshwater stream, a tributary of the Salt Fork of the Vermilion River, Vermilion County, east central Illinois (Shearer 1989) along with other species *Aniptodera fusiformis*, and *A. margarition*.

##### Distribution.

Illinois (USA) Not a well-documented species.

### *Paraaniptodera* K.L. Pang, C.L. Lu, W.T. Ju & E.B.G. Jones

*Paraaniptodera* was introduced for *Aniptodera longispora* as it did not group with the type species of the genus *Aniptodera* (*A. chesapeakensis*).

#### Paraaniptodera

Taxon classificationFungiMicroascalesHalosphaeriaceae

K.L. Pang, C.L. Lu, W.T. Ju & E.B.G. Jones, Bot. Mar. 60 (4): 460 (2017)

ACA6797A-77C3-57E2-8005-0825D21E709C

Index Fungorum: IF818212

##### Description.

Saprobic on wood. **Sexual morph. *Ascomata*** pyriform to subglobose, lying horizontally immersed in the substratum, membranous, light brown to back, ostiolate, papillate, periphysate, peridial wall thick (15–28 µm). ***Catenophyses*** present. ***Asci*** 8-spored, clavate, short pedicel, unitunicate, persistent, thickened apically with a distinct pore. ***Ascospores*** cylindrical with rounded apices, 1-septate, hyaline, lacking appendages. **Asexual morph**. Undetermined.

##### Type species.

*Paraaniptodera longispora* (K.D. Hyde) K.L. Pang, C.L. Lu, W.T. Ju & E.B.G. Jones, Bot. Mar. 60 (4): 460 (2017).

##### Notes.

*Paraaniptodera longispora* was originally described by Hyde (1990c) from dead roots of the mangrove tree *Rhizophora apiculata*, based on cylindrical ascospores with rounded apices and lacking polar appendages and these features distinguish it from other *Aniptodera* species. Morphologically it shares features with *Nais* and *Lignincola*, genera with 1-septate ascospores lacking appendages, but asci in these genera lack apical pores (Kohlmeyer and Kohlmeyer 1979). Sequence data shows that it does not group with the type species of the genus, but forms a highly supported sister clade to two strains of *Cucullosporella mangrovei* (Jones et al. 2017) in the Halosphaeriaceae. Ascospores of *C. mangrovei* have similar measurements (49–64 × 16–24 µm) to *P. longispora* and both occur in mangrove habitats (Alias and Jones 2010). They differ in that *C. mangrovei* has bipolar unfurling appendages that are extruded through a collar-like structure (Alias et al. 2001).

##### Molecular evaluation.

In Fig. 4, *P. longispora* formed a strongly supported sister clade with *Cucullosporella mangrovei*. Divergence-time analysis places this split near the K–Pg boundary (~66 MYA, 95% CI: 18.9–233.6 MYA), supporting the recognition of *Paraaniptodera* as an independent lineage within the Halosphaeriaceae (Fig. 5).

#### Paraaniptodera longispora

Taxon classificationFungiMicroascalesHalosphaeriaceae

(K.D. Hyde) K.L. Pang, C.L. Lu, W.T. Ju & E.B.G. Jones, Bot. Mar. 60 (4): 460 (2017)

445ACFD5-B7E9-5652-9FF9-979DDA57B393

Index Fungorum: IF818215

Aniptodera longispora K.D. Hyde, Bot. Mar. 33 (4): 335–338 (1990). Synonym.

##### Description.

Saprobic on wood. **Sexual morph. *Ascomata*** 270–390 µm long, 180–260 µm wide and 150–205 µm thick, pyriform to subglobose in frontal view, ellipsoidal in end view, lying horizontally immersed in the substrate, membranous, light brown to black above, hyaline below, ostiolate, papillate, solitary or gregarious. ***Peridium*** 15–28 µm thick, outer layer of melanized thick-walled angular cells, inner layer of hyaline thin-walled elongate cells. ***Necks*** periphysate. ***Catenophyses*** present. ***Asci*** 145–201 × 24–31 µm, 8-spored, clavate, short pedicel, unitunicate, persistent, thin-walled, apically thickened with a pore. ***Ascospores*** 39–51 × 9–13.5 µm, cylindrical with rounded apices, 2–3 seriate, 1-septate, slightly constricted at the septum, each cell with a prominent guttule, hyaline, lacking appendages or a sheath. **Asexual morph**. Undermined. (after Hyde 1990c).

##### Distribution.

Australia, Belize, Bermuda, Brunei, Malaysia, Thailand, USA (Florida).

##### Notes.

Saprophytic on dead roots and wood of mangrove trees.

### *Phaeonectriella* R.A. Eaton & E.B.G. Jones

*Phaeonectriella* was introduced by Eaton and Jones (1970) to accommodate *Phaeonectriella lignicola*, originally described from test blocks exposed in water cooling towers and was not assigned to any order or family (Jones 1995; Hyde et al. 1999b). On re-examination it was assigned to the Halosphaeriaceae (Jones 1995). Currently the genus has three accepted species with *P. appendiculata*, recovered from wood in a stream in Queensland, Australia (Hyde et al. 1999b) and *P. alba*, collected on *Phragmites australis*, from the River Nile, Aswan Governorate, Egypt (Abdel-Aziz et al. 2019), both freshwater species.

#### Phaeonectriella

Taxon classificationFungiMicroascalesHalosphaeriaceae

R.A. Eaton & E.B.G. Jones, Nova Hedwigia 19 (3–4): 779 (1971)

A8628B74-8461-5A54-B030-7F2B9B73D92B

Index Fungorum: IF3928

##### Description.

Saprobic on wood. **Sexual morph. *Ascomata*** globose, solitary, immersed in the wood, membranous, hyaline to pale yellow, long periphysate necks. ***Asci*** unitunicate, clavate. 8-spored, lacking paraphyses. ***Ascospores*** 2-septate, brown. **Asexual morph**. Undetermined.

##### Type species.

*Phaeonectriella lignicola* R.A. Eaton & E.B.G. Jones, Nova Hedwigia 19 (3–4): 779 (1971).

##### Notes.

*Phaeonectriella* is like *Aniptodera*, as both genera possess hyaline ascomata with long periphysate necks, asci with an apical pore and catenophyses, however they differ in that ascospores in *Phaeonectriella* are brown and lack polar hamate appendages that form long viscous threads (Jones 1995).

*Phaeonectriella* species differ in their ascospore measurements, presence of polar appendages and ecologically: *P. lignicola* known on wood from brackish water, ascospores 26–30 × 9.5–11 µm, lacking polar appendages; *P. appendiculata*, freshwater on wood, ascospores 32–42 × 10–12 µm with polar appendages; and *P. alba* on *Phragmites australis* stems in freshwater, ascospores 25–33 × 8–13 µm lacking polar appendages (Abdel-Aziz et al. 2019). *Phaeonectriella appendiculata* is the only species with appendaged ascospores. Ascospore appendages were not mentioned in the holotype of *P. lignicola* nor in the Taiwanese specimen (Eaton and Jones 1970; Jones 1995). However, a specimen from Mauritius identified as *P. lignicola* by Hyde et al. (1999b) did produce polar appendages.

##### Molecular evaluation.

*Phaeonectriella alba* grouped consistently with the type species *P. lignicola* in the Halosphaeriaceae with strong support in all analyses, with 100% statistical support in ML/MP and BYPP (Fig. 4). Divergence-time analysis places the crown of *Phaeonectriella* in the Miocene (~9 MYA, 95% CI: 4.9–18.3 MYA), supporting its recognition as a distinct and cohesive genus within the family (Fig. 5). However, *Phaeonectriella appendiculata* needs to be recollected and sequenced to determine if it belongs in the genus.

#### Phaeonectriella lignicola

Taxon classificationFungiMicroascalesHalosphaeriaceae

R.A. Eaton & E.B.G. Jones, Nova Hedwigia 19 (3–4): 779 (1971)

D9E8130F-E245-5F5F-BB65-D5A00B0ED85C

Index Fungorum: IF319613

##### Description.

Saprobic on wood. **Sexual morph. *Ascomata*** 280–575 µm long, 100–150 µm wide, solitary, immersed, globose to subglobose, hyaline to pale brown, membranous. ***Necks*** 100–317 µm long, 46–93 µm wide, periphysate, hyaline, darkening with age, protruding from the wood. ***Paraphyses*** lacking. ***Asci*** 100–417 × 22–35 µm, clavate with an apical pore, 8-spored and unitunicate. ***Ascospores*** 26–30 × 9.5–11 µm,1-septate, asymmetrical, ellipsoidal, hyaline becoming brown at maturity. Ascospores wall smooth with apical appendages. **Asexual morph**. Undetermined.

##### Notes.

This species was originally described from brackish water on exposed beech and Scots pine test blocks in water cooling towers, located in Great Britain. At a later date, new samples were found on twigs from a freshwater stream near Taipei (Taiwan) and from Black River (Mauritius) on submerged wood (Jones 1995; Hyde et al. 1999b; Abdel-Aziz et al. 2019).

### *Praelongicaulis* E.B.G. Jones, Abdel-Wahab & K.L. Pang

A monotypic genus created to accommodate *Halosarpheia kandeliae*, a marine mangrove fungus, which was phylogenetically unrelated to the genus *Halosarpheia* (Sakayaroj et al. 2011; Jones et al. 2015).

#### Praelongicaulis

Taxon classificationFungiMicroascalesHalosphaeriaceae

E.B.G. Jones, Abdel-Wahab & K.L. Pang, Fungal Diversity 73: 54 (2015)

085462EE-B7AD-55C9-A338-3F388A7CC4E6

Index Fungorum: IF812594

##### Description.

Saprobic on intertidal, decaying mangrove wood and bark. **Sexual morph. *Ascomata*** ellipsoidal, dark-colored, membranous to coriaceous, immersed, ostiolate, papillate. ***Necks*** periphysate. ***Peridium*** two layers of cells of *textural angularis*, outer layer of polygonal dark brown, melanized cells with large lumina, inner layer of elongated, polygonal, light brown cells. ***Catenophyses*** present. ***Asci*** thin-walled, unitunicate, 8-spored, clavate, persistent, with a long stalk. ***Ascospores*** hyaline, ellipsoidal, thin-walled, 1-septate, not constricted at the septum. *Appendages* bipolar, unfurling to form a long fine thread in sea water. **Asexual morph**. Undetermined.

##### Type species.

*Praelongicaulis kandeliae* (Abdel-Wahab & E.B.G. Jones) E.B.G. Jones, Abdel-Wahab & K.L. Pang, Fungal Diversity 73: 54 (2015).

##### Notes.

*Praelongicaulis kandeliae* was found to be unrelated to the type species of *Halosarpheia* (*H. fibrosa*) in a phylogenetic review of the Halosphaeriaceae (Sakayaroj et al. 2011), but no formal taxonomic change was made.

##### Molecular evaluation.

Phylogeny of *Praelongicaulis* was poorly resolved in early studies. In our study *Praelongicaulis kandeliae* is in a highly supported clade, sister (but without support) to the *Saagaromyces* clade (Fig. 4). Divergence-time estimates place the *Praelongicaulis*–*Saagaromyces* split in the Jurassic (~168 MYA, 95% CI: 57–469.4 MYA), supporting its recognition as an independent lineage within the Halosphaeriaceae (Fig. 5).

#### Praelongicaulis kandeliae

Taxon classificationFungiMicroascalesHalosphaeriaceae

(Abdel-Wahab & E.B.G. Jones) E.B.G. Jones, Abdel-Wahab & K.L. Pang, Fungal Diversity 73: 54 (2015)

C9E4A8F1-2A7A-5EAD-8968-29F91531F2D1

Index Fungorum: IF812595

Halosarpheia kandeliae Abdel-Wahab & E.B.G. Jones, Mycol. Res. 103 (11): 1500 (1999). Basionym.

##### Description.

Saprobic on intertidal, decaying mangrove wood and bark. **Sexual morph. *Ascomata*** 200–(235)–310 µm high × 120–(157)–180 µm diam., ellipsoidal, black upper side, light brown bottom, membranous, immersed under blackened bark, ostiolate, papillate. ***Peridium*** 25–(33)–42 µm thick, two layered, forming *textura angularis*: outer stratum 10–(12)–15 µm thick, 3–5 layers of polygonal dark brown melanized cells with large lumina; inner stratum 15–(21)–28 µm thick, 5–7 layers of elongated polygonal, light brown cells with narrow lumina. ***Necks*** 110–(130)–137 µm long × 40–(63)–77 µm diam., light brown, periphysate. ***Catenophyses*** present. ***Asci*** 50–(79)–110 × 12–(19)–22 µm, with narrow long stalks, thin-walled, unitunicate, 8-spored, clavate, persistent, 10–(28)–44 µm long, 1–(2)–3 µm diam. ***Ascospores*** 12–(15)–21 × 4–(6)–8 µm, hyaline, ellipsoidal, thin-walled, 1-septate, not constricted at the septum, appendaged. ***Appendages*** bipolar, 5–(10)–13 µm long, 2–(3)–4 µm diam., unfurling into fine thread in water. **Asexual morph**. Undetermined.

##### Notes.

The main characteristic of *P. kandeliae* is its asci with a long stalk (Abdel-Wahab et al. 1999). This species is saprobic on intertidal, decaying mangrove wood and bark.

##### Distribution.

Hong Kong, Taiwan.

### *Panorbis* J. Campb., J.L. Anderson & Shearer

Schmidt (1974a) described *Halosphaeria viscosa* from rotten wood submerged in seawater from Germany, but was transferred to *Halosarpheia* by Shearer and Crane (1980). Later, Hyde et al. (1999b) collected *H. viscosa* from freshwater habitats in the Philippines and South Africa. Campbell et al. (2003) established the genus *Panorbis* and synonymised *Halosphaeria viscosa* as *Panorbis viscosus* based on morphology and phylogeny.

#### Panorbis

Taxon classificationFungiMicroascalesHalosphaeriaceae

J. Campbell, J.L. Anderson & Shearer, Mycologia 95 (3): 544 (2003)

ED0D6317-8604-5B2B-95FD-B868C29B4E94

Index Fungorum: IF28704

##### Description.

Saprobic on woody and herbaceous plant debris in freshwater, brackish and marine habitats. **Sexual morph. *Ascomata*** globose to subglobose ostiolate hyaline at first become black with age. Peridium membranous, thin walled forming *textura angularis*. ***Necks*** long cylindrical periphysate hyaline at the apex and pigmented at the base. ***Hamathecium*** absent or present as catenophyses. ***Asci*** ellipsoid to clavate, thin-walled, persistent to deliquescent, lacking an apical pore and apical apparatus. ***Ascospores*** hyaline, 1-septate, ellipsoid tapered or rounded at the apices and often flattened on one side, with an apical appendage at each end. ***Appendages*** small, hamate at first, less than or equal to one half the ascospore length, unfurling in water to form long, sticky, thread-like structures. **Asexual morph**. Undetermined.

##### Type species.

*Panorbis viscosus* (I. Schmidt) J. Campb., J.L. Anderson & Shearer, Mycologia 95 (3): 544 (2003).

##### Notes.

*Panorbis* is a monotypic genus described from marine habitats in Germany (Schmidt 1974a, 1985). Later it was recorded from freshwater habitats (Hyde et al. 1999b). Originally described as a *Halosphaeria* species, however, molecular phylogenetic studies based on ribosomal genes placed it as a distinct lineage apart from *Halosarpheiasensu stricto* and a new genus was proposed to accommodate it (Campbell et al. 2003).

##### Molecular evaluation.

In Fig. 4, two strains of *P. viscosus* group in a long branch with a number of weakly resolved genera (*Kochiella*, *Ocostaspora* and *Morakotiella*). Divergence-time analysis places this lineage in the Paleogene–Neogene interval (~18 MYA, 95% CI: 5–64.3 MYA), but its position remains unstable (Fig. 5). Further collections are required for isolation and sequencing.

#### Panorbis viscosus

Taxon classificationFungiMicroascalesHalosphaeriaceae

(I. Schmidt) J. Campb., J.L. Anderson & Shearer, Mycologia 95 (3): 544 (2003)

158636A1-EB10-5A24-863D-DF213DC8AA99

Index Fungorum: IF373678

Halosphaeria viscosa I. Schmidt, Natur Naturschutz Mecklenburg 12: 70 (1974). Basionym.
Halosarpheia viscosa (I. Schmidt) Shearer & J.L. Crane, Bot. Mar. 23: 608 (1981). Synonym.

##### Description.

Saprobic on woody and herbaceous plant debris in freshwater, brackish and marine habitats. **Sexual morph. *Ascomata*** 140–230 µm in diam., globose, subglobose or ellipsoidal, immersed, semi-immersed or superficial, black, membranous, ostiolate, periphysate. ***Peridium*** up to 10 µm wide, comprising compressed cells. ***Catenophyses*** present. ***Asci*** 62–75 × 17–20 µm, 8-spored, clavate, pedicellate, lacking an apical pore or thickening, deliquescent. ***Ascospores*** 19–27 × 7.5–9.5 µm, hyaline, ellipsoidal, 1-septate, not constricted at the septum, thin-walled, with bipolar hamate appendages, which unravel in water to form long filamentous threads. **Asexual morph**. Undetermined.

##### Material examined.

Philippines, • Luzon, Laguna, Los Ba5os, Mt. Maquiling, on wood submerged in a freshwater stream, Sept. 1995, K. D. Hyde and T. Umali PHIL33 (HKU(M) 2527); South Africa, • Durban, Palmiet River, on submerged wood, Nov. 1994, K. D. Hyde and T. Steinke SAPR7 (HKU(M) 2219).

##### Notes.

*Panorbis viscosus* differs from species in the *Halosarpheiasensu stricto* clade by having thin-walled peridium forming *textura angularis* in surface view; ellipsoid to clavate, thin-walled, persistent to deliquescent asci; 1-septate, ellipsoidal ascospores tapered or rounded at the apices and often flattened on one side, with an apical appendage at each end; appendages small, hamate at first, less than or equal to one-half the ascospore length, unfurling in water to form long, sticky, thread-like structures (Hyde et al. 1999b; Campbell et al. 2003). *Panorbis viscosus* also resembles *Natantispora retorquens*, but differs in ascospore appendage morphology. *Panorbis viscosus* recorded on woody (intertidal wood, mangrove wood) and herbaceous plant debris in freshwater, brackish and marine habitats (Schmidt 1974a, 1985; Hyde et al. 1999b; Campbell et al. 2003; Calabon et al. 2021).

##### Distribution.

Australia, Brunei, China, Denmark, Egypt, Fiji, Germany, Hong Kong, India, Indonesia, Malaysia, Mauritius, Philippines, Portugal, Seychelles, South Africa, Taiwan, Thailand, USA.

### *Pileomyces* K.L. Pang & J.S. Jheng

A monotypic genus described from trapped bamboo on a rocky shore in Taiwan (Pang and Jheng 2012b).

#### Pileomyces

Taxon classificationFungiMicroascalesHalosphaeriaceae

K.L. Pang & J.S. Jheng, Bot. Stud. (Taipei) 53: 536 (2012)

997E77E9-8C78-58DE-855E-B0D977525305

Index Fungorum: IF519625

##### Description.

Saprobic on trapped bamboo. **Sexual morph. *Ascomata*** dark-colored, pyriform with globose to subglobose venter, immersed, erumpent or exposed, coriaceous, ostiolate, smooth. ***Necks*** short. ***Periphyses*** absent. ***Peridium*** dark-colored, 1-layered, composed of a few layers of elongate cells. ***Catenophyses*** present, irregular in shape. ***Asci*** unitunicate, broadly clavate, apically thickened with retraction of plasmalemma, with an apical pore, short pedunculate, thin-walled, 8-spored, persistent or semi-persistent, developing from inner wall of ascoma base. ***Ascospores*** ellipsoidal with rounded apices, 1-septate, hyaline, smooth, thin-walled. ***Appendage*** initially covering one end of ascospores, gradually becoming detached from the ascospores wall to form an ellipsoidal sheet. **Asexual morph**. Undetermined.

##### Type species.

*Pileomyces formosanus* K.L. Pang & J.S. Jheng, Bot. Stud. (Taipei) 53: 536 (2012).

##### Notes.

*Pileomyces* has typical morphological characteristics of the Halosphaeriaceae, such as pseudoparenchymatous centrum, broadly clavate asci, presence of catenophyses, and ellipsoidal/fusiform, mostly hyaline ascospores with appendages (Kohlmeyer and Kohlmeyer 1979). *Pileomyces* (*P. formosanus*), however, is distinct in the family in having a sheet-like appendage covering one end of the ascospores, which detach when mounted in seawater (Pang and Jheng 2012b).

##### Molecular evaluation.

A combined analysis of the 18S and 28S rDNA suggested a close relationship between *P. formosanus* and *Tirispora unicaudata* (Jones et al. 2017). In this study (Fig. 4), *P. formosanus* constituted a separate branch, with no close relatives. Divergence-time analysis places the *Pileomyces* lineage in the Cretaceous (~90 MYA, 95% CI: 50.6–262.4 MYA), underscoring its long independent history and supporting its recognition as a distinct genus within the Halosphaeriaceae (Fig. 5).

#### Pileomyces formosanus

Taxon classificationFungiMicroascalesHalosphaeriaceae

K.L. Pang & J.S. Jheng, Bot. Stud. (Taipei) 53: 536 (2012)

E73AC00A-35FD-5E68-8165-684AE4D025E4

Index Fungorum: IF563712

Fig. 35

##### Description.

Saprobic on trapped bamboo. **Sexual morph. *Ascomata*** 221–(231)–241 × 182–(234)–285 µm, dark-colored, pyriform with globose to subglobose venter, immersed, erumpent or exposed, coriaceous, ostiolate, smooth. ***Necks*** short. ***Periphyses*** absent. ***Peridium*** 15-(19)-24 µm (n=4), dark-colored, 1-layered, composed of a few layers of elongate cells. ***Catenophyses*** present, irregular in shape. ***Asci*** 98–(111)–128 × 27–(31)–37 µm, unitunicate, broadly clavate, apically thickened with retraction of plasmalemma, with an apical pore, short pedunculate, thin-walled, 8-spored, persistent or semi-persistent, developing from inner wall of ascoma base. ***Ascospores*** 26–(30)–36 × 9–(10)–12 µm, ellipsoidal with rounded apices, 1-septate, hyaline, smooth, thin-walled. ***Appendages*** initially covering one end of the ascospores, gradually detach from ascospore to form an ellipsoidal sheet. **Asexual morph**. Undetermined.

**Figure 35.**
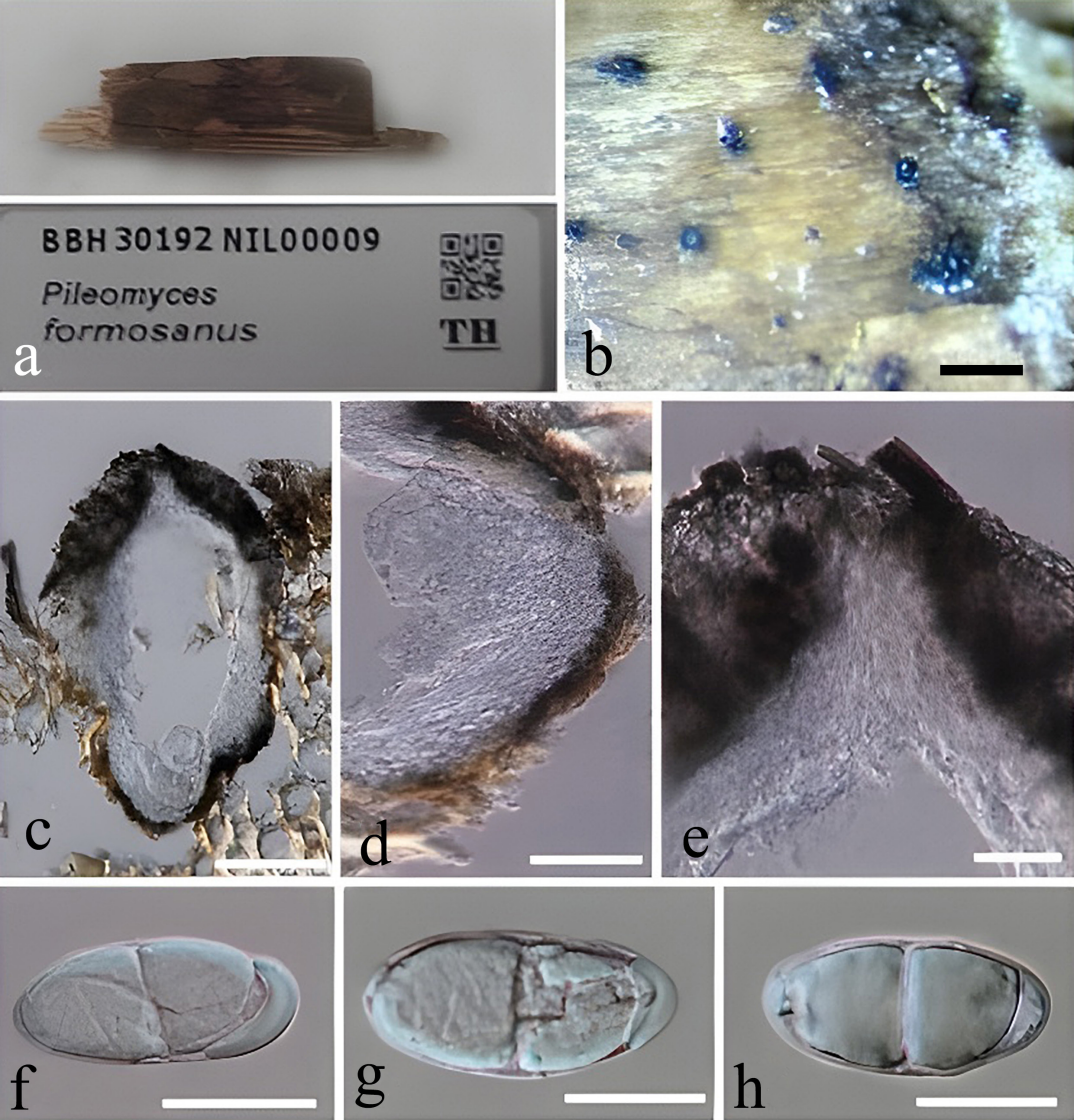
*Pileomyces formosanus* (BBH 30192, holotype)**. a**. herbarium material; **b**. Ascomata on host; **c**. Section through ascomata; **d**. Section through neck region; **e**. Peridium; **f–h**. Ascospores. Scale bars: 50 µm (**c**); 20 µm (**d**); 10 µm (**e**); 5 µm (**f–h**).

##### Notes.

*Pileomyces formosanus* was saprobic on trapped bamboo.

##### Distribution.

Taiwan.

### *Pseudolignincola* Chatmala & E.B.G. Jones

Molecular phylogeny introduced the genus from Thai mangroves with *P. siamensis* as the type species with a humicola-like asexual morph (*Humicola siamensis*).

#### Pseudolignincola

Taxon classificationFungiMicroascalesHalosphaeriaceae

Chatmala & E.B.G. Jones, Nova Hedwigia 83 (1–2): 225 (2006)

2E380E12-DE93-559B-B750-1CE98D7015D1

Index Fungorum: IF29053

##### Description.

Saprobic on wood. **Sexual morph. *Ascomata*** globose, dark brown, coriaceous, long periphysate neck, solitary, immersed in the wood. ***Catenophyses*** present. ***Asci*** 8-spored, unitunicate, clavate to slightly cylindrical, long pedicellate, apex truncate with a refractive apical thickening, apical pore and plasmalemma retracting at the apex. ***Ascospores*** cylindrical, 1–4-septate, hyaline, smooth-walled, lacking a sheath or appendage. **Asexual morph**. Hyphomycetous, mycelium immersed in the substratum, hyphae brown, forming sporodochium-like clusters, conidia holoblastic, unicellular, pyriform to hemispherical, dark brown, borne on a short conidiogenous cell and described as *Humicola siamensis*.

##### Type species.

*Pseudolignincola siamensis* Chatmala & E.B.G. Jones, Nova Hedwigia 83 (1–2): 226 (2006).

##### Notes.

*Pseudolignincola* is similar to *Pseudohalonectria*, a freshwater Ascomycota, but phylogenetically they are distantly related (Minoura and Muroi 1978). The genus grouped with *Magnisphaera spartinae* in a weakly supported clade in the Halosphaeriaceae, but shares no morphological features (Jones et al. 2006).

##### Molecular evaluation.

*Pseudolignincola siamensis* and *Humicola* sp. formed a clade with *Safagamyces marinus* with weak support (Fig. 4). Divergence-time analysis indicates that this lineage originated in the Early Cretaceous (~125 MYA, 95% CI: 28.9–501.5 MYA). Further collections of both species are required to strengthen their phylogenetic resolution (Fig. 5).

#### Pseudolignincola siamensis

Taxon classificationFungiMicroascalesHalosphaeriaceae

Chatmala & E.B.G. Jones, Nova Hedwigia 83 (1–2): 226 (2006)

8972E067-793D-503F-99D2-55479F44E832

Index Fungorum: IF334781

Humicola siamensis Chatmala & E.B.G. Jones, Nova Hedwigia 83 (1–2): 226 (2006). Synonym.

##### Description.

Saprobic on *Nypa* palm. **Sexual morph. *Ascomata*** 410–580 µm high, 445–620 µm diam., globose, dark brown, coriaceous, solitary, with a long, periphysate neck 710–1485 µm long, 34–138 µm wide, immersed in the substrate. ***Peridium*** 45–50 µm thick. Centrum breaking up into catenophyses. ***Asci*** 65–90 × 10–15 µm, 8-spored, clavate to slightly cylindrical, unitunicate, thin-walled, apex truncate, with a refractive apical thickening, a pore and the plasmalemma retracting from the ascus wall. ***Ascospores*** 15–32 × 3–6 µm, 1–4 septate, hyaline, smooth-walled, lacking a sheath or appendages. **Asexual morph. *Mycelium*** hyphomycetous, immersed in the substratum, hyphae brown, 2–3 µm wide, forming sporodochium-like clusters. ***Conidia*** holoblastic, unicellular 14–27 × 10–19 µm, pyriform to hemispherical, dark brown, borne on a short conidiogenous cell (Adapted from Jones et al. 2006).

##### Material examined.

Thailand, • Andaman Sea, Had Chao Mai National Park, coll. I. Chatmala on palm *Nypa fruticans*, July 2000 Herb BIOTEC IT41 (holotype).

##### Notes.

*Pseudolignincola siamensis* resembles *Lignincola nypae* in ascus and hyaline ascospore shape, but differs in that the former has 1–4 septate ascospores. The asexual morph was produced from a germinating single ascospore and sporulates readily in culture. Both sexual and asexual morphs occurred on natural substrata. It is saprobic on mangrove wood.

### *Qarounispora* Nourel-Din, Abdel-Aziz & Abdel-Wahab

*Qarounispora* is a monotypic marine genus that was recently established by Nourel-Din et al. (2022) to accommodate *Q. grandiappendiculata* that was described from wood submerged in Qaroun Lake, El-Faiyum governorate, Egypt.

#### Qarounispora

Taxon classificationFungiMicroascalesHalosphaeriaceae

Nourel-Din, Abdel-Aziz & Abdel-Wahab, Phytotaxa 530 (1): 91 (2022)

72D4E665-0E73-5102-AD76-E391E3771C4F

Index Fungorum: IF841141

##### Description.

Saprobic on submerged marine substrates. **Sexual morph. *Ascomata*** perithecial, ostiolate, papillate, partly immersed or superficial, globose to subglobose, yellow to orange brown in color, membranous. ***Necks*** cylindrical to conical, hyaline to yellow, periphysate. ***Peridium*** membranous, one-layered, forming *textura angularis*. ***Catenophyses*** present, developing from the pseudoparenchyma of the centrum. ***Asci*** unitunicate, thin-walled, without an apical apparatus, developing at the base of the ascomatal venter, 8-spored, semi-persistent, clavate or broadly ellipsoidal. ***Ascospores*** hyaline to yellow-orange in color, 1-septate, thick-walled, distoseptate, ellipsoidal to broadly ellipsoidal, with one polar appendage. **Asexual morph**. Undetermined.

##### Type species.

*Qarounispora grandiappendiculata* Nourel-Din, Abdel-Aziz & Abdel-Wahab, Phytotaxa 530 (1): 91 (2022).

##### Notes.

*Qarounispora* is characterized by its yellow to orange-brown ascomata, thin-walled, semipersistent asci, hyaline to yellow-orange, thick-walled, distoseptate ascospores with one polar appendage that is amorphous in structure, large in size and irregular in shape.

##### Molecular evaluation.

Multigene analyses based on 28S, 18S and ITS rDNA placed the new fungus as a distinct branch within the Halosphaeriaceae as a distinct branch from morphologically related fungal taxa with one polar appendage to the ascospores: *Moana*, *Oceanitis*, *Okeanomyces*, *Ophiodeira* and *Tirispora* (Nourel-Din et al. 2022). *Qarounispora grandiappendiculata* formed a monophyletic clade with *Jinshana tangtangiae* (Fig. 4) and they share some morphological details in common, especially the nature of their appendages. Divergence-time analysis places their split in the late Miocene to Pliocene (~4 MYA, 95% CI: 1.3–10.5 MYA), supporting their recognition as closely related but distinct genera (Fig. 5).

#### Qarounispora grandiappendiculata

Taxon classificationFungiMicroascalesHalosphaeriaceae

Nourel-Din, Abdel-Aziz & Abdel-Wahab, Phytotaxa 530 (1): 91 (2022)

327C8D61-59EB-529F-811F-1D3B52451207

Index Fungorum: IF841142

Fig. 36

##### Description.

Saprobic on decaying submerged wood. **Sexual morph. *Ascomata*** 125–200 μm in diam., perithecial, ostiolate, papillate, superficial or partly immersed, globose to subglobose, yellow to orange-brown in color, membranous. ***Necks*** 50–90 μm long, 45–60 μm diam., cylindrical to conical, hyaline to yellow-orange, periphysate, ascospores ooze from the ostiolar canal forming spore mass that is bright yellow-orange in color. ***Peridium*** 12.3–22.8 μm thick, membranous, one-layered, yellow to orange-brown in color, forming *textura angularis*. ***Catenophyses*** present. ***Asci*** 65–115 × 30–45 μm, unitunicate, thin-walled, without an apical apparatus, 8-spored, developing at the base of the ascomatal venter, semi-persistent, clavate or broadly ellipsoidal. ***Ascospores*** 18–40 × 13–18 μm, hyaline to yellow-orange in color, thick-walled, distoseptate, 1- septate, not constricted at the septum, ellipsoidal to broadly ellipsoidal. ***Appendages*** present, one polar appendage coiled inside a globose sheath connected with the ascospore by a hyaline rib and the appendage swells in water to form irregular amorphous large structure 20.3–59 × 14–24.2 μm. **Asexual morph**. Unknown.

**Figure 36.**
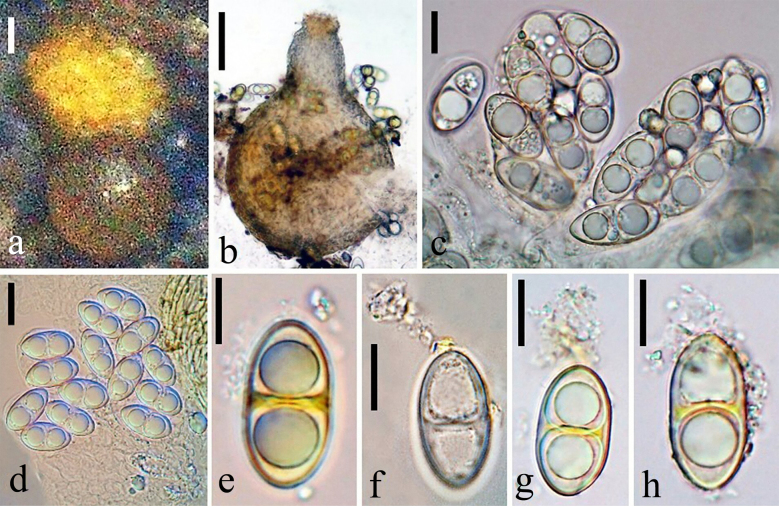
*Qarounispora grandiappendiculata* (SUMCC H-17009, holotype). **a**. Ascomata on natural wood; **b**. Squash of ascoma and asci mounted in seawater; **c, d**. Variously shaped asci; **e–h**. Variously shaped ascospores with amorphous polar appendage. Scale bars: 100 μm (**a, b**); 20 μm (**c–h**).

##### Material examined.

Egypt, • El-Faiyum governorate: Qaroun Lake, on decaying submerged wood, 29°29'00"N, 30°49'09"E, 20 Nov. 2018, A. A. H. Nourel-Din (SUMCC H-17009, holotype).

##### Notes.

*Qarounispora grandiappendiculata* has 1-septate, thick-walled, distoseptate, hyaline to yellow-orange, broadly ellipsoidal ascospores with amorphous, large and irregular single polar appendage. *Halosarpheia japonica* also has one polar appendage that consists of amorphous material enclosed in cellular sheath that dissolves in water and the appendage swell to form huge tree-like appendage that is similar in its nature and shape to the appendages of *Q. grandiappendiculata*. However, *H. japonica* differs from *Q. grandiappendiculata* by having brown to black, large ascomata with thick peridium and with unicellular ascospores. Molecular phylogenetic analyses of the ribosomal genes placed *H. japonica* in *Halosarpheia* (Abdel-Wahab and Nagahama 2012).

*Qarounispora grandiappendiculata* is a saprobic fungus growing on decaying submerged wood in a saline lake (Nourel-Din et al. 2022).

### *Remispora* Linder

*Remispora* was introduced by Barghoorn and Linder (1944) to accommodate *Remispora maritima*. Kohlmeyer (1972) referred the genus to *Halosphaeria* based on his interpretation of ascospore appendage morphology, but the genus was re-established by Johnson et al. (1984). Phylogenetic data indicated that *Halosphaeria* was polyphyletic, and many species transferred to other genera: *Kochiella*, *Okeanomyces*, *Tubakiella*, *Haiyanga* and *Nohea* (Sakayaroj et al. 2011; Jones et al. 2019). In this study, *Remispora* was inferred to be polyphyletic, and *Remisporiopsis* is introduced to accommodate *R. quadri-remis*, *R. stellata*, *R. spitsbergenensis* and *R. submersa*. Currently, there are three accepted species in *Remispora* (Table 1).

#### Remispora

Taxon classificationFungiMicroascalesHalosphaeriaceae

Linder, Farlowia 1 (3): 409 (1944)

26A2AF72-80C5-53AE-BAEB-0E816CD816DF

Index Fungorum: IF4674

##### Description.

Saprobic on different woody substrates. **Sexual morph. *Ascomata*** are solitary or gregarious, globose to subglobose, immersed or superficial, ostiolate, papillate, eperiphysate, membranous, hyaline to cream colored, upper part often darker. ***Peridium*** composed of 6–9 layers of cells which are thick-walled, hyaline, elongated and with large lumina. ***Necks*** are subcylindrical, with or without periphyses. ***Pseudoparenchyma*** with catenophyses present, cells thin-walled, large, ellipsoidal or polygonal. ***Asci*** 8-spored, clavate to broadly fusoid, apiculate, pedunculate, unitunicate, thin-walled, deliquescing early and developing at the base of the ascocarp. ***Ascospores*** ellipsoidal to ovoid, 1-septate, not constricted at the septum, hyaline and appendaged. Polar appendages are pleomorphic (Johnson et al. 1984). **Asexual morph**. Undetermined.

##### Type species.

*Remispora maritima* Linder, Farlowia 1 (3): 410 (1944).

##### Notes.

*Remispora* is distinguished from *Halosphaeria* by the following characters: perithecia usually hyaline, ascocarp wall composed of 6–9 layers of elongate, hyaline, cells; catenophyses present; ascospores have pleomorphic polar appendages; appendages formed by fragmentation or dissolution of an exosporium which totally surrounds the spore, and appendages composed of two components, the amorphous component dissolving in water at maturity (Johnson et al. 1984). Ascospore appendage morphology differs between the *Remispora* species: 1. Appendages wing-like (*R. maritima*, *R. pilleata*); 2. Appendages crown-like with radiating appendages (*R. spitsbergenensis*, *R. quadri-remis*, *R. stellata*), suggesting the latter might belong to a different genus and supported by sequence data (Sakayaroj et al. 2011; Gonçalves et al. 2021). A new genus, *Remisporiopsis*, is established for these species in this monograph.

Gonçalves et al. (2021) introduced *Remispora submersa* from submerged wood baits of *Pinus pinaster* at Ria de Aveiro, Portugal with first report of an asexual stage for the genus *Remispora*. Characteristic features include conidiophores erect, arising directly from the vegetative hyphae, hyaline, smooth-walled, aseptate, 23.7–34.1 × 1.5–2.1 mm and with aseptate conidia hyaline, smooth, cylindrical to narrowly ellipsoid, 3.3–4.3 × 1.2–1.8 μm. Sadly, details of the conidiogenous cells and conidial development are not given or any comment on the morphological features of the species, especially as this is the first report of an asexual stage in the genus. *Remispora submersa* is phylogenetically closely related to *R. spitsbergenensis* (CY5279), *R. quadri-remis* (CBS 334.62), and *R. stellata* (CBS 258.60) (Gonçalves et al. 2021).

##### Molecular evaluation.

*Remispora* was polyphyletic (Fig. 4) and species not grouping with type species (*R. maritima*) are assigned to other genera. *Remispora maritima*, *R. pilleata* (with molecular data) and *R. minuta* (no sequences) are retained in *Remispora*. Species in this genus have a single polar appendage. *Remispora submersa*, *R. spitsbergenensis*, *R. stellata* and *R. quadri-remis* are referred to the new genus *Remisporiopsis* based on molecular data, distantly placed from *Remispora* (Fig. 4) and morphologically ascospore polar appendages composed of a crown of 4–6 appendages. Divergence-time analysis further supports this separation: the *Remisporiopsis* lineage originated in the Cretaceous (~84 MYA, 95% CI: 28.9–246.7 MYA), while *Remisporasensu stricto* dates to the Oligocene (~30 MYA, 95% CI: 19.7–44.7 MYA), confirming their independent evolutionary trajectories (Fig. 5).

#### Remispora maritima

Taxon classificationFungiMicroascalesHalosphaeriaceae

Linder, Farlowia 1 (3): 410 (1944)

C7F9B94F-4ABC-59CF-92B5-CAC462676FAF

Index Fungorum: IF290432

Halosphaeria maritima (Linder) Kohlm., Canad. J. Bot. 50 (9): 1956 (1972). Synonymy.
Remispora lobata Höhnk, Veröff. Inst. Meeresf. Bremerhaven 3: 206 (1955).

##### Description.

Saprobic on wood. **Sexual morph. *Ascomata*** 180–570(–670) µm in diam., globose, subglobose, or ovoid, immersed or superficial, ostiolate, papillate, coriaceous, almost hyaline, cream-colored or smoke-gray, sometimes the upper part darker than the lower, solitary or gregarious. ***Peridium*** is 8–32 µm thick, composed of six to nine layers of thick-walled, more or less elongated cells with large lumina, forming a *textura angularis*, merging into the pseudoparenchyma of the venter. ***Necks*** 100–390(–600) × 20–60 µm, subcylindrical or truncate-conical, sometimes forked or two on one ascocarp, centric or eccentric. ***Pseudoparenchyma*** of thin-walled, large, ellipsoidal or polygonal cells filling the venter of young ascocarps; eventually breaking up into catenophyses, 7–9.5 µm in diameter. ***Asci*** 72–148 × 21.5–39 µm, 8-spored, clavate or broadly fusoid, somewhat apiculate, pedunculate, unitunicate, thin-walled, aphysoclastic, without apical apparatuses, early deliquescing, developing at the base of the ascocarp venter. ***Ascospores*** 18–30(–31.5) × 8–13 µm (excluding appendages), ellipsoidal, ovoid or broadly ellipsoidal, 1-septate, not constricted at the septum, hyaline, appendaged; at first surrounded by a subgelatinous, exosporic sheath that unfolds, remaining attached at both ends of the ascospore; appendages pleomorphic, most apices attenuate and irregularly stretched out (based on Kohlmeyer and Kohlmeyer 1979). **Asexual morph**. Undetermined.

##### Notes.

All species are saprobic on different woody substrates. *Remispora maritima* and *R. pilleata* can be regarded as cold water species, while *R minuta* is a tropical/subtropical species (Pugh and Jones 1986; Pang et al. 2008c; Hagestad et al. 2020).

### *Remisporiopsis* K.L. Pang, E.B.G. Jones, E. Azevedo & M.F. Caeiro, gen. nov.

Sakayaroj et al. (2011) sequenced *Remispora* species which indicated the genus was polyphyletic, while Pang (2012) noted 0.7–7.4% p-distance in the 28S rDNA gene, between *Remispora* species ‘implying that they represented more than one genus’. Gonçalves et al. (2021) introduced *Remispora submersa*, a species from hyphae, lacking any morphological details. This study confirms two phylogenetic groups in *Remispora*: 1. *R. maritima* (type species) and *R. pilleata* and 2. *R. macrocephala*, *R. stellata*, *R. spitsbergenensis*, *R. submersa* and *R. quadri-remis*. The second group is transferred to this new genus *Remisporiopsis*. A new combination, *Remisporiopsis macrocephala*, is made based on their close phylogeny with *Remisporiopsis*.

#### Remisporiopsis

Taxon classificationFungiMicroascalesHalosphaeriaceae

K.L. Pang, E.B.G. Jones, E. Azevedo & M.F. Caeiro gen. nov.

1F8D49A5-A075-51FE-9E40-9739E8830214

Index Fungorum: IF903254

##### Etymology.

From *remi* for *Remispora* and *sporiopsis*.

##### Description.

Saprobic on wood **Sexual morph. *Ascomata*** subglobose, globose, ellipsoidal, immersed or superficial, ostiolate, papillate, coriaceous, creamed colored, solitary or gregarious. ***Peridium*** thick. ***Necks*** variable in length. ***Catenophyses*** present. ***Asci*** 8-spored, clavate, pedunculate, unitunicate, thin-walled, lacking an apical pore, deliquescing early. ***Ascospores*** ellipsoidal, hyaline, 1-septate, not constricted at the septum, appendaged. ***Appendages*** apical, formed by fragmentation of exosporium, forming a crown 4–6 appendages. **Asexual morph**. Undetermined.

##### Type species.

*Remisporiopsis stellatus* (Kohlm.) K.L. Pang, E.B.G. Jones, E. Azevedo & M.F. Caeiro.

##### Notes.

We designate *Remispora stellata* as the type species of this new genus and isolate 3129J (28S rDNA–KM272364, ITS rDNA–KM272365) as the neotype from Rämä et al. (2014), collected on driftwood from Norway (Arctic Ocean), as it best fits the description of *Remispora stellata*.

*Remispora* and *Remisporiopsis* can be distinguished by their appendage morphology: *Remispora* species have a single sheath-like appendage while those of *Remisporiopsis* form a crown of 4–6 apical appendages. Johnson et al. (1984) showed that in a TEM study, these appendages were formed by fragmentation of an exosporial sheath which comprised a fibrous component embedded in an amorphous matrix. When fixed with permanganate, the fibrillar component of *R. stellatus* arises through pores in the episporium (Manimohan et al. 1993). The morphology of *Remisporiopsis submersa* was unknown as sequences were derived from mycelium (Gonçalves et al. 2021). In this monograph we provide a photo plate from material collected in Portugal by E. Azevedo.

Species of this genus are saprobic on intertidal and driftwood, test panels, mangrove wood, seeds of *Cocos nucifera*, calcareous linings of empty shipworm tubes.

##### Molecular evaluation.

The three *Remisporiopsis* species (*R. quadri-remis*, *R. stellatus*, *R. submersa*) formed a well-supported monophyletic clade with *Cirrenalia macrocephala*, sistering with *Ascoglobospora marina* and *Aniptosporopsis lignatilis* (Fig. 4). *Cirrenalia macrocephala* is transferred to *Remisporiopsis* in this study as an asexual species. Divergence-time analysis places the crown of *Remisporiopsis* in the late Eocene (~36 MYA, 95% CI: 9.2–144.3 MYA), supporting its recognition as a distinct genus within the Halosphaeriaceae (Fig. 5).

#### Remisporiopsis stellatus

Taxon classificationFungiMicroascalesHalosphaeriaceae

(Kohlm.) K.L. Pang, E.B.G. Jones, E. Azevedo & M.F. Caeiro comb. nov.

E94D2A32-8C00-57BD-B012-A60ADD2F2F62

Index Fungorum: IF903255

Remispora stellata Kohlm., Nova Hedwigia 2: 334 (1960). Synonyms.
Halosphaeria stellata (Kohlm.) Kohlm., Canad. J. Bot. 50 (9): 1957 (1972).

##### Description.

Saprobic on wood **Sexual morph. *Ascomata*** 172–471 µm high, 226–471 µm diam., subglobose or ovoid, immersed or superficial, ostiolate, papillate, coriaceous, creamed colored, yellowish brown, solitary or gregarious. ***Peridium*** 12–25 µm thick, six layers of roundish or ellipsoidal, thick-walled cells, forming a *textura angularis*. ***Necks*** variable up to 622 µm long. ***Catenophyses*** present. ***Asci*** 72–80 × 19–28 µm, 8-spored, clavate, pedunculate, unitunicate, thin-walled, lacking an apical pore, deliquescing early. ***Ascospores*** 24–30 × 8.5–12.5 µm, ellipsoidal, hyaline, 1-septate, not constricted at the septum, appendaged. ***Appendages*** 13–24.5 µm long, apical, formed by fragmentation of exosporium which is striate in a gelatinous matrix, forming a crown 4–6 appendages. **Asexual morph**. Undetermined.

##### Type sequences.

From isolate 3129J (28S – KM272364, ITS – KM272365).

##### Notes.

*Remisporiopsis stellatus* is found generally in temperate waters on a variety of timber species with Schmidt (1974a) and Koch and Jones (1983) reporting many collections in the Baltic Sea.

##### Substrates.

intertidal and driftwood, test panels *Abies* sp., *Pinus sylvestris*, submerged cones of *Pseudotsuga* sp.

##### Distribution.

Canada, Denmark, Germany, Sweden, UK (England), USA

#### Remisporiopsis macrocephala

Taxon classificationFungiMicroascalesHalosphaeriaceae

(Meyers & R.T. Moore) K.L. Pang. E.B.G. Jones & M.F. Caeiro comb. nov.

5A719F1A-F87F-5BE3-BAF3-1B5D433A55F4

Index Fungorum: IF903849

Helicoma macrocephalum Kohlm., Ber. Deutsch. Bot. Ges. 71 (2): 99 (1958). Basionym.
Cirrenalia macrocephala (Kohlm.) Meyers and R.T. Moore, Amer. J. Bot. 47 (5): 347 (1960). Synonym.

##### Description.

Saprobic on plant debris in marine habitats. **Sexual morph**. Undetermined. **Asexual morph. *Colonies*** effuse, thin, black, granular. ***Hyphae*** 1.5–2 µm diam., septate, ramose, hyaline to dark-brown. ***Conidiophores*** 3.5–25 µm long, 2–5 µm in diam., cylindrical, occasionally apically somewhat inflated, zero- to three-septate, simple, straight or curved, hyaline to yellowish. ***Conidiogenous cells*** monoblastic, integrated, terminal, determinate. ***Conidia*** acrogenous, solitary, helicoid, rarely straight, 0.25 to 1 time contorted, two- to seven-septate, strongly constricted at the septa, multiguttulate, reddish fuscous; cells increasing in diameter and pigmentation from base to apex, distinctly dissimilar; spirals 12–31.5(–35) µm high, 12–23.5 µm diam.; terminal cell 5.5–13.5 µm high, 6.5–14(–17) µm diam., subglobose, basally flattened, reddish fuscous, largest and darkest of all cells; basal cells 1.5–6(–7) µm high, 2.5–7 diam., semiglobose or obtusely conical, hyaline; central cells subglobose, cylindrical or doliform, brownish.

##### Material examined.

Italy, • on panels submerged off shore, J. Kohlmeyer (IMS, J. K. 5188).

##### Notes.

*Remisporiopsis macrocephala* (= *Cirrenalia macrocephala*) is cosmopolitan in its distribution with more records from temperate waters. It was recorded from various substrata including decaying intertidal wood of *Avicennia marina*, *Rhizophora mangle*, *R. mucronata*, driftwood and decaying leaves.

##### Distribution.

Australia, Canada, Denmark, France, Germany, Greece, Iceland, Ivory Coast, Italy, Malaysia, South Africa, New Zealand, UK, USA.

#### Remisporiopsis quadri-remis

Taxon classificationFungiMicroascalesHalosphaeriaceae

(Höhnk) K.L. Pang, E.B.G. Jones, E. A zevedo and M.F. Caeiro comb. nov.

B17A087E-C593-51D8-AAC3-F730BCA7F3B0

Index Fungorum: IF903292

Fig. 2b

Halosphaeria quadri-remis (Höhnk) Kohlm., Canad. J. Bot. 50 (9): 1957 (1972). Synonym.
Palomyces quadri-remis Höhnk, Veröff. Inst. Meeresf. Bremerhaven 3: 213 (1955).
Arenariomyces quadri-remis (Höhnk) Meyers, Mycologia 49: 505 (1957).
Remispora quadri-remis (Höhnk) Kohlm., Nova Hedwigia 2: 332 (1960).

##### Type sequences.

From the isolate CBS 334.62.

#### Remisporiopsis spitsbergenensis

Taxon classificationFungiMicroascalesHalosphaeriaceae

(K.L. Pang & Vrijmoed) K.L. Pang & Vrijmoed comb. nov.

E80BA077-E629-5B25-B01D-31BD75CE5152

Index Fungorum: IF903291

Remispora spitsbergenensis K.L. Pang & Vrijmoed, Mycologia 101 (4): 533 (2009). Synonym.

#### Remisporiopsis submersa

Taxon classificationFungiMicroascalesHalosphaeriaceae

(M. Gonçalves, A. Abreu & A. Alves) K.L. Pang, E.B.G. Jones, E. Azevedo & M.F. Caeiro comb. nov.

B33BB71D-1F5C-52C1-8920-E0E80017EB47

Index Fungorum: IF903289

Fig. 37

Remispora submersa M. Gonç., A. Abreu & A. Alves, Mycologia 113: 4 (2021). Synonym.

##### Notes.

**Figure 37.**
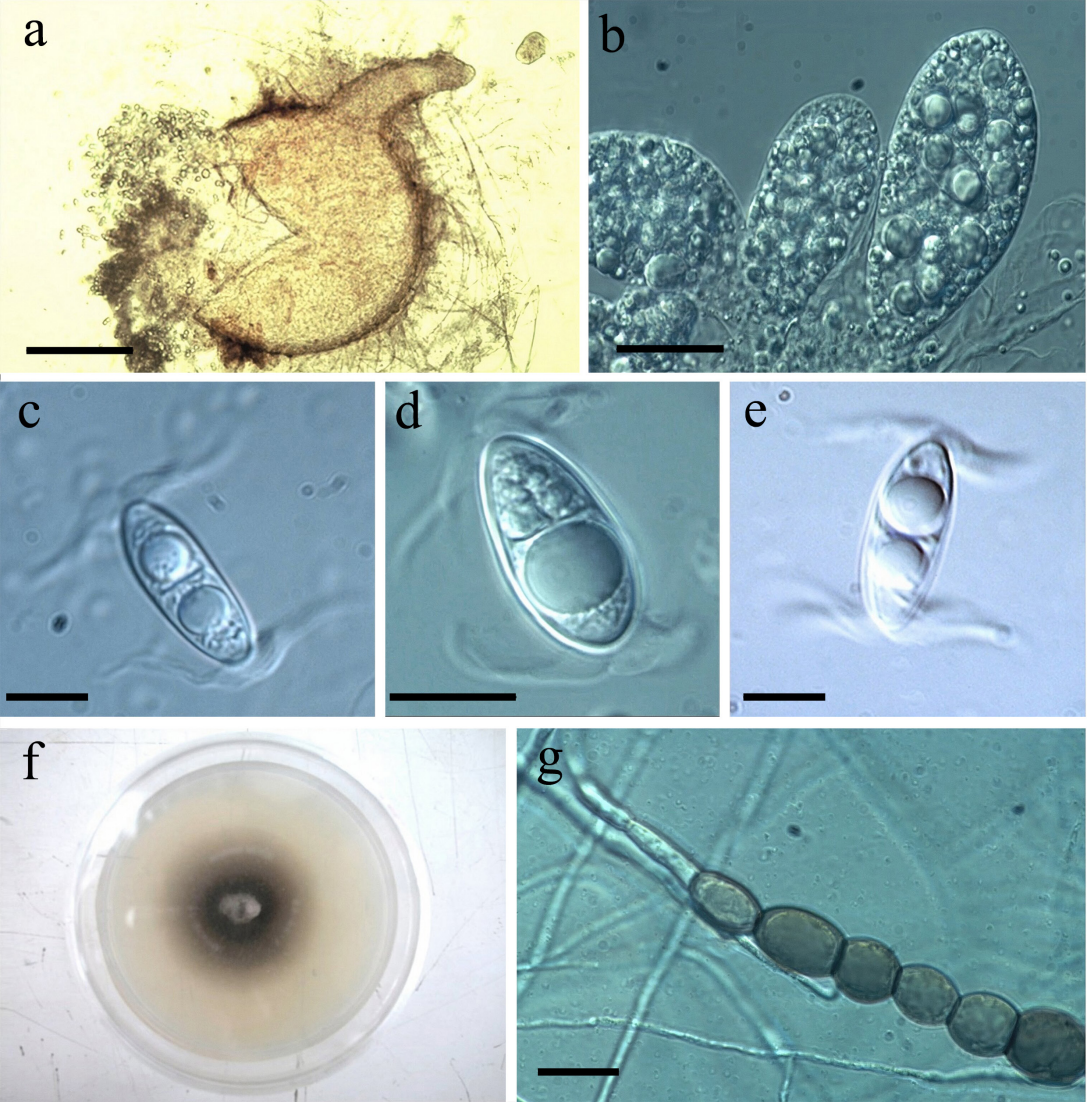
*Remisporiopsis submersa* (FCUL 041207SP3). **a**. Light brown ascomata; **b**. Immature asci; **c–e**. Hyaline ellipsoidal ascospores with four bipolar appendages; **f**. Colony after 15 days at room temperature, on corn meal agar (obverse); **g**. Chain of globose chlamydospores with thick walls, on cornmeal agar. Scale bars: 100 µm (**a**); 50 µm (**b**); 10 µm (**c, e, g**); 20 µm (**d**).

Gonçalves et al. (2021) introduced *Remisporiopsis submersa* based on a sequence derived from fungal mycelium, with an asexual morph with hyaline, aseptate conidia. On request the culture was re-sequenced, grouping in the *Sarocladium* clade and the species *S. kiliense* with high statistical support (Calabon et al. 2023). In this study, a collection of a *Remispora* species was sequenced which matched that of *Remispora submersa*. Herbarium material of this isolate enabled illustration of the sexual morph of *Remisporiopsis submersa* for the first time (Fig. 37). Ascospores of *Re. submersa* has a crown of polar appendages similar to that of *Re. quadri-remis* and producing chlamydospores in culture.

### *Saagaromyces* K.L. Pang & E.B.G. Jones

The genus *Saagaromyces* was introduced by Pang et al. (2003a) for taxa that did not group with *Halosarpheia* or *Nais* (Patil and Borse 1982; Kohlmeyer 1984; Crane and Shearer 1986). Pang et al. (2003b) in a phylogenetic study of selected taxa in the Halosphaeriaceae, showed that *Halosarpheia abonnis* and *H. ratnagiriensis* did not group with type species of *Halosarpheia* (*H. fibrosa*) and introduced the genus *Saagaromyces*. Similarly, *Naïs glitra* did not group in *Naïs* (*N. inornata*) but grouped with *Saagaromyces* species in a well-supported clade (Pang et al. 2003a; Sakayaroj et al. 2011).

#### Saagaromyces

Taxon classificationFungiMicroascalesHalosphaeriaceae

K.L. Pang & E.B.G. Jones, Mycol. Progr. 2 (1): 35 (2003)

EA1C3D79-DFE8-5905-84F8-862C35A8121F

Index Fungorum: IF28748

##### Description.

Saprobic on wood. **Sexual morph. *Ascomata*** globose, subglobose to ellipsoidal, immersed, ostiolate, papillate, membranous to coriaceous, hyaline, cream-colored, brown to black. ***Necks*** periphysate. ***Catenophyses*** present. ***Asci*** 8-spored, cylindrical-clavate, pedunculate, unitunicate, thin-walled, persistent. ***Ascospores*** oval, hyaline, heavily guttulated, with or without unfurling appendages. **Asexual morph**. Undetermined.

##### Type species.

*Saagaromyces ratnagiriensis* (S.D. Patil & Borse) K.L. Pang & E.B.G. Jones, Mycol. Progr. 2 (1): 35 (2003).

##### Notes.

Several new collections have been made of *Saagaromyces* species and details are presented herein. Currently, three *Saagaromyces* species are accepted (Jones et al. 2019). They are saprobic on decaying mangrove wood.

This genus is easily distinguishable from the other genera in Halosphaeriaceae in having 1-septate, broadly ellipsoidal ascospores with a heavy guttulation pattern and large hamate polar appendages that are equal to or longer than half of the ascospore length and width (Pang et al. 2003a).

##### Molecular evaluation.

All species of *Saagaromyces* formed a strong monophyletic group, sistering, without support, with *Praelongicaulis* and *Alisea* (Fig. 4). Divergence-time analysis places the crown of *Saagaromyces* in the Oligocene (~29 MYA, 95% CI: 7.4–117.3 MYA), indicating it is a relatively young genus within the Halosphaeriaceae (Fig. 5).

#### Saagaromyces ratnagiriensis

Taxon classificationFungiMicroascalesHalosphaeriaceae

(S.D. Patil & Borse) K.L. Pang & E.B.G. Jones, Mycol. Progr. 2 (1): 35 (2003)

3257A41F-D073-526A-86B8-C2E3B6A2AF3B

Index Fungorum: IF373274

Fig. 38

Halosarpheia ratnagiriensis S.D. Patil & Borse, Indian Botanical Reporter: 102 (1982). Basionym.
Littispora ratnagiriensis (S.D. Patil & Borse) J. Campb., J.L. Anderson & Shearer, Mycologia 95 (3): 549 (2003). Synonym.

##### Description.

Saprobic on decaying mangrove wood. **Sexual morph. *Ascomata*** 350–600 µm high, 170–560 diam., globose, sub-globose to ellipsoidal, membranous, immersed, hyaline, ostiolate. ***Necks*** 350–900 µm long, 100–175 µm diam., cylindrical, emerging with whitish, fizzy tips over the wood surface, centrally or laterally inserted; ostiolar canal periphysate. ***Peridium*** 20–50 µm diam., with 2–4 layers of *textura angularis*. ***Catenophyses*** present. ***Asci*** 155–230 × 40–65 µm, thin-walled, thicker below the apex, unitunicate, 8-spored, clavate to ellipsoid, long-pedicellate. ***Ascospores*** 50–65 × 17–25 µm, hyaline, oblong to obovate, walls up to 0.1 µm thick, 1-septate, slightly constricted at the septum, with granular deposits and apical to subapical appendages. ***Appendages*** cap-like, stiff and homogenous, one attached at each apex and run partially along the side of the spore, 20–40 µm long, 5–15 µm wide, at maturity becoming soft and scoop-like, eventually transforming into a coil of delicate fibers that uncoil and form sticky filaments, but remain attached to the ascospore apices with their thick bases. **Asexual morph**. Undetermined.

**Figure 38.**
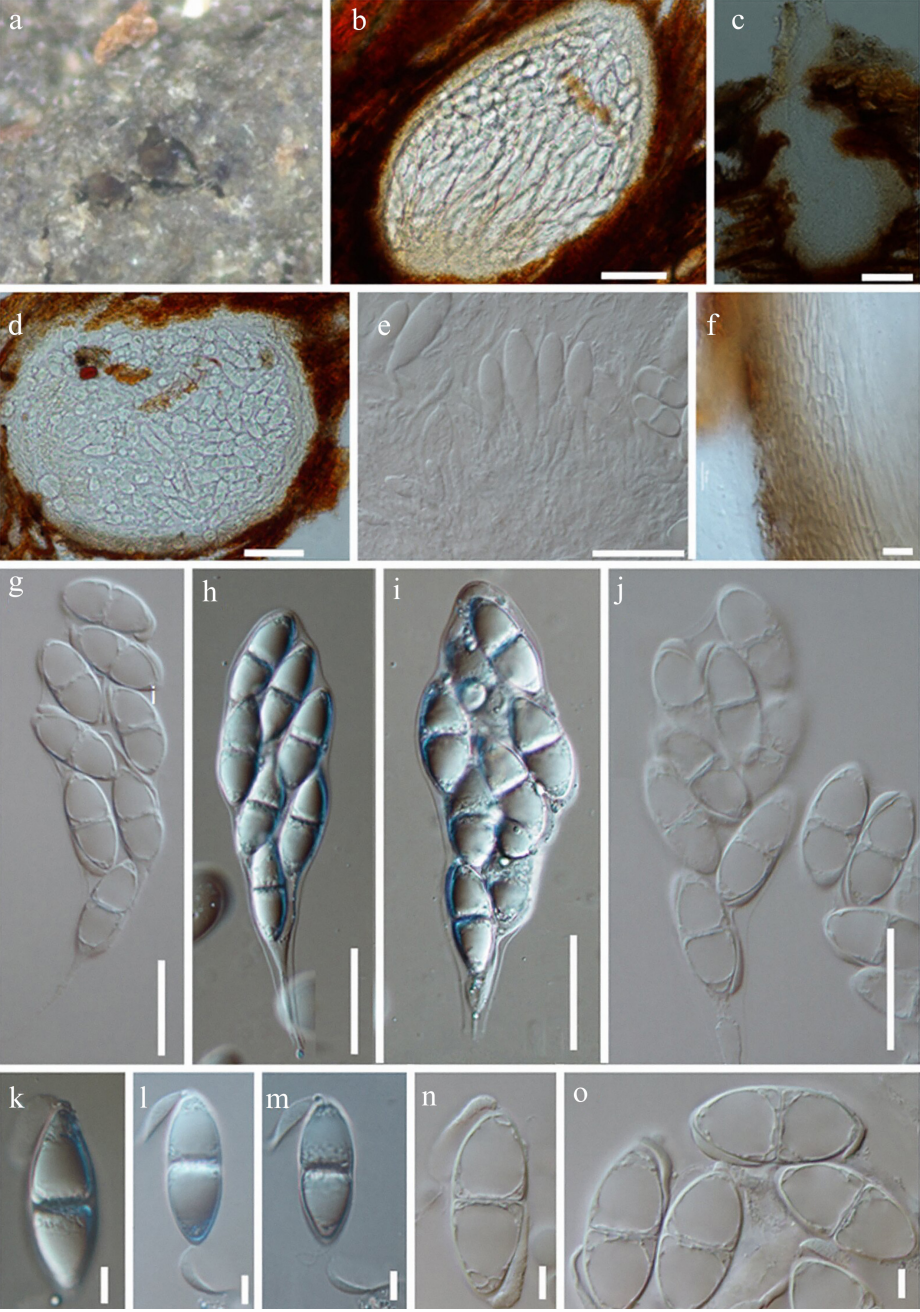
*Saagaromyces ratnagiriensis*. **a**. Ascomata immersed in the decaying wood of *Rhizophora mucronata*; **b, d**. Longitudinal sections of immersed ascomata; **c**. Ostiole; **e**. Paraphyses; **f**. Section showing peridium; **g–j**. Immature and mature asci; **k–o**. Ascospores with bipolar appendages. Scale bars: 100 μm (**b–d**); 50 μm (**e–j**); 10 μm (**k–o**).

##### Material examined.

India, • Puducherry, Thengaithittu mangroves (11.5°N, 79.5°E), on decaying wood of *Rhizophora mucronata* (Rhizophoraceae), 20 January 2017, B. Devadatha (AMH-10010).

##### Notes.

The present collection of *Saagaromyces ratnagiriensis*, from Pondicherry mangroves, shares similar morphological characteristics with the type material reported by Patil and Borse (1982). Campbell et al. (2003) proposed the name *Littispora ratnagiriensis*, but was invalid as the generic name *Saagaromyces* had been proposed earlier by Pang et al. (2003a) and had priority. A widely collected species in the tropics on a wide range of substrates.

##### Distribution.

Andaman Islands, Australia, Bahamas, Belize, Bermuda, Brunei, China. Egypt, Hong Kong, India, Indonesia, Japan, Malaysia. Mauritius, Mexico, Seychelles, South Africa, Taiwan, Thailand, USA.

#### Saagaromyces abonnis

Taxon classificationFungiMicroascalesHalosphaeriaceae

(Kohlm.) K.L. Pang & E.B.G. Jones, Mycol. Progr. 2 (1): 35 (2003)

D37C3F1F-2F62-5672-A5BC-8994DF0C886E

Index Fungorum: IF373272

Halosarpheia abonnis Kohlm., Mar. Ecol. 5 (4): 339 (1984). Basionym.
Littispora abonnis (Kohlm.) J. Campb., J.L. Anderson & Shearer, Mycologia 95 (3): 549 (2003). Synonym.

##### Description.

Saprobic on decaying mangrove wood. **Sexual morph. *Ascomata*** 640–950 µm high, 340–953 µm diam., ellipsoidal, light-brown, coriaceous, immersed, ostiolate, papillate. ***Necks*** 280–530 µm long, 80–180 µm diam., periphysate. ***Peridium*** 30–50 µm thick, two-layered, composed of an outer layer of small, subglobose cells forming a *textura angularis*, an inner layer of large, elongate and flattened cells. ***Catenophyses*** present. ***Asci*** 225–295 × 40–65 µm, thin-walled, unitunicate, 8-spored, clavate, persistent, thickened at the apex, pedicellate. ***Ascospores*** 45–55 × 20–25 µm, hyaline, ellipsoidal, 1-septate, slightly constricted at the septum, appendaged. ***Appendages*** bipolar, thick, hamate extending to the mid-septum, unfurling into fine threads in water. **Asexual morph**. Undetermined.

##### Material examined.

India, • Puducherry, Veerampattinam mangroves (11.5°N, 79.5°E), on decaying wood of *Avicennia marina* (Acanthaceae), 8 October 2017, B. Devadatha (AMH-10011).

##### Notes.

This species was initially described as a *Halosarpheia* species, but the large ascospores, prominent bipolar appendages and a long ascus stalk distinguish it from species in that genus. Molecular data confirm that it is not monophyletic with *Halosarpheia*, and a new genus *Saagaromyces* was proposed.

##### Distribution.

Belize, Bermuda, Hawaii (USA), Mexico.

#### Saagaromyces glitra

Taxon classificationFungiMicroascalesHalosphaeriaceae

(J.L. Crane & Shearer) K.L. Pang & E.B.G. Jones, Mycol. Progr. 2 (1): 35 (2003)

6AD670C7-6B53-5733-998D-35FFAE8B6176

Index Fungorum: IF373273

Fig. 39

Naïs glitra J.L. Crane & Shearer, Trans. Brit. Mycol. Soc. 86 (3): 509 (1986). Basionym.

##### Description.

Saprobic on decaying mangrove wood. **Sexual morph. *Ascomata*** 470–1000 µm high, 250–450 µm diam., globose to subglobose, cream-colored becoming black with age, membranous, immersed, ostiolate. ***Necks*** 150–450 µm long, 100–200 µm diam., dark brown at base becoming light brown to subhyaline at apex, periphysate. ***Peridium*** 30–70 µm diam. two-layered, with an outer layer of cells of *textura angularis* and an inner layer of elongate cells with large lumina. ***Catenophyses*** present. ***Asci*** 175–230 × 15–60 µm, thin-walled, unitunicate, 8-spored, clavate to ellipsoid, deliquescing at maturity, with an apical pore, long pedicellate. ***Ascospores*** 25–42 × 18–25 µm, hyaline, oblong to obovate, walls up to 0.1 µm thick, 1-septate, slightly constricted at the septum, with granular deposits, one large globule in each cell, apical cell may be either equal in length and width or longer (1.0–15.6 µm) and broader (1.0–6.9 µm) than the basal cell, not appendaged.

**Figure 39.**
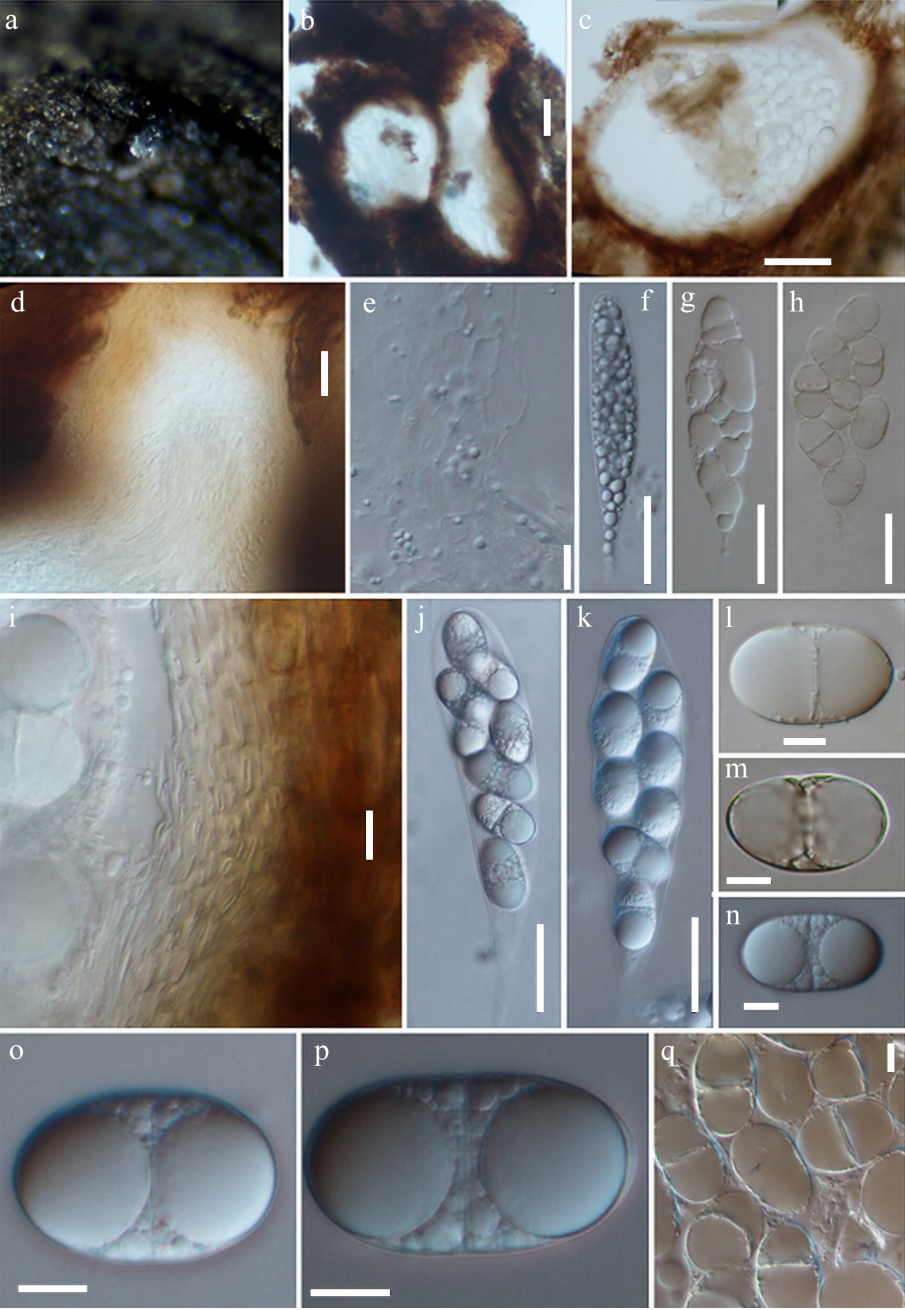
*Saagaromyces glitra*. **a**. Ascomata immersed in decaying wood of *Avicennia marina*; **b, c**. Longitudinal sections of immersed ascomata; **d**. Ostiole; **e**. Paraphyses; **f–h, j, k**. Immature and mature asci; **i**. Section showing peridium; **l–q**. Hyaline, ellipsoidal ascospores with many globules. Scale bars: 100 μm (**b–d**); 10 μm (**e–q**).

##### Material examined.

India, • Tamil Nadu, Tiruvarur, Muthupet mangroves (10.4°N, 79.5°E), on decaying wood of *Avicennia marina* (Acanthaceae), 15 August 2015, B. Devadatha (AMH-10003).

##### Notes.

*Saagaromyces glitra* was first introduced as *Naïs* based on its characteristic small oil globules around the middle septum of the ascospores (Crane and Shearer 1986). Molecular phylogeny results suggested that it clustered together with *Halosarpheia abonnis* (≡ *S. abonnis*) and *H. ratnagiriensis* (≡ *S. ratnagiriensis*), therefore it was transferred to *Saagaromyces* (Pang et al. 2003b). *Saagaromyces glitra* resembles *Naïs inornata* in having hyaline, 1-septate ascospores with small oil globules along the septum. However, the former is distinguishable from the latter in possessing relatively larger cylindrical-clavate asci with a long pedicel and an apical pore and highly guttulated ascospores with one large globule in each cell. While the other *Saagaromyces* species differs from *S. glitra* in having bipolar appendages and lack large globules which suggests that unfurling ascospore appendages are not vital in the delineation of genera in the Halosphaeriaceae.

##### Distribution.

Andaman Islands, Australia, Brunei, Hong Kong, India, Indonesia, Malaysia, Singapore, South Africa, Thailand, USA.

### *Sablicola* E.B.G. Jones, K.L. Pang & Vrijmoed

A monotypic genus described from driftwood and trapped wood between rocks collected at an island in southern China. The fungus was later collected inside the Arctic circle in Norway (Pang et al. 2011a).

#### Sablicola

Taxon classificationFungiMicroascalesHalosphaeriaceae

E.B.G. Jones, K.L. Pang & Vrijmoed, Canad. J. Bot. 82 (4): 486 (2004)

6B657731-26F0-56D7-A15E-94ECAA8D6844

Index Fungorum: IF28801

##### Description.

Saprobic on wood. **Sexual morph. *Ascomata*** light-colored, coriaceous. ***Catenophyses*** absent. ***Asci*** clavate, thin-walled, unitunicate, 8-spored, deliquescing, developing at the base of ascoma venter. ***Ascospores*** ellipsoidal, thin-walled, with one appendage at each pole and four equatorial appendages of equal dimension. ***Appendages*** flattened, attenuate and strap-like with parallel striations, later splitting into fine threads. **Asexual morph**. Undetermined.

##### Type species.

*Sablicola chinensis* E.B.G. Jones, K.L. Pang & Vrijmoed, Canad. J. Bot. 82 (4): 486 (2004).

##### Notes.

*Sablicola* (*S. chinensis*) possesses hyaline, membranous ascomata, early deliquescing asci and ascospores with polar and equatorial appendages, characteristics of the Halosphaeriaceae (Kohlmeyer and Kohlmeyer 1979). The obclavate and striated ascospore appendages of *S. chinensis* which taper to a fine point and become fibrillary in seawater are unique in the family, and so a new genus was proposed for this species (Pang et al. 2004b).

##### Molecular evaluation.

*Sablicola chinensis* formed an unsupported relationship with *Remispora* spp. (Sakayaroj et al. 2011). In this study (Fig. 4), this species constituted a branch with no close relatives. Further collections with a wider range of genes are required to confirm its taxonomic position. Divergence-time analysis places the *Sablicola* lineage in the Paleogene–Neogene interval (~30 MYA, 95% CI: 19.7–44.7 MYA) (Fig. 5).

#### Sablicola chinensis

Taxon classificationFungiMicroascalesHalosphaeriaceae

E.B.G. Jones, K.L. Pang & Vrijmoed, Canad. J. Bot. 82 (4): 486 (2004)

E8949C18-DAF4-5181-B0A4-CCF2A81C311A

Index Fungorum: IF370532

##### Description.

Saprobic on wood. **Sexual morph. *Ascomata*** 100–(176)–288 × 100–(132)–160 µm, solitary, hyaline, yellow to brown, ellipsoidal to subglobose, immersed or partly immersed, and coriaceous. ***Peridium*** 14–(24)–40 µm of *textura angularis*. ***Necks*** 32–(38)–52 × 24–(36)–48 µm. ***Catenophyses*** absent. ***Asci*** 70 × 29 µm, clavate, pedunculate, thin-walled, unitunicate, 8-spored and deliquescing or persistent, developing at the base of the ascoma venter. ***Ascospores*** 18–(20)–24 × 10–(12)–14 µm, ellipsoidal, hyaline, thin-walled, 1-septate, with one appendage at each pole and four at the equatorial position, of equal dimension. ***Appendages*** 13–(17)–22 × 3–(4)–6 µm, flattened, attenuate and strap-like with parallel striations, later splitting into fine threads. **Asexual morph**. Undetermined.

##### Notes.

*Sablicola chinensis* is saprobic on wood (Pang et al. 2004b).

##### Distribution.

China, Norway, Singapore.

### *Safagamyces* Bakhit & Abdel-Wahab

The monotypic genus *Safagamyces* was introduced for a marine. Asexual species *Safagamyces marinus* which was described from decaying stem of *Phragmites australis* inside Safaga mangrove, Red Sea, Egypt (Bakhit and Abdel-Wahab 2022). The genus is characterized by straight or slightly curved, branched, smooth, 2–6 septate conidia and sympodial conidial proliferation. Conidial cells increase in size and pigmentation from hyaline at the base to dark brown at the apex (Bakhit and Abdel-Wahab 2022).

#### Safagamyces

Taxon classificationFungiMicroascalesHalosphaeriaceae

Bakhit & Abdel-Wahab, Phytotaxa 568 (2): 223 (2022)

A5475A0C-2BA7-55D9-89EE-676991F7F52D

Index Fungorum: IF844732

##### Description.

Saprobic on decaying stems of *Phragmites australis* in marine mangrove. **Asexual morph. *Hyphae*** septate, rarely branched, superficial and immersed, hyaline to light-brown, smooth. ***Conidiophores*** micronematous, simple, cylindrical, hyaline, smooth, present or obsolete. ***Conidiogenesis*** holoblastic with sympodial conidial proliferation. ***Conidia*** straight or slightly curved, branched, smooth, variable in shape, septate, strongly constricted at the septa, cells increase in size and pigmentation from the base to the apex; apical cells globose to subglobose, brown to dark-brown, thick walled, smooth; basal cells, cylindrical to clavate, hyaline. **Sexual morph**. Undetermined.

##### Type species.

*Safagamyces marinus* Bakhit & Abdel-Wahab, Phytotaxa 568 (2): 225 (2022).

##### Notes.

Several marine fungi have conidia varying from helicoid to those composed of a mass of cells when mature, that are somewhat similar to *Safagamyces*, e.g. *Cirrenalia*, *Cucurbitinus*, *Cumulospora*, *Juncigena*, *Moleospora* and *Moromyces*. Phylogenetic analysis placed *Cumulospora*, *Moleospora* and *Moromyces* in Lulworthiales and *Juncigena* in Torpedosporales (Abdel-Wahab et al. 2010; Maharachchikumbura et al. 2015; Hyde et al. 2020b). Phylogenetic analyses placed *Safagamyces marinus* in Halosphaeriaceae and formed a basal branch to a node that contains *Pseudolignincola* in a well-supported clade. Morphologically, *Safagamyces* shares common characteristics with *Cirrenalia* and *Cucurbitinus* in having conidial cells constricted at the septa and increasing in size and pigmentation from the base to the apex (Meyers and Moore 1960; Liu et al. 2020). However, *Safagamyces* differs from the other two genera by having branched conidia with sympodial conidial proliferation. *Pseudolignincola* differs from *Safagamyces* in having unicellular, dark-brown conidia and its teleomorphic stage has clavate asci with truncate, thickened apex, a pore and plasmalemma retraction and cylindrical ascospores without appendages (Jones et al. 2006).

##### Molecular evaluation.

Phylogenetic analysis of the combined 18S and 28S rDNA sequences dataset indicated that *S. marinus* forms a distinct branch basal to a node that contains the three genera: *Cirrenalia*, *Cucurbitinus* and *Pseudolignincola* in the family Halosphaeriaceae (Bakhit and Abdel-Wahab 2022). In this study, *Safagamyces marinus* formed a sister clade to *Pseudolignincola siamensis* (Fig. 4), suggestive of closely related (congeneric?) species but not in accordance with the morphological data. Divergence-time analysis places the *Safagamyces*–*Pseudolignincola* split in the Early Cretaceous (~125 MYA, 95% CI: 28.9–501.5 MYA), highlighting a long evolutionary separation (Fig. 5).

#### Safagamyces marinus

Taxon classificationFungiMicroascalesHalosphaeriaceae

Bakhit & Abdel-Wahab, Phytotaxa 568 (2): 225 (2022)

7B8BAB33-6C0A-5306-B682-86B976126A40

Index Fungorum: IF844733

##### Description.

Saprobic on decaying stems of *Phragmites australis*. **Asexual morph. *Hyphae*** 2–3 μm diam., septate, rarely branched, smooth, hyaline to light-brown, superficial and immersed. ***Conidiophores*** 8–15 μm long, 3–4.5 μm in diam., 0- to 1-septate, micronematous, simple, cylindrical, smooth, hyaline, present or obsolete. ***Conidiogenesis*** holoblastic with sympodial conidial proliferation. ***Conidia*** 16–30 μm long, 10–29 μm wide, straight or slightly curved, branched, smooth, variable in shape, 2–6 septate, strongly constricted at the septa, cells increase in size and pigmentation from the base to the apex; apical cells globose to subglobose, 8–11.5 μm wide, brown to dark-brown, thick-walled, smooth; basal cells 3–6.5 μm long × 2.5–5.5 μm wide, cylindrical to clavate, hyaline. **Sexual morph**. Undetermined.

##### Notes.

*Safagamyces marinus* differs from *Remisporiopsis macrocephala* by having straight or slightly curved, branched conidia that are brown in color with sympodial conidial proliferation. Conidia in *R. macrocephala* are helicoid, reddish fuscous with determinate conidiogenesis cells (Meyers and Moore 1960; Zhao et al. 2007). *Safagamyces marinus* and the two *Cucurbitinus* species have straight or slightly curved conidia with constricted septa, however, *S. marinus* was phylogenetically distant from *Cucurbitinus* and has different conidial morphology. *Cucurbitinus constrictus* has much longer conidia than *S. marinus* and larger apical cells. Conidia in *S. marinus* are branched and brown in color, while conidia in *C. constrictus* are unbranched and reddish-brown in color (Schmidt 1985; Kohlmeyer and Volkmann-Kohlmeyer 1991a; Liu et al. 2020). *Cucurbitinus ibericus* has unbranched conidia that are larger than those of *S. marinus*.

*Safagamyces marinus* is saprobic on decaying stems of *Phragmites australis*, Safaga mangrove, Red Sea (Bakhit and Abdel-Wahab 2022).

##### Material examined.

Egypt, • Safaga, Red Sea (26°23'58"N, 34°06'54"E), decaying stems of *Phragmites australis* inside mangrove site, 3 November 2020, Coll. M.A. Abdel-Wahab, holotype (SUMCC H-20001, holotype).

##### Distribution.

Red Sea Egypt.

### *Sheareromyces* Abdel-Wahab, Maharachch. & E.B.G. Jones, gen. nov.

A new genus is designated for *Aniptodera aquibella* (Li et al. 2016), as it does not belong in *Aniptodera* based on sequencing data.

#### Sheareromyces

Taxon classificationFungiMicroascalesHalosphaeriaceae

Abdel-Wahab, Maharachch. & E.B.G. Jones gen. nov.

ECC8C143-BB8B-5A89-AD3E-F9A5FDCD2628

Index Fungorum: IF904269

##### Etymology.

In honor of marine mycologist Carol Shearer and dominative ella.

##### Description.

Saprobic on decaying, submerged twigs in freshwater habitats. **Sexual morph. *Ascomata*** superficial or immersed, globose or subglobose, scattered, hyaline or greyish, membranous. ***Necks*** cylindrical to conical, hyaline, periphysate. ***Peridium*** composed of several layers of hyaline walled cells of *textura globulosa*. ***Catenophyses*** sparse, hyaline, septate, consisting of elongated cells, slightly constricted at the septa. ***Asci*** 8-spored, thin-walled, clavate, becoming balloon-shaped or swollen, flattened at apex, tapering to a pointed pedicel, unitunicate, wall thickened at the apex, subapical cytoplasm retracted, mostly persistent, with a J-, apical thickening, which has an apical pore. ***Ascospores*** 1-euseptate, slightly constricted at the septa, thin-walled, hyaline, smooth-walled, ellipsoidal, 2–3-seriate, guttulate, sometimes with indistinct appendages at both ends. **Asexual morph**. Undetermined.

##### Type species.

*Sheareromyces aquibella* (J. Yang & K.D. Hyde) Abdel-Wahab, Maharachch. & E.B.G. Jones.

#### Sheareromyces aquibella

Taxon classificationFungiMicroascalesHalosphaeriaceae

(J. Yang & K.D. Hyde) Abdel-Wahab, Maharachch. & E.B.G. Jones comb. nov.

4CD85CBA-4459-51A9-ABED-443F9A578080

Index Fungorum: IF904270

##### Etymology.

From the Latin aqua = water, bellus = lovely, referring to the freshwater habitat.

##### Holotype.

MFLU 15–1140.

##### Description.

Saprobic on decaying, submerged twigs in freshwater habitats. **Sexual morph. *Ascomata*** 130–160 × 150–200 μm, superficial or immersed, globose or subglobose, scattered, hyaline or greyish, membranous. ***Necks*** 80–110 × 40–60 μm, cylindrical to conical, hyaline, with periphyses. ***Peridium*** 7–15 μm thick, composing several layers of hyaline-walled cells of *textura globulosa*. ***Catenophyses*** sparse, hyaline, septate, consisting of elongated cells, slightly constricted at the septa. ***Asci*** 60–110 × 25–45 μm, 8-spored, thin-walled, clavate, becoming balloon-shaped or swollen, flattened at apex, tapering to a pointed pedicel, unitunicate, wall thickened at the apex, subapical cytoplasm retracted, mostly persistent, with a J-, apical thickening, which has an apical pore. ***Ascospores*** 25–30 × 7–10 μm, 1-euseptate, slightly constricted at the septa, thin-walled, hyaline, smooth-walled, ellipsoidal, 2–3-seriate, guttulate, sometimes with indistinct appendages at both ends. **Asexual morph**. Undetermined. Description based on Yang and Hyde, in Li et al. (2016).

##### Culture characteristics.

Ascospores germinating on PDA within 24 h and germ tubes produced from both polar cells. Colony on MEA slow-growing, reaching 5–10 mm diam. at 14 days, dark brown in the middle, conspicuous paler and sparser at edge, with dense white mycelium on the surface in the middle of the colony; in reverse with a dark brown middle and olive-green smooth margin. Mycelium immersed and superficial in the media, composed of branched, septate, smooth-walled, hyaline aerial hyphae and dark brown hyphae near or within the media.

##### Distribution.

On submerged wood in freshwater, Thailand.

##### Type material.

Thailand, • Prachuap Khiri Khan Province, Hua Hin, Kaeng Krachan, near Pala-U Waterfall, stream outside national park, on submerged wood, 25 December 2014, Jaap van strien (MFLU 15–1140, holotype), ex-type living culture, MFLUCC 15–0605, GZCC 15–0055.

##### Notes.

The genus *Aniptodera* was established by Shearer and Miller (1977) with *A. chesapeakensis* as the type species, with hyaline or light colored ascomata, catenophyses, apically thickened persistent asci with a distinct pore and subapical retraction of cytoplasm, and hyaline, thick-walled, 1-septate ascospores with or without appendages (Shearer and Miller 1977; Raja and Shearer 2008). *Sheareromyces aquibella* differs from *Aniptodera chesapeakensis* as follows: peridium consists of *textura globulosa* cells, asci are clavate to balloon-shaped while the ascospores are thin-walled and with bipolar appendages. In Luo et al. (2019) and Bao et al. (2023), the species *A. aquibella* formed a sister clade to *A. chesapeakensis*, although this relationship was supported by only weak statistical support. Furthermore, in the current study, *A. aquibella* does not cluster within the *Aniptodera* clade, a finding consistent with Li et al. (2016). *Sheareromyces aquibella* is known only from freshwater habitats, while *A. chesapeakensis* has been reported from marine and freshwater habitats. Consequently, a new genus is introduced for *A. aquibella*.

The genus *Aniptodera* is characterised by thick- and thin-walled ascomata and species with both appendaged and non-appendaged ascospores, which has led to confusion in the delineation of the species (Volkmann-Kohlmeyer and Kohlmeyer 1994). Sakayaroj et al. (2011) and Jones et al. (2017) demonstrated that the genus is polyphyletic, resulting in the transfer of many *Aniptodera* species to new genera: *Aniptosporopsis* and *Paraaniptodera* (Jones et al. 2017), and *Pangia* and *Shiiraspora* in this monograph. Other *Aniptodera* species lack sequence data and need recollection, isolation, and sequencing to resolve their position in the genus.

#### Shiiraspora

Taxon classificationFungiMicroascalesHalosphaeriaceae

(Nakagiri & Tad. Ito) P. Corriea, Abdel-Wahab & E.B.G. Jones gen. nov.

BD03A0EB-8B77-52F8-B3E1-0E98F953D8B0

Index Fungorum: IF903256

##### Etymology.

Named after the Shiira River where the fungus was collected.

##### Description.

Saprobic on decaying mangrove wood. **Sexual morph. *Ascomata*** ellipsoidal to flattened, horizontal, black upper side, hyaline bottom, solitary, immersed, ostiolate, papillate. ***Necks*** lateral, bending upward, cylindrical, hyaline to black in color, periphysate. ***Peridium*** membranous, composed in the upper region of black elongate cells, interspersed among host cells; in the lower region of hyaline flat cells. ***Catenophyses*** present. ***Asci*** 8-spored, cylindrical to clavate, pedunculate, unitunicate, thin-walled, persistent, apex truncate with an apical thickening (ring) with a pore, with cytoplasmic retraction in the subapical region. Asci develop in a hymenium at the base of the ascocarp and are easily detached from the base and released into the ascocarp venter. ***Ascospores*** ellipsoidal to fusiform, hyaline, 1-septate, not constricted at the septum, with apical appendages. ***Appendages*** attached apically at both ends, initially stiff, unfurling in seawater into a membranous structure. **Asexual morph**. Undetermined.

##### Type species.

*Shiiraspora salsuginosa* (Nakagiri & Tad. Ito) P. Corriea, Abdel-Wahab & E.B.G. Jones.

##### Notes.

Multi-gene phylogeny of Halosphaeriaceae (Fig. 4) in this study placed *Aniptodera salsuginosa* in a weakly supported clade with *Antennospora quadricornuta* and *Arenariomyces* spp. that is phylogenetically distant from the type species *Aniptodera chesapeakensis*. As a result, we erected the new genus *Shiiraspora* to accommodate the taxon.

##### Molecular evaluation.

The multi-gene phylogenetic analysis (Fig. 4) revealed that *S. salsuginosa* formed a separate branch from *Antennospora* and *Arenariomyces*. Divergence-time analysis indicates that *Shiiraspora* originated in the Early Cretaceous (~128 MYA, 95% CI: 37.7–437.3 MYA), supporting its recognition as an independent genus within the Halosphaeriaceae (Fig. 5).

#### Shiiraspora salsuginosa

Taxon classificationFungiMicroascalesHalosphaeriaceae

(Nakagiri & Tad. Ito) P. Correia, Abdel-Wahab & E.B.G. Jones comb. nov.

5A020726-78E6-5380-9917-3F34021A0B05

Index Fungorum: IF903257

Aniptodera salsuginosa Nakagiri & Tad. Ito, Mycol. Res. 98: 931 (1994). Basionym.

##### Description.

Saprobic on decaying mangrove wood. **Sexual morph. *Ascomata*** 240–280 µm diam., 92–112 µm high, ellipsoidal to flattened, horizontal, black upper side, hyaline bottom, solitary, immersed just below the epidermis of bark, ostiolate, papillate. ***Necks*** lateral, bending upward, 380–680 µm long, 35–48 µm diam., cylindrical, hyaline to subhyaline in the upper region, black in the lower region, periphysate. ***Peridium*** 6–10 µm thick, membranous, composed in the upper region of black elongate cells, interspersed among host cells; in the lower region of hyaline flat cells. ***Catenophyses*** present. ***Asci*** 76–90 × 12–14 µm, 8-spored, cylindrical to clavate, pedunculate, unitunicate, thin-walled, persistent, apex truncate with an apical thickening (ring) with a pore, with cytoplasmic retraction in the subapical region. Asci develop in a hymenium at the base of the ascocarp and are easily detached from the base and released into the ascocarp venter. ***Ascospores*** 12–¬23 × 4–7 µm, ellipsoidal to fusiform, hyaline, 1-septate, not constricted at the septum, with apical appendages. ***Appendages*** attached apically at both ends, initially stiff, 9–14 µm long, unfurling in seawater into a membranous structure. **Asexual morph**. Undetermined.

##### Material examined.

Japan, • Iriomote Island, Shiira River, decomposing wood of *Bruguiera gymnorrhiza*, 26 Nov. 1991, A. Nakagiri (IFO H-12161, holotype).

##### Notes.

*Shiiraspora salsuginosa* differs from *A. chesapeakensis* by having black ascomata with horizontal axis, persistent asci with a ring and pore and thin-walled ascospores with polar appendage that unfurl in water to form membranous structure. *Aniptodera chesapeakensis* has hyaline, membranous ascomata, early deliquescing asci and thick-walled ascospores with or without bipolar appendages that unfurl in water to form thin, long filaments (Shearer and Miller 1977; Shearer and Crane 1980; Shearer 1989; Nakagiri and Ito 1994).

### *Thalassogena* Kohlm. & Volkm.-Kohlm.

*Thalassogena* is a monotypic marine genus that was established by Kohlmeyer and Volkmann-Kohlmeyer (1987a) to accommodate *Th. sphaerica* that was collected from submerged subtidal wood collected at 3 m depth in Belize. Later the species was frequently recorded from decaying wood of *Rhizophora apiculata* collected at low tide level from Kampong Kapok mangrove, Brunei (Hyde 1990d). *Thalassogena sphaerica* was also recorded from mangroves in Hong Kong and Taiwan (Tsui and Hyde 2004; Pang and Jheng 2012a).

#### Thalassogena

Taxon classificationFungiMicroascalesHalosphaeriaceae

Kohlm. & Volkm.-Kohlm., Syst. Ascomycetum 6: 223 (1987)

CE628E61-93AC-57B2-8170-47E5F8E3C592

Index Fungorum: IF25208

##### Description.

Saprobic on wood. **Sexual morph. *Ascomata*** globose, immersed to superficial, ostiolate, periphysate, papillate, cream-colored, single. ***Peridium*** thin, forming a *textura angularis*. ***Catenophyses*** developing from the thin-walled pseudoparenchyma. ***Asci*** 8-spored, clavate, pedunculate with an apical pore, non-amyloid, thin-walled, unitunicate, persistent, maturing successively on an ascogenous tissue at the bottom of the locule. ***Ascospores*** subglobose, one-celled, hyaline, without sheaths or appendages. **Asexual morph**. Undetermined.

##### Type species.

*Thalassogena sphaerica* Kohlm. & Volkm.-Kohlm., Syst. Ascomycetum 6: 225 (1987).

##### Notes.

Thalassogena is a monotypic marine genus described from plant remains at 3 m depth in Belize (Kohlmeyer and Volkmann-Kohlmeyer 1987a) and it was recorded from mangrove wood at low tide levels in Hong Kong and Taiwan (Hyde 1990d; Tsui and Hyde 2004; Pang and Jheng 2012a). Thalassogena unicellularis was also reported from mangrove (Abdel-Wahab and Nagahama 2011).

##### Molecular evaluation.

Molecular data is not available for *Thalassogena sphaerica* and further collections are required to ensure the phylogenetic position of this genus within Halosphaeriaceae. *Thalassogena unicellularis* formed a strong monophyletic group with *Moana*, *Gesasha* and *Neogesasha* (Fig. 4).

#### Thalassogena sphaerica

Taxon classificationFungiMicroascalesHalosphaeriaceae

Kohlm. & Volkm.-Kohlm., Syst. Ascomycetum 6: 225 (1987)

551F5B2A-D94F-59B6-966F-0366FDD5F606

Index Fungorum: IF132448

Fig. 40

##### Description.

Saprobic on wood. **Sexual morph. *Ascomata*** 150–260 µm diam., subglobose, immersed to superficial, ostiolate, periphysate, papillate, coriaceous, cream-colored, single, surrounded by brown hyphae. ***Necks*** 230–570 µm long, 50–85 µm wide, ostiolar canal filled with periphyses. ***Peridium*** 10–16 µm thick, composed of 4–5 layers of polygonal cells, forming a *textura angularis*, merging with the thin-walled pseudoparenchyma cells. ***Catenophyses*** 3–11 µm diam., developing from the pseudoparenchyma that originally fills the central cavity. ***Asci*** 72–89 × 22–27 µm, 8-spored, clavate, pedunculated, flattened at the apex and with a pore, thin-walled, unitunicate, persistent, maturing successively on an ascogenous tissue at the bottom of the locule. ***Ascospores*** 12.5–16.5 µm, subglobose, rarely ellipsoidal, 1-septate, hyaline, without sheaths or appendages, with one large lipid globule surrounded by numerous small non-lipoid droplets. **Asexual morph**. Undetermined.

**Figure 40.**
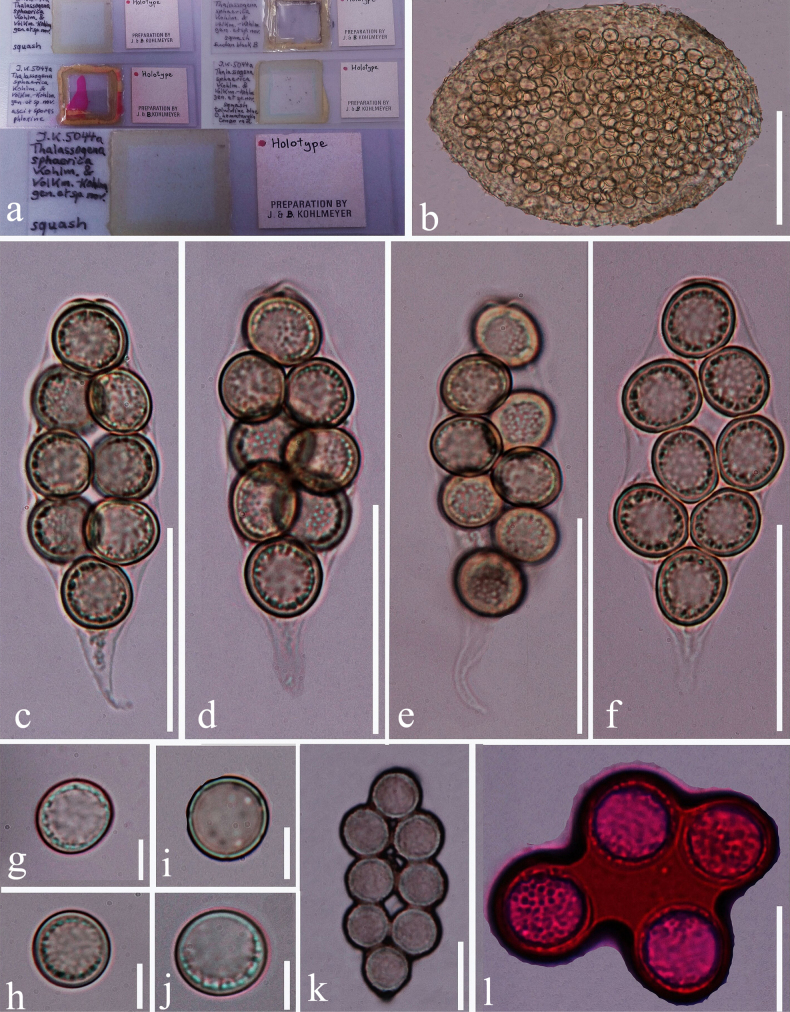
*Thalassogena sphaerica*. **a**. Micro-slides of type material; **b**. Section through ascoma filled with ascospores (NY 1353404); **c–f**. Asci (NY 1353403); **g–j**. Ascospores (NY 1353403); **k**. Asci stained with Sudan Black B (NY 13534060); **l**. Ascospores stained with Phloxine (NY 1353403). Scale bars: 500 μm (**b**); 50 μm (**c–f**); 10 μm (**g–j**); 20 μm (**k, l**).

##### Material examined.

Belize, • Carrie Bow Cay, on submerged tree, riddled by shipworms, in 3 m depth, on the sediment among a patch reef (16°48'N, 88°05'W), 25 May 1987, incubated in moist chamber for 4 months, J. Kohlmeyer (J. K. 5044a IMS, holotype).

##### Notes.

Several taxa in the Halosphaeriaceae are characterized by unicellular ascospores, such as *Anisostagma rotundatum*, *Moana turbinulata*, *Halosarpheia unicellularis*, *Iwilsoniella rotunda* and *Gesasha unicellularis*. Three taxa which have no appendages or sheaths to the ascospores, namely *A. rotundatum*, *I. rotunda* and *G. unicellularis*. No molecular data is available for *A. rotundatum* and *I. rotunda*. This species is worldwide in distribution, but largely from the tropics on dead mangrove wood.

##### Distribution.

Belize, Brunei, Denmark, Hong Kong, Mauritius, Philippines, South Africa, Taiwan, Thailand.

#### Thalassogena unicellularis

Taxon classificationFungiMicroascalesHalosphaeriaceae

(Abdel-Wahab & Nagahama) Abdel-Wahab comb. nov.

875C3BBF-77C3-501A-9A6E-CD0D07385A2E

Index Fungorum: IF903293

Fig. 41

Gesasha unicellularis Abdel-Wahab & Nagah., Nova Hedwigia 92 (3–4): 505 (2011). Basionym.

##### Description.

Saprobic on decaying mangrove wood. **Sexual morph. *Ascomata*** 300–340 μm diam., subglobose, light-brown to brown, coriaceous, immersed or erumpent, solitary, ostiolate with a long hyaline neck. ***Necks*** 200–400 × 50–60 μm, with wide base and pointed tip, hyaline, periphysate. ***Periphyses*** up to 10 μm long and 0.5 μm wide. ***Peridium*** 30–45 μm wide, consisting of 6–12 layers of elongated, thick-walled cells, forming *textura angularis*, cells are hyaline except the first two outside layers which are brown in color. ***Catenophyses*** present. ***Asci*** 36–54 × 10–12 μm, unitunicate, clavate, with apical depression, apical thickening and ring, apically truncate with cytoplasmic retraction, persistent, with eight overlapping biseriate ascospores. ***Ascospores*** 8–11 × 5–7 μm, unicellular, subglobose, ellipsoidal or oval, without appendages, hyaline becoming light brown with age. **Asexual morph**. Undetermined.

**Figure 41.**
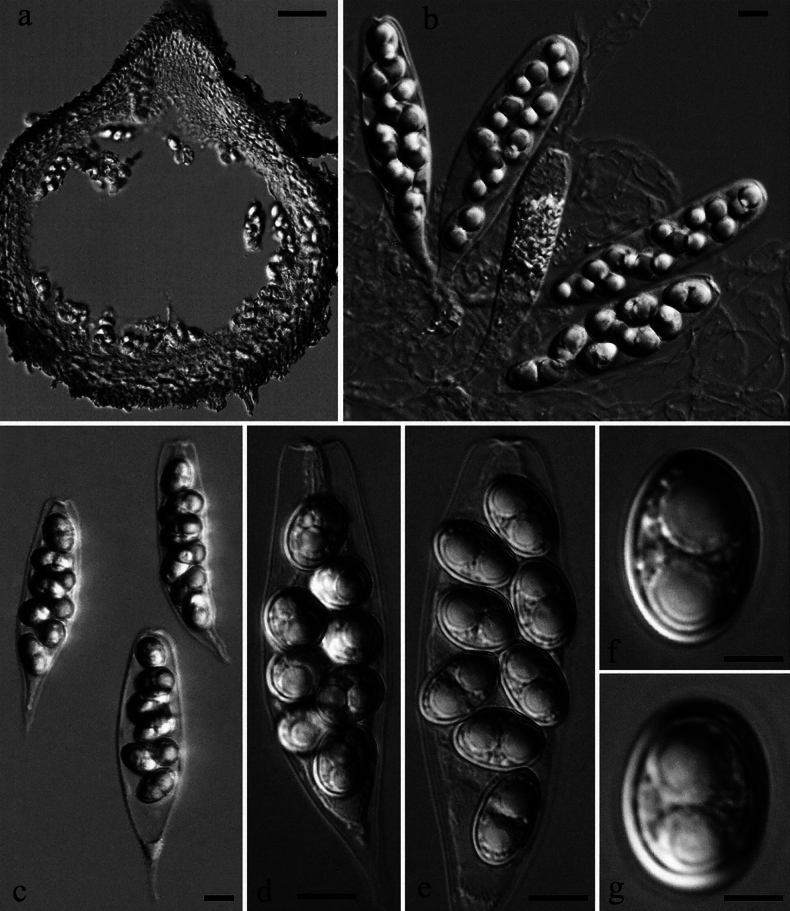
*Thalassogena unicellularis*. **a**. Vertical section through ascoma; **b–e**. Asci at different stages of development; **f**, **g**. Mature ascospores. Scale bars: 50 μm (**a**); 10 μm (**b**, **c**); 5 μm (**d–g**)

##### Material examined.

Japan, • Okinawa, Higashi-son, Gesashi mangroves, on decayed wood in the intertidal zone, July 2008, coll. M. A. Abdel-Wahab (holotype, IMI 397959).

##### Notes.

Our multigene phylogenetic analysis (Fig. 4) placed *Thalassogena unicellularis* (= *Gesasha unicellularis*), and *Moana turbinulata*, in a monophyletic clade with high support bootstrap (100/98/1). However, *Th. unicellularis* cannot be placed in *Moana* because they have significant morphological differences. *Moana turbinulata* has asci without apical apparatus or a pore and ascospores with a single, top-shaped appendage which unfurls in water to produce a long ribbon, while *Th. unicellularis* has persistent asci with apical apparatus and ascospores without appendages (Kohlmeyer and Volkmann-Kohlmeyer 1989; Abdel-Wahab and Nagahama 2011). As *Gesasha unicellularis* did not group with the type species *Gesasha peditatus* but has morphological characteristics that encompass *Thalassogena* we assign it to that genus. *Thalassogena unicellularis* differs from *Th. sphaerica* by having light-brown to brown ascomata, smaller asci (36–54 × 10–12 vs. 72–89 × 22–27 μm for *Th. unicellularis* and *Th. sphaerica*, respectively) and smaller ascospores (8–11 × 5–7 vs. 13–17 μm diam. for *Th. unicellularis* and *Th. sphaerica*, respectively) (Kohlmeyer and Volkmann-Kohlmeyer 1987a; Abdel-Wahab and Nagahama 2011).

### *Thalespora* Chatmala & E.B.G. Jones

The genus *Thalespora* was introduced by Jones et al. (2006) to accommodate the new species *Th. appendiculata* found on driftwood.

#### Thalespora

Taxon classificationFungiMicroascalesHalosphaeriaceae

Chatmala & E.B.G. Jones, Nova Hedwigia 83 (1–2): 228 (2006)

A5357BFD-DDCE-5F57-B2F9-4C4C0F2E9E31

Index Fungorum: IF29054

##### Description.

Saprobic on driftwood. **Sexual morph. *Ascomata*** ellipsoidal, immersed in wood, ostiolate, papillate, coriaceous, light brown and solitary. ***Peridium*** is thin-walled with light brown cells. ***Necks*** cylindrical, slightly inflated at the tip, eccentric, extending above the wood surface. ***Paraphyses*** absent. ***Asci*** 8-spored, ellipsoidal, unitunicate, thin-walled, deliquescing before ascospores mature. ***Ascospores*** hyaline, elongate-fusiform, rounded at the upper pole, tapering toward the base, straight or curved, 1-septate, not or slightly constricted at the septum, appendaged (adapted from Jones et al. 2006). **Asexual morph**. Undetermined.

##### Type species.

*Thalespora appendiculata* Chatmala & E.B.G. Jones, Nova Hedwigia 83 (1–2): 229 (2006).

##### Notes.

*Thalespora* shares similarities with *Bathyascus*, both genera with spathulate, hyaline, unicellular, or 1-septate ascospores that are longer than 100 µm, however, *Bathyascus* species lack apical ascospore appendages. No sequences are available of *Bathyascus* species so their phylogenetic affinities cannot be determined.

##### Molecular evaluation.

Jones et al. (2006) showed that *Th. appendiculata* grouped with isolates of *Okeanomyces cucullatus* and its anamorph *Periconia prolifica* in a well-supported clade with a sister clade comprising the genera *Aniptodera*, *Ascosacculus* and *Nais*. This was confirmed in a phylogenetic study by Jones et al. (2017) of selected genera in the Halosphaeriaceae, in which *Th. appendiculata* grouped in a clade comprising *O. cucullatus*, *Aniptodera chesapeakensis*, and *Ascosacculus aquaticus* and in a sister clade with *Aniptosporopsis lignatilis*. These taxa showed few morphological features in common as *Th. appendiculata* has appendages at one end of the ascospores.

*Thalespora appendiculata* (IT200) formed a sister clade to the *Okeanomyces* clade (Fig. 4). Divergence-time estimates place the split between *Thalespora* and *Okeanomyces* in the Paleogene–Neogene interval (~10 MYA, 95% CI: 7.5–92.6 MYA. With only one isolate of *Th. appendiculata* that has molecular sequence data available for the 28S and 18S rDNA loci, further collections and sequencing of a wider range of genes are required to confirm its position in the Halosphaeriaceae (Fig. 5).

#### Thalespora appendiculata

Taxon classificationFungiMicroascalesHalosphaeriaceae

Chatmala & E.B.G. Jones, Nova Hedwigia 83 (1–2): 229 (2006)

DDCC1C72-1A25-5F0F-B5A0-D79B3895A1DE

Index Fungorum: IF334944

Fig. 42

##### Description.

Saprobic on driftwood. **Sexual morph. *Ascomata*** 133–193 µm high, 70–165 µm diam., ellipsoidal, immersed in wood, ostiolate, papillate, coriaceous, thin-walled, light brown and solitary. ***Peridium*** composed of 2–3 layers of thin-walled, light brown cells, with mature ascospores visible through the wall. ***Necks*** up to 290 µm long, 19–39 µm diam., cylindrical, slightly inflated at the tip, eccentric, extending above the wood surface. ***Paraphyses*** absent. ***Asci*** 102 × 22 µm, 8-spored, ellipsoidal, unitunicate, thin-walled, deliquescing early before the ascospores mature, originating from the base of the ascoma and releasing the spores into the venter. ***Ascospores*** 65–110 × 6.5–8.5 µm, hyaline, elongate-fusiform, rounded at the upper pole, tapering toward the base, straight to slightly curved, 1-septate below the center of the spore, not or slightly constricted at the septum, with 2–4(-5) radial appendages. ***Appendages*** develop from the upper cell of the ascospores after release from the asci and when mounted in seawater. ***Appendages*** 40–65 µm long (adapted from Jones et al. 2006). **Asexual morph**. Undetermined

**Figure 42.**
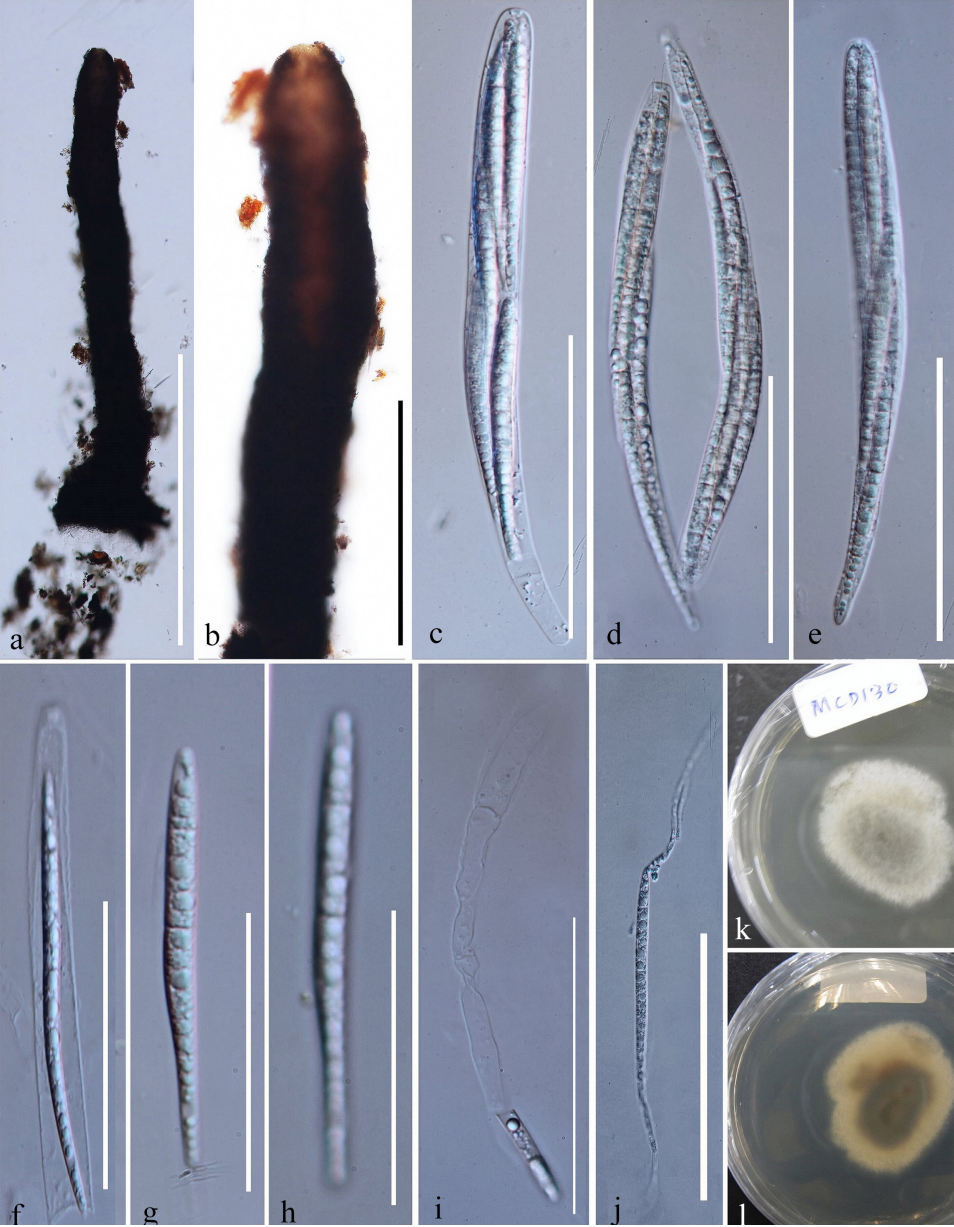
*Thalespora appendiculata*. **a**. Ascomata semi-immersed in decaying wood; **b**. Ascomatal neck; **c–e**. Asci; **f–h**. Ascospores; **i**. Paraphyses; **j**. Germinating ascospore; **k, l**. Culture on potato dextrose agar. Scale bars: 100 μm (**a, b**); 50 μm (**c–h, j**); 20 μm (**i**).

##### Notes.

This species is saprobic, occurring on driftwood.

##### Distribution.

Thailand.

### *Tinhaudeus* K.L. Pang, S.Y. Guo & E.B.G. Jones

A monotypic genus described from decaying mangrove wood in Taiwan (Ariyawansa et al. 2015).

#### Tinhaudeus

Taxon classificationFungiMicroascalesHalosphaeriaceae

K.L. Pang, S.Y. Guo & E.B.G. Jones, Fungal Diversity 75: 160 (2015)

C34AD673-9D1C-5C2F-87D5-6709A76BB839

Index Fungorum: IF812936

##### Description.

Saprobic on intertidal, decaying mangrove wood. **Sexual morph. *Ascomata*** globose to subglobose, immersed, ostiolate, with long necks, coriaceous, light- to dark-colored. ***Necks*** periphysate. ***Peridium*** one-layered, composed of elongate cells with large lumina of *textura angularis*. ***Catenophyses*** present, persistent. ***Asci*** 8-spored, clavate, with a long stalk, unitunicate, thin-walled, slightly thickened at the apex, persistent, developing at the base of the ascoma venter on a convex cushion of ascogenous cells. ***Ascospores*** ellipsoidal, 1-septate, not or slightly constricted at the septum, hyaline. ***Appendages*** bipolar, initially adpressed to the ascospore wall and extended over the mid-septum, unraveling immediately in sea water to form a long thin filament. **Asexual morph**. Undetermined.

##### Type species.

*Tinhaudeus formosanus* K.L. Pang, S.Y. Guo & E.B.G. Jones, Fungal Diversity 75: 164 (2015).

##### Notes.

*Tinhaudeus* (*T. formosanus*) morphologically resembles those genera with plasmalemma retraction of ascus and unfurling ascospore appendages in the Halosphaeriaceae, i.e. *Aniptodera*, *Saagaromyces*, *Phaeonectriella* and *Tirispora*. However, *T. formosanus* differs in having different spore size, coloration, spore wall thickness and ontogeny of ascospore appendages (Ariyawansa et al. 2015). Phylogenetically, *T. formosanus* was also unrelated to these genera based on a combined analysis of the 18S and 28S rDNA (Fig. 4).

##### Molecular evaluation.

*Tinhaudeus* formed an unsupported sister clade with *Sablicola* and *Remispora* (Ariyawansa et al. 2015). In this study, the two strains of *Tinhaudeus formosanus* (NTOU3580, NTOU3805) formed a sister clade to the *Nohea* clade (Fig. 4). Divergence-time analysis places the *Tinhaudeus*–*Nohea* split in the Paleogene (~40 MYA, 95% CI: 34–47.1 MYA), supporting their recognition as independent but relatively young genera within the Halosphaeriaceae (Fig. 5).

#### Tinhaudeus formosanus

Taxon classificationFungiMicroascalesHalosphaeriaceae

K.L. Pang, S.Y. Guo & E.B.G. Jones, Fungal Diversity 75: 164 (2015)

06F6AE21-979A-545E-92DC-1987D696BC26

Index Fungorum: IF812937

##### Description.

Saprobic on intertidal, decaying mangrove wood. **Sexual morph. *Ascomata*** 211–(295)–442 × 114–(180)–263 µm, light- to dark-colored when mature, globose to subglobose, solitary or gregarious, immersed, coriaceous, ostiolate. ***Necks*** 104–(141)–174 µm long, 38–(64)–80 µm diam., periphysate. ***Peridium*** 7–(15)–31 µm, composed of one layer of elongate cells with a large lumina of *textura angularis*. ***Catenophyses*** present, persistent. ***Asci*** 133–(159)–181 × 32–(39)–49 µm, clavate, unitunicate, 8-spored, thin-walled, persistent, with a long stalk, developing from inner wall of ascoma base, with a slightly thickened apex, plasmalemma retracted. ***Ascospores*** 26–(33)–38 × 10–(12)–14 µm, ellipsoidal, hyaline, 1-septate, not or slightly constricted at the septum. ***Appendages*** bipolar, initially adpressed to the ascospore wall and extended over the mid-septum, unraveling immediately in sea water to form a long thin filament. **Asexual morph**. Undetermined.

##### Notes.

This species is saprobic on intertidal, decaying mangrove wood.

##### Distribution.

Taiwan.

### *Tirispora* E.B.G. Jones & Vrijmoed

The genus *Tirispora* was introduced by Jones et al. (1994) to accommodate a saprobic marine fungus species in dead mangrove wood, *Tirispora unicaudata*. Since the introduction of the genus there have been two additions to this genus; *Tirispora mandoviana* introduced by Sarma and Hyde (2000) and *Aniptodera indica* described by Ananda and Sridhar (2001). The latter is reduced to synonymy with *T. unicaudata* (https://www.marinefungi.org).

#### Tirispora

Taxon classificationFungiMicroascalesHalosphaeriaceae

E.B.G. Jones & Vrijmoed, Canad. J. Bot. 72 (9): 1373 (1994)

66AC216D-5EC0-5DC6-8544-F2EA627BAD4F

Index Fungorum: IF27405

##### Description.

Saprobic marine fungi on dead mangrove wood. **Sexual morph. *Ascomata*** globose to subglobose, solitary, superficial, ostiolate, periphysate, papillate, pale to dark brown. ***Peridium*** thin. ***Catenophyses*** present. ***Asci*** 8-spored, clavate, pedunculate, with a ring and apical plate, thin-walled, unitunicate, persistent, maturing successively on ascogenous cushion at base of ascoma. ***Ascospores*** ellipsoid, 1-septate, thick-walled, hyaline with a single appendage at one pole that is initially adpressed to spore wall but unfurls to form a long filamentous thread (Description adapted from Jones et al. 1994). **Asexual morph**. Undetermined.

##### Type species.

*Tirispora unicaudata* E.B.G. Jones & Vrijmoed, Canad. J. Bot. 72 (9): 1373 (1994).

##### Notes.

*Tirispora* is most similar to *Aniptodera*, however they differ in that the former has unipolar, unfurling ascospores appendages.

##### Molecular evaluation.

*Tirispora unicaudata* formed a strongly supported clade with *Pangia* (Fig. 4). However, sequences (ITS, 28S) of one strain are available for *Tirispora unicaudata* but none for *Tirispora mandoviana*. Divergence-time analysis indicates that *Tirispora* diverged from *Pangia* during the Paleogene–Neogene interval (~48 MYA, 95% CI: 12.9–178.3 MYA), suggesting that these genera represent distinct lineages (Fig. 5). New collections, isolates and sequences are required.

#### Tirispora unicaudata

Taxon classificationFungiMicroascalesHalosphaeriaceae

E.B.G. Jones & Vrijmoed, Canad. J. Bot. 72 (9): 1373 (1994)

B33815B1-CF75-5CE5-AD97-F0990D336F05

Index Fungorum: IF363434

Fig. 43

##### Description.

Saprobic on mangrove wood. **Sexual morph. *Ascomata*** 90–300 µm long, 66–216 µm in diam., globose to subglobose, superficial, ostiolate, periphysate, papillate, pale to dark brown, single. ***Necks*** 22–48 µm long 12–46 µm wide. ***Peridium*** thin, 4–6 layers of cells. ***Catenophyses*** present. ***Asci*** 40–80 × 14–28 µm, 8-spored, clavate, pedunculate, with a ring and apical plate, thick-walled, unitunicate, persistent, maturing successively on an ascogenous cushion at the base of the ascomata. ***Ascospores*** 24–32 × 8–12 µm, thick-walled, ellipsoid, 1-septate, hyaline, with a single appendage at one pole, which is initially adpressed to the spore wall but unfurls to form a long filamentous thread (Description adapted from Jones et al. 1994). **Asexual morph**. Undetermined.

**Figure 43.**
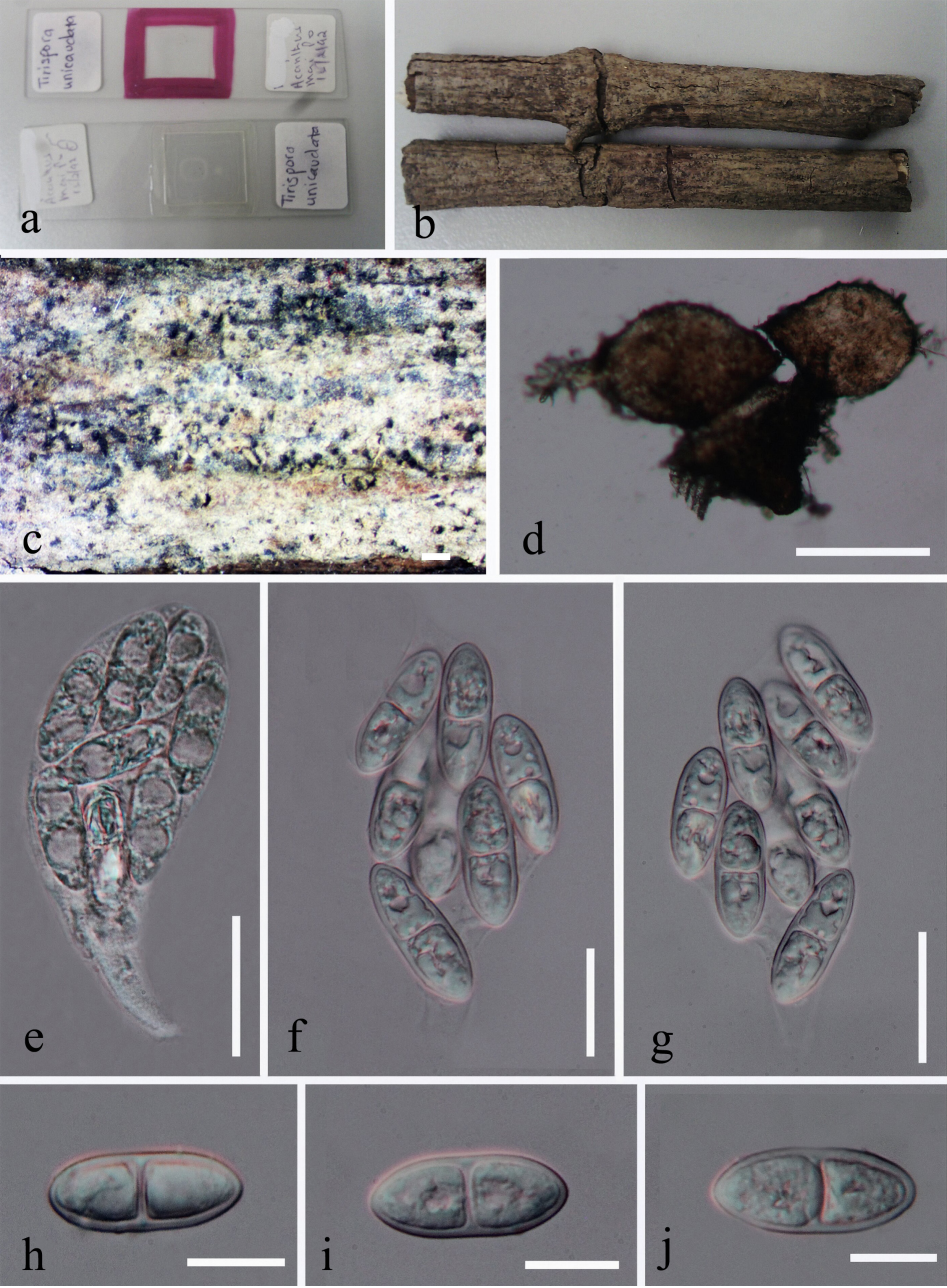
*Tirispora unicaudata* (IMI 359653, holotype). **a**. Micro slide of type material; **b**. Herbarium material; **c**. Fruiting bodies on host surface; **d**. Surface view of fruiting bodies (micro-slide); **e–g**. Asci; **h–j**. Ascospores. Scale bars: 500 µm (**c**); 200 µm (**d**); 20 µm (**e–g**); 10 µm (**h–j**).

##### Notes.

*Tirispora unicaudata* differs from *T. mandoviana* in ascospore measurements, while *T. mandoviana* lacks catenophyses and in the retraction of the plasmalemma in the asci. *Tirispora* species are saprobic on wood in mangrove and freshwater habitats with a tropical distribution in Egypt, Hong Kong, India, Malaysia and Taiwan.

##### Distribution.

Hong Kong, India, Portugal.

### *Toriella* Sakayaroj, K.L. Pang & E.B.G. Jones

*Toriella tubulifera* was initially described as a *Halosphaeria* species (Kohlmeyer 1960), but was transferred to *Ceriosporopsis* as Kohlmeyer (1972) did not regard that marine ascomycetes were sufficiently well delineated. Johnson et al. (1987) pointed out that a new genus might be required to accommodate *C. tubulifera* and this is supported by molecular data which placed it distantly from the type species *Ceriosporopsis halima* (Sakayaroj et al. 2011).

#### Toriella

Taxon classificationFungiMicroascalesHalosphaeriaceae

Sakayaroj, K.L. Pang & E.B.G. Jones, Fungal Diversity 46: 99 (2011)

838BA657-AB4F-5726-B7C2-049F7FBFDF7E

Index Fungorum: IF518774

##### Description.

Saprobic on wood. **Sexual morph. *Ascomata*** globose, cylindrical or elongate, immersed, ostiolate, papillate, coriaceous, brown to black, solitary or gregarious, no catenophyses; ***Asci*** thin-walled, unitunicate, clavate, pedunculate, no apical pore, deliquescing early. ***Ascospores*** hyaline, ellipsoidal, 1-septate, with equatorial and polar appendages. **Asexual morph**. Undetermined.

##### Type species.

*Toriella tubulifera* (Kohlm.) Sakay., K.L. Pang & E.B.G. Jones, Fungal Diversity 46: 100 (2011).

##### Notes.

*Toriella* is a monotypic genus typified by *Toriella tubulifera*, and is accepted in all major classifications (Jones et al. 2015; Maharachchikumbura et al. 2015; Jones et al. 2019).

##### Molecular evaluation.

*Toriella tubulifera* formed a strongly supported group with *Ondiniella torquata* (Fig. 4). Divergence-time analysis places the split between *Toriella* and *Ondiniella* in the late Cretaceous (~72 MYA, 95% CI: 21.1–248 MYA) (Fig. 5).

#### Toriella tubulifera

Taxon classificationFungiMicroascalesHalosphaeriaceae

(Kohlm.) Sakay., K.L. Pang & E.B.G. Jones, Fungal Diversity 46: 100 (2011)

D193F55C-2501-5222-B348-DFF6D83AFEE7

Index Fungorum: IF518775

Fig. 23H

Halosphaeria tubulifera Kohlm., Nova Hedwigia 2: 312 (1960). Basionym.
Ceriosporopsis tubulifera (Kohlm.) P.W. Kirk ex Kohlm., Canad. J. Bot. 50 (9): 1953 (1972). Synonym.

##### Description.

Saprobic on submerged and intertidal wood. **Sexual morph. *Ascomata*** 165–324 µm high, 231–574 µm diam., globose, cylindrical or elongate, immersed, ostiolate, papillate, coriaceous, brown to black, solitary or gregarious, no catenophyses. ***Peridium*** 8–17.5 µm thick, composed of 3–5 layers of cells. ***Necks*** 46.5–165 µm long, 33–73 µm diam. ***Asci*** 8-spored, thin-walled, unitunicate, clavate, pedunculate, no apical pore, deliquescing early. ***Ascospores*** 14.5–23 × 8.5–11 µm, hyaline, ellipsoidal, 1-septate, with equatorial and polar appendages, constricted at the septum. ***Appendage ontogeny*** exosporium folds to form an annulus-like equatorial appendage while the polar appendages are formed inside and the end chamber consisted of two electron-dense layers. **Asexual morph**. Undetermined.

##### Material examined.

Denmark, • (Wales) UK on unidentified intertidal wood (BCC33511, BCC33512, BCC33513, living cultures).

##### Notes.

*Toriella tubulifera* is a saprobic fungus described from submerged and intertidal wood collected from cold-water locations including Canada, Denmark, Germany, Iceland, Sweden, UK, USA and Grytviken, South Georgia, Antarctic (Pugh and Jones 1986; Johnson et al. 1987; Sakayaroj et al. 2011; Tibell et al. 2020). It is reported from a wide range of timbers and on test panels. At South Georgia, Antarctic it was the most frequently recorded species with 37 collections on the submerged test panels.

### *Trichomaris* Hibbits, G.C. Hughes & Sparks

*Trichomaris* is a monotypic genus introduced by Hibbits et al. (1981) to accommodate the species *Trichomaris invadens*, a pathogen of *Chionoecetes bairdi*, a species of snow crab also known as tanner crab. *Trichomaris invadens* forms a dark pigmented encrusting mycelium over the entire exoskeleton and also invades the internal organs (Sparks 1982).

#### Trichomaris

Taxon classificationFungiMicroascalesHalosphaeriaceae

Hibbits, G.C. Hughes & Sparks, Canad. J. Bot. 59 (11): 2123 (1981)

98126AE8-CE2D-5254-A439-89B07A29A830

Index Fungorum: IF5559

##### Description.

Parasitic on *Chionoecetes bairdi* (Tanner crab). **Sexual morph. *Ascomata*** partially immersed in a subiculum, globose, coriaceous, black, with a centric ostiole, that lacks papillae or occasionally when present is short and subcylindrical. Without paraphyses. ***Asci*** elongate, clavate and unitunicate, 8-spored. ***Ascospores*** oblong, 1–3 septate, hyaline, with a hamate appendage at either end of the spore, that unfurls to form fine hair-like threads (based on Hibbits et al. 1981). **Asexual morph**. Undetermined.

##### Type species.

*Trichomaris invadens* Hibbits, G.C. Hughes & Sparks, Canad. J. Bot. 59 (11): 2123 (1981).

##### Notes.

This species shares similarities with various genera in the Halosphaeriaceae, with hyaline, septate, ascospores and hamate appendages unfurling to form fine hair-like sticky threads, such as *Halosarpheia*, *Panorbis* and *Natantispora* (Campbell et al. 2003; Pang et al. 2003b). These grow on wood and other cellulosic substrates making *Trichomaris* the only known obligate parasite in marine Crustacean.

##### Molecular evaluation.

There are no available sequences in databases for *Trichomaris invadens*. *Trichomaris invadens* requires recollection, isolation, and sequencing to determine if it belongs in the Halosphaeriaceae.

#### Trichomaris invadens

Taxon classificationFungiMicroascalesHalosphaeriaceae

Hibbits, G.C. Hughes & Sparks, Canad. J. Bot. 59 (11): 2123 (1981)

EB0969C3-DF9F-5CA3-95E3-8768BADCCF04

Index Fungorum: IF111983

##### Description.

Parasitic on *Chionoecetes bairdi* (Tanner crab), **Sexual morph. *External hyphae*** black, 5.0–5.5 µm in diam., sparingly septate and branched, forming an encrusting subiculum on the carapace of the host. ***Internal hyphae*** found in tissues of the host is nonpigmented, 2.0–2.5 µm in diam., sparingly septate and branched. ***Ascomata*** thick-walled, coriaceous, lacking papilla or occasionally when present, short and subcylindrical, aparaphysate, periphysate, nonstromatic, 250–400 µm in diam., with diaporthe-type centrum. ***Asci*** elongate clavate, unitunicate, thin-walled, 85.0–155.0 × 12.0–17.0 µm. ***Ascospores*** hyaline, oblong to ellipsoidal 15.0–22.0 × 4.6–5.0 µm, 1–3 septate, predominantly 1-septate, having hamate appendages at either end, unfurling to form fine hair-like threads, 50.0–360.0 µm in length (Based on Hibbits et al. 1981). **Asexual morph**. Undetermined.

##### Notes.

*Trichomaris invadens*, is a parasite of the tanner crab, previous isolations originated in Alaska. Crabs, that were colonized by this fungus, exhibit symptoms of “discrete black spots on the orange, somewhat iridescent exoskeleton” (Sparks 1982). These black spots eventually cover the entire carapace with hyphae and fruiting bodies that slowly make their way inside the crab via the connective tissue. This prevents moulting and may lead to death due to tissue destruction (Sparks 1982).

##### Distribution.

Alaska.

### *Tubakiella* K.L. Pang & E.B.G. Jones

Tubaki (1968) introduced *Remispora galerita* from wood collected in Japan and originally referred to as *Lentescospora submarina* (*nomen dubium*, see Kohlmeyer and Kohlmeyer 1979). Kohlmeyer (1972) transferred it to *Halosphaeria*, as he was of the opinion that many marine ascomycetes lacked clear generic limits. Jones and Moss (1978) and Jones et al. (1983a) did not accept these opinions and undertook a major revision of genera in the Halosphaeriaceae by studying ascospore appendage ontogeny and development at the TEM and SEM level. This resulted in the introduction of the genus *Tubakiella*, based on ultrastructural and sequencing data, as *Remispora galerita* did not group with the type species *Remispora maritima* (Sakayaroj et al. 2011).

#### Tubakiella

Taxon classificationFungiMicroascalesHalosphaeriaceae

K.L. Pang & E.B.G. Jones, Fungal Diversity 46: 97 (2011)

0F8BBA1F-19BF-524F-ADCD-F9DAE0B50EE9

Index Fungorum: IF518772

##### Description.

Saprobic on intertidal, drift and submerged wood. **Sexual morph. *Ascomata*** globose, subglobose to pyriform, superficial to immersed, ostiolate, papillate, coriaceous, thin-walled, hyaline to yellowish or light brown. ***Asci*** ellipsoidal or ovoid, thin-walled, deliquescing at maturity. ***Ascospores*** ellipsoidal, 1-septate, hyaline, thick-walled at the apices; ascospore appendages at first a gelatinous sheath covers ascospores completely, later only a subglobose subgelatinous appendages. **Asexual morph**. Undetermined.

##### Type species.

*Tubakiella galerita* (Tubaki) Sakayaroj, K.L. Pang & E.B.G. Jones, Fungal Diversity 46: 99 (2011).

##### Notes.

Sakayaroj et al. (2011) introduced *Tubakiella* to accommodate *Remispora galerita*, a monotypic genus. The genus remains monotypic and is accepted in all major classifications (Jones et al. 2015, 2019; Marharachchikumbura et al. 2015).

*Tubakiella* is widely distributed in France, Denmark, Germany, Japan, Mexico, Spain, Taiwan, USA, and Yugoslavia (Jones et al. 2009). *Tubakiella* showed different appendage morphology with other genera in the Halosphaeriaceae. It has appendages formed by fragmentation of an exosporic layer and morphologically has little in common with either of the associated species.

##### Molecular evaluation.

In the phylogenetic tree they were placed close to *Nautosphaeria cristaminuta* (sister clade) in a side branch apart from most Halosphaeriaceae (Fig. 4). Divergence-time analysis indicates that *Tubakiella* and *Nautosphaeria* separated during the Jurassic–Early Cretaceous (~146 MYA, 95% CI: 58–370.3 MYA) (Fig. 5).

#### Tubakiella galerita

Taxon classificationFungiMicroascalesHalosphaeriaceae

(Tubaki) Sakayaroj, K.L. Pang & E.B.G. Jones, Fungal Diversity 46: 99 (2011)

59979DA1-81A8-5559-B127-74D1DE625E90

Index Fungorum: IF518773

Fig. 23A, B

Remispora galerita Tubaki, Publs Setomar. biol. Lab. 15(5): 362 (1967). Basionym.
Halosphaeria galerita (Tubaki) I. Schmidt, Natur Naturschutz Mecklenburg 12: 49 (1974). Synonym.

##### Description.

Saprobic on wood. **Sexual morph. *Ascomata*** 100–280 µm diam., globose, subglobose to pyriform, superficial to immersed, ostiolate, papillate, coriaceous, thin-walled, hyaline to yellowish or light brown. ***Peridium*** 8–15 µm thick, composed of 3–4 layers of thick-walled cells. ***Necks*** 120–440 × 20–50 µm. ***Asci*** 56–96 × 20–45 µm, 8-spored, ellipsoidal or ovoid, thin-walled, deliquescing at maturity. ***Ascospores*** 20–28(–35) × 20–45 µm, ellipsoidal, 1-septate, hyaline, thick-walled at the apices; ascospore appendages at first a gelatinous sheath covers ascospores completely, later only a subglobose, subgelatinous appendages discernable. **Asexual morph**. Undetermined.

##### Material examined.

Denmark, • unidentified intertidal wood (BCC33500, type specimen).

##### Notes.

This species is saprobic on intertidal, drift and submerged wood, and on test panels immersed in the sea.

## Discussion

Using morphology and phylogenetic analyses of 18S and 28S rDNA and protein coding genes (RPB1, RPB2, TEF1-ɑ, MCM7), seven new genera (*Neoaniptodera*, *Neogesasha*, *Neohalosarpheia*, *Pangia*, *Remisporiopsis*, *Sheareromyces*, *Shiiraspora*), one new species *Ocostaspora japonica* and the following new combinations (*Halosarpheia australiensis*, *Halosphaeriopsis alopallonella*, *Neoaniptodera juncicola*, *Neogesasha mangrovei*, *Neohalosarpheia marina*, *Pangia limnetica*, *Remisporiopsis macrocephala*, *R. quadri-remis*, *R. spitsbergenensis*, *R. stellatus*, *R. submersa*, *Sheareromyces aquibella*, *Shiiraspora salsuginosa* and *Thalassogena unicellularis*) are introduced (Table 1). The number of genera in the family Halosphaeriaceae has increased from 15 (Kohlmeyer and Kohlmyer 1964) to 77 and 202 species (this study).

The systematics of the family Halosphaeriaceae has been problematic due to several reasons. Many genera are known only from the original description, have rarely been collected and are difficult to culture which has hindered a phylogenetic study to determine their placement in the family (for example, *Amphitrite*, *Bathyascus*, *Chadefaudia*, *Iwilsoniella* and *Luttrellia*). Further issues include quality of the sequences, especially in early studies, and with few genes sequenced for a number of species. Most species need to be recollected, isolated and a wider range of loci sequenced, including protein-coding genes.

Morphologically, genera of this family are dissimilar in ascospore shape, from taxa with oval, round, ellipsoidal, cylindrical, fusiform and filiform ascospores, with many having characteristic appendages, with few asexual morphs. Ascospore appendages fall into a number of morphological types:

1. Appendages hamate, unfurling in water to form long, thin, sticky threads (*Aniptodera*, *Halosarpheia*, *Natantispora*, *Trichomaris*, *Saagaromyces*, *Tirispora*);
2. Ellipsoidal ascospores with peritrichous polar and equatorial appendages formed by fragmentation of an exosporic spore wall layer (*Corollospora*);
3. Polar, strap-like ascospore appendages (*Haligena*);
4. Ascospores enveloped by a sheath which fragments to form threads still attached to the polar region (*Carbosphaerella*);
5. Polar, tube-like appendages releasing fine thread-like mucilage (*Toriella*);
6. Ascospores with a single amorphous, polar appendage (*Qarounispora*);
7. Polar and equatorial, spoon-like appendages which are outgrowths of the spore wall (*Halosphaeria*);
8. Ascospores with one polar tuft and four tufts equatorial hair-like appendages (*Nautosphaeria*, *Nereiospora*).

Although these morphological ascospore appendages are distinct, and do not fall into the same phylogenetic clades, for example those in group 1 fell into different clades (*Tirispora*, *Halosarpheia*, *Saagaromyces*) (Fig. 4). Likewise, those in group 8 (*Nautosphaeria*, *Nereiospora*) were distantly placed phylogenetically. The peritrichous genus *Corollospora*, with their polar and equatorial appendaged ascospores, formed a cohesive and characteristic group in a highly supported clade. This raises the question whether this morphological form arose many times in the evolution of the group, along with their adaptation to an arenicolous life style.

*Nautosphaeria*, *Tubakiella* and *Haligena* formed a basal clade within the Halosphaeriaceae, and may represent a different family in the Microascales. In a study of the taxonomical position of the genus *Marinosphaera*, with *Microascus trigonosporus* and *Petriella setifera* as the outgroup, they fell outside the Halosphaeriaceae (Samhita et al., unpublished results). Similarly, in Bao et al. (2023), *Nautosphaeria* and *Tubakiella* were excluded from Halosphaeriaceae and placed in Microascales genera *incertae sedis*, with *Dothidea insculpta* and *D. sambuci* as outgroup taxa. However, we retain these two genera in the Halosphaeriaceae as morphologically they possess features characteristic of the family, namely perithecial ascoma with necks, periphyses, catenophyses, unitunicate, early deliquescing asci lacking apical pores, and one septate, hyaline ascospores with appendages.

Halosphaeriaceae species occur widely on different substrates, are worldwide in distribution and play an important role in the recycling of complex organic material. They are often early colonizers of wood in the marine environment (e.g. *Ceriosporopsis halima*, *Lignincola laevis*) (Jones 1963) and are the source of a wide range of enzymes: cellulase (*Aniptodera salsuginosa*, *Lignincola laevis*), β-2,3-glucanase (*Corollospora maritima*), xylanase (*Bathyascus grandisporus*, *C. maritima*), alginase (*Corollospora intermedia*) (Velmurugan and Lee 2012). Many are active in the decay of wood causing soft rot decay: *Natantispora retorquens*, *Halosphaeria appendiculata*, *Marinospora longissima*, *Remispora maritima* etc. (Mouzouras 1986; Bucher et al. 2004). They are also the source of natural products: corollosporine (*Corollospora maritima*), puchellalactam, melinacidins III and ganacidin W (*Corollospora pulchella*), 7-hydroxyergosterol and a ceramide (*Lignincola laevis*), eniatin G (*Halosarpheia* sp.) and halosmysins B and C (Halosphaeriaceae sp. OUPS-135D-4) (Furuya et al. 1985; Abraham et al. 1994; Liberra et al. 1998; Lin et al. 2002; Yamada et al. 2022).

The origins of marine fungi are controversial but the most favoured pathway is from a terrestrial habitat to freshwater and marine habitats (Vijaykrishna et al. 2006). Divergent-time estimates for the origins of the Halosphaeriaceae is dependent on the calibrations used and thus yield differing figures: 280 MYA (Hyde et al. 2017), and 100–179 MYA (Bao et al. 2023). In this study, we estimate a divergence time of ~545 MYA (95% CI: 351–846 MYA), placing it in the Paleozoic period. Among the early diverging lineages are *Haligena* (~455 MYA) and *Nautosphaeria* (~400 MYA), with the genera *Halosphaeria*, *Lignincola*, and *Corollospora* diversifying much later (~129–196 MYA) in the Jurassic–Cretaceous. Taxa diversifying in the Cenozoic include *Shiiraspora* (~27 MYA) and *Pangia* (~2.5 MYA). The Halosphaeriaceae remains a fascinating family because of their morphology, physiology, and ecology, with most well adapted to life in the marine environment and a continually evolving group, e.g. the genus *Corollospora*.

## Additional information

### Artificial Intelligence (AI) use

The authors accept full responsibility for the content of the manuscript, including the disclosure of any use of AI.

No AI tools were used in the preparation of this manuscript.

### Author ORCIDs

E. B. G. Jones https://orcid.org/0000-0002-7286-5471

A. H. Bahkali https://orcid.org/0000-0003-3936-0436

B. Devadatha https://orcid.org/0000-0001-6400-5407

K. D. Hyde https://orcid.org/0000-0002-2191-0762

M. A. Abdel-Wahab https://orcid.org/0000-0002-3176-8675

M. S. Calabon https://orcid.org/0000-0002-6603-1876

M. S. Bakhit https://orcid.org/0000-0001-9510-1581

J. Sakayaroj https://orcid.org/0000-0001-7473-614X

C. Prematunga https://orcid.org/0009-0008-5521-5080

M. F. Caeiro https://orcid.org/0000-0003-0559-6344

E. Azevedo https://orcid.org/0000-0002-5057-8148

R. Asghari https://orcid.org/0009-0006-4897-5327

C.C.S. Apurillo https://orcid.org/0000-0003-4348-0887

A. T. Bhagya https://orcid.org/0009-0002-3988-298X

H. D. Aumentado https://orcid.org/0000-0003-2888-2669

C. Phukhamsakda https://orcid.org/0000-0002-1033-937X

S. S. Maharachchikumbura https://orcid.org/0000-0001-9127-0783

S.-Y. Guo https://orcid.org/0000-0002-5141-0697

K.-L. Pang https://orcid.org/0000-0003-4403-925X

### Data availability

All of the data that support the findings of this study are available in the main text or Supplementary Information.

## Supplementary materials

Supplementary material 1 GenBank accession numbers of the sequences used in phylogenetic analysis Data type docx https://binary.pensoft.net/file/1687612This dataset is made available under the Open Database License (http://opendatacommons.org/licenses/odbl/1.0/). The Open Database License (ODbL) is a license agreement intended to allow users to freely share, modify, and use this Dataset while maintaining this same freedom for others, provided that the original source and author(s) are credited. Monika C. Dayarathne, E. B. Gareth Jones, Ali H. Bahkali, Bandarupalli Devadatha, Kevin D. Hyde, Mohamed A. Abdel-Wahab, Mark S. Calabon, Pedro Correia, Faten A. Abdel-Aziz, Mahmoud S. Bakhit, Jariya Sakayaroj, Chasika Prematunga, Maria F. Caeiro, Egídia Azevedo, Raheleh Asghari, Carlo Chris S. Apurillo, Amuhenage Tharindu Bhagya, Herbert Dustin Aumentado, Samhita Mukhopadhyay, Chayanard Phukhamsakda, Yanpeng Chen, Sajeewa S. Maharachchikumbura, Sheng-Yu Guo, Ka-Lai Pang
