# Supplementary material for: Taxonomy and molecular evaluation of the Halosphaeriaceae (Microascales, Hypocreomycetidae)
Source: MycoKeys. 2026 Jun 25;135:1–202. doi: 10.3897/mycokeys.135.187540 (PMC13329432; doi:10.3897/mycokeys.135.187540)
Supplement: Supplementary material 1 — GenBank accession numbers of the sequences used in phylogenetic analysis [file mycokeys-135-001-s001.docx]

**Supplementary Table 1.** GenBank accession numbers of the sequences used in phylogenetic analysis.

| **Taxon** | **Isolate number** | **Gene region** | | | | | |
| --- | --- | --- | --- | --- | --- | --- | --- |
|  |  | **28S rDNA** | **18S rDNA** | **TEF1a** | **MCM7** | **RPB1** | **RPB2** |
| *Alisea longicolla* | CP2464b BF | EU118365 | EU118370 |  |  |  |  |
| *Alisea longicolla* | CP4167 | KX686751 | KX686750 |  |  |  |  |
| *Aniptodera chesapeakensis* | ATCC 32818 | U46882 | U46870 | DQ471064 |  | DQ471138 | DQ470896 |
| *Aniptosporopsis lignatilis* | NTOU3553 | KX686753 | KX686752 | PV573404 | PV573428 |  | PV573380 |
| *Antennospora quadricornuta* | GR89 | EF383130 |  |  |  |  |  |
| *Antennospora quadricornuta* | NTOU3763 | KX686757 | KX686756 | PV573405 | PV573429 |  | PV573381 |
| *Arenariomyces majusculus* | NTOU3989 | KX686759 | KX686758 | PV573406 | PV573430 | PV573451 | PV573382 |
| *Arenariomyces majusculus* | NTOU5689 | OQ130060 |  |  |  |  |  |
| *Arenariomyces parvulus* | SAT909 | KX686761 | KX686760 |  |  |  |  |
| *Arenariomyces trifurcatus* | NTOU3773 | KX686762 |  |  |  |  |  |
| *Arenariomyces trifurcatus* | NTOU3986 | PV539776 |  |  |  | PV573452 |  |
| *Arenariomyces triseptatus* | NTOU3778 | KX686764 | KX686763 | PV573407 | PV573431 | PV573453 | PV573383 |
| *Arenariomyces truncatellus* | NTOU3757 | KX686766 | KX686765 |  |  |  |  |
| *Ascoglobospora marina* | CBS H-23855 | NG_243128 | NG_242848 |  |  |  |  |
| *Ascoglobospora marina* | MAAW-2022a | OP151088 | OP150939 |  |  |  |  |
| *Ascosacculus aquaticus* | A444-1D | AY227136 |  |  |  |  |  |
| *Ascosacculus fusiformis* | MFLUCC 14-0036 | MK835842 | MK834779 |  |  |  |  |
| *Ascosacculus heteroguttulatus* | A108-11B | AY227122 |  |  |  |  |  |
| *Ascosacculus heteroguttulatus* | A108-7D | AY227121 | AY227138 |  |  |  |  |
| *Ceriosporopsis halima* | NTOU3876 | KX686768 | KX686767 | PV573408 | PV573432 |  | PV573384 |
| *Ceriosporopsis halima* | NTOU4001 | PV539777 |  |  |  |  |  |
| *Ceriosporopsis intricata* | BCC33465 | HQ111029 |  |  |  |  |  |
| *Ceriosporopsis intricata* | BCC33466 | HQ111031 |  |  |  |  |  |
| *Corollospora anglusa* | MD 827 | AB361008 |  |  |  |  |  |
| *Corollospora angusta* | NBRC 32101 | JN941477 | JN941668 |  |  | JN992398 |  |
| *Corollospora cinnamomea* | NBRC 32125 | AB361017 | JN941666 |  |  | JN992396 |  |
| *Corollospora cinnamomea* | NBRC 32126 | JN941480 | JN941665 |  |  | JN992395 |  |
| *Corollospora colossa* | NBRC 32103 | JN941481 | JN941664 |  |  | JN992394 |  |
| *Corollospora filiformis* | CBS:125146 | MH874958 |  |  |  |  |  |
| *Corollospora filiformis* | NTOU5709 | OQ130068 |  |  |  |  |  |
| *Corollospora fusca* | NBRC 32107 | JN941483 | JN941662 |  |  | JN992393 |  |
| *Corollospora gracilis* | NBRC 32110 | JN941486 | JN941660 |  |  | JN992390 |  |
| *Corollospora intermedia* | PP3910 | AF491258 |  |  |  |  |  |
| *Corollospora lacera* | NBRC 32121 | JN941488 | JN941658 |  |  | JN992388 |  |
| *Corollospora lacera* | NBRC 32122 | JN941489 | JN941657 |  |  | JN992387 |  |
| *Corollospora luteola* | NBRC 31315 | JN941490 | JN941656 |  |  |  |  |
| *Corollospora luteola* | NBRC 31316 | JN941491 | JN941655 |  |  |  |  |
| *Corollospora marina* | AFTOL-ID 5008 | FJ176898 | FJ176843 |  |  |  |  |
| *Corollospora maritima* | NTOU4008 | KX686770 | KX686769 | PV573409 | PV573433 | PV573454 | PV573385 |
| *Corollospora maritima* | NTOU4055 | PV539778 | PV539771 | PV573410 |  | PV573455 | PV573386 |
| *Corollospora mediterranea* | MUT:1950 | MW584963 | MW584972 | MW703376 |  | MW645217 | MW666026 |
| *Corollospora mediterranea* | MUT:5040 | MW584958 | MW584967 | MW703371 |  | MW645213 | MW666022 |
| *Corollospora parvula* | CBS 116644 | MH874556 |  |  |  |  |  |
| *Corollospora parvula* | TUB407 | AY762987 |  |  |  |  |  |
| *Corollospora portsaidica* | MD 832 | AB361016 |  |  |  |  |  |
| *Corollospora portsaidica* | MD1301 | KJ406564 |  |  |  |  |  |
| *Corollospora pseudopulchella* | NBRC 32112 | JN941494 | JN941652 |  |  | JN992384 |  |
| *Corollospora pseudopulchella* | NBRC 32113 | JN941495 | JN941651 |  |  | JN992383 |  |
| *Corollospora pulchella* | NBRC 32123 | JN941496 | JN941650 |  |  | JN992382 |  |
| *Corollospora pulchella* | NBRC 32124 | JN941497 | JN941649 |  |  | JN992381 |  |
| *Corollospora quinqueseptata* | NBRC 32114 | JN941498 | JN941648 |  |  | JN992380 |  |
| *Corollospora quinqueseptata* | NBRC 32115 | JN941499 | JN941647 |  |  | JN992379 |  |
| *Corollospora ramulosa* | NBRC 31325 | AB361020 |  |  |  |  |  |
| *Cucullosporella mangrovei* | NTOU3632 | PV539779 |  | PV573411 | PV573434 | PV573456 | PV573387 |
| *Cucullosporella mangrovei* | NTOU3744 | KX686772 | KX686771 |  |  |  |  |
| *Cucurbitinus constrictus* | CGMCC3.19640 | MN431420 | MN437321 |  |  |  |  |
| *Cucurbitinus ibericus* | FMR 12149 | KY853496 |  |  |  |  |  |
| *Ebullia octonae* | NTOU3882 | KC692149 | KC692148 |  |  |  |  |
| *Ebullia octonae* | NTOU3884 | KC692151 | KC692150 | PV573412 | PV573435 | PV573457 | PV573388 |
| *Gesasha peditatus* | n/a | HM570067 |  |  |  |  |  |
| *Arenariomyces salinus* | NTOU3996 | PV539780 | PV539772 | PV573413 | PV573436 | PV573458 | PV573389 |
| *Arenariomyces salinus* | NTOU3998 | KX686774 | KX686773 | PV573414 | PV573437 | PV573459 | PV573390 |
| *Haligena elaterophora* | NTOU3831 | KX686776 | KX686775 | PV573415 | PV573438 | PV573460 | PV573391 |
| *Haligena elaterophora* | PP4705 | AY864845 |  |  |  |  | HQ111040 |
| *Halosarpheia australiensis* | NTOU6106 | PV539781 | PV539773 |  |  |  |  |
| *Halosarpheia fibrosa* | JK 5166A | U46886 | U48422 |  |  |  | KT225543 |
| *Halosarpheia fibrosa* | NTOU3992 | KX686778 | KX686777 |  |  |  |  |
| *Halosarpheia japonica* | IMI 397961 | HQ009884 | HQ009885 |  |  |  |  |
| *Halosarpheia japonica* | IMI 397962 | HQ009886 | HQ009887 |  |  |  |  |
| *Halosarpheia trullifera* | CBS 253.64 | MH870061 |  |  |  |  |  |
| *Halosarpheia trullifera* | PP4268 | AF396875 |  |  |  |  |  |
| *Halosarpheia unicellularis* | CY2980 | AF396876 |  |  |  |  |  |
| *Halosphaeria appendiculata* | CBS:197.60 | MH869504 | U46872 | FJ238390 |  |  |  |
| *Halosphaeria appendiculata* | NTOU4004 | KX686782 | KX686781 |  |  | PV573461 | PV573392 |
| *Halosphaeriopsis alopallonella*  (= *Trichocladium alopallonella*) | NTOU1288 | PV539782 | PV539774 | PV573416 | PV573439 | PV573462 |  |
| *Halosphaeriopsis mediosetigera* | NTOU3816 | KX686784 | KX686783 |  |  |  |  |
| *Havispora longyearbyenensis* | CY5278 | HQ111023 |  |  |  |  |  |
| *Jinshana tangtangiae* | F0036020 | OQ418019 | OQ418018 |  |  |  |  |
| *Kochiella crispa* | BCC33502 | HQ111020 |  |  |  |  |  |
| *Kochiella crispa* | BCC33504 | HQ111018 |  |  |  |  |  |
| *Lignincola laevis* | JK 5180A | U46890 | U46873 |  |  |  | DQ836886 |
| *Lignincola laevis* | NTOU699 | PV539783 |  |  |  | PV573463 |  |
| *Lignincola tropica* | PP 7777 | AF539474 |  |  |  |  |  |
| *Magnisphaera spartinae* | A221-1C | AY227129 | AF352076 |  |  |  |  |
| *Magnisphaera spartinae* | A330-1A | AY227130 |  |  |  |  |  |
| *Magnisphaera stevemossago* | A409-1B | AY227134 | AY227140 |  |  |  |  |
| *Magnisphaera stevemossago* | CBS 139776 | KT278704 | KT278691 |  |  |  | KT278740 |
| *Marinospora calyptrata* | BBH28307 | HQ111035 |  |  |  |  | HQ111042 |
| *Marinospora calyptrata* | CY3491 | HQ111036 |  |  |  |  |  |
| *Marinospora longissima* | PP0868 | AF491266 |  |  |  |  |  |
| *Microascus trigonosporus* | AFTOL-ID 914 | DQ470958 | DQ471006 | DQ471077 |  | DQ471150 | DQ470908 |
| *Moana turbinulata* | NTOU3806 | KX686786 | KX686785 | PV573417 | PV573440 | PV573464 | PV573393 |
| *Morakotiella salina* | BCC12781 | AY864844 |  |  |  |  | HQ111039 |
| *Morakotiella salina* | NTOU185 | KX686788 | KX686787 | PV573418 | PV573441 | PV573465 | PV573394 |
| *Naïs inornata* | ATCC 200453 | AF539476 |  |  |  |  |  |
| *Natantispora lotica* | A214-3A | AY227123 | AF352079 |  |  |  |  |
| *Natantispora lotica* | A333-1A | AY227124 | AF352080 |  |  |  |  |
| *Natantispora retorquens* | A231-1D | AY227128 | AF352086 |  |  |  |  |
| *Natantispora retorquens* | ATCC38867 | AY227127 | AF352087 |  |  |  |  |
| *Natantispora unipolaris* | F27870 | KM624522 | KM624521 |  |  |  |  |
| *Nautosphaeria cristaminuta* | BBH28308 | HQ111009 |  |  |  |  |  |
| *Nautosphaeria cristaminuta* | NTOU4051 | KX686792 | KX686791 | PV573419 | PV573442 | PV573466 | PV573395 |
| *Neoaniptodera juncicola* | 5522F |  | U43845 |  |  |  |  |
| *Neogesasha mangrovei* | n/a | HM570069 |  |  |  |  |  |
| *Neohalosarpheia marina* | JK 5103B | AY227125 | AF352082 |  |  |  |  |
| *Neptunella longirostris* | PP4563 | AF539472 |  |  |  |  |  |
| *Neptunella longirostris* | PP4648 | AF539473 |  |  |  |  |  |
| *Nereiospora comata* | PP2520 | AF491267 |  |  |  |  |  |
| *Nereiospora comata* | PP3283 | AF491275 |  |  |  |  |  |
| *Nereiospora cristata* | PP5988 | AF491268 |  |  |  |  |  |
| *Nimbospora bipolaris* | NTOU3795 | KC692143 | KC692142 | PV573420 | PV573443 |  | PV573396 |
| *Nimbospora effusa* | JK 5104A | U46892 | U46877 |  |  |  | DQ836887 |
| *Nimbospora effusa* | NTOU4018 | KX686794 | KX686793 | PV573421 | PV573444 |  | PV573397 |
| *Nohea delmarensis* | MF982 | HQ268017 |  |  |  |  |  |
| *Naufragella spinibarbata* | BCC33508 | HQ111033 |  |  |  |  |  |
| *Naufragella spinibarbata* | PP6886 | HQ111032 |  |  |  |  |  |
| *Nohea umiumi* | JK 5103F | U46893 | U46878 |  |  |  |  |
| *Nohea umiumi* | NTOU4006 | KX686796 | KX686795 | PV573422 | PV573445 | PV573467 | PV573398 |
| *Oceanitis abyssalis* | 6K1555-W2 | LC789975 | LC789976 |  |  |  |  |
| *Oceanitis cincinnatula* | A318-1C | AY227120 | AF352077 |  |  |  |  |
| *Oceanitis cincinnatula* | CY1061 | AY150220 |  |  |  |  |  |
| *Oceanitis scuticella* | CP4157 | KX686798 | KX686797 |  |  |  |  |
| *Oceanitis scuticella* | M0229768 | KU712509 |  |  |  |  |  |
| *Oceanitis unicaudata* | CY1333 | AY150222 |  |  |  |  |  |
| *Oceanitis viscidula* | ATCC24310 | AY150223 |  |  |  |  |  |
| *Oceanitis viscidula* | PP0218 | AY227131 |  |  |  |  |  |
| *Ocostaspora apilongissima* | LP53 | HQ111005 |  |  |  |  |  |
| *Ocostaspora apilongissima* | NTOU4061 | KX686800 | KX686799 | PV573423 | PV573446 | PV573468 | PV573399 |
| *Ocostaspora japonica* | NBRC 105291 | PV535940 | PV535939 |  |  |  |  |
| *Okeanomyces cucullatus* | LP67 | AY490787 |  |  |  |  |  |
| *Okeanomyces cucullatus* | NTOU778 | KX686802 | KX686801 | PV573424 | PV573447 | PV573469 | PV573400 |
| *Okeanomyces guttulatus* | CGMCC 3.22360 | OQ758153 | OQ758186 |  |  |  |  |
| *Okeanomyces guttulatus* | CGMCC 3.22373 | OQ758155 | OQ758188 |  |  |  |  |
| *Okeanomyces marinus* | MFLUCC:20-0123 | MT068207 | MT509714 |  |  |  |  |
| *Ondiniella torquata* | BCC33480 | HQ111037 |  |  |  |  |  |
| *Ondiniella torquata* | BCC34303 | HQ111038 |  |  |  |  |  |
| *Ophiodeira monosemeia* | JK5164A | U46894 | U46879 |  |  |  |  |
| *Pangia limnetica* | NBRC 32471 | LC868236 |  |  |  |  |  |
| *Pangia limnetica* | NBRC 32472 | LC868237 |  |  |  |  |  |
| *Panorbis viscosus* | A231-2B | AY094184 | AF352084 |  |  |  |  |
| *Panorbis viscosus* | K5380A | AY227133 |  |  |  |  |  |
| *Paraaniptodera longispora* | BBH28304 | HQ111008 |  |  |  |  |  |
| *Paraaniptodera longispora* | NTOU3670 | KX686755 | KX686754 |  |  |  |  |
| *Petriella setifera* | AFTOL-ID 956 | DQ470969 | DQ471020 | DQ836911 |  | DQ842034 | DQ836883 |
| *Phaeonectriella alba* | AUMC-12004-H | MG383396 |  |  |  |  |  |
| *Phaeonectriella lignicola* | PP7008 | AY150224 |  |  |  |  |  |
| *Pileomyces formosanus* | BBH30192 | KX686804 | KX686803 | PV573425 | PV573448 | PV573470 | PV573401 |
| *Praelongicaulis kandeliae* | CY1492 | HQ111025 |  |  |  |  |  |
| *Praelongicaulis kandeliae* | NTOU3698 | KX686780 | KX686779 | PV573426 | PV573449 | PV573471 | PV573402 |
| *Pseudolignincola siamensis* | IT41 | DQ237873 | DQ237872 |  |  |  |  |
| *Pseudolignincola siamensis (anamorph)* | IT110 | DQ237875 | DQ237874 |  |  |  |  |
| *Qarounispora grandiappendiculata* | SUMCC H-17009 | OK043820 | OK043819 |  |  |  |  |
| *Remispora maritima* | BBH28309 | HQ111012 | HQ111002 |  |  |  | HQ111041 |
| *Remispora pilleata* | BBH28305 | HQ111021 |  |  |  |  |  |
| *Remispora pilleata* | BBH28306 | HQ111022 |  |  |  |  |  |
| *Remisporiopsis macrocephala* | MUCL 15736 | AY856915 | AY856954 |  |  |  |  |
| *Remisporiopsis quadri-remis* | BCC15555 | HQ111010 |  |  |  |  |  |
| *Remisporiopsis quadri-remis* | CBS:334.62 | MH869762 |  |  |  |  |  |
| *Remisporiopsis spitsbergenensis* | CY5279 | HQ111011 |  |  |  |  |  |
| *Remisporiopsis stellatus* | 3129J | KM272364 |  |  |  |  |  |
| *Remisporiopsis stellatus* | CBS:258.60 | MH869531 |  |  |  |  |  |
| *Remisporiopsis submersa* | CMG 53 | MT235738 |  |  |  |  |  |
| *Saagaromyces abonnis* | NTOU3618 | KT159904 | KT159900 |  |  |  |  |
| *Saagaromyces abonnis* | NTOU3995 | PV539784 |  |  |  |  |  |
| *Saagaromyces glitra* | NTOU3988 | PV539785 |  |  |  |  |  |
| *Saagaromyces glitra* | PP4672 | AF539475 |  |  |  |  |  |
| *Saagaromyces ratnagiriensis* | CY0732 | AF539470 |  |  |  |  |  |
| *Saagaromyces ratnagiriensis* | UM27 | KX686806 | KX686805 | PV573427 | PV573450 | PV573472 | PV573403 |
| *Sablicola chinensis* | BCC22809 | HQ111024 |  |  |  |  |  |
| *Safagamyces marinus* | SUMCC H-20001 | ON244695 |  |  |  |  |  |
| *Sheareromyces aquibella* | MFLU 15-1140 | KU556854 | KU556853 |  |  |  |  |
| *Sheareromyces aquibella* | MFLUCC 15-0605 | NG_057100 | NG_063599 |  |  |  |  |
| *Shiiraspora salsuginosa* | NBRC 32577 | LC831801 |  |  |  |  |  |
| *Shiiraspora salsuginosa* | NBRC 32578 | LC831802 |  |  |  |  |  |
| *Thalassogena unicellularis* | n/a | HM570068 | HM570066 |  |  |  |  |
| *Thalespora appendiculata* | IT200 | DQ237877 | DQ237876 |  |  |  |  |
| *Tinhaudeus formosanus* | NTOU3580 | KT159898 | KT159896 |  |  |  |  |
| *Tinhaudeus formosanus* | NTOU3805 | KT159899 | KT159897 |  |  |  |  |
| *Tirispora unicaudata* | CY2370 | AY150225 |  |  |  |  |  |
| *Toriella tubulifera* | BCC33511 | HQ111026 |  |  |  |  |  |
| *Toriella tubulifera* | BCC33512 | HQ111027 |  |  |  |  |  |
| *Tubakiella galerita* | BCC33500 | HQ111014 | HQ111003 |  |  |  |  |
| *Tubakiella galerita* | NTOU4012 | PV539786 | PV539775 |  |  | PV573473 |  |
